# Blood metabolites reflect the effect of gut microbiota on differentiated thyroid cancer: a Mendelian randomization analysis

**DOI:** 10.1186/s12885-025-13598-y

**Published:** 2025-02-28

**Authors:** Hanfei Zhang, Yuhao Li, Lin Li

**Affiliations:** Department of Nuclear Medicine, West China Hospital, Sichuan University, No. 37, Guo Xue Xiang, Chengdu, 610041 China

**Keywords:** Papillary thyroid cancer, Follicular thyroid cancer, Blood metabolites, Gut Microbiota, Mendelian randomization

## Abstract

**Background:**

Studies have linked gut microbiome and differentiated thyroid cancer (DTC). However, their causal relationships and potential mediating factors have not been well defined. Our study investigated the causal relationships between the gut microbiome, papillary thyroid cancer (PTC) and follicular thyroid cancer (FTC), as well as the mediating effect of potential blood metabolites, using genetic approaches.

**Methods:**

Leveraging the summary statistics of gut microbial taxa, blood metabolites, PTC and FTC from the largest genome-wide association studies (GWAS) to date, we applied the bidirectional and mediation Mendelian randomization (MR) design. The multivariable MR approach based on Bayesian model averaging (MR-BMA) was used to prioritize the most likely causal taxa. Furthermore, metabolic pathway analysis was performed via the web-based Metaconflict 4.0.

**Results:**

After sensitivity analyses, we identified 4 taxa, 19 blood metabolites, and 5 gut bacterial pathways were causally associated with PTC. Similarly, 3 taxa, 31 blood metabolites, and 3 gut bacterial pathways were found to be causally associated with FTC, with 2 blood metabolites exhibiting bidirectional causal relationships. Metabolic pathway analysis revealed 8 significant pathways in PTC and FTC. MR-BMA analysis pinpointed species *Bifidobacterium longum* as the primary causal taxon for PTC and genus *Bacteroides* for FTC. The mediation MR analysis showed that sphingomyelin (d18:2/23:0, d18:1/23:1, d17:1/24:1) and 2-hydroxysebacate mediated the causal effects of specific gut microbiota on PTC and FTC, respectively.

**Conclusion:**

The study suggested a causal relationship between several gut microbial taxa and DTC, and that specific blood metabolites might mediate this relationship.

**Supplementary Information:**

The online version contains supplementary material available at 10.1186/s12885-025-13598-y.

## Introduction

Thyroid cancer (TC) is classified as an endocrine malignancy and ranks as the fifth most common cancer globally. Differentiated thyroid cancer (DTC), which includes papillary thyroid cancer (PTC) and follicular thyroid cancer (FTC), accounts for over 90% of all pathological diagnoses in this category [1]. Compelling evidence suggests that the gut microbiome is a risk or preventive factor for various diseases, including cancer, and is closely related to systemic treatment responses [2–5]. Furthermore, the role of the gut microbiome in the link between modifiable factors, such as inflammatory responses and diet, has been widely recognized, partly due to the accumulation of metabolites from probiotic communities [6, 7]. The concept of the "thyroid-gut axis" has been proposed to explore the relationship between thyroid cancer and the gut microbiome [8, 9]. Therefore, elucidating the causal relationships between the gut microbiome, metabolites, and DTC is crucial. This may provide new perspectives for microbiome-based prevention and targeted interventions for DTC.

The gut microbiota is associated with changes in the levels of certain bioactive metabolites in the blood. Previous studies have shown notable alterations in the serum concentrations of gamma-aminobutyric acid, phospholipids, and linolenic acid in patients with TC [10, 11]. Additionally, changes in the levels of lipids (specifically, linolenic acid and sphingomyelin), galactinol, and inosine were identified through metabolic profiling of TC tissue [12, 13]. Several cohort studies have identified distinct gut microbiota compositions and functions in patients with TC compared to healthy controls [14, 15]. Moreover, some MR studies have revealed a causal relationship between TC, including DTC, and gut microbiota composition [16–18]. However, these studies did not investigate the underlying mechanisms of this relationship. Furthermore, DTC comprises two histological types, papillary carcinoma and follicular carcinoma, each with different characteristics regarding predisposing populations, metastatic features, prognosis, genetic mutations, and other factors. The causal relationship between gut microbiota and specific subtypes of tumor tissue remains unclear.

In this study, we used a two-sample bidirectional and mediation MR design to evaluate the causal relationship between gut microbiota and DTC, including PTC and FTC, as well as the potential role of blood metabolites as mediators. Importantly, we utilized linkage disequilibrium score regression (LDSC) to estimate the genetic correlation between the gut microbiome and DTC, and conducted multivariable MR analysis using Bayesian model averaging (MR-BMA) to identify the most likely causal microbiota. Our research aims to explore the etiological basis of DTC and gut microbiota from a metabolomics perspective, with the goal of enabling early intervention and more precise treatment for patients.

## Materials and methods

### Study design

**Fig. 1:**
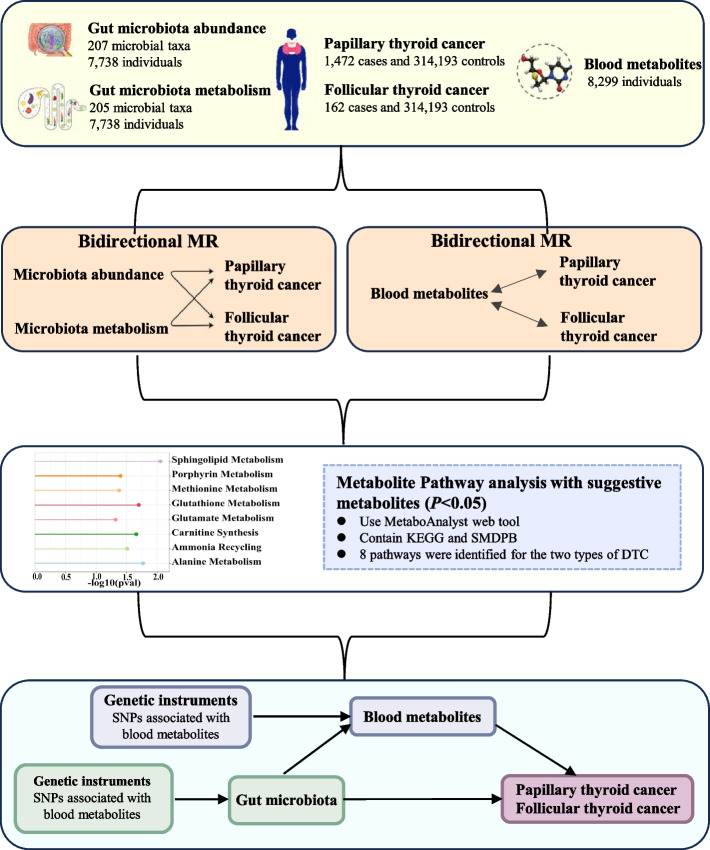
Study design and flowchart. Footnote: MR, Mendelian randomization; DTC, Differentiated thyroid cancer

We conducted a systematic assessment of the causal relationship between gut microbiota composition, blood metabolites, and DTC using bidirectional two-sample, multivariable, and two-step MR design. A robust MR study should adhere to 3 key assumptions: (1) genetic instruments should be directly associated with the exposure, (2) genetic instruments should not be associated with the outcome and should be independent of any potential confounders, and (3) the effects of IVs on the outcomes should be solely mediated by the exposures of interest. We completed the STROBE-MR checklist for this observational study (Additional file 1). The research design is illustrated in Fig. 1.

### Data source

The summary statistics for gut microbiome, metabolites, and DTC were derived from prior GWAS studies, as detailed in Additional file 2: Table S1.

Data for bacterial taxa were acquired from a GWAS encompassing 7,738 individuals of European descent, part of the Dutch Microbiome Project (DMP). The characterization of the gut microbiome was conducted through shotgun metagenomic sequencing of stool samples. A total of 207 microbial taxa (5 phyla, 10 classes, 13 orders, 26 families, 48 genera, and 105 species) and 205 functional pathways were included in this study.

Data on blood metabolite measurements were extracted from extensive GWAS, encompassing 1,091 metabolites and 309 metabolite ratios in 8,299 individuals from the Canadian Longitudinal Study on Aging (CLSA). Out of the 1,091 plasma metabolites examined, 850 were identified within eight superpathways: lipid, amino acid, xenobiotics, nucleotide, cofactor and vitamins, carbohydrate, peptide, and energy. The remaining 241 metabolites were classified as either unknown or 'partially' characterized molecules.

Data on PTC and FTC were obtained from the FinnGen consortium R10 release data. This study included 314,355 individuals of Finnish descent, comprising 1,472 cases and 314,193 controls for PTC, and 162 cases and 314,193 controls for FTC. The subtypes of thyroid cancer were defined based on International Classification of Diseases (ICD) codes retrieved from the Finnish National Registry.

### Genetic instrumental variable selection

We utilized genetic variants of microbiota taxa meeting the GWAS testing P-value threshold (< 1 × 10^−5^) and effect allele frequency (EAF) > 0.01 as selection criteria. The instrumental variable inclusion threshold of *P* < 1 × 10^−5^ was determined by maximizing the genetic variance explained by genetic predictors, consistent with prior gut microbiome MR studies [19]. Genetic instrumental variable selection for blood metabolites adhered to a genome-wide significance P-value threshold (< 5 × 10^−8^). Subsequently, independent SNPs were then clumped to a linkage disequilibrium (LD) threshold of r2 < 0.001 within ± 10 000 kb distance at 1000 Genomes reference panel [20]. In cases where no shared SNPs existed between the exposure and outcome, proxies from the 1000 Genomes European reference panel (r2 ≥ 0.8) were incorporated. Lastly, SNPs with an F-statistic < 10 (indicating weak instrumental strength) were excluded to mitigate weak instrumental bias [21].

### Statistical analysis

The inverse-variance weighted (IVW) method, serving as the primary MR method, was employed for causal estimation. A two-sided P-value that passed the Bonferroni correction threshold was deemed statistically significant: 2.4 × 10^–4^ (0.05/207) for microbial taxonomy and 3.6 × 10^–5^ (0.05/1400) for blood metabolites. Associations with *P* < 0.05 were considered suggestively significant. The Cochran’s Q test within the IVW approach was employed to examine heterogeneity among the included SNPs in each analysis. Sensitivity analyses were conducted using CML-MA, RAPS, and weighted median methods. Furthermore, the MR pleiotropy residual sum and outlier (MR-PRESSO) method identified potential outliers and compute causal estimates post the removal of these outliers [22]. Additionally, Steiger filtering excluded variants with stronger associations with the outcome than with the exposure. To illustrate the genetic correlation among DTC and gut microbiota, we conducted bivariate linkage disequilibrium score regression (LDSC) using GWAS summary statistics.

Given the substantial correlation among microbial taxa, MR-BMA, an extension of multivariable MR, was employed to identify and prioritize candidate risk factors with shared genetic predictors using a Bayesian framework, as described previously [23]. The marginal inclusion probability (MIP) was calculated to indicate the likelihood of each taxon being a causal determinant of disease risk. Additionally, we computed the model-averaged causal effect, representing the average causal effect of each taxon on DTC across models.

Metabolic pathways were assessed using the online platform Metaconflict 4.0 (https://www.metaboanalyst.ca/) [24]. Functional enrichment and pathway analysis modules were employed to identify potential metabolite groups or pathways relevant to the biological processes of DTC. This study utilized two repositories, namely the Small Molecule Pathway Database (SMPDB) and the Kyoto Encyclopedia of Genes and Genomes (KEGG) database, for pathway analysis, setting the significance level at 0.10.

A two-step MR analysis was conducted to assess the mediating influence of blood metabolites on the connections between gut microbiota and DTC. Estimation of the proportion of the total effect mediated by blood metabolites was computed by dividing the indirect effect by the total effect (β1 × β2/β3). Here, β1 denotes the effect of gut microbiota on the metabolites, β2 signifies the effect of blood metabolites on DTC, and β3 represents the effect of gut microbiota on DTC. Standard errors were computed utilizing the delta method, and effect estimates were derived from the two-sample MR analysis.

Analysis was conducted in R version 4.3.1. Two-sample MR was performed using “TwoSampleMR” and “MRPRESSO” R packages, LDSC was conducted using “ldscr” R package, and MR-BMA was based on the R-code available on GitHub (https://github.com/verena-zuber/demo_AMD). Figures were produced using the “ggforestplot” and ‘circlize’ R packages.

## Results

### Selection of IVs

The detailed characteristics of SNPs associated with microbial taxa, blood metabolites, and PTC can be found in Additional file 2: Tables S2–S4, respectively. Due to the number of FTC cases was small, IVs were not extracted at the 5 × 10^−8^ threshold. The F statistics for all analyzed SNPs were greater than 10. Steiger filtering tests revealed no evidence of reverse causality among the genetic instruments used (Additional file 2: Tables S5–S6). Additionally, leave-one-out analyses (Figure S1-S4) and funnel plots (Figure S5-S8) ruled out the possibility of potential outliers and horizontal pleiotropy.

### Causal associations of microbial taxa with DTC

**Fig. 2:**
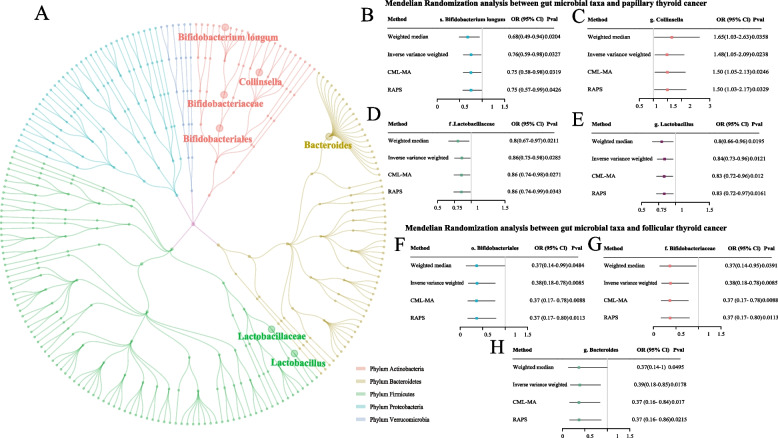
**A** The classification of microbial taxa included in the current study. Each dot represents a microbial taxon. The main branches represent different microbial phyla, and each phylum is shown in a different specific color. Within each main branch (the same phylum), there are additional branches representing further classifications as class, order, family, genus and species, from the inside to the outside. The suggestively significant taxa found in this study were labelled. **B**-**H** The forest plot shows the causal estimates between microbial taxa and papillary thyroid cancer or follicular thyroid cancer. The prefixes o., f., g. and s. in the taxa column indicated order, family, genus and species respectively. Footnote: OR, odds ratio; CI, confidence interval

Using the IVW method, we identified 11 and 7 microbial taxa potentially associated with PTC and FTC, respectively. After sensitivity analyzes, 4 taxa for PTC and 3 for FTC met the criteria. The results showed that species *Bifidobacterium longum* (OR = 0.76, 95% CI = 0.59–0.98, *P* = 0.032), genus *Collinsella* (OR = 1.48, 95% CI = 1.05–2.09, *P* = 0.0238), family *Lactobacillaceae* (OR = 0.86, 95% CI = 0.75–0.98, *P* = 0.0285), and genus *lactobacillus* (OR = 0.84, 95% CI = 0.73–0.96, *P* = 0.0121) had a causal effect on PTC. Besides, order *Bifidobacteriales* (OR = 0.38, 95% CI = 0.18–0.78, *P* = 0.0085), family *Bifidobacteriaceae* (OR = 0.38, 95% CI = 0.18–0.78, *P* = 0.0085), and genus *Bacteroides* (OR = 0.39, 95% CI = 0.18–0.85, *P* = 0.018) also had a suggestively causal effect on FTC (Fig. 2 and Additional file 2: Tables S7). Conversely, reverse MR indicated no bidirectional causal relationship between the 4 taxon and PTC (Additional file 2: Tables S8). We did not perform a reverse MR analysis for taxon and FTC due to the absence of extracted IVs for FTC. A test of pleiotropy using the MR-Egger intercept indicated that the P-values for the intercept were greater than 0.05, indicating no evidence of directional pleiotropy in these results.

We further included microbial taxa that showed potentially associations with DTC in the univariable MR analysis into the MR-BMA analysis. The top five models, ranked by their model posterior probability, are presented in Additional file 2: Table S9-S10, while Table 1 display the ranking of taxa according to their MIP. Species *Bifidobacterium longum* was identified as the top-ranked microbial taxa that increases the risk of PTC (MIP = 0.8551, MACE = 0.3436, and a Bonferroni-adjusted *P*-value of 0.045). Additionally, genus *Bacteroides* was identified as the top-ranked microbial taxa decreasing the risk of FTC (MIP = 0.8375, MACE = -0.5009, and a Bonferroni-adjusted P-value of 0.027). We also conducted a bivariate LDSC analysis of these taxa to evaluate their genetic correlations. The results revealed no significant genetic correlations between taxa and DTC (Additional file 2: Tables S11).

**Table 1:** Prioritization of causal microbial taxa of differentiated thyroid cancer using the MR-BMA method

Papillary thyroid cancer					Follicular thyroid cancer				
Exposure	MACE	MIP	Empirical *P*-value	Bonferroni-adjusted *P*-value	Exposure	MACE	MIP	Empirical *P*-value	Bonferroni-adjusted *P*-value
s. Bifidobacterium longum	0.3436	0.8551	0.0450	0.0450					
g. Lactobacillus	-0.3061	0.8531	0.0240	0.0320	g. Bacteroides	-0.5009	0.8375	0.0270	0.0270
g. Collinsella	-0.1020	0.6293	0.0040	0.0080	f. Bifidobacteriaceae	-0.3977	0.7222	0.0020	0.0030
f. Lactobacillaceae	-0.0984	0.6249	0.0030	0.0080	o. Bifidobacteriales	-0.3976	0.7221	0.0020	0.0030

Empirical *P*-values were computed using 1000 permutations. The prior probability and prior variance used in the MR-BMA analysis were 0.5 and 0.5

*MIP* marginal inclusion probability, *MACE* model-averaged causal effect

### Causal associations of blood metabolites with DTC

**Fig. 3:**
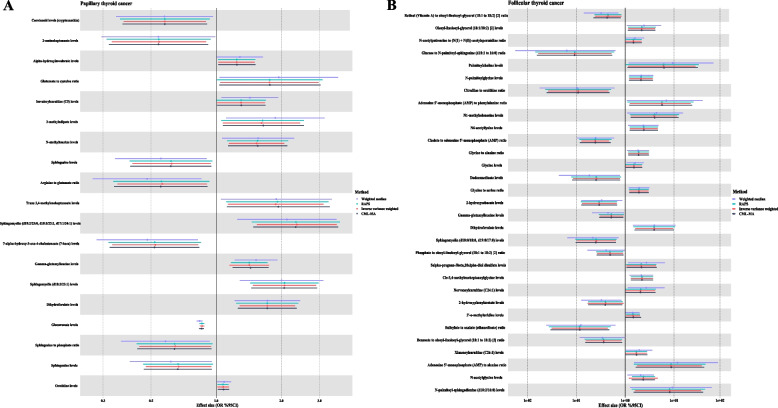
**A** Forest plot of the causal associations between blood metabolites and papillary thyroid cancer. **B** Forest plot of the causal associations between blood metabolites and follicular thyroid cancer. Footnote: Dots depict the point estimate. Horizontal bars depict 95% confidence interval (CI); RAPS, robust adjusted profile score; CML-MA, constrained maximum likelihood and model averaging

Consistent with sensitivity analyses, we identified 17 metabolites potentially related to PTC. Glucuronate levels and sphingomyelin (d18:2/23:1) levels were observed to have a significant causal effect on PTC after Bonferroni adjustment (OR = 0.86, 95% CI = 0.84–0.88, *P* = 1.4 × 10^–37^; OR = 2.07, 95% CI = 1.48–2.9, *P* = 2.37 × 10^–5^, respectively) (Fig. 3A; Additional file 2: Table S12). As for FTC, 30 metabolites were shown to have a suggestively causal effect, but only cholate to adenosine 5'-monophosphate (AMP) ratio was observed to have a significant causal effect after Bonferroni adjustment (OR = 0.24, 95% CI = 0.12–0.51, *P* = 2.36 × 10^–5^) (Fig. 3B; Additional file 2: Table S13). Additionally, we identified a bidirectional causal relationship between the arginine to glutamate ratio and PTC, as well as between the glutamate to cysteine ratio and PTC (Additional file 2: Table S14). No horizontal pleiotropy was identified using the MR-Egger method.

### Analysis of metabolic and gut bacterial pathways

**Fig. 4:**
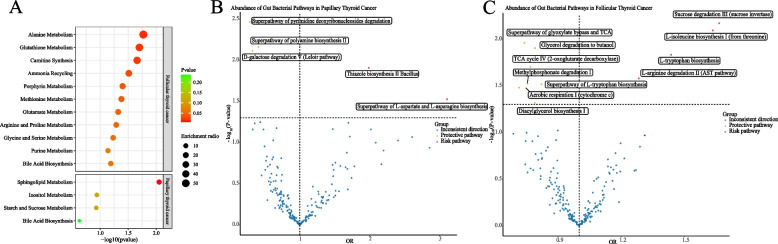
**A** Enriched significant metabolic pathways of papillary thyroid cancer and follicular thyroid cancer. **B** Volcano plot showing the causal estimates of 205 microbiota metabolism pathways on papillary thyroid cancer with inverse–variance weighted method. **C** Volcano plot showing the causal estimates of 205 microbiota metabolism pathways on follicular thyroid cancer with inverse–variance weighted method

The metabolic pathway analysis identified 8 significant metabolic pathways in differentiated thyroid cancer (Fig. 4A). Our results show that the “sphingolipid metabolism” (*p* = 0.009) pathway was found to be associated with the pathogenetic process of PTC, whereas the pathways for “alanine metabolism”, “glutathione metabolism”, “carnitine synthesis”, “ammonia recycling”, “porphyrin metabolism”, “methionine metabolism”, and “glutamate metabolism” were associated with FTC (Table 2).

**Table 2:** Metabolic pathways involved in differentiated thyroid cancer

**Cancer**	**Metabolite set**	**Total**	**Expect**	**Hits**	**Enrichment radio**	***P*val**
Papillary thyroid cancer	Sphingolipid Metabolism	40	0.160	2	12.50	0.009
Papillary thyroid cancer	Inositol Metabolism	30	0.120	1	8.33	0.115
Papillary thyroid cancer	Starch and Sucrose Metabolism	31	0.124	1	8.06	0.118
Papillary thyroid cancer	Bile Acid Biosynthesis	65	0.259	1	3.86	0.236
Follicular thyroid cancer	Alanine Metabolism	17	0.017	1	58.82	0.017
Follicular thyroid cancer	Glutathione Metabolism	20	0.020	1	50.00	0.020
Follicular thyroid cancer	Carnitine Synthesis	22	0.022	1	45.45	0.022
Follicular thyroid cancer	Ammonia Recycling	31	0.031	1	32.36	0.031
Follicular thyroid cancer	Porphyrin Metabolism	40	0.040	1	25.06	0.040
Follicular thyroid cancer	Methionine Metabolism	42	0.042	1	23.87	0.042
Follicular thyroid cancer	Glutamate Metabolism	48	0.048	1	20.88	0.048
Follicular thyroid cancer	Arginine and Proline Metabolism	52	0.052	1	19.27	0.052
Follicular thyroid cancer	Glycine and Serine Metabolism	59	0.059	1	16.98	0.059
Follicular thyroid cancer	Bile Acid Biosynthesis	65	0.065	1	15.41	0.065
Follicular thyroid cancer	Purine Metabolism	73	0.073	1	13.72	0.073
Enrichment Ratio is computed by Hits / Expect						

Based on the IVW method, we identified that an increased abundance of 12 bacterial metabolism pathways had causal effects on PTC and 5 pathways had causal effects on FTC (Fig. 4B, C). After conducting sensitivity analysis, we found that certain metabolic pathways exhibited causal effects on PTC and FTC. Specifically, L-isoleucine biosynthesis I (from threonine), sucrose degradation III (sucrose invertase), glycerol degradation to butanol, the superpathway of glyoxylate bypass and TCA, and L-tryptophan biosynthesis were found to have causal effects on PTC, while the superpathway of L-aspartate and L-asparagine biosynthesis, thiazole biosynthesis II Bacillus, and D-galactose degradation V (Leloir pathway) were found to have causal effects on FTC (Additional file 2: Table S15-S16).

### Mediation analysis of microbiota, blood metabolites, and DTC

**Fig. 5:**
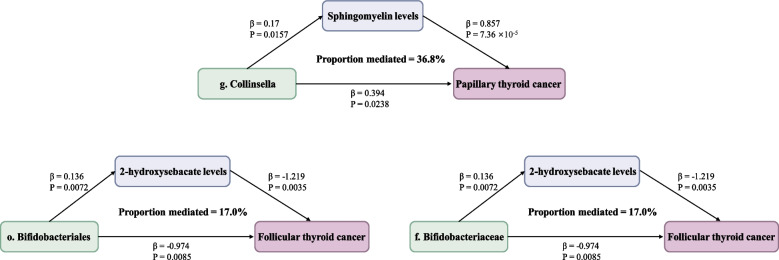
The specific blood metabolites mediated the causal effect of microbial taxa on papillary thyroid cancer or follicular thyroid cancer. Footnote: The β value between microbial taxa and metabolites and cancer are the MR estimates using the inverse–variance weighted method. Mediation effect was derived by using the delta method

Among the 50 blood metabolites that were causally associated with DTC, 5 were significantly associated with the above 7 taxa using IVW method (Additional file 2: Table S17). Two-step MR analyses were conducted on these 5 potential mediators, including sphingomyelin, Alpha-hydroxyisovalerate, 2-hydroxysebacate, Cis-3,4-methyleneheptanoylglycine, and 5alpha-pregnan-3beta 20alpha-diol disulfate, using the delta method to approximate the confidence intervals. Overall, we observed effects of sphingomyelin (d18:2/23:0, d18:1/23:1, d17:1/24:1) in the associations between the genus *Collinsella* and PTC, with a mediated proportion of 36.84% (95% CI = 23.03–50.65%, *P* = 0.039); and 2-hydroxysebacate in the association between the order *Bifidobacteriales* and the family *Bifidobacteriaceae* within the order *Bifidobacteriales* and FTC, with a mediated proportion of 17.01% (95% CI = 0.61–33.41%, *P* = 0.048) (Fig. 5; Additional file 2: Table S17).

## Discussion

In the present large-scale and comprehensive MR study, we identified that four microbial taxa were causally associated with the risk of PTC and three microbial taxa were causally associated with the risk of FTC after conducting sensitive analyses. Additionally, eight significant metabolic pathways and eight significant bacterial metabolism pathways involved in the 2 subtypes of DTC were detected. The MR-BMA results revealed species *Bifidobacterium longum* as the primary taxa associated with PTC and the genus *Bacteroides* as the primary taxa associated with FTC. Moreover, sphingomyelin (d18:2/23:0, d18:1/23:1, d17:1/24:1) may account for 36.84% of the causal effect of genus *Collinsella* on PTC, while 2-hydroxysebacate may mediate 17.01% of the causal effect of the family *Bifidobacteriaceae* or the order *Bifidobacteriales* on FTC. Our MR analysis on the relationship between gut microbiota, blood metabolites, and DTC has offered potential insights that may guide further developments in precision treatments.

Several previous MR studies investigated the alterations of the gut microbiota in patients with TC or DTC using the Mibiogen database, which encompasses 211 distinct gut microbiota taxa [16–18]. However, these studies have not yet delved deeply enough to identify specific microbial species and histological types of DTC. Additionally, the effects of the gut microbiota and its corresponding metabolites in individuals with DTC were not thoroughly screened. Feng et al. observed that the phylum *Firmicutes* and *Proteobacteria* increased in the TC cohort, while Yu et al. observed no significant difference between TCs and healthy controls at the phylum level [14, 15]. This disparity could arise from variations in tumor subtype, inadequate statistical power, and insufficient control for potential confounders. The MR study design offers a more dependable analysis by reducing the impact of confounding variables.

Our findings add to the evidence that species *Bifidobacterium longum* is the primary causal factor for PTC risk among microbial metrics. While microbiota exhibit correlations, our study's methodology, MR-BMA, can identify true causal risk factors even in situations where the candidate risk factors are strongly correlated. *Bifidobacterium longum* is a probiotic bacterium known for its ability to aid in food digestion, nutrient absorption, and immune system defense against illnesses. A previous study reported that administering methimazole alongside *Bifidobacterium longum* improved thyroid function in patients with Graves' disease by modulating the gut-thyroid axis [25]. The improvement in thyroid function may be achieved through several hypothetical mechanisms, including the promotion of short-chain fatty acids (SCFAs) secretion, regulation of abnormal levels of blood minerals, changes in bile acid, and dopamine concentrations [26–29]. *Bifidobacterium longum* has also been shown to inhibit carcinogen-induced colorectal tumorigenesis in rodents since the early 1980s [30, 31]. Meanwhile, reactivity toward *Bifidobacterium longum* demonstrated a robust CD8^+^ T cell response and better prognosis in HBV-related hepatocellular carcinoma [32]. However, research on the relationship between *Bifidobacterium longum* and PTC remains limited, and further investigation is needed to determine the causal effect.

The findings of our study suggest that targeting the gut microbiota genus *Bacteroides*, as a primary causal factor for FTC risk, could be a promising strategy for preventing FTC in clinical practice. One study has shown that the gut microbiomes of TC patients are characterized by a decrease in the genus *Bacteroides* compared to healthy controls [33]. Genus *Bacteroides* has been shown to promote the secretion of SCFAs, especially butyrate, which exerts a negative regulation on the NLRP3-mediated inflammatory signaling pathway [34]. This action inhibits the activation of macrophages and the secretion of pro-inflammatory mediators such as IL-18 and IL-1β. Colonization with *Bacteroides* has been demonstrated to effectively ameliorate intestinal epithelial cell damage induced by chronic inflammation and to inhibit the formation of colon tumors [34]. However, translating our research findings into clinical practice for FTC still requires further investigation into the underlying processes and mechanisms of gut microbiota dysbiosis in patients with FTC.

Our study identified glucuronate and sphingomyelin (d18:2/23:1) levels as causally associated with PTC after Bonferroni adjustment. Notably, the sphingolipid metabolism pathway was also found to be associated with the pathogenesis of PTC. Sphingolipids are essential structural components of cell membranes, typically composed of phosphatidylcholine and sphingomyelin, and play a pivotal role in regulating various biological processes [35, 36]. Previous studies have indicated that sphingolipids and their metabolite, sphingosine-1-phosphate, are involved in the occurrence and progression of PTC and can serve as predictive biomarkers for lymph node metastasis [37]. Our results further support these findings and highlight the importance of sphingolipid metabolism as a strategy for improving the health of PTC patients.

We identified sphingomyelin (d18:2/23:0, d18:1/23:1, d17:1/24:1) as a mediator in the causal link between genus *Collinsella* and PTC, while 2-hydroxysebacate as a mediator in the causal link between family *Bifidobacteriaceae* or order *Bifidobacteriales* and FTC. The interplay between the microbiome and blood metabolites has been elucidated through murine experiments and metagenomic sequencing analyses [38, 39]. Disruption of gut bacterial metabolic pathways correlates with the initiation and severe advancement of various human cancers, such as breast, ovarian, and colorectal cancers [40–42]. Moreover, a prior investigation indicated that cholesterol and 27-hydroxycholesterol might heighten the aggressiveness of TC [43]. However, the exact mechanisms through which the gut microbiome impacts blood metabolites remain elusive. Further investigation in this area has the potential to enhance our comprehension of the gut microbiome's involvement in DTC.

Our research indicates that personalized treatment, tailored according to gut microbiota profiles and blood metabolites, can enhance the precision and effectiveness of strategies to prevent DTC in clinical settings. This personalized approach may include various therapeutic methods, such as small-molecule antibiotics, engineered bacteria (both commensal and probiotic), prebiotics, and bioactive metabolites to modify gut microbiota composition [44, 45]. We believe further research is warranted to explore the potential role of flora such as *Bifidobacterium longum* and *Bacteroides*, and how they interact with other gut microbiota and host factors such as genetics, diet, and lifestyle.

This study had several limitations. Firstly, while MR offers a valuable approach for assessing causal effects, it is important to recognize that MR estimates reflect long-term genetic exposures and may not accurately capture the effects of short-term changes in the microbiome [19]. Secondly, the gut microbiome can be influenced by various factors, including demographics, diet, and medications. Many of these factors are characterized by heterogeneity, interindividual variability, and low heritability, which can reduce the statistical power and robustness of the findings [46]. Thirdly, the generalizability of our findings to specific ethnic groups needs to be confirmed, as our analysis primarily focused on European populations. The uneven distribution of genetic variants across different ethnic or racial groups can result in population stratification, potentially biasing the study outcomes.

## Conclusion

Our MR study identifies causal links between gut microbiota, blood metabolites, and DTC, including PTC and FTC. Specifically, *Bifidobacterium longum* is a primary causal factor for PTC risk, while *Bacteroides* is a key determinant for FTC risk. Furthermore, sphingomyelin and 2-hydroxysebacate mediate the effects of *Collinsella* and *Bifidobacteriaceae* on PTC risk, respectively. These findings could inform future clinical strategies and research in this area.

## Supplementary Information

Supplementary Material 1.

Supplementary Material 2.

Supplementary Material 3.

## Data Availability

No datasets were generated or analysed during the current study.

## Abbreviations

TC: Thyroid cancer
DTC: Differentiated thyroid cancer
PTC: Papillary thyroid cancer
FTC: Follicular thyroid cancer
GWAS: Genome-wide association studies
MR: Mendelian randomization
SNPs: Single-nucleotide polymorphisms
IVs: Instrumental variables
IVW: Inverse-variance weighted
MR-BMA: MR Bayesian model averaging analysis
MACE: Model-averaged causal effects
MIP: Marginal inclusion probability
LDSC: Linkage disequilibrium score regression
