# Supplementary material for: Blood metabolites reflect the effect of gut microbiota on differentiated thyroid cancer: a Mendelian randomization analysis
Source: BMC Cancer. 2025 Feb 28;25:368. doi: 10.1186/s12885-025-13598-y (PMC11869591; doi:10.1186/s12885-025-13598-y)
Supplement: Supplementary file 3 — Supplementary Material 3. [file 12885_2025_13598_MOESM3_ESM.zip › Figure S1 Leave-one-out analysis for MR causal effects of microbiota abundance and metabolism on PTC.pdf]

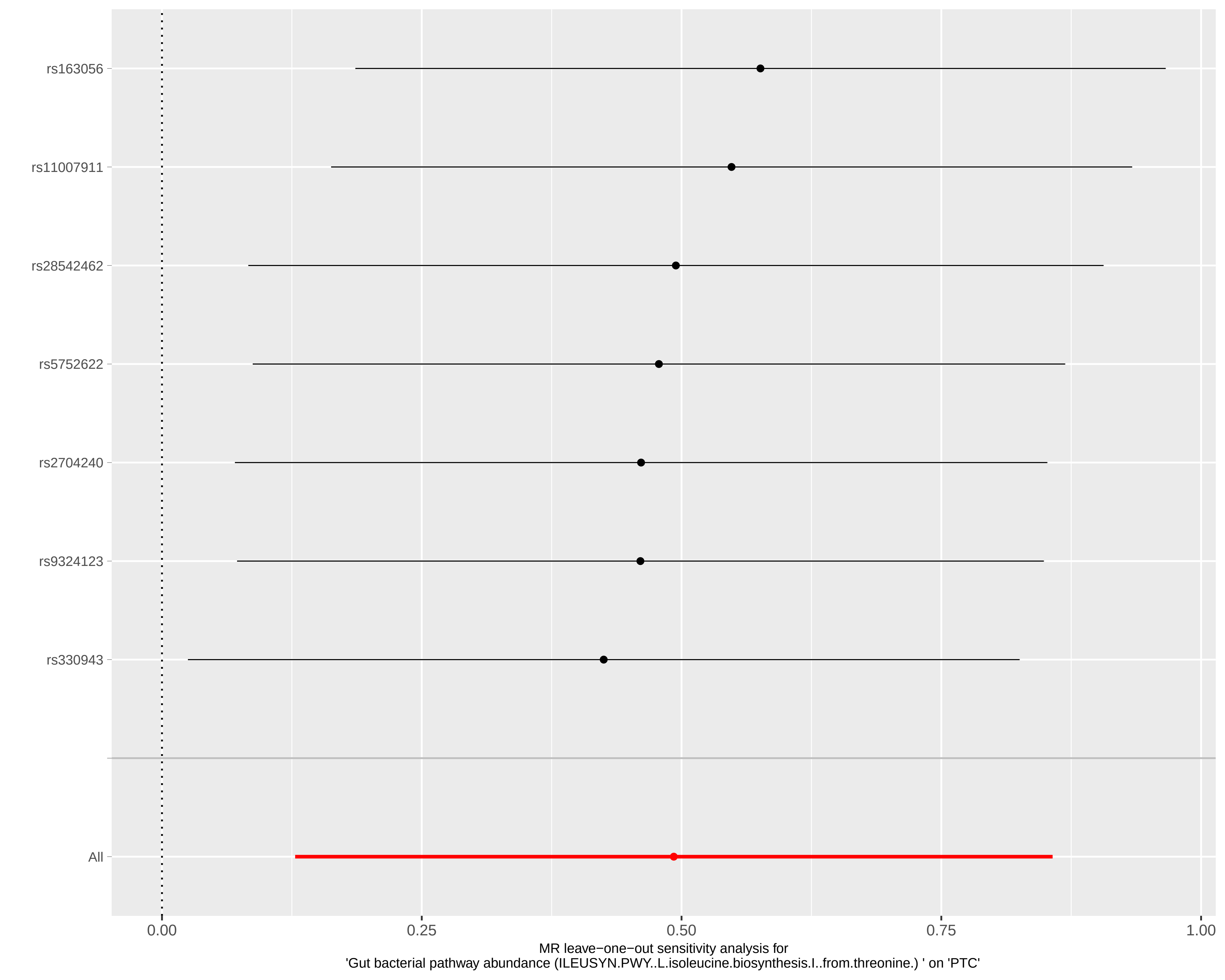

rs163056
rs11007911
rs28542462
rs5752622
rs2704240
rs9324123
rs330943
All
0.00
0.25
0.50
0.75
1.00
MR leave−one−out sensitivity analysis for
'Gut bacterial pathway abundance (ILEUSYN.PWY..L.isoleucine.biosynthesis.I..from.threonine.) ' on 'PTC'

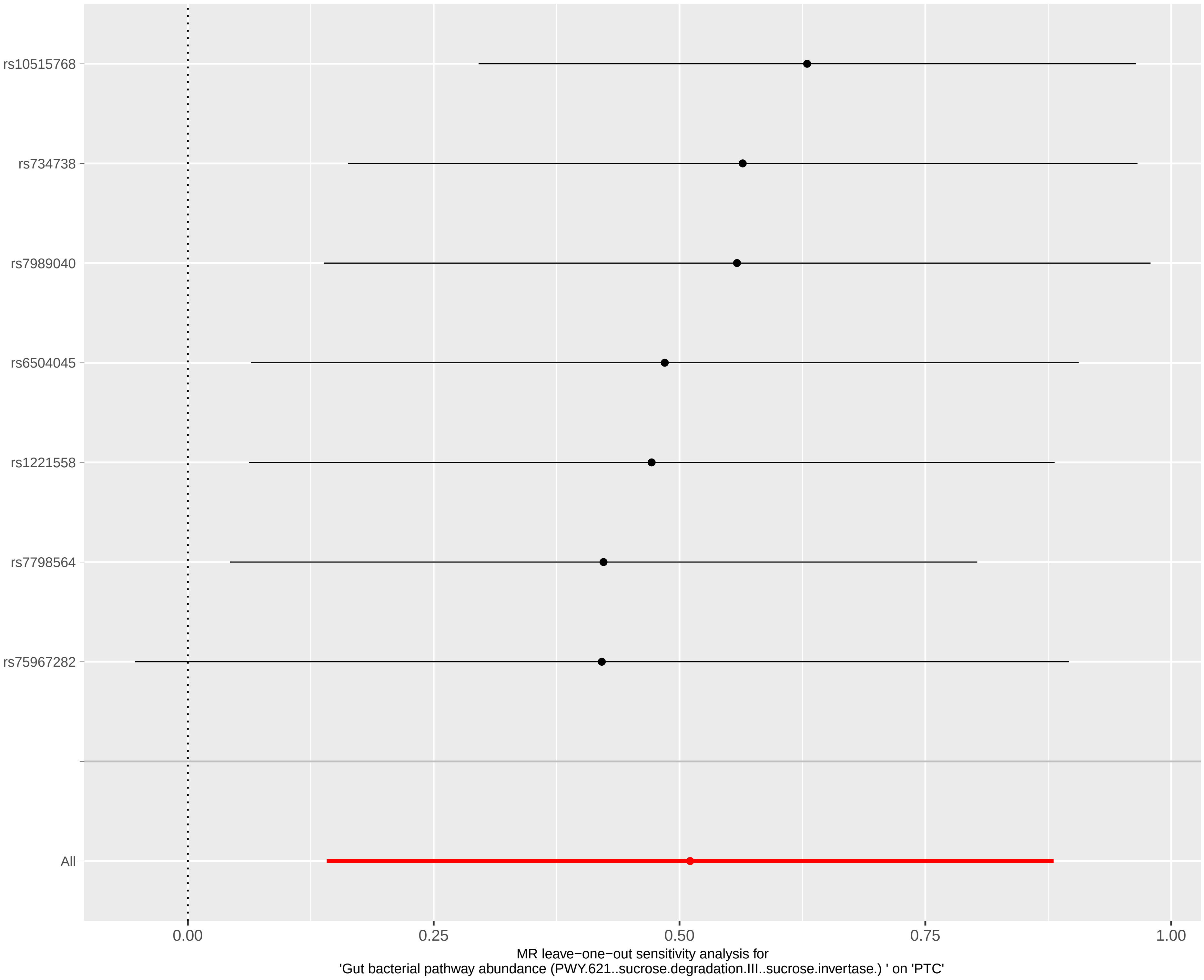
rs10515768
rs734738
rs7989040
rs6504045
rs1221558
rs7798564
rs75967282
All
0.00
0.25
0.50
0.75
1.00
MR leave−one−out sensitivity analysis for
'Gut bacterial pathway abundance (PWY.621..sucrose.degradation.III..sucrose.invertase.) ' on 'PTC'

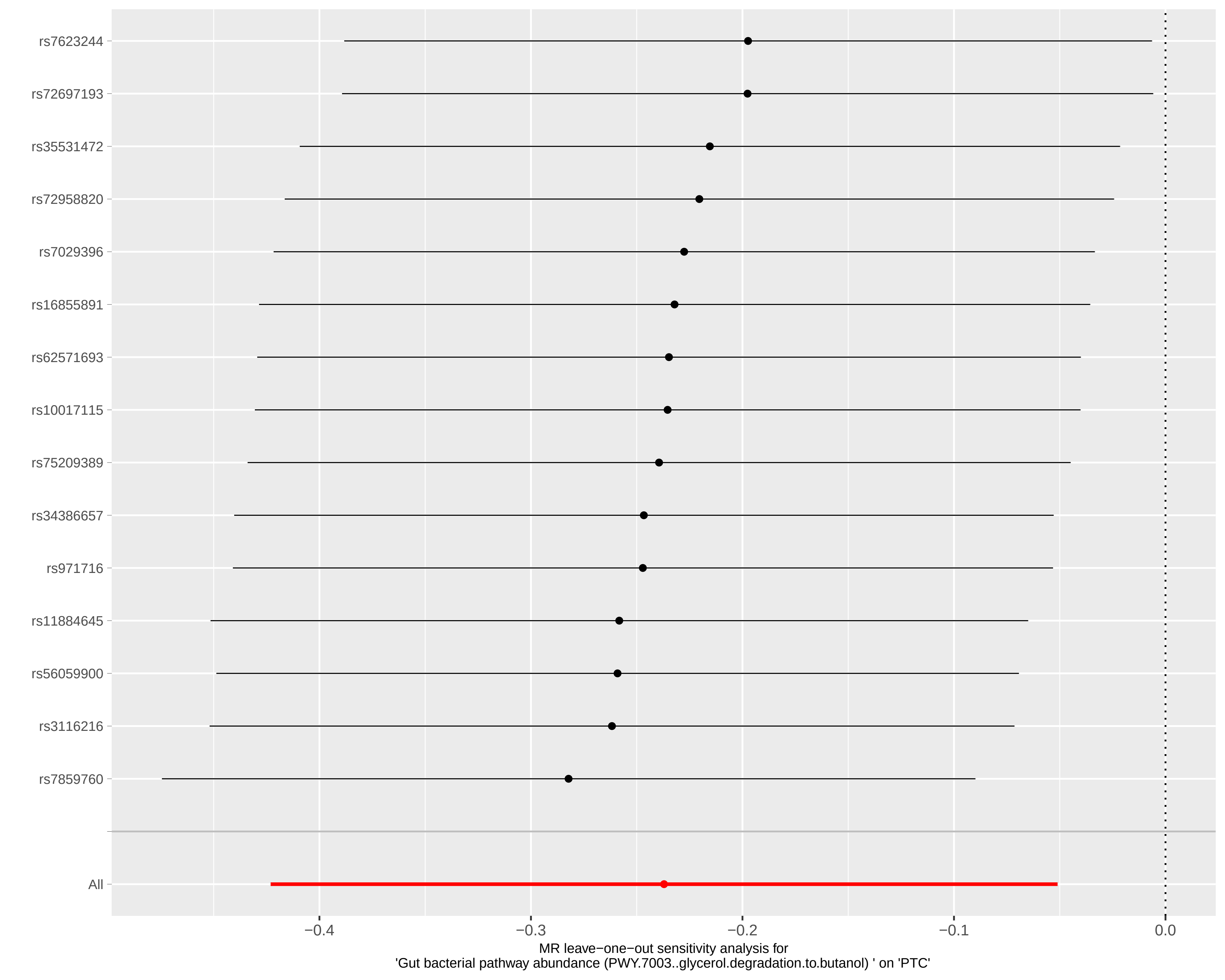
rs7623244
rs72697193
rs35531472
rs72958820
rs7029396
rs16855891
rs62571693
rs10017115
rs75209389
rs34386657
rs971716
rs11884645
rs56059900
rs3116216
rs7859760
All
−0.4
−0.3
−0.2
−0.1
0.0
MR leave−one−out sensitivity analysis for
'Gut bacterial pathway abundance (PWY.7003..glycerol.degradation.to.butanol) ' on 'PTC'

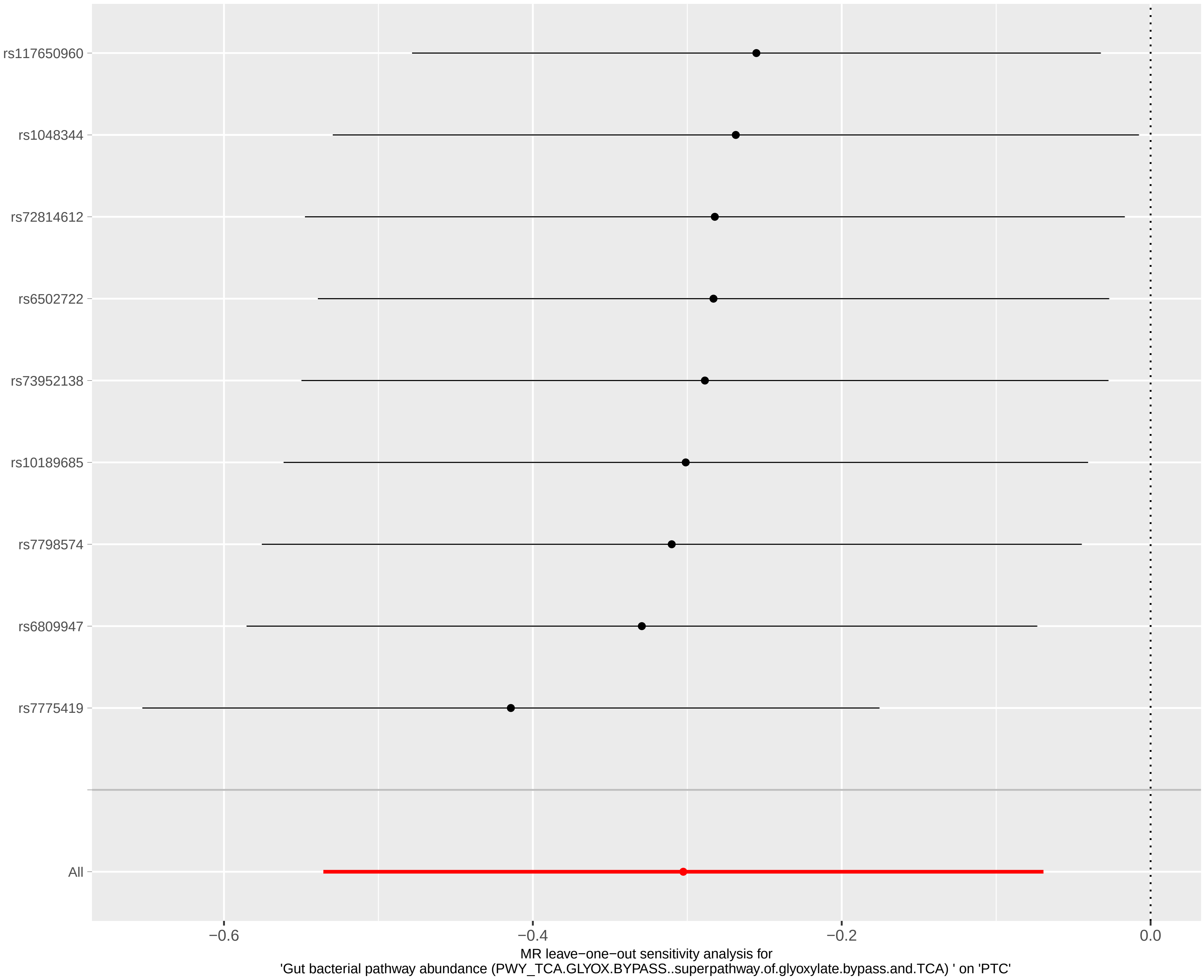
rs117650960
rs1048344
rs72814612
rs6502722
rs73952138
rs10189685
rs7798574
rs6809947
rs7775419
All
−0.6
−0.4
−0.2
0.0
MR leave−one−out sensitivity analysis for
'Gut bacterial pathway abundance (PWY_TCA.GLYOX.BYPASS..superpathway.of.glyoxylate.bypass.and.TCA) ' on 'PTC'

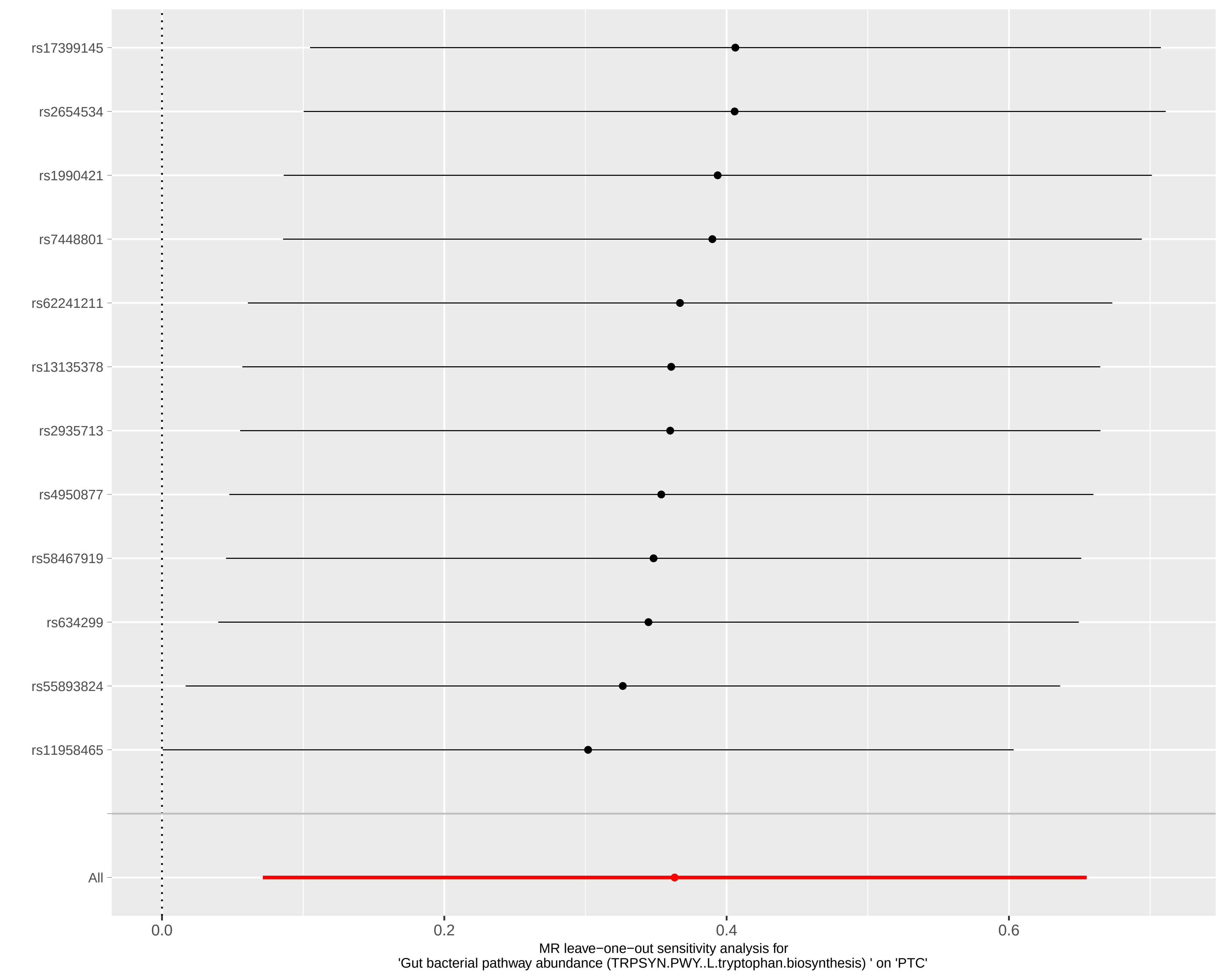

rs17399145
rs2654534
rs1990421
rs7448801
rs62241211
rs13135378
rs2935713
rs4950877
rs58467919
rs634299
rs55893824
rs11958465
All
0.0
0.2
0.4
0.6
MR leave−one−out sensitivity analysis for
'Gut bacterial pathway abundance (TRPSYN.PWY..L.tryptophan.biosynthesis) ' on 'PTC'

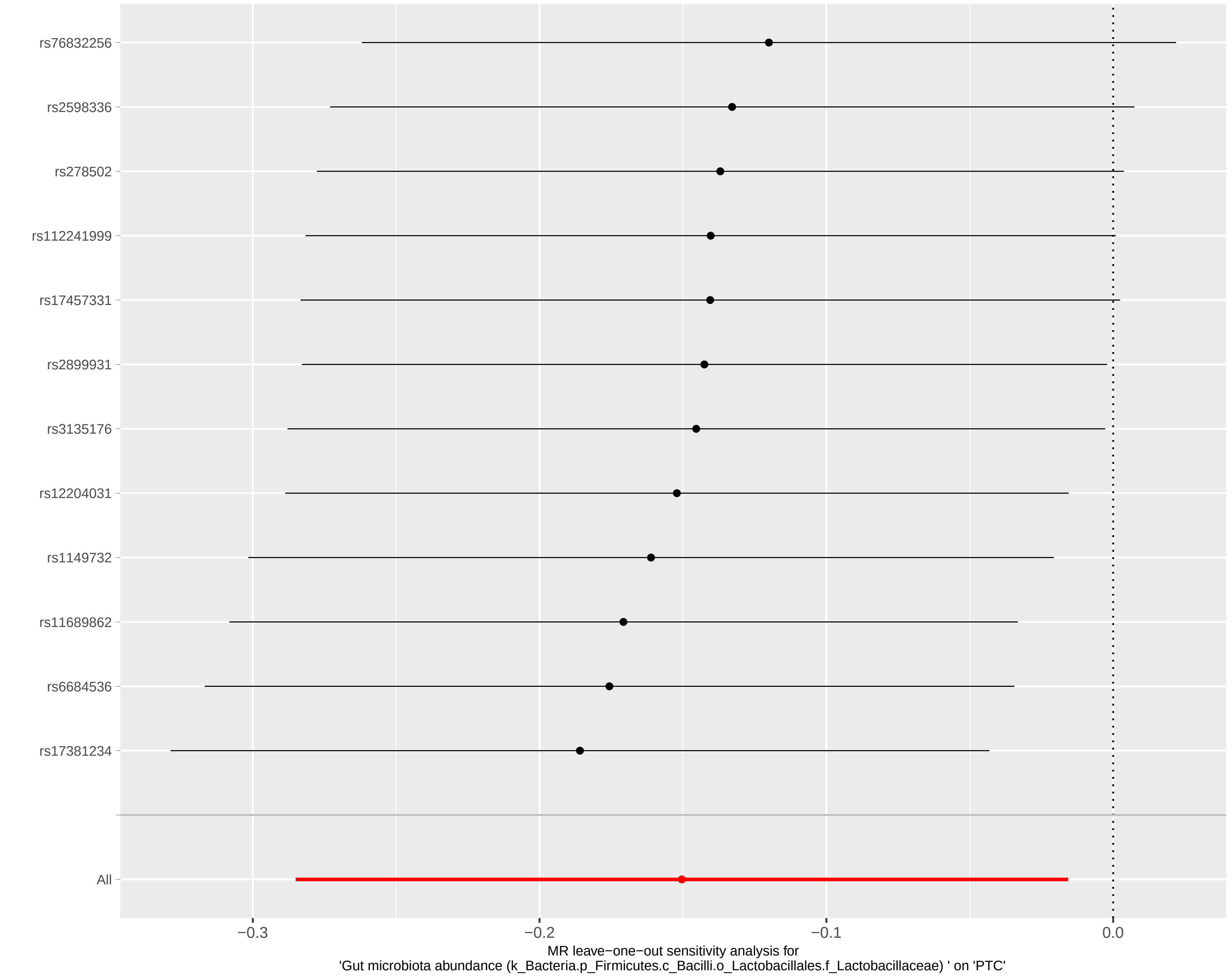
rs76832256
rs2598336
rs278502
rs112241999
rs17457331
rs2899931
rs3135176
rs12204031
rs1149732
rs11689862
rs6684536
rs17381234
All
−0.3
−0.2
−0.1
0.0
MR leave−one−out sensitivity analysis for
'Gut microbiota abundance (k_Bacteria.p_Firmicutes.c_Bacilli.o_Lactobacillales.f_Lactobacillaceae) ' on 'PTC'

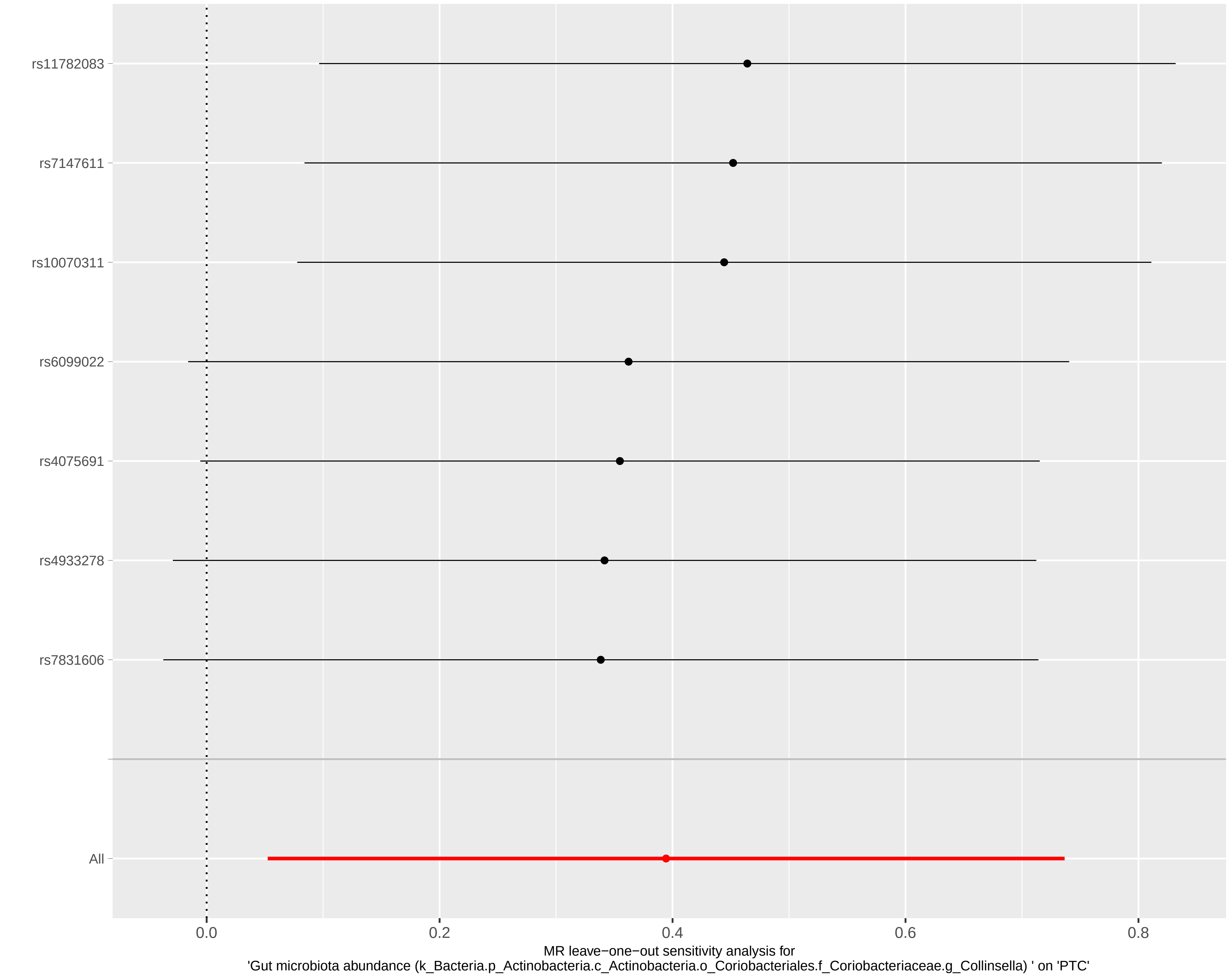
rs11782083
rs7147611
rs10070311
rs6099022
rs4075691
rs4933278
rs7831606
All
0.0
0.2
0.4
0.6
0.8
MR leave−one−out sensitivity analysis for
'Gut microbiota abundance (k_Bacteria.p_Actinobacteria.c_Actinobacteria.o_Coriobacteriales.f_Coriobacteriaceae.g_Collinsella) ' on 'PTC'

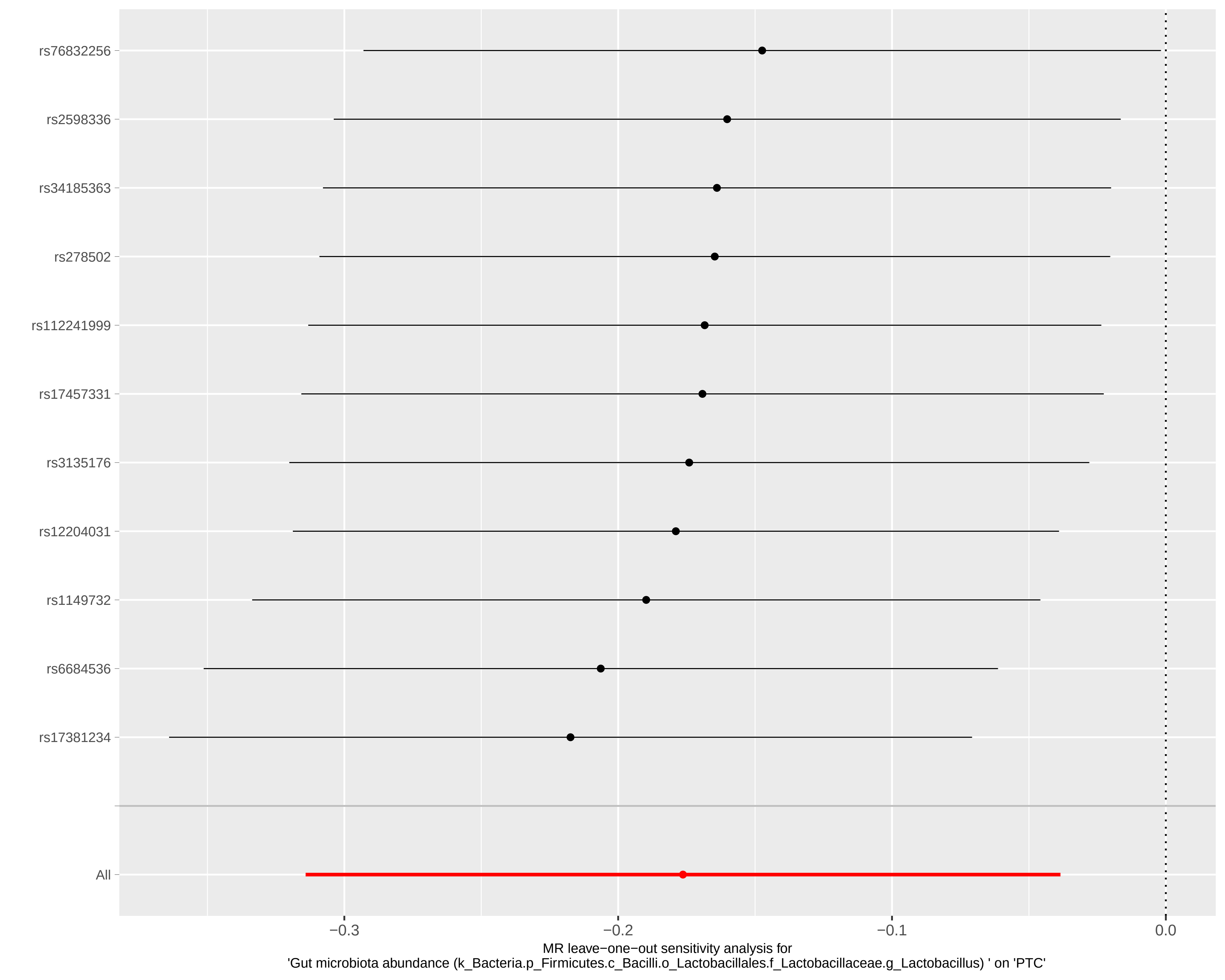
rs76832256
rs2598336
rs34185363
rs278502
rs112241999
rs17457331
rs3135176
rs12204031
rs1149732
rs6684536
rs17381234
All
−0.3
−0.2
−0.1
0.0
MR leave−one−out sensitivity analysis for
'Gut microbiota abundance (k_Bacteria.p_Firmicutes.c_Bacilli.o_Lactobacillales.f_Lactobacillaceae.g_Lactobacillus) ' on 'PTC'

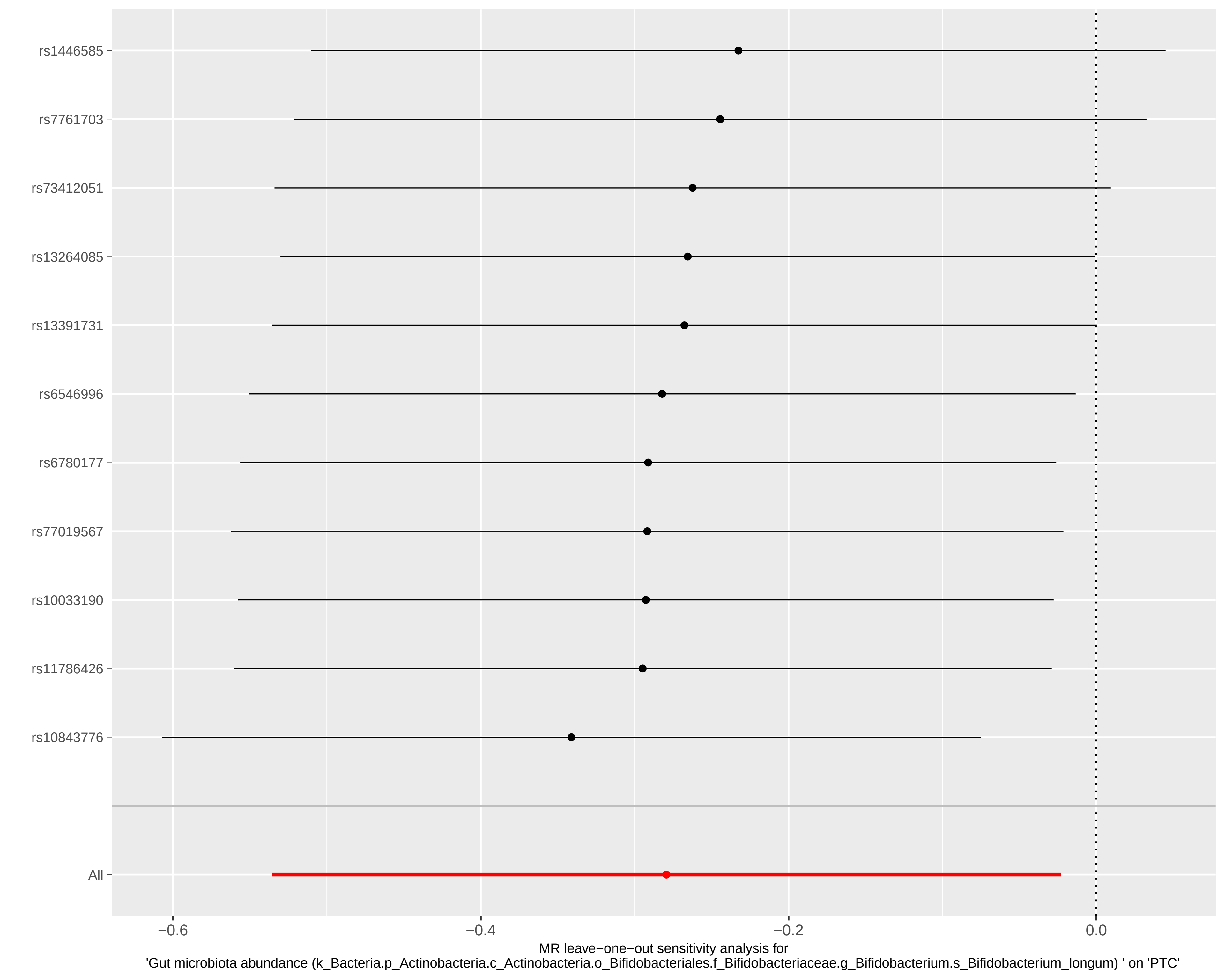
rs1446585
rs7761703
rs73412051
rs13264085
rs13391731
rs6546996
rs6780177
rs77019567
rs10033190
rs11786426
rs10843776
All
−0.6
−0.4
−0.2
0.0
MR leave−one−out sensitivity analysis for
'Gut microbiota abundance (k_Bacteria.p_Actinobacteria.c_Actinobacteria.o_Bifidobacteriales.f_Bifidobacteriaceae.g_Bifidobacterium.s_Bifidobacterium_longum) ' on 'PTC'
