# Supplementary material for: Blood metabolites reflect the effect of gut microbiota on differentiated thyroid cancer: a Mendelian randomization analysis
Source: BMC Cancer. 2025 Feb 28;25:368. doi: 10.1186/s12885-025-13598-y (PMC11869591; doi:10.1186/s12885-025-13598-y)
Supplement: Supplementary file 3 — Supplementary Material 3. [file 12885_2025_13598_MOESM3_ESM.zip › Figure S2 Leave-one-out analysis for MR causal effects of microbiota abundance and metabolism on FTC.pdf]

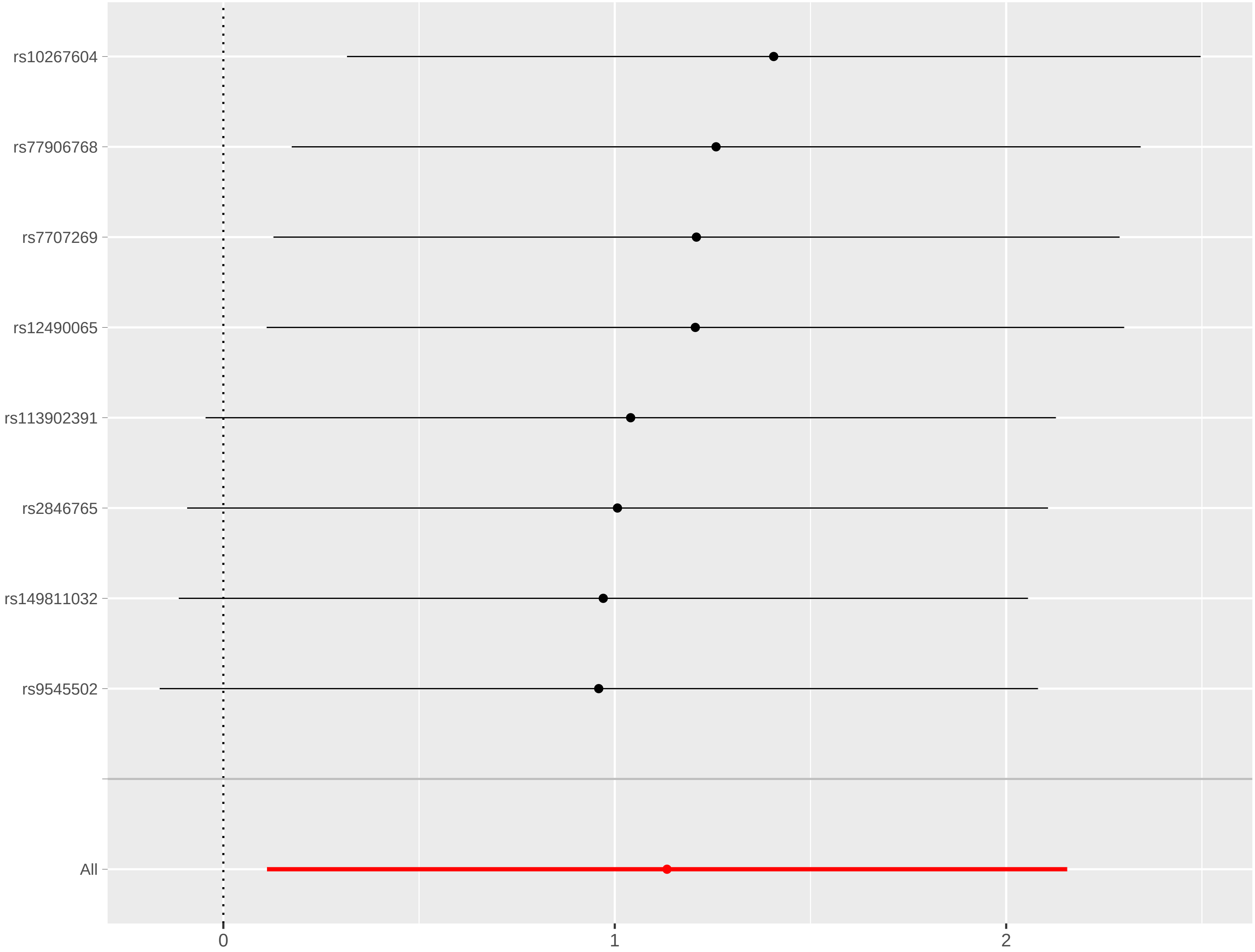

MR leave−one−out sensitivity analysis for
'Gut bacterial pathway abundance (ASPASN.PWY..superpathway.of.L.aspartate.and.L.asparagine.biosynthesis) ' on 'FTC'

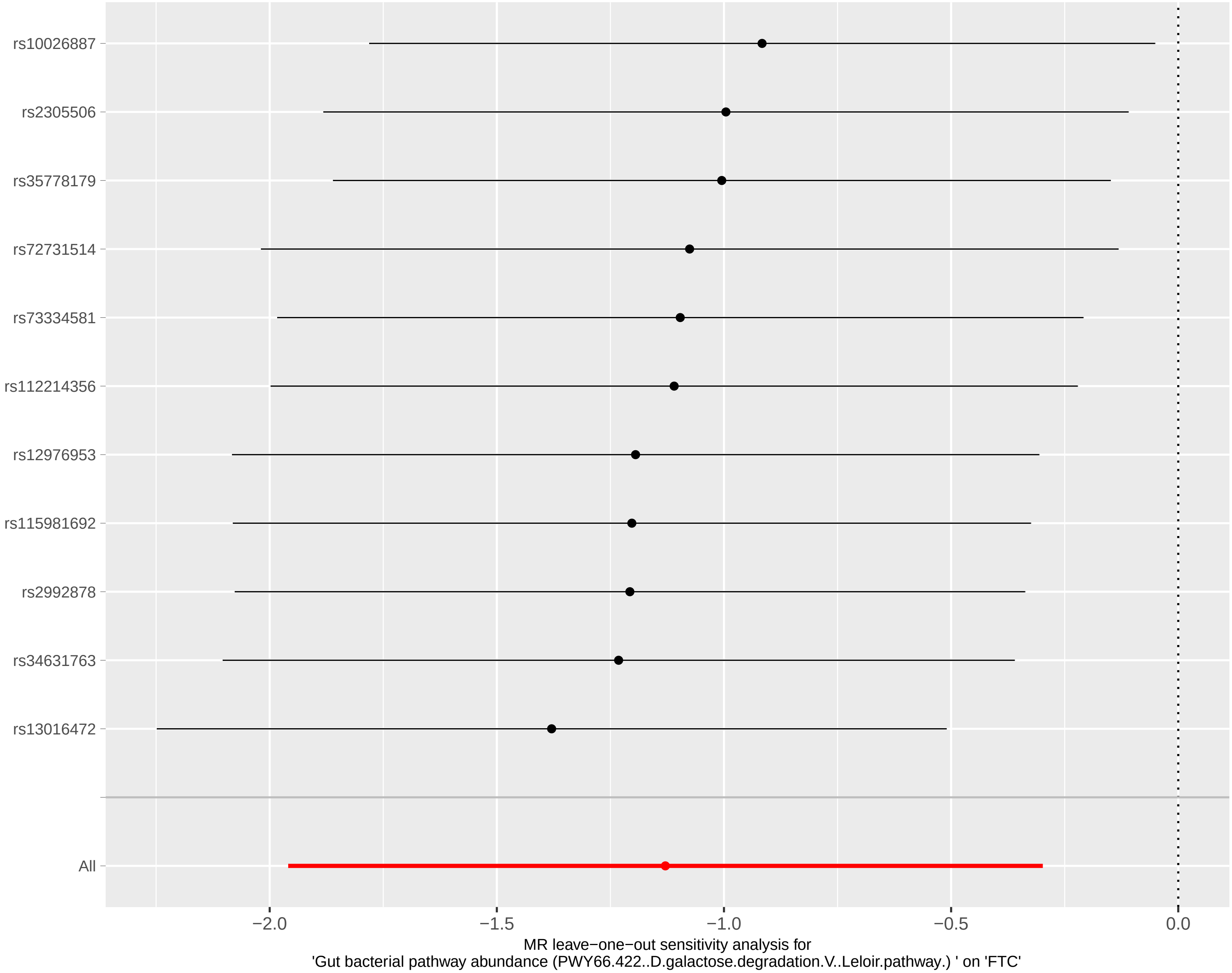
rs10026887
rs2305506
rs35778179
rs72731514
rs73334581
rs112214356
rs12976953
rs115981692
rs2992878
rs34631763
rs13016472
All
−2.0
−1.5
−1.0
−0.5
0.0
MR leave−one−out sensitivity analysis for
'Gut bacterial pathway abundance (PWY66.422..D.galactose.degradation.V..Leloir.pathway.) ' on 'FTC'

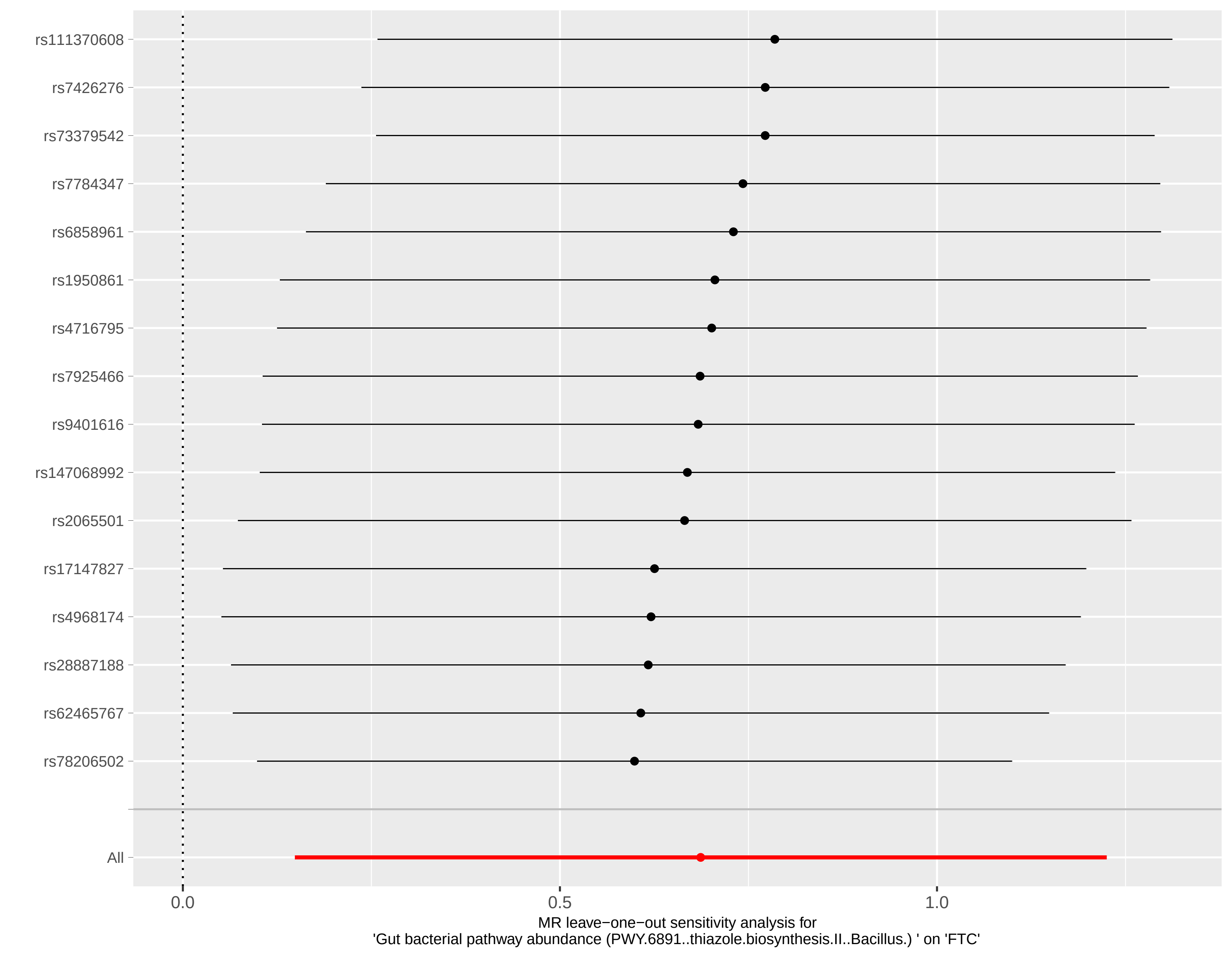
rs111370608
rs7426276
rs73379542
rs7784347
rs6858961
rs1950861
rs4716795
rs7925466
rs9401616
rs147068992
rs2065501
rs17147827
rs4968174
rs28887188
rs62465767
rs78206502
All
0.0
0.5
1.0
MR leave−one−out sensitivity analysis for
'Gut bacterial pathway abundance (PWY.6891..thiazole.biosynthesis.II..Bacillus.) ' on 'FTC'

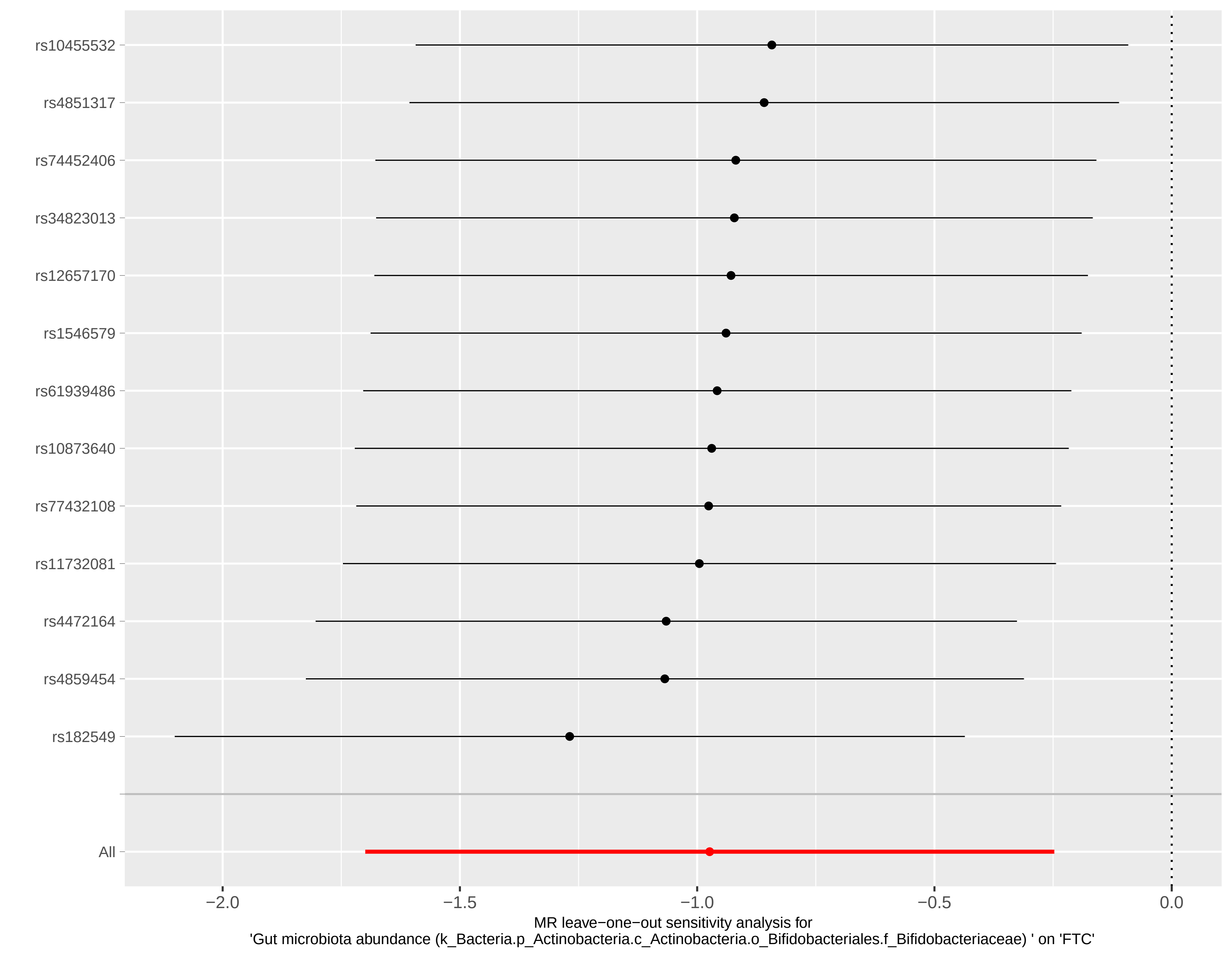

rs10455532
rs4851317
rs74452406
rs34823013
rs12657170
rs1546579
rs61939486
rs10873640
rs77432108
rs11732081
rs4472164
rs4859454
rs182549
All
−2.0
−1.5
−1.0
−0.5
0.0
MR leave−one−out sensitivity analysis for
'Gut microbiota abundance (k_Bacteria.p_Actinobacteria.c_Actinobacteria.o_Bifidobacteriales.f_Bifidobacteriaceae) ' on 'FTC'

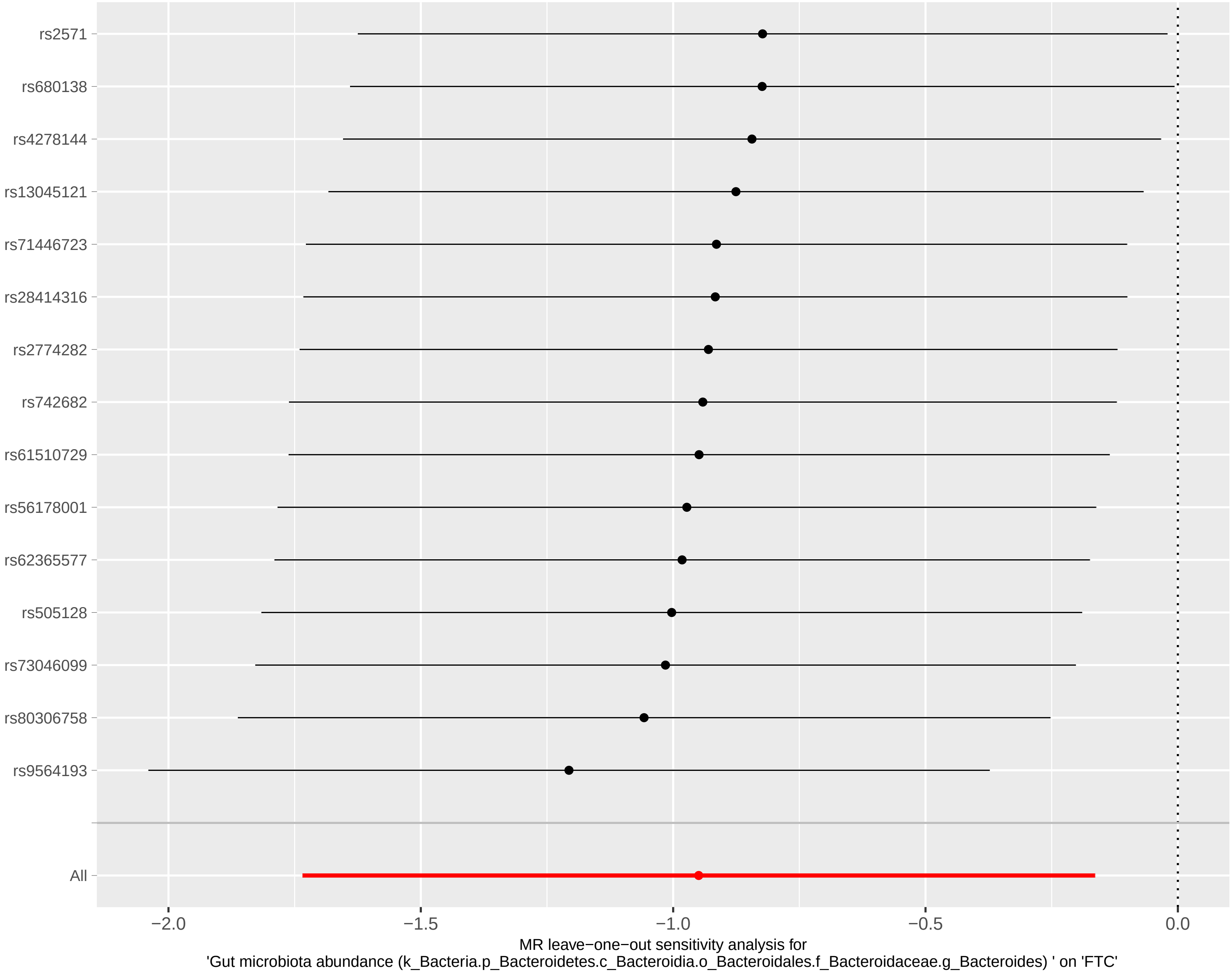
rs2571
rs680138
rs4278144
rs13045121
rs71446723
rs28414316
rs2774282
rs742682
rs61510729
rs56178001
rs62365577
rs505128
rs73046099
rs80306758
rs9564193
All
−2.0
−1.5
−1.0
−0.5
0.0
MR leave−one−out sensitivity analysis for
'Gut microbiota abundance (k_Bacteria.p_Bacteroidetes.c_Bacteroidia.o_Bacteroidales.f_Bacteroidaceae.g_Bacteroides) ' on 'FTC'

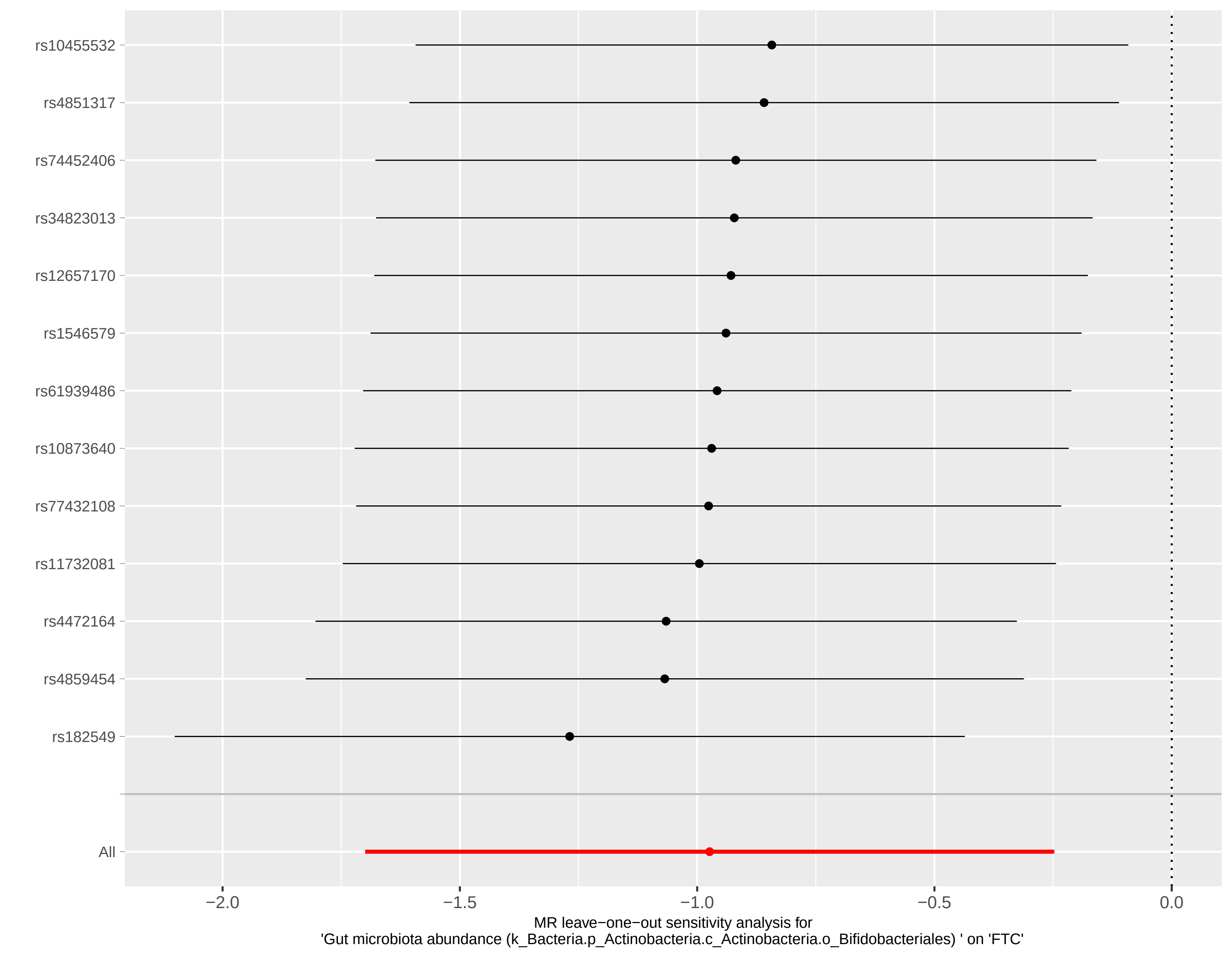
rs10455532
rs4851317
rs74452406
rs34823013
rs12657170
rs1546579
rs61939486
rs10873640
rs77432108
rs11732081
rs4472164
rs4859454
rs182549
All
−2.0
−1.5
−1.0
−0.5
0.0
MR leave−one−out sensitivity analysis for
'Gut microbiota abundance (k_Bacteria.p_Actinobacteria.c_Actinobacteria.o_Bifidobacteriales) ' on 'FTC'
