# Supplementary material for: Blood metabolites reflect the effect of gut microbiota on differentiated thyroid cancer: a Mendelian randomization analysis
Source: BMC Cancer. 2025 Feb 28;25:368. doi: 10.1186/s12885-025-13598-y (PMC11869591; doi:10.1186/s12885-025-13598-y)
Supplement: Supplementary file 3 — Supplementary Material 3. [file 12885_2025_13598_MOESM3_ESM.zip › Figure S3 Leave-one-out analysis for MR causal effects of blood metabolites on PTC.pdf]

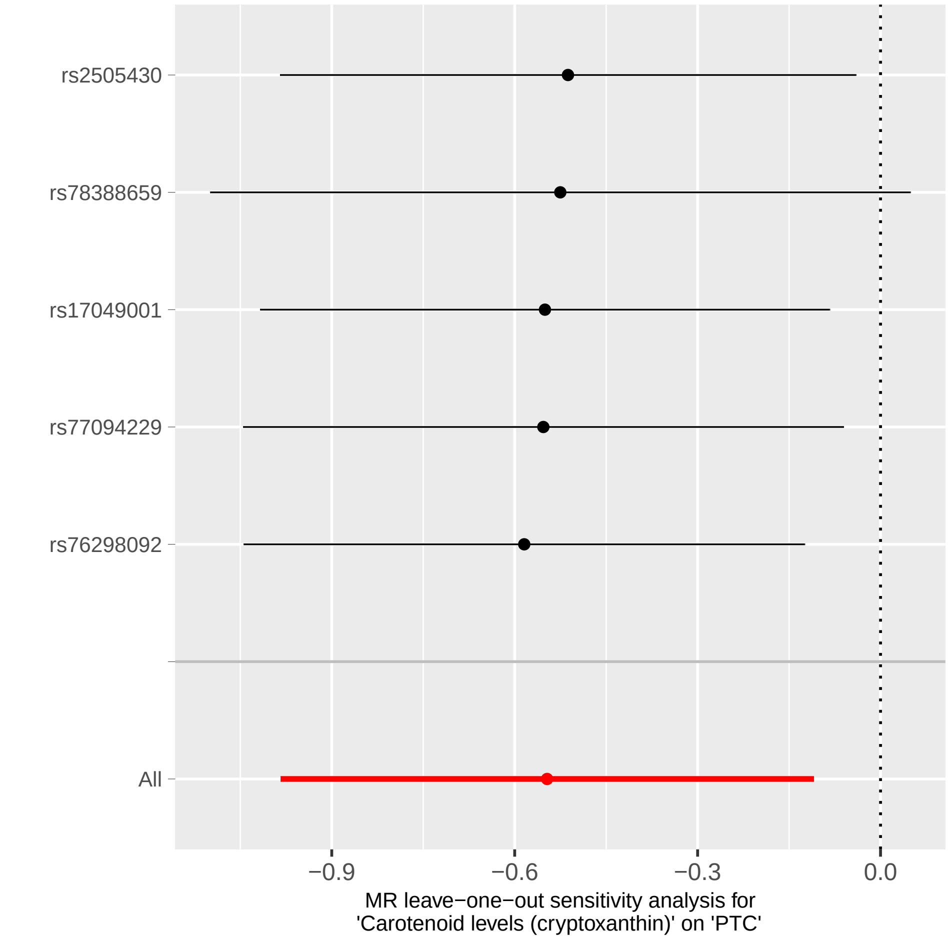

rs2505430
rs78388659
rs17049001
rs77094229
rs76298092
All
−0.9
−0.6
−0.3
0.0
MR leave−one−out sensitivity analysis for
'Carotenoid levels (cryptoxanthin)' on 'PTC'

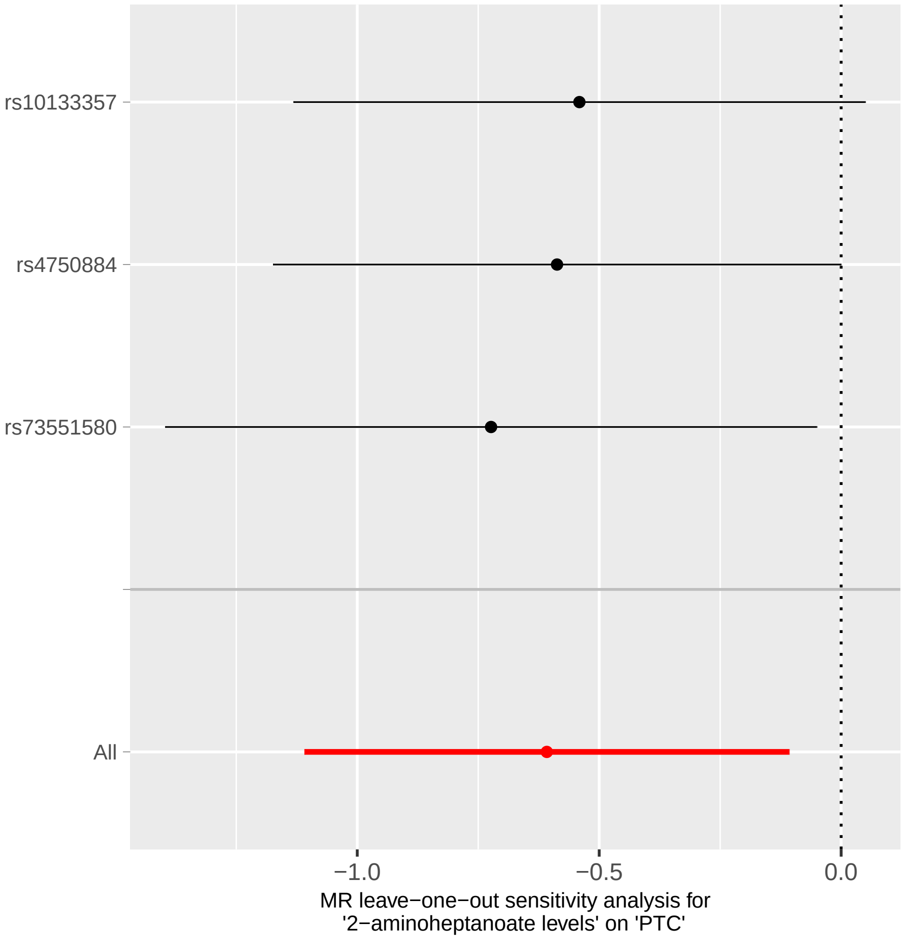
rs10133357
rs4750884
rs73551580
All
−1.0
−0.5
0.0
MR leave−one−out sensitivity analysis for
'2−aminoheptanoate levels' on 'PTC'

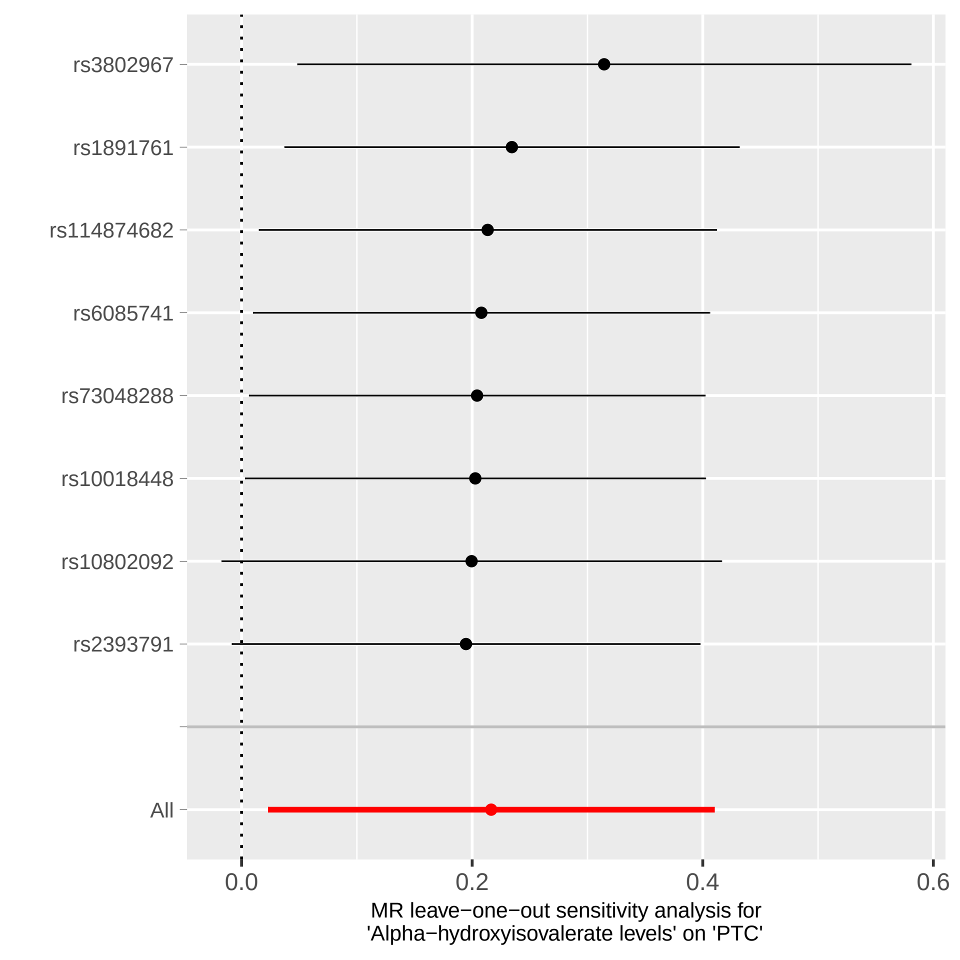

rs3802967
rs1891761
rs114874682
rs6085741
rs73048288
rs10018448
rs10802092
rs2393791
All
0.0
0.2
0.4
0.6
MR leave−one−out sensitivity analysis for
'Alpha−hydroxyisovalerate levels' on 'PTC'

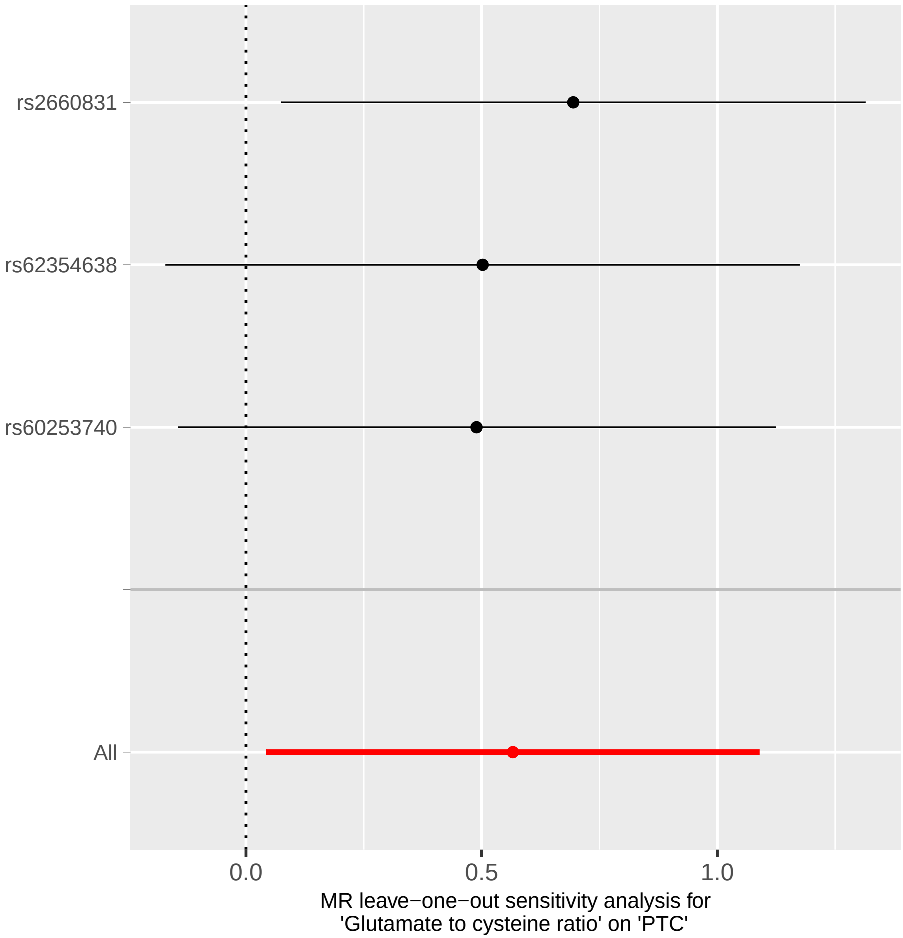

rs2660831
rs62354638
rs60253740
All
0.0
0.5
1.0
MR leave−one−out sensitivity analysis for
'Glutamate to cysteine ratio' on 'PTC'

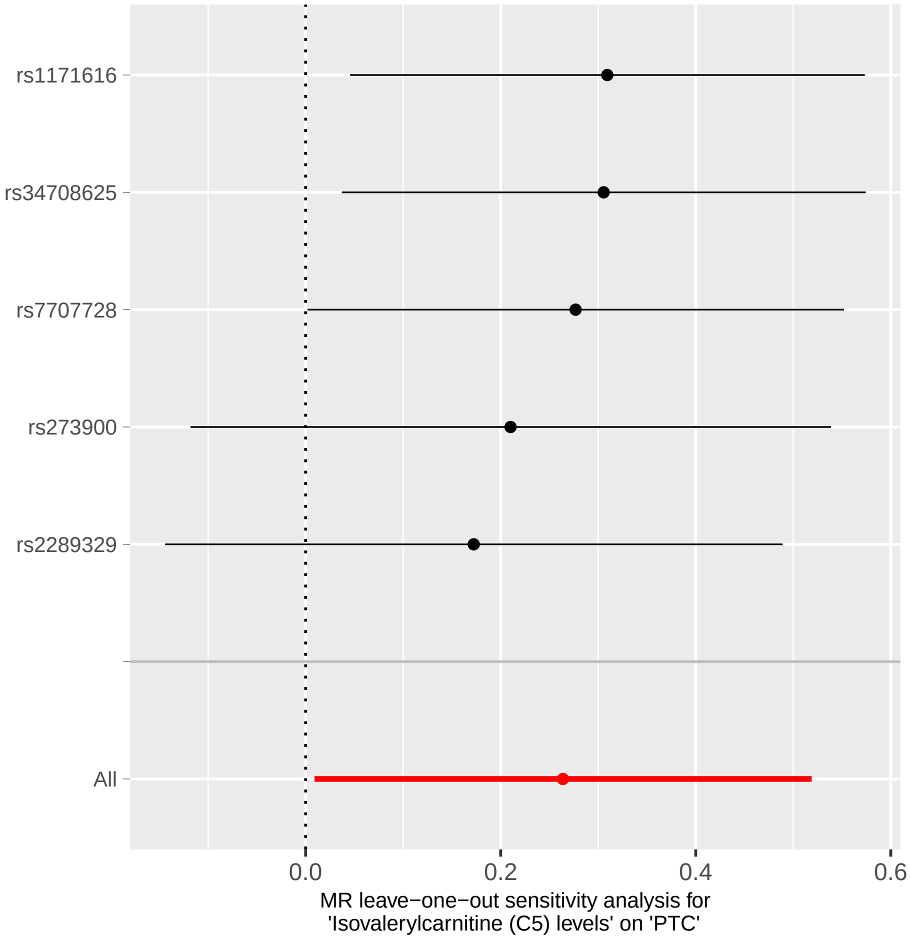
rs1171616
rs34708625
rs7707728
rs273900
rs2289329
All
0.0
0.2
0.4
0.6
MR leave−one−out sensitivity analysis for
'Isovalerylcarnitine (C5) levels' on 'PTC'

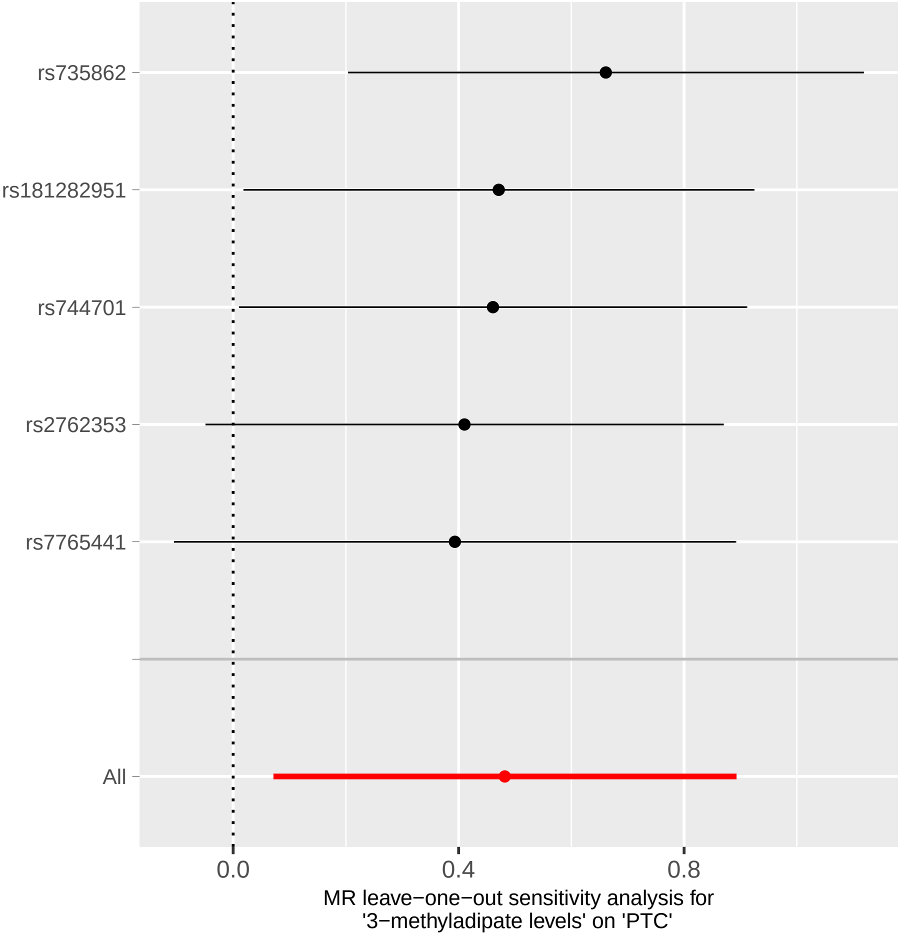

rs735862
rs181282951
rs744701
rs2762353
rs7765441
All
0.0
0.4
0.8
MR leave−one−out sensitivity analysis for
'3−methyladipate levels' on 'PTC'

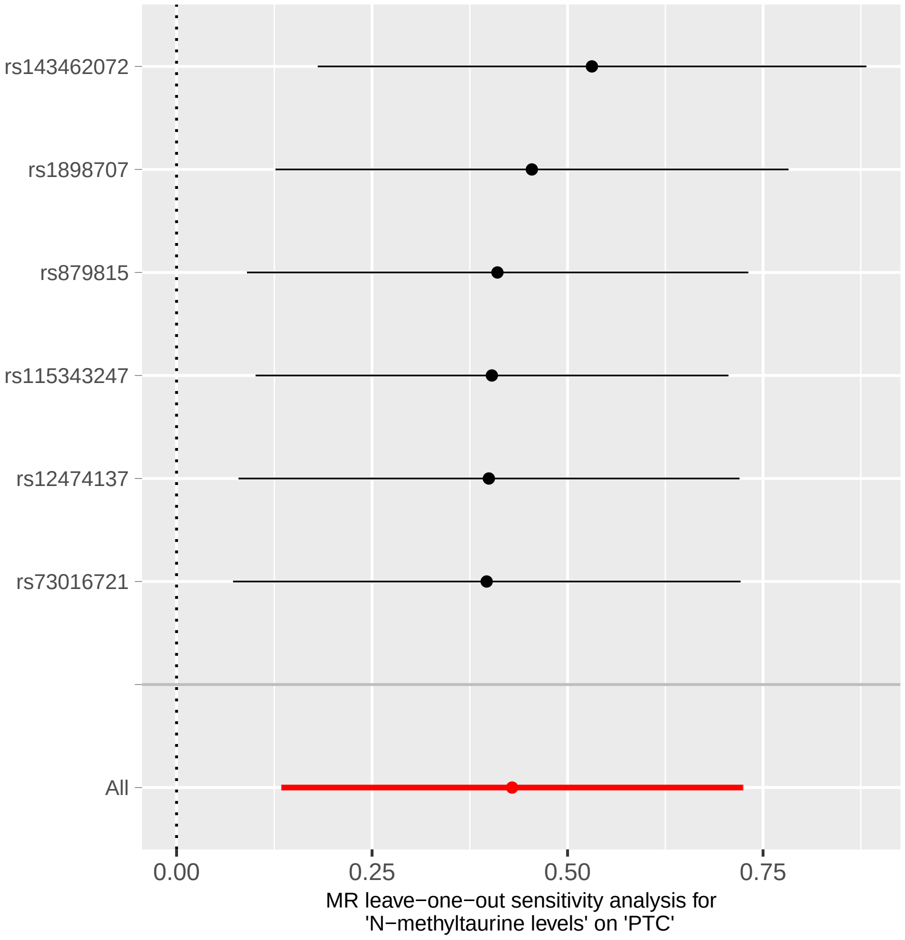

rs143462072
rs1898707
rs879815
rs115343247
rs12474137
rs73016721
All
0.00
0.25
0.50
0.75
MR leave−one−out sensitivity analysis for
'N−methyltaurine levels' on 'PTC'

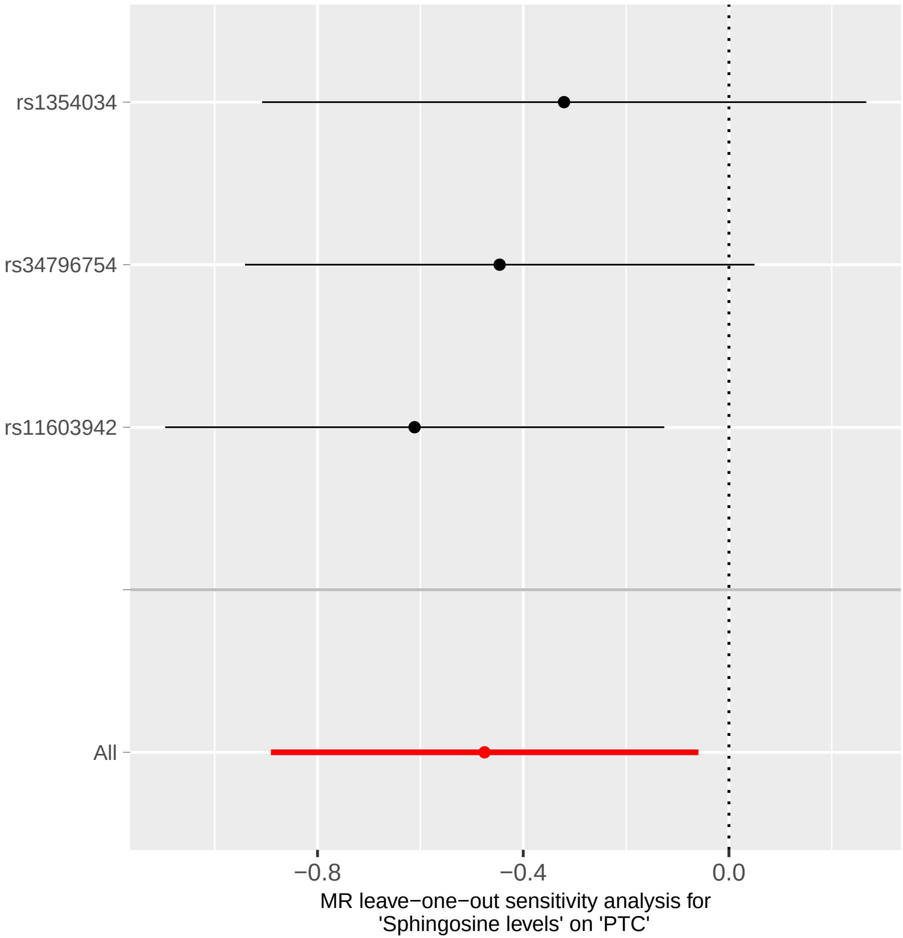

rs1354034
rs34796754
rs11603942
All
−0.8
−0.4
0.0
MR leave−one−out sensitivity analysis for
'Sphingosine levels' on 'PTC'

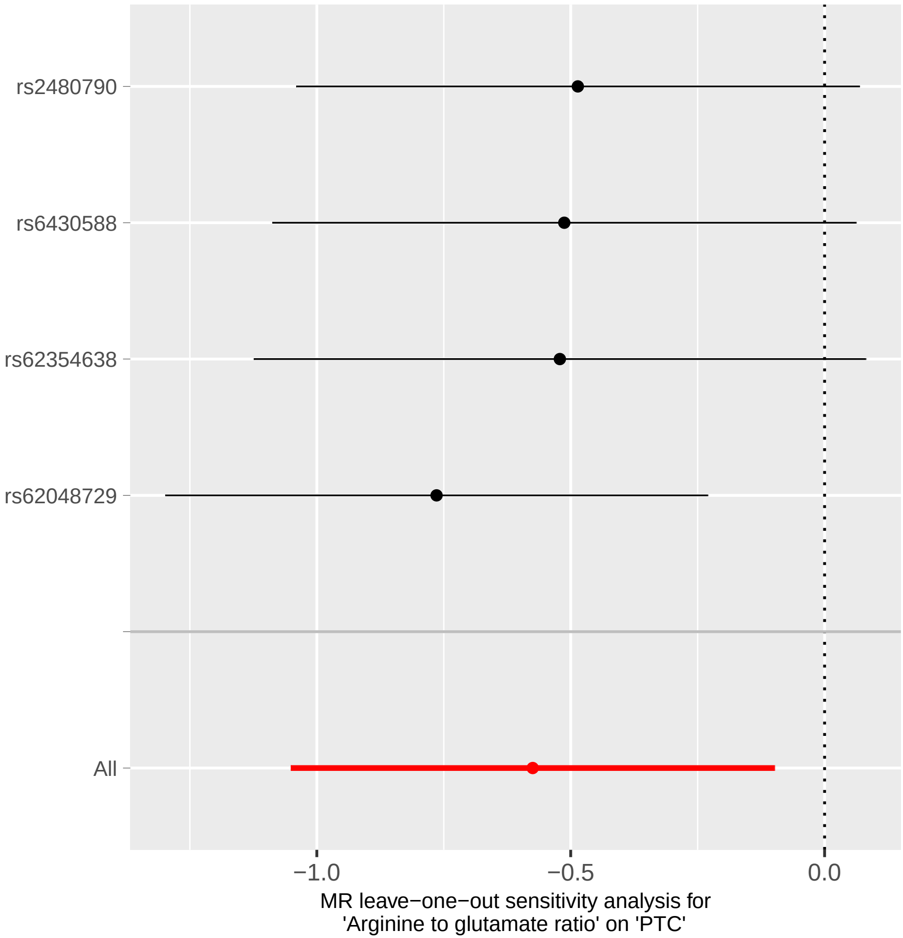
rs2480790
rs6430588
rs62354638
rs62048729
All
−1.0
−0.5
0.0
MR leave−one−out sensitivity analysis for
'Arginine to glutamate ratio' on 'PTC'

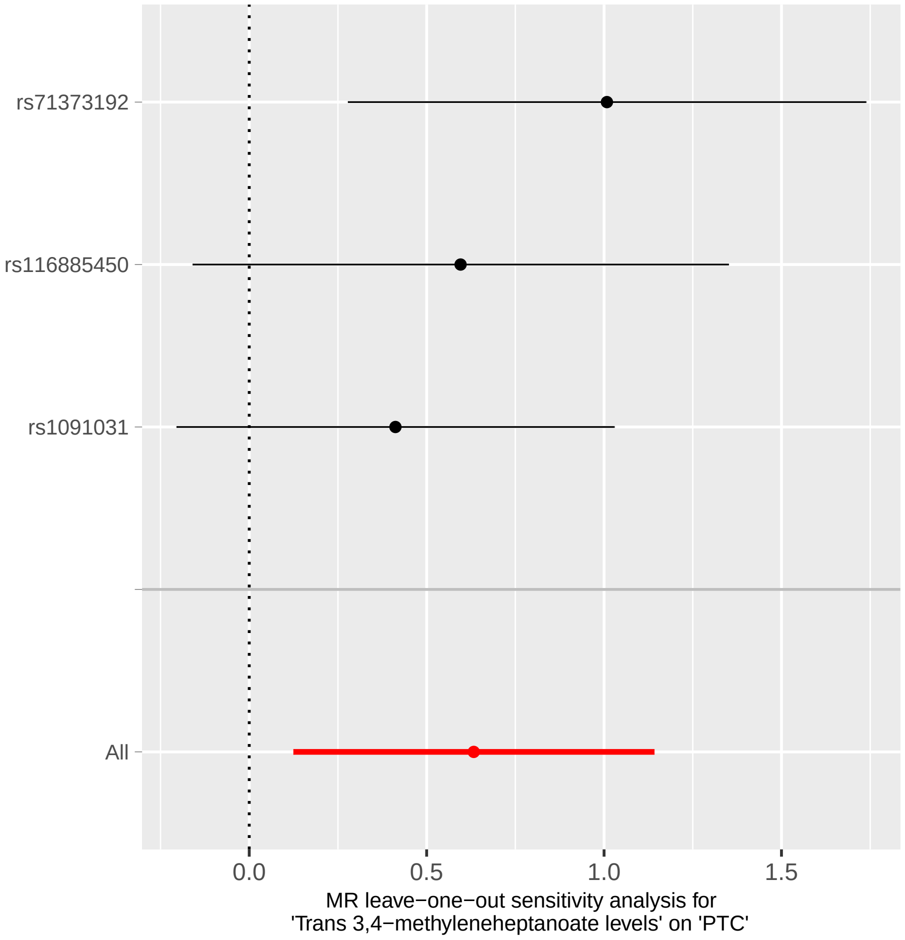
rs71373192
rs116885450
rs1091031
All
0.0
0.5
1.0
1.5
MR leave−one−out sensitivity analysis for
'Trans 3,4−methyleneheptanoate levels' on 'PTC'

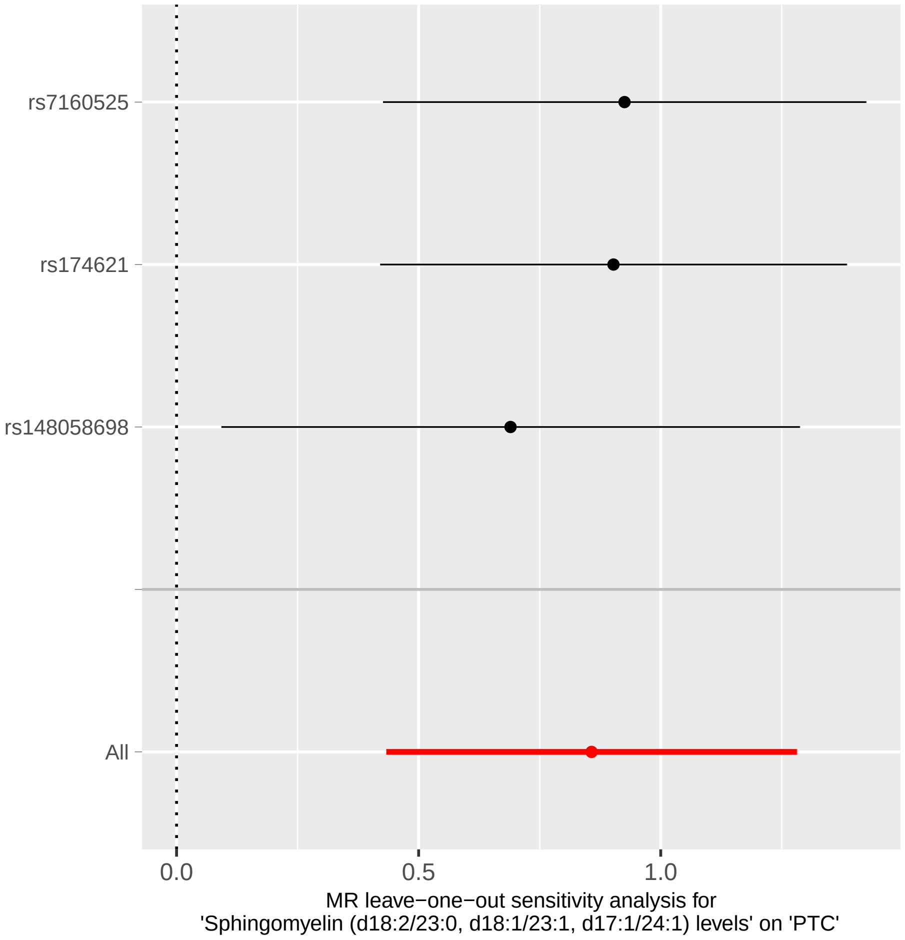

rs7160525
rs174621
rs148058698
All
0.0
0.5
1.0
MR leave−one−out sensitivity analysis for
'Sphingomyelin (d18:2/23:0, d18:1/23:1, d17:1/24:1) levels' on 'PTC'

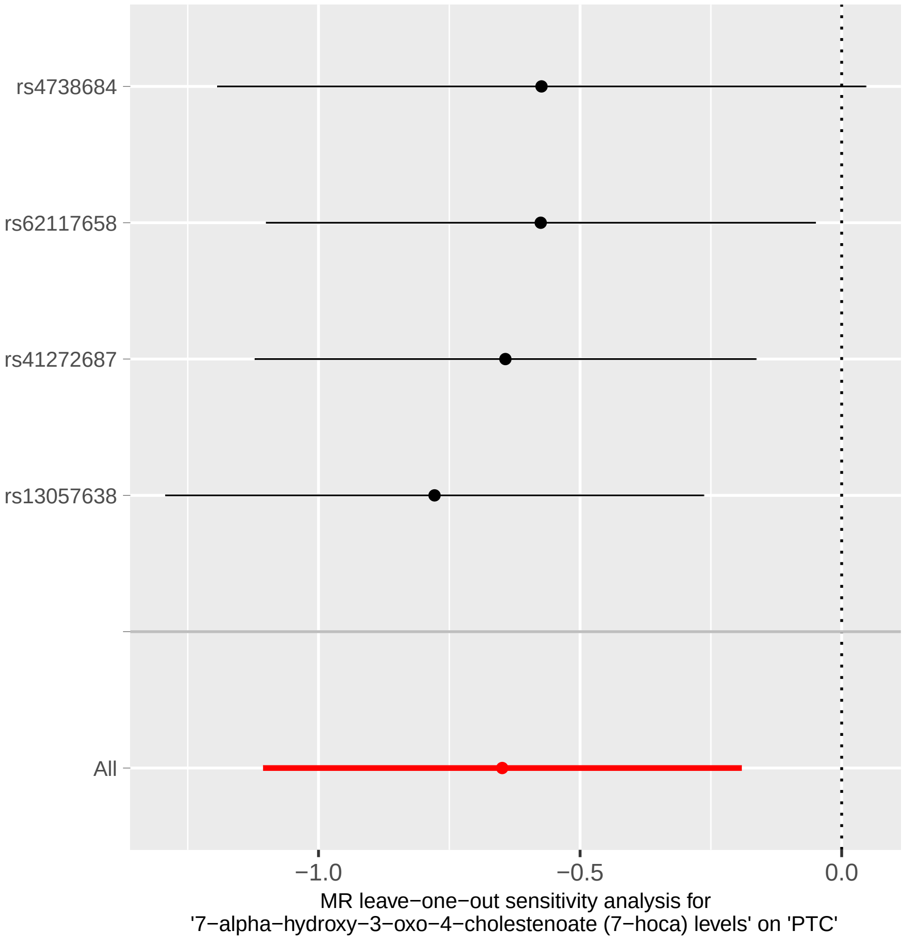
rs4738684
rs62117658
rs41272687
rs13057638
All
−1.0
−0.5
0.0
MR leave−one−out sensitivity analysis for
'7−alpha−hydroxy−3−oxo−4−cholestenoate (7−hoca) levels' on 'PTC'

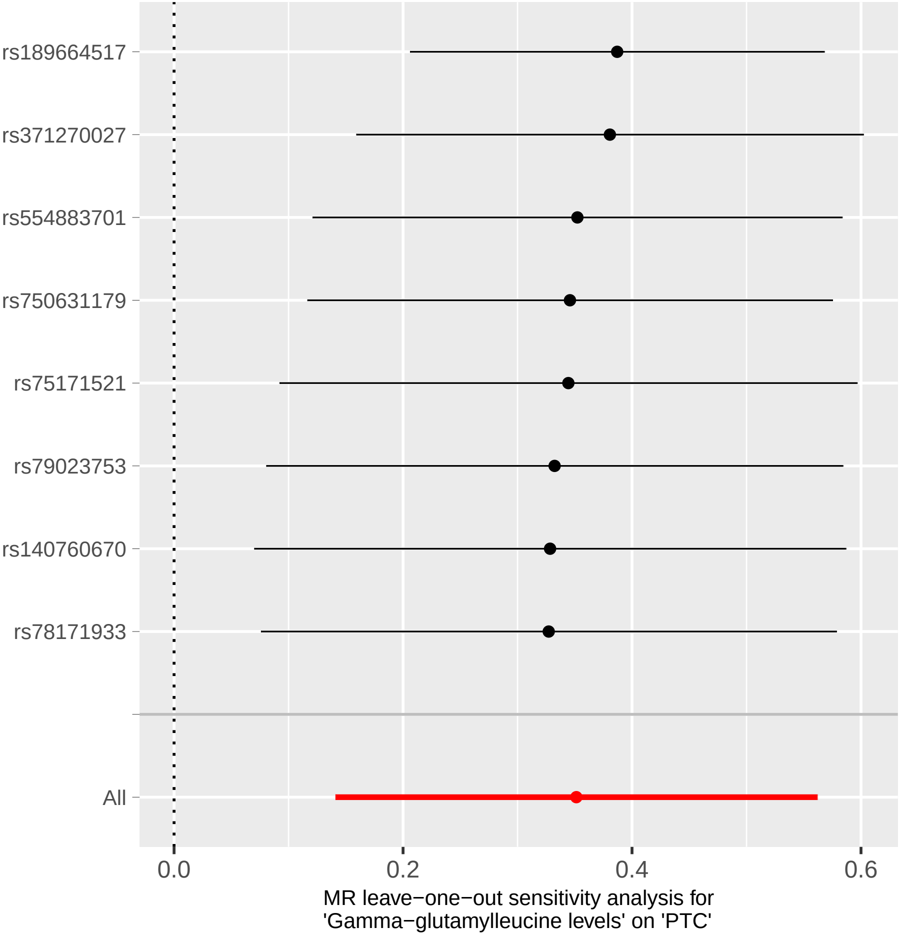
rs189664517
rs371270027
rs554883701
rs750631179
rs75171521
rs79023753
rs140760670
rs78171933
All
0.0
0.2
0.4
0.6
MR leave−one−out sensitivity analysis for
'Gamma−glutamylleucine levels' on 'PTC'

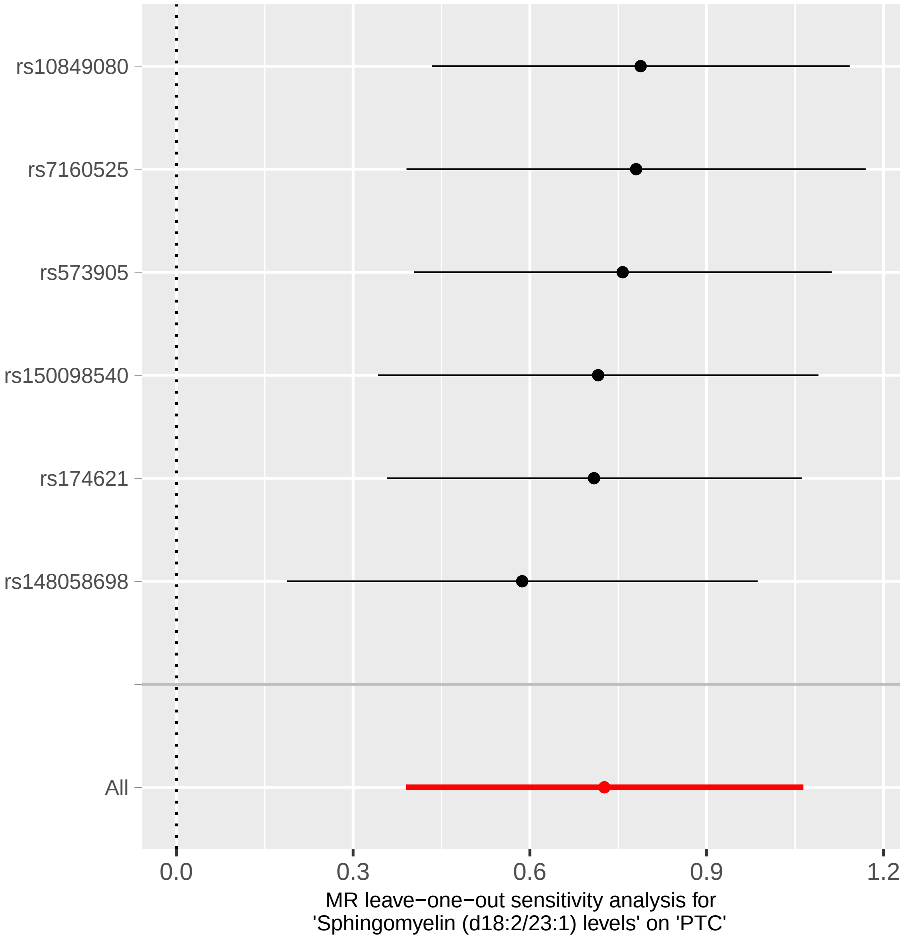

rs10849080
rs7160525
rs573905
rs150098540
rs174621
rs148058698
All
0.0
0.3
0.6
0.9
1.2
MR leave−one−out sensitivity analysis for
'Sphingomyelin (d18:2/23:1) levels' on 'PTC'

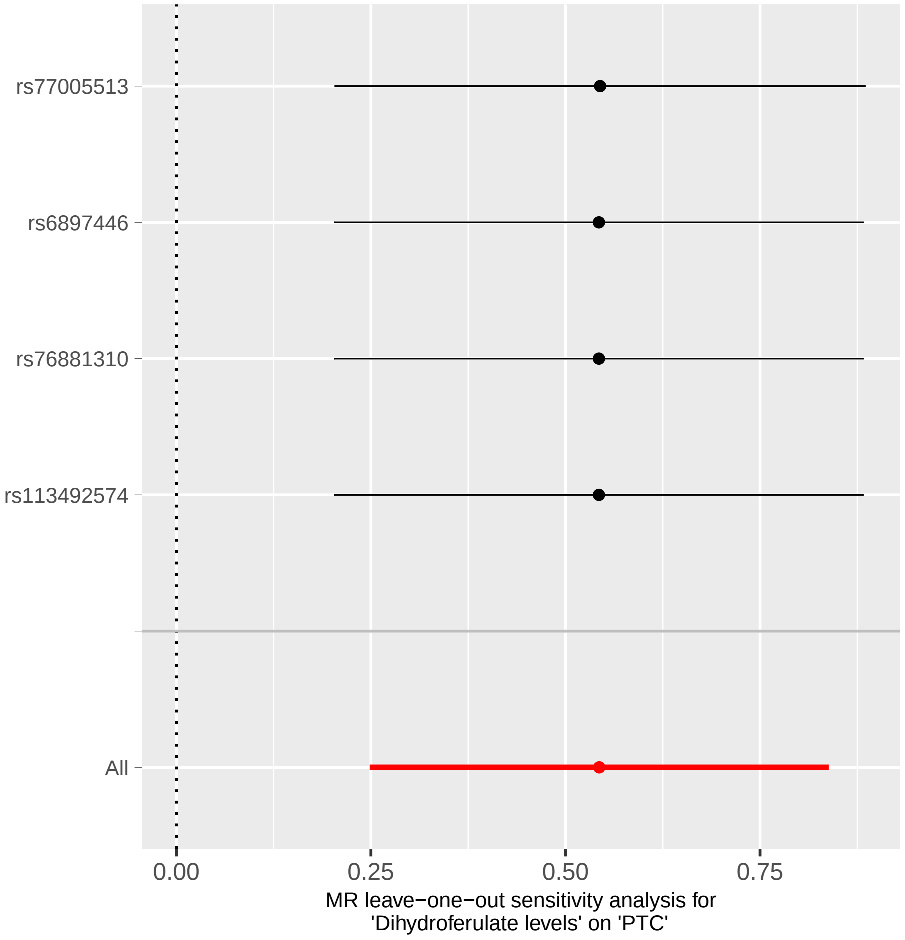
rs77005513
rs6897446
rs76881310
rs113492574
All
0.00
0.25
0.50
0.75
MR leave−one−out sensitivity analysis for
'Dihydroferulate levels' on 'PTC'

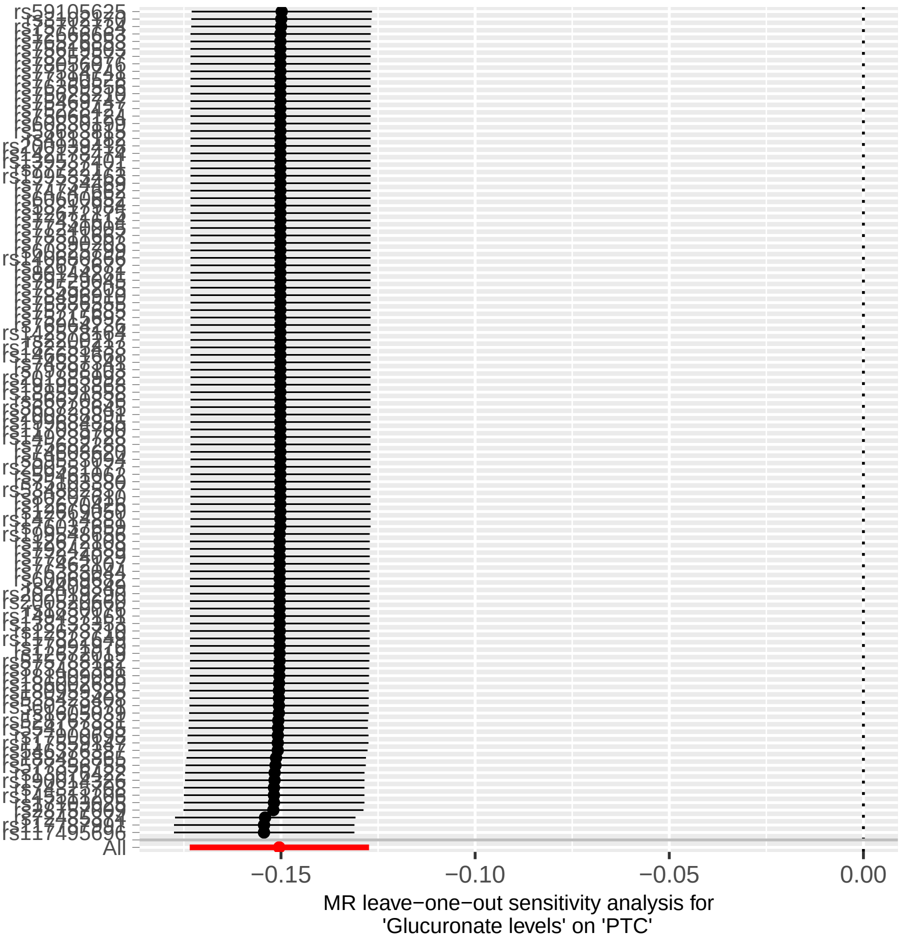
rs59105625
rs117495096
All
−0.15
−0.10
−0.05
0.00
MR leave−one−out sensitivity analysis for
'Glucuronate levels' on 'PTC'

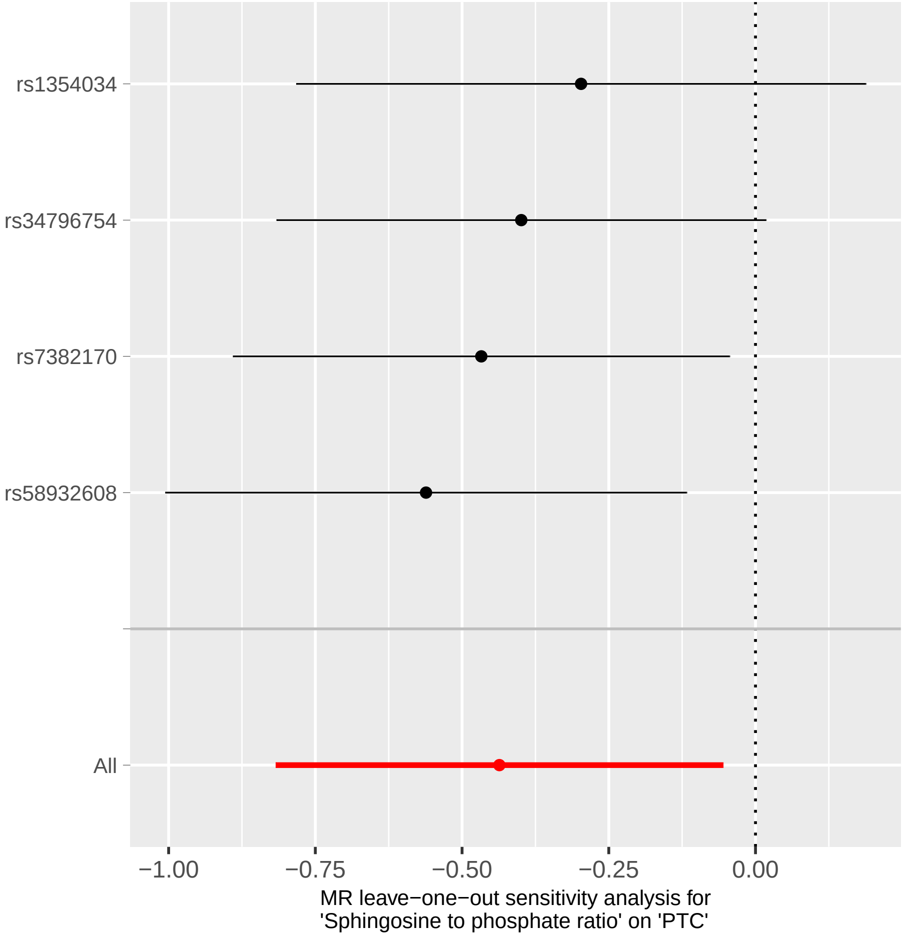
rs1354034
rs34796754
rs7382170
rs58932608
All
−1.00
−0.75
−0.50
−0.25
0.00
MR leave−one−out sensitivity analysis for
'Sphingosine to phosphate ratio' on 'PTC'

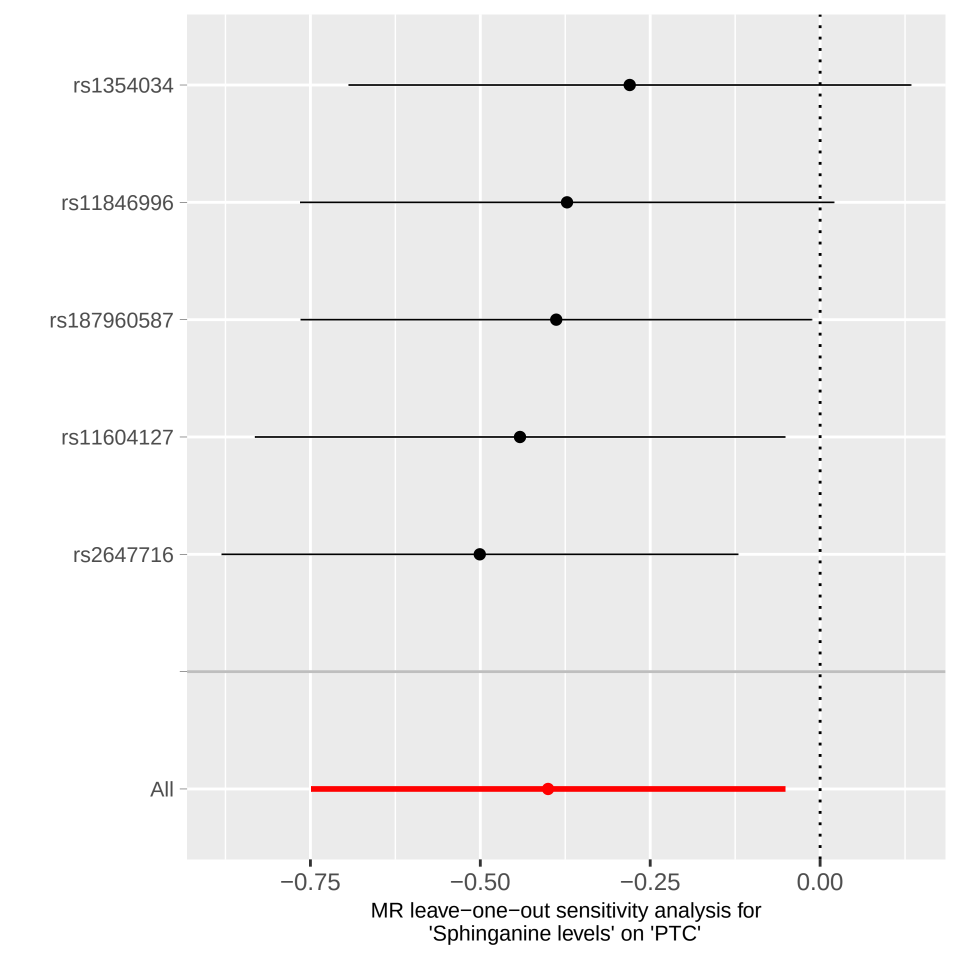

rs1354034
rs11846996
rs187960587
rs11604127
rs2647716
All
−0.75
−0.50
−0.25
0.00
MR leave−one−out sensitivity analysis for
'Sphinganine levels' on 'PTC'

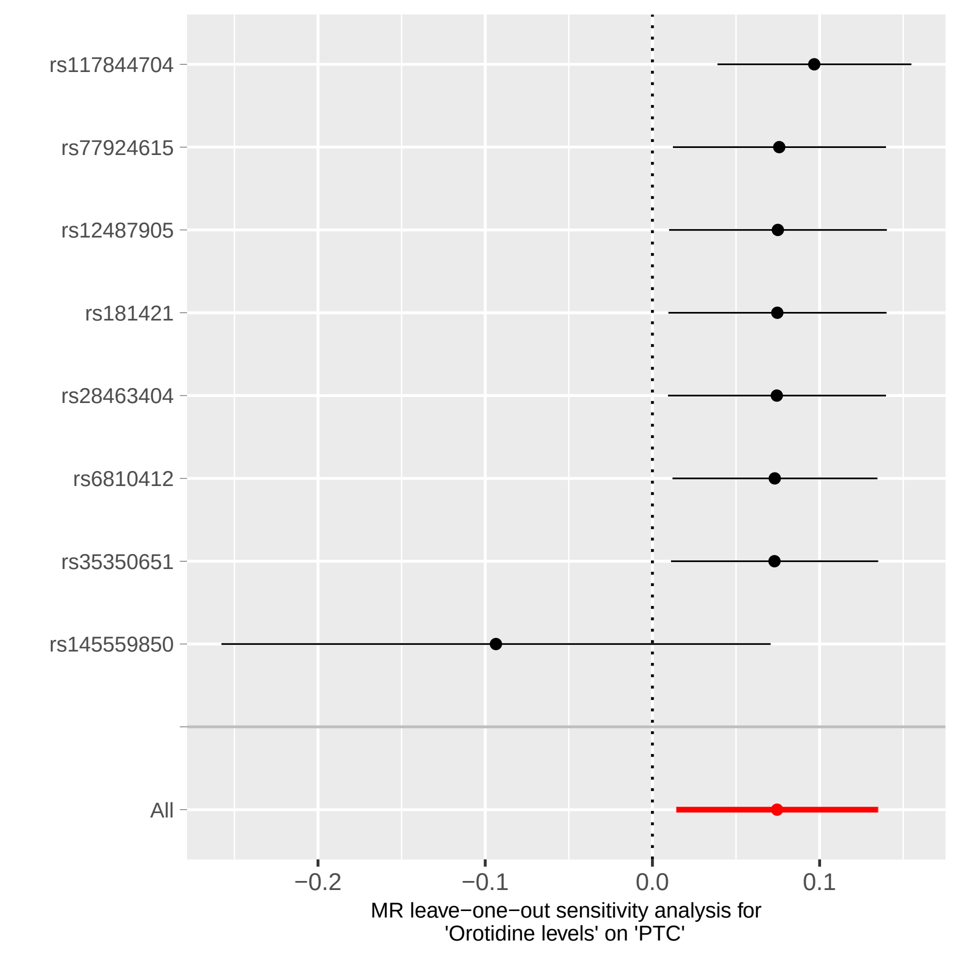

rs117844704
rs77924615
rs12487905
rs181421
rs28463404
rs6810412
rs35350651
rs145559850
All
−0.2
−0.1
0.0
0.1
MR leave−one−out sensitivity analysis for
'Orotidine levels' on 'PTC'
