# Supplementary material for: Blood metabolites reflect the effect of gut microbiota on differentiated thyroid cancer: a Mendelian randomization analysis
Source: BMC Cancer. 2025 Feb 28;25:368. doi: 10.1186/s12885-025-13598-y (PMC11869591; doi:10.1186/s12885-025-13598-y)
Supplement: Supplementary file 3 — Supplementary Material 3. [file 12885_2025_13598_MOESM3_ESM.zip › Figure S4 Leave-one-out analysis for MR causal effects of blood metabolites on FTC.pdf]

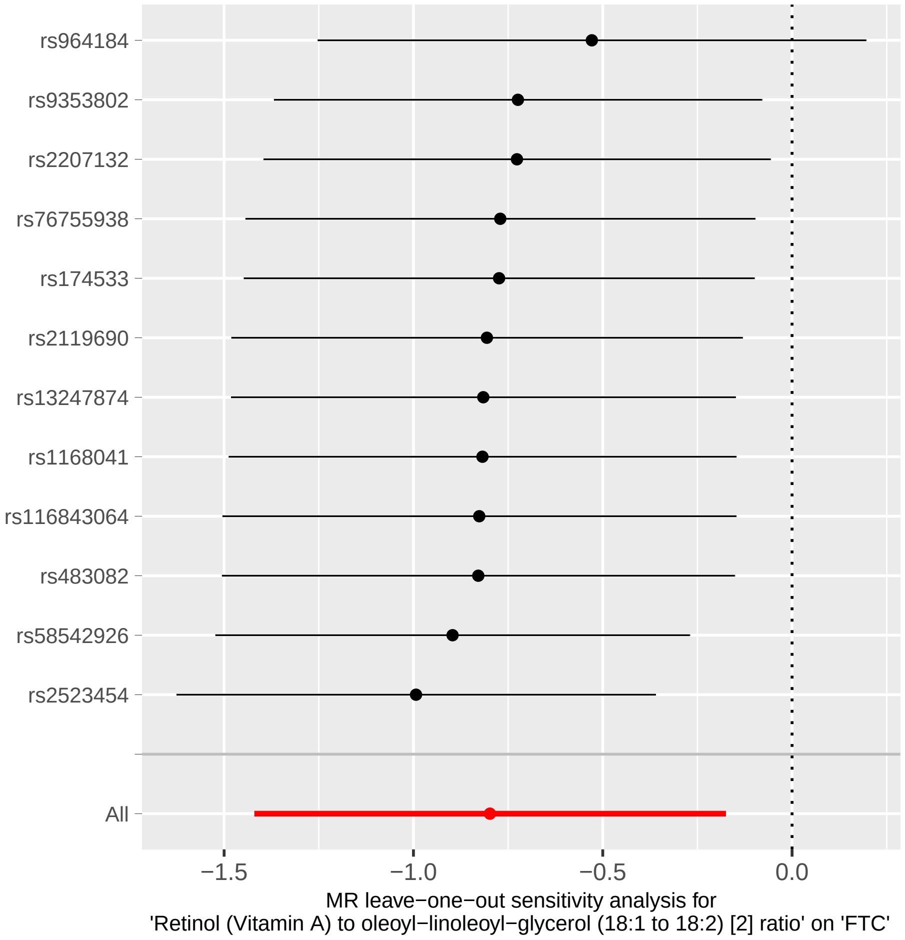
rs964184
rs9353802
rs2207132
rs76755938
rs174533
rs2119690
rs13247874
rs1168041
rs116843064
rs483082
rs58542926
rs2523454
All
−1.5
−1.0
−0.5
0.0
MR leave−one−out sensitivity analysis for
'Retinol (Vitamin A) to oleoyl−linoleoyl−glycerol (18:1 to 18:2) [2] ratio' on 'FTC'

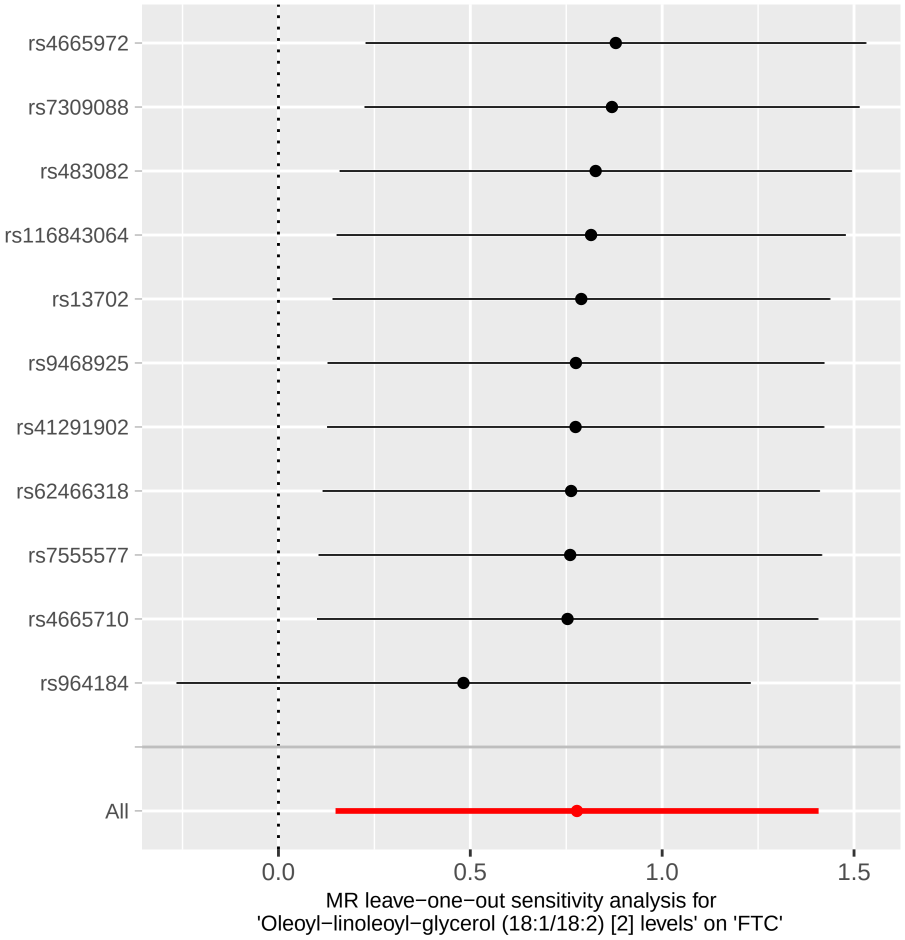
rs4665972
rs7309088
rs483082
rs116843064
rs13702
rs9468925
rs41291902
rs62466318
rs7555577
rs4665710
rs964184
All
0.0
0.5
1.0
1.5
MR leave−one−out sensitivity analysis for
'Oleoyl−linoleoyl−glycerol (18:1/18:2) [2] levels' on 'FTC'

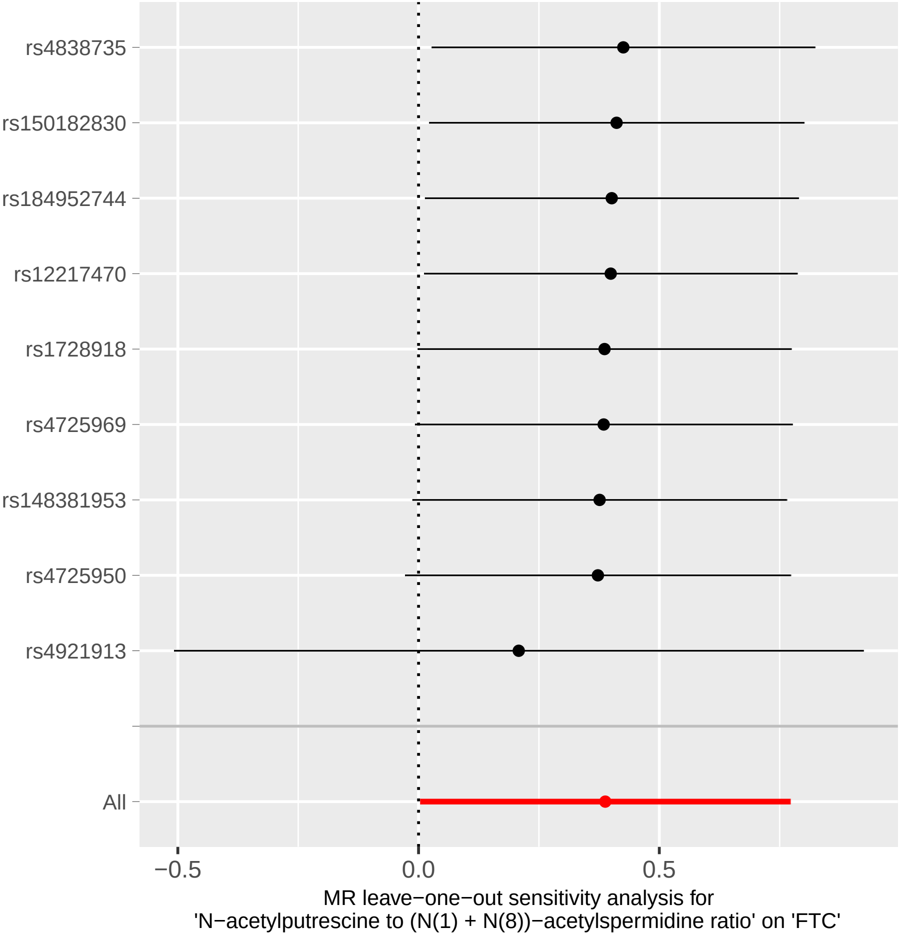

rs4838735
rs150182830
rs184952744
rs12217470
rs1728918
rs4725969
rs148381953
rs4725950
rs4921913
All
−0.5
0.0
0.5
MR leave−one−out sensitivity analysis for
'N−acetylputrescine to (N(1) + N(8))−acetylspermidine ratio' on 'FTC'

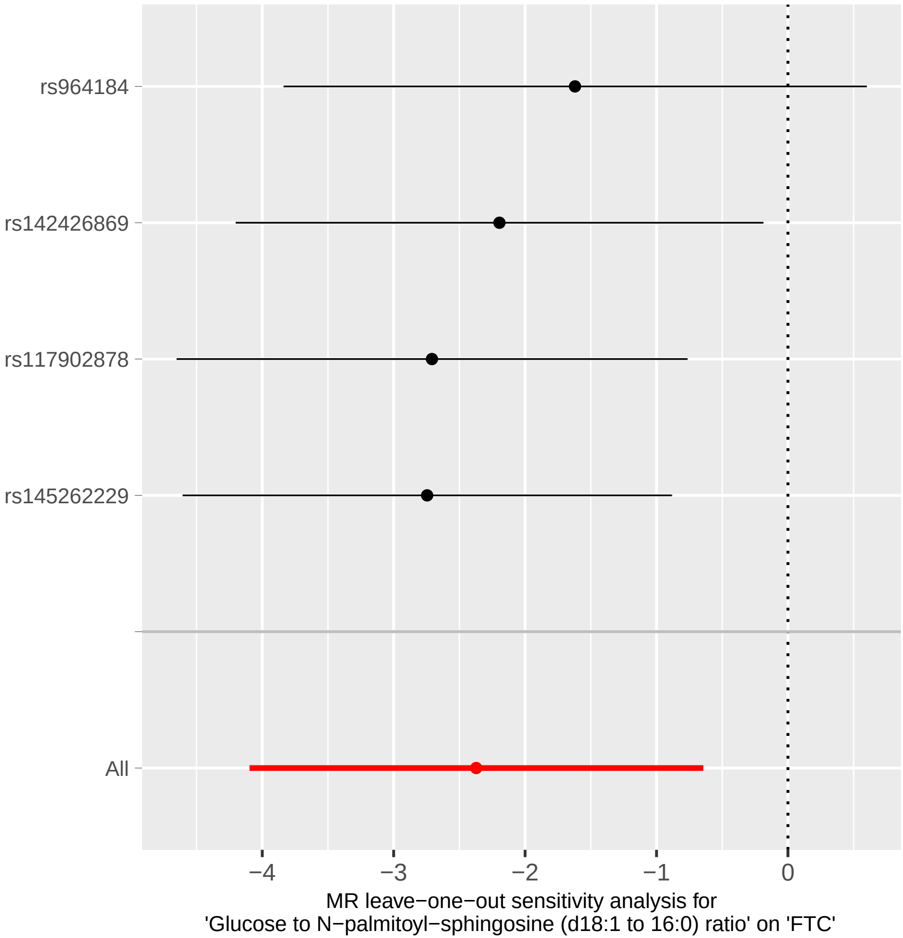
rs964184
rs142426869
rs117902878
rs145262229
All
−4
−3
−2
−1
0
MR leave−one−out sensitivity analysis for
'Glucose to N−palmitoyl−sphingosine (d18:1 to 16:0) ratio' on 'FTC'

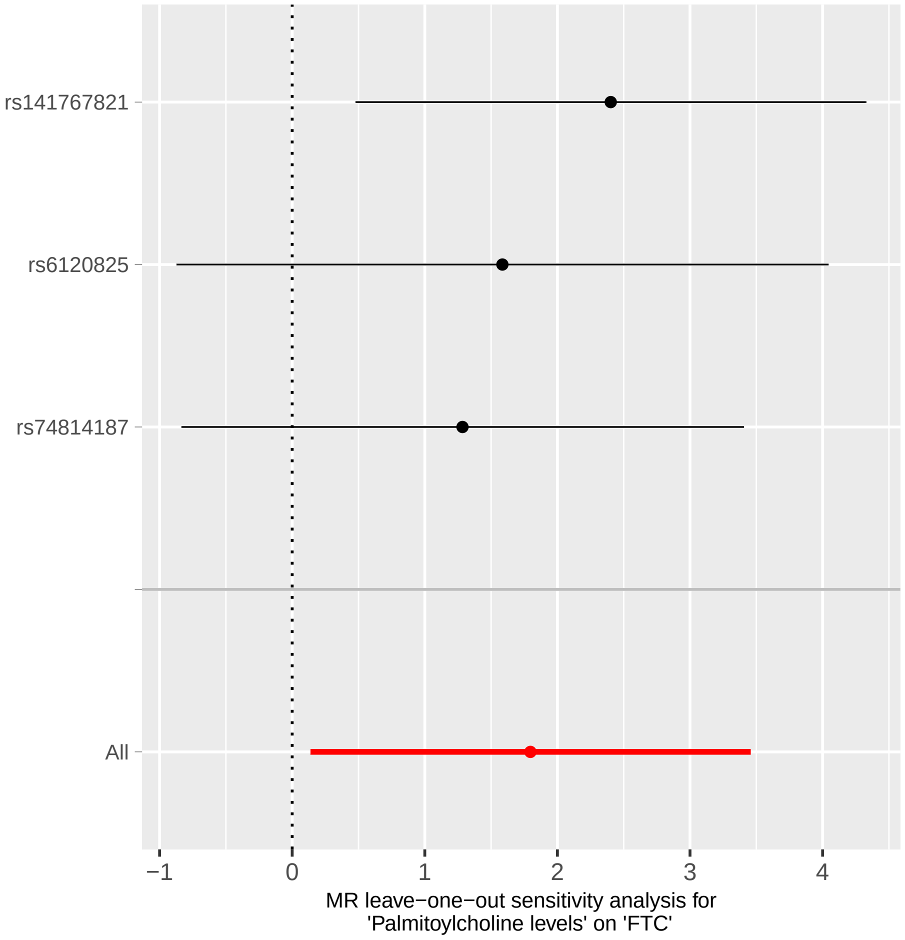

rs141767821
rs6120825
rs74814187
All
−1
0
1
2
3
4
MR leave−one−out sensitivity analysis for
'Palmitoylcholine levels' on 'FTC'

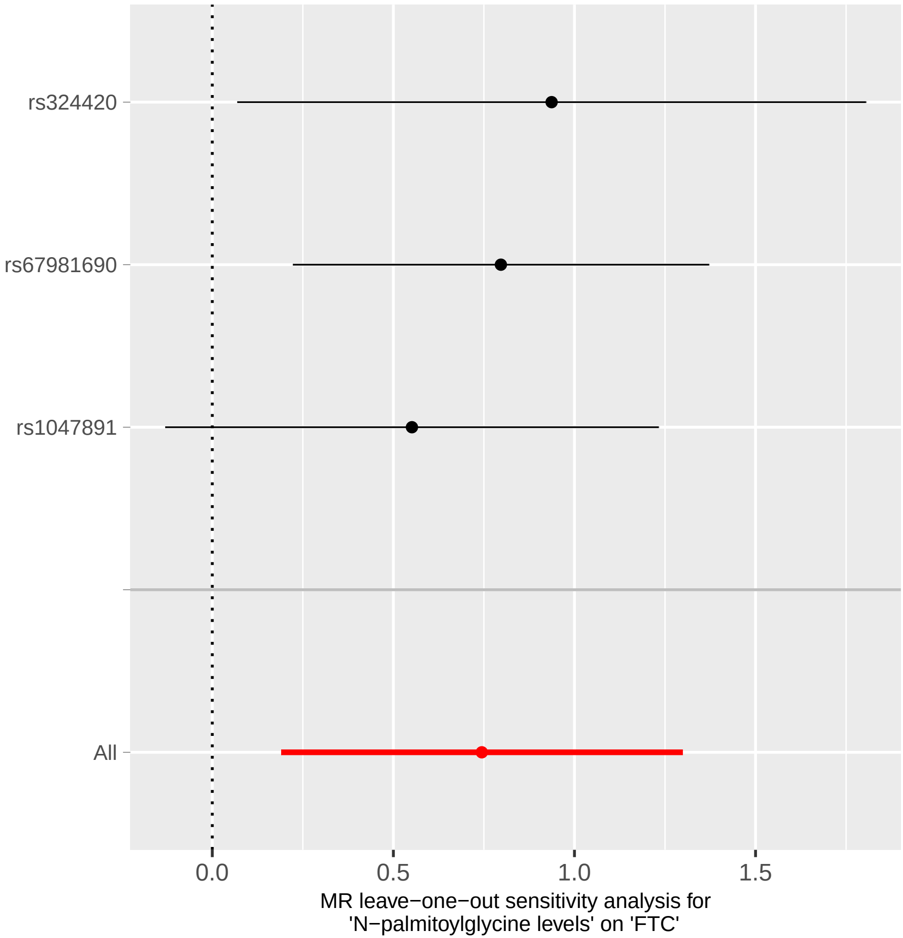

rs324420
rs67981690
rs1047891
All
0.0
0.5
1.0
1.5
MR leave−one−out sensitivity analysis for
'N−palmitoylglycine levels' on 'FTC'

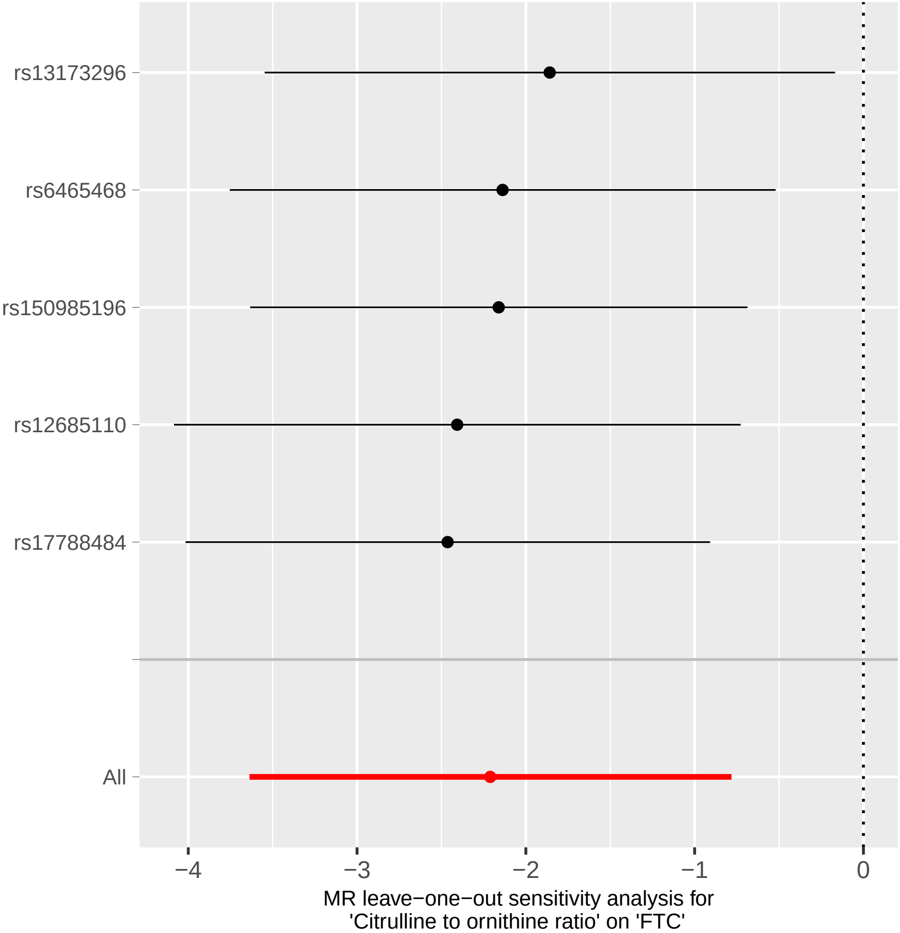

rs13173296
rs6465468
rs150985196
rs12685110
rs17788484
All
−4
−3
−2
−1
0
MR leave−one−out sensitivity analysis for
'Citrulline to ornithine ratio' on 'FTC'

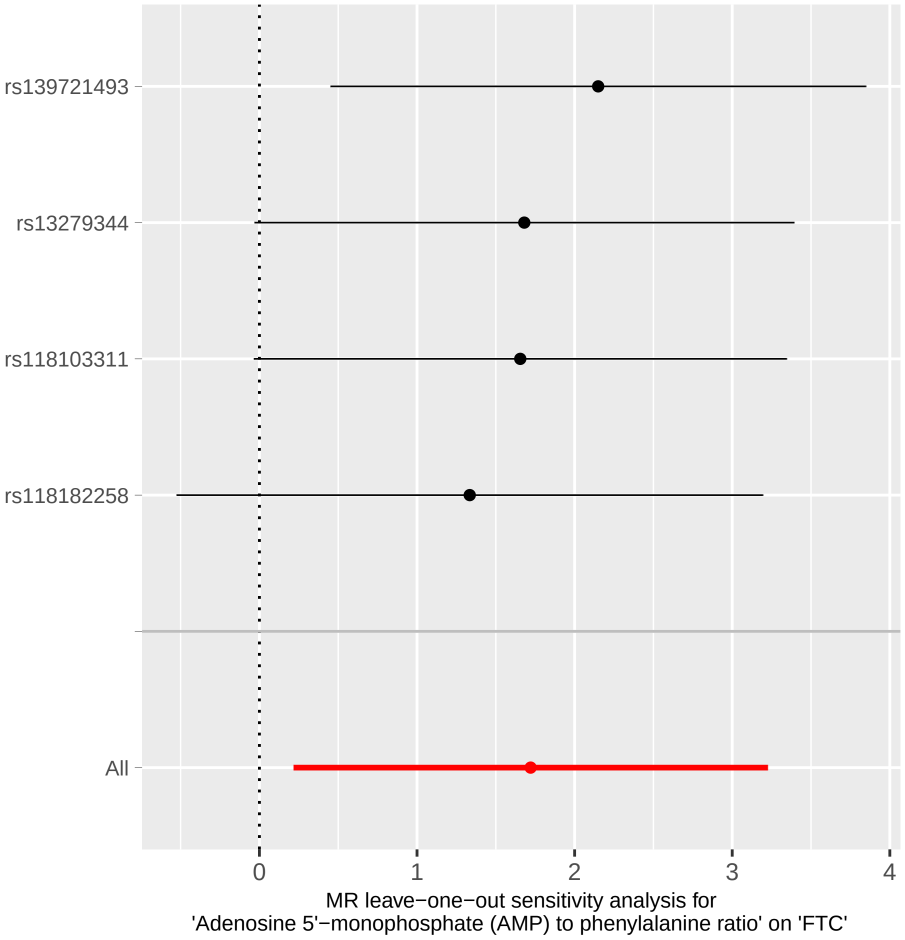
rs139721493
rs13279344
rs118103311
rs118182258
All
0
1
2
3
4
MR leave−one−out sensitivity analysis for
'Adenosine 5'−monophosphate (AMP) to phenylalanine ratio' on 'FTC'

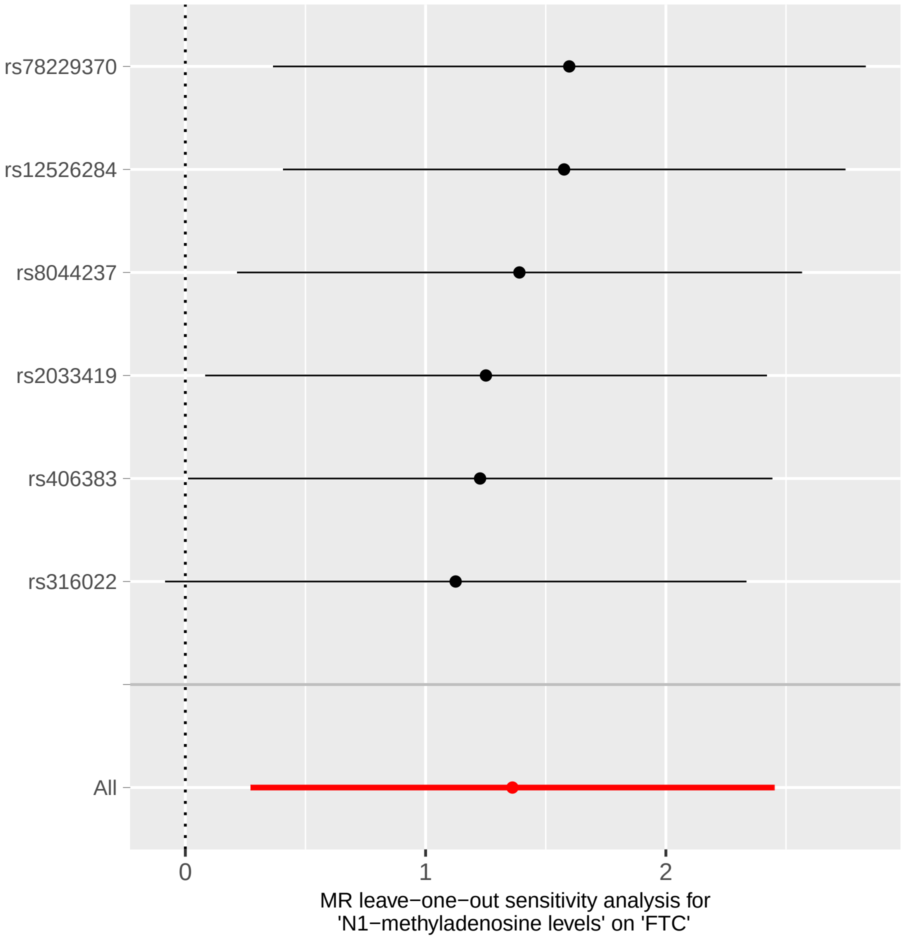

rs78229370
rs12526284
rs8044237
rs2033419
rs406383
rs316022
All
0
1
2
MR leave−one−out sensitivity analysis for
'N1−methyladenosine levels' on 'FTC'

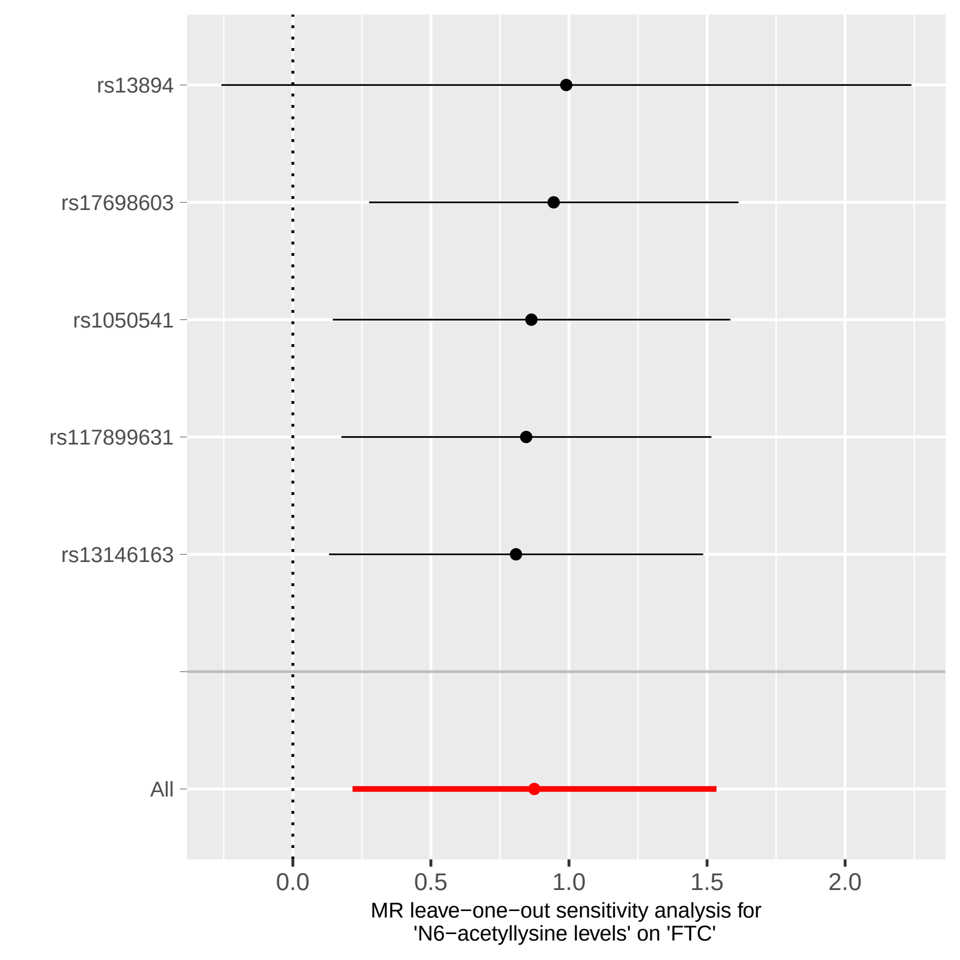

rs13894
rs17698603
rs1050541
rs117899631
rs13146163
All
0.0
0.5
1.0
1.5
2.0
MR leave−one−out sensitivity analysis for
'N6−acetyllysine levels' on 'FTC'

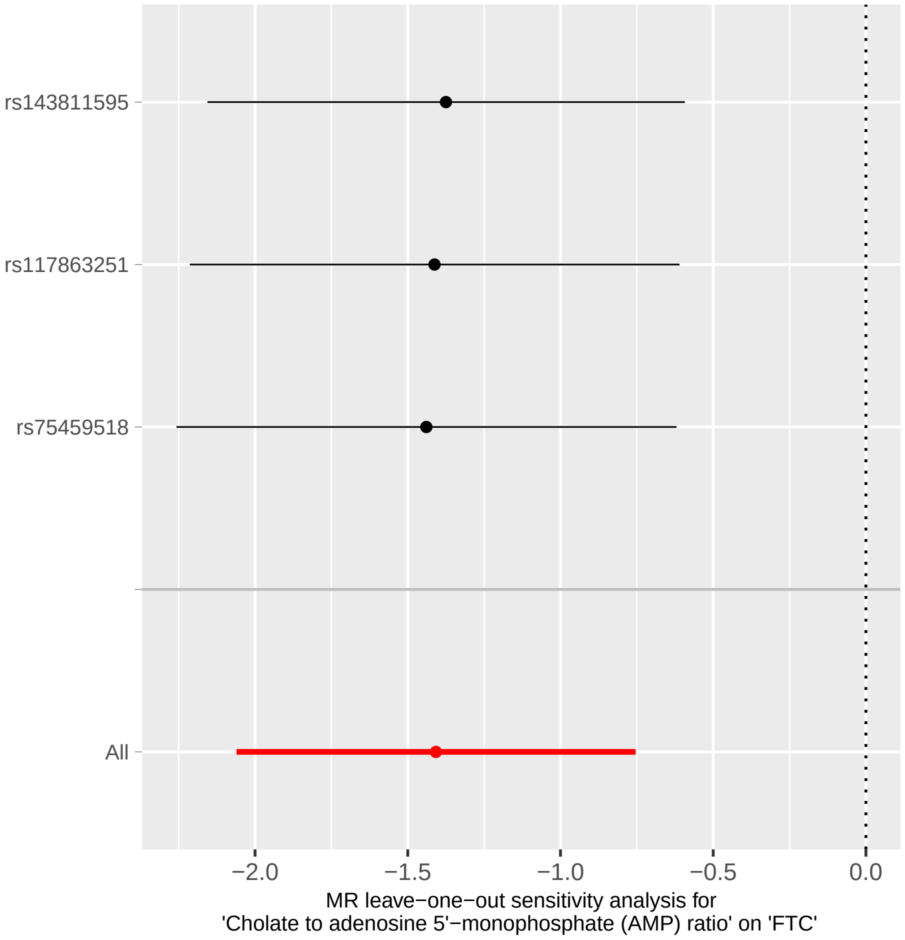

rs143811595
rs117863251
rs75459518
All
−2.0
−1.5
−1.0
−0.5
0.0
MR leave−one−out sensitivity analysis for
'Cholate to adenosine 5'−monophosphate (AMP) ratio' on 'FTC'

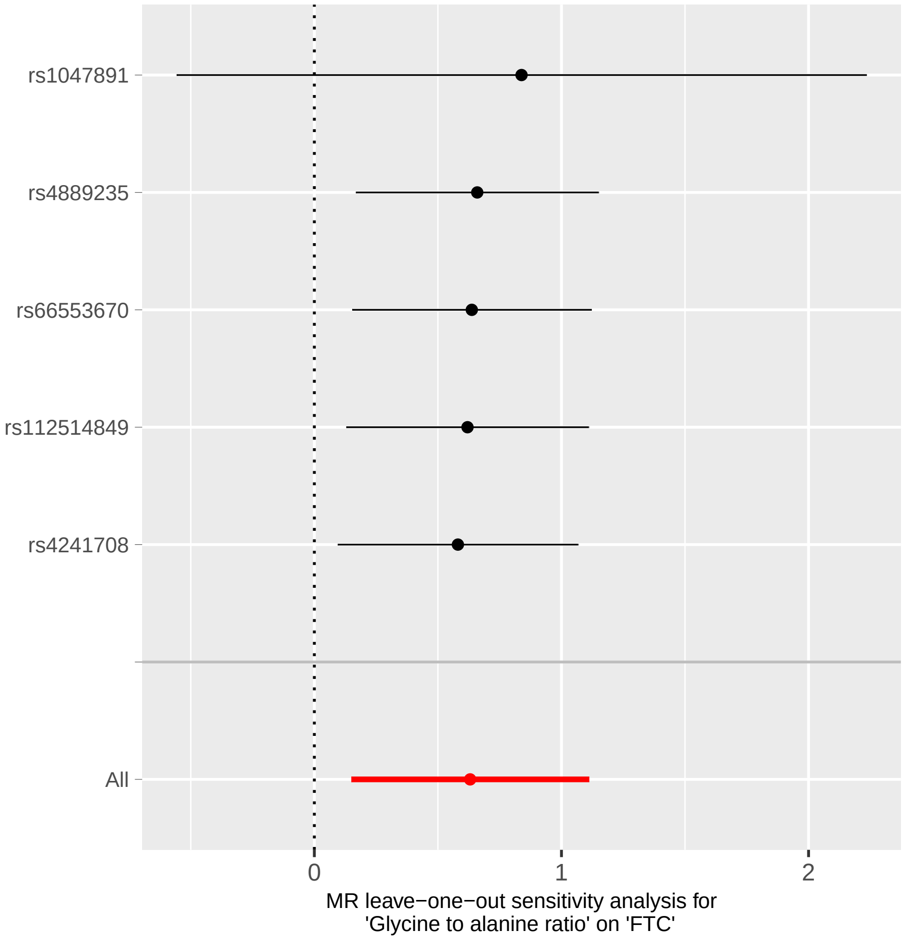
rs1047891
rs4889235
rs66553670
rs112514849
rs4241708
All
0
1
2
MR leave−one−out sensitivity analysis for
'Glycine to alanine ratio' on 'FTC'

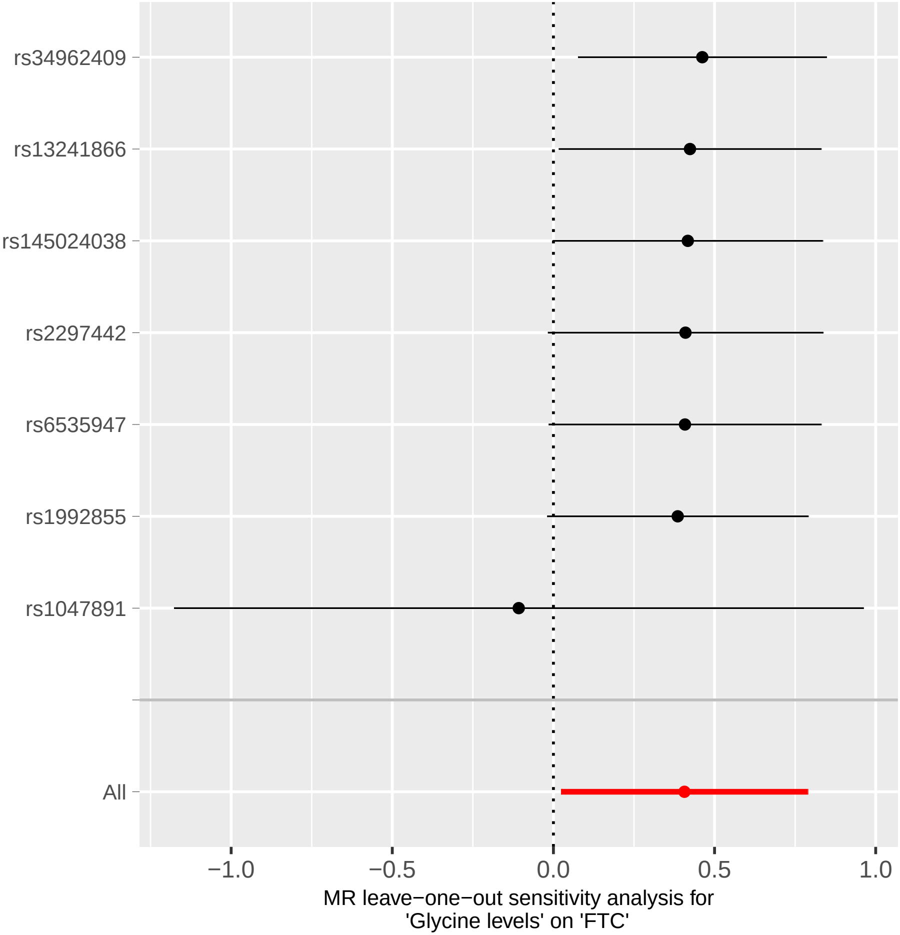
rs34962409
rs13241866
rs145024038
rs2297442
rs6535947
rs1992855
rs1047891
All
−1.0
−0.5
0.0
0.5
1.0
MR leave−one−out sensitivity analysis for
'Glycine levels' on 'FTC'

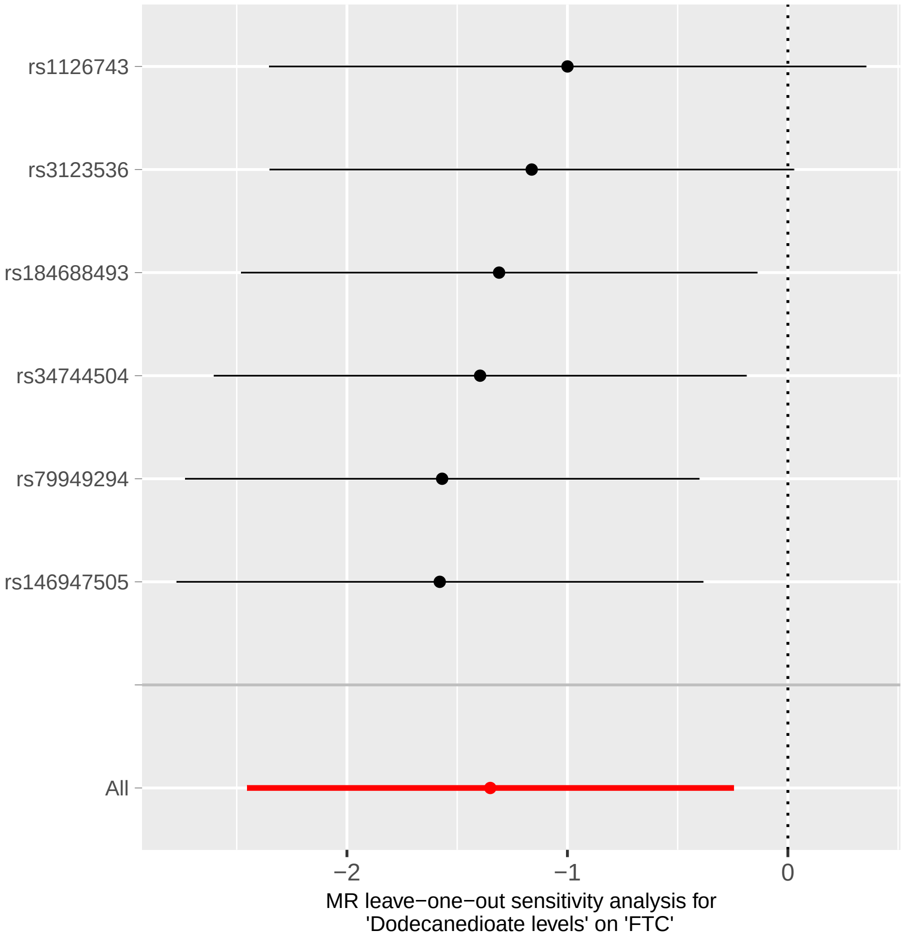
rs1126743
rs3123536
rs184688493
rs34744504
rs79949294
rs146947505
All
−2
−1
0
MR leave−one−out sensitivity analysis for
'Dodecanedioate levels' on 'FTC'

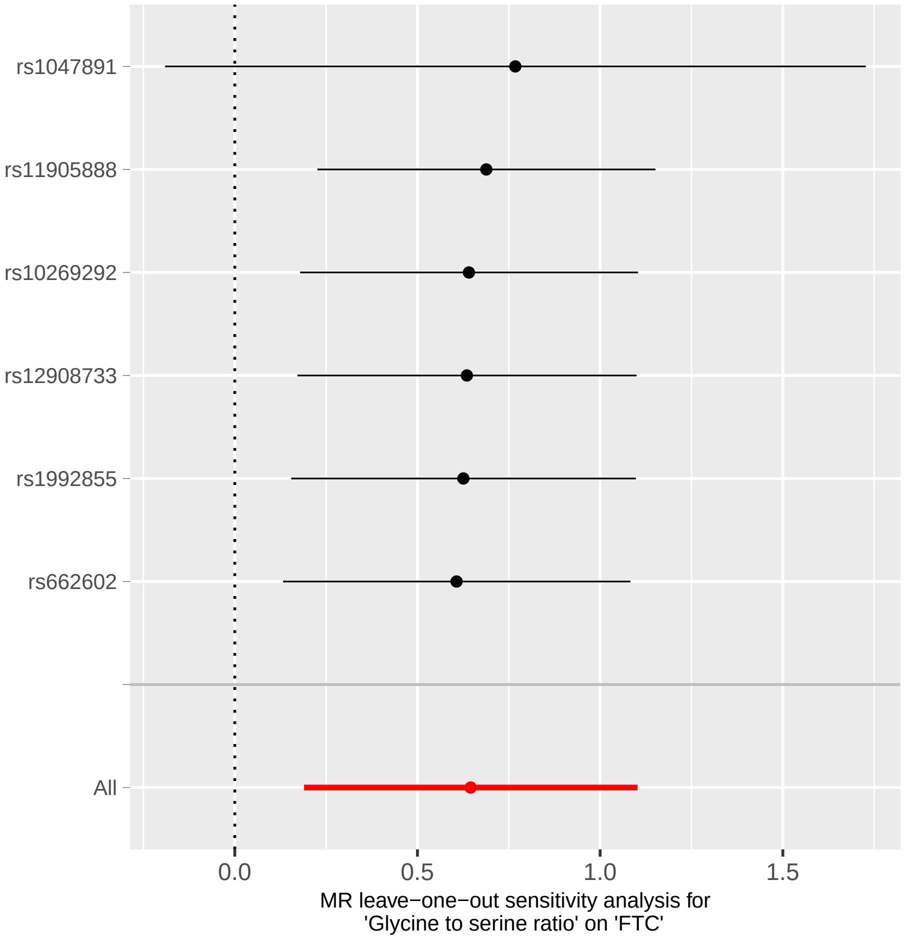
rs1047891
rs11905888
rs10269292
rs12908733
rs1992855
rs662602
All
0.0
0.5
1.0
1.5
MR leave−one−out sensitivity analysis for
'Glycine to serine ratio' on 'FTC'

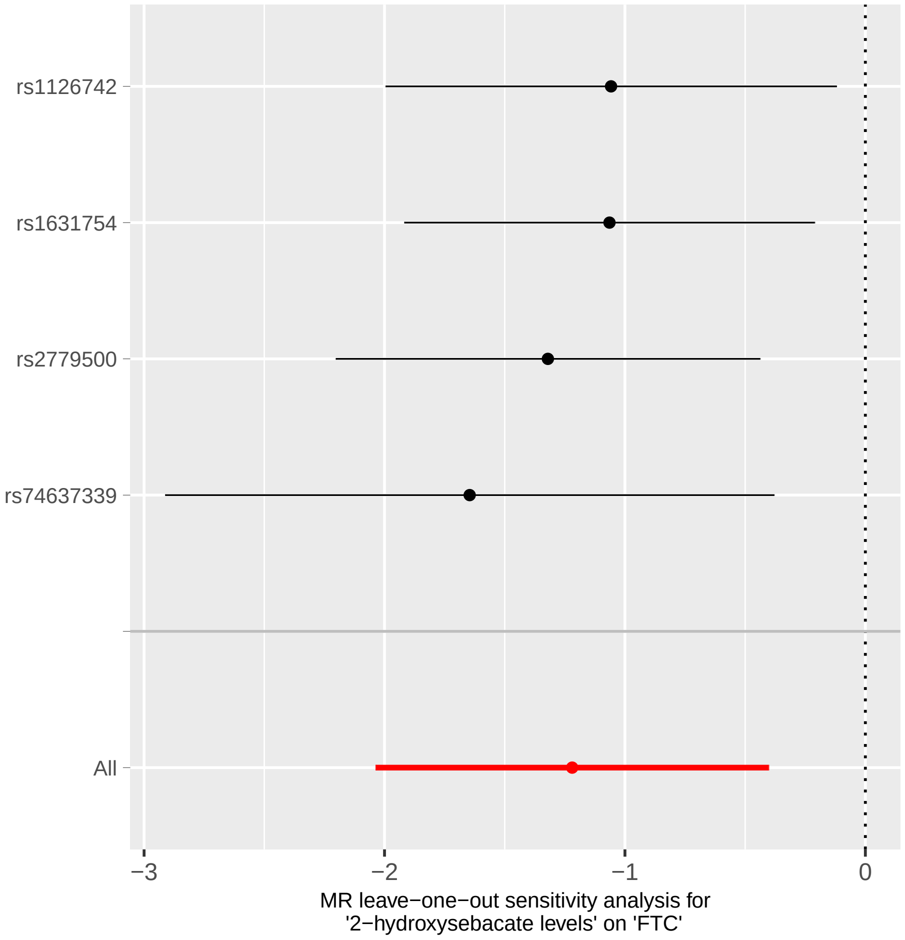
rs1126742
rs1631754
rs2779500
rs74637339
All
−3
−2
−1
0
MR leave−one−out sensitivity analysis for
'2−hydroxysebacate levels' on 'FTC'

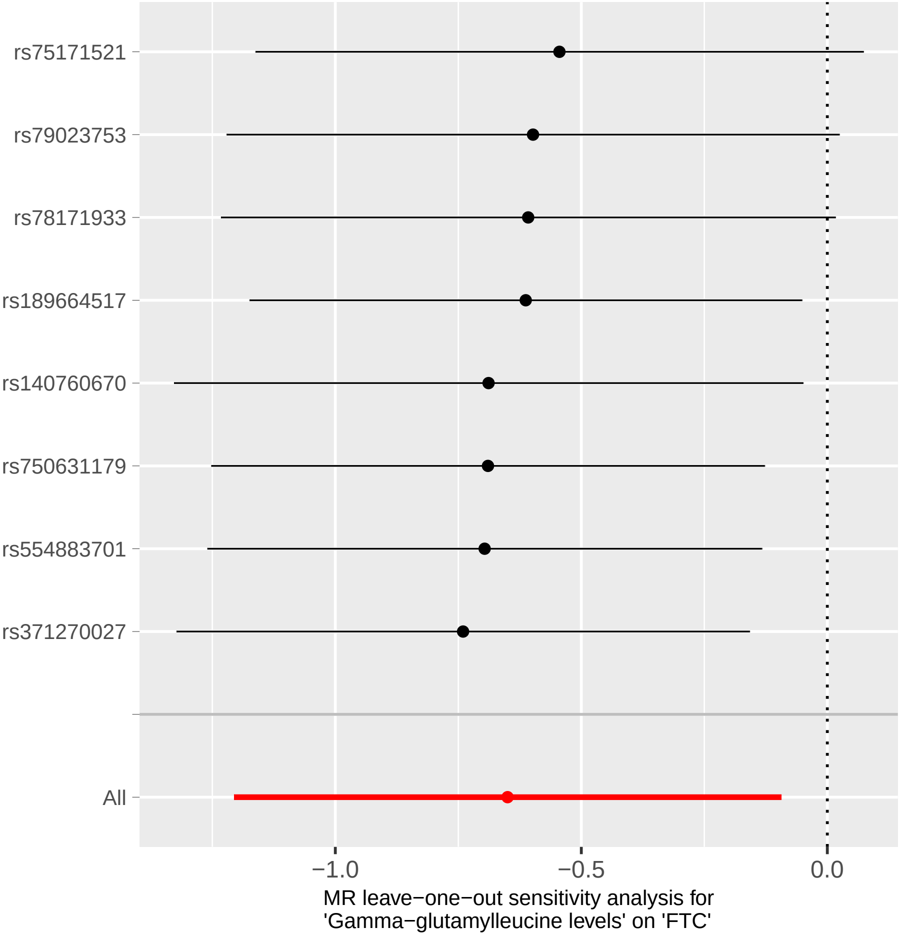

rs75171521
rs79023753
rs78171933
rs189664517
rs140760670
rs750631179
rs554883701
rs371270027
All
−1.0
−0.5
0.0
MR leave−one−out sensitivity analysis for
'Gamma−glutamylleucine levels' on 'FTC'

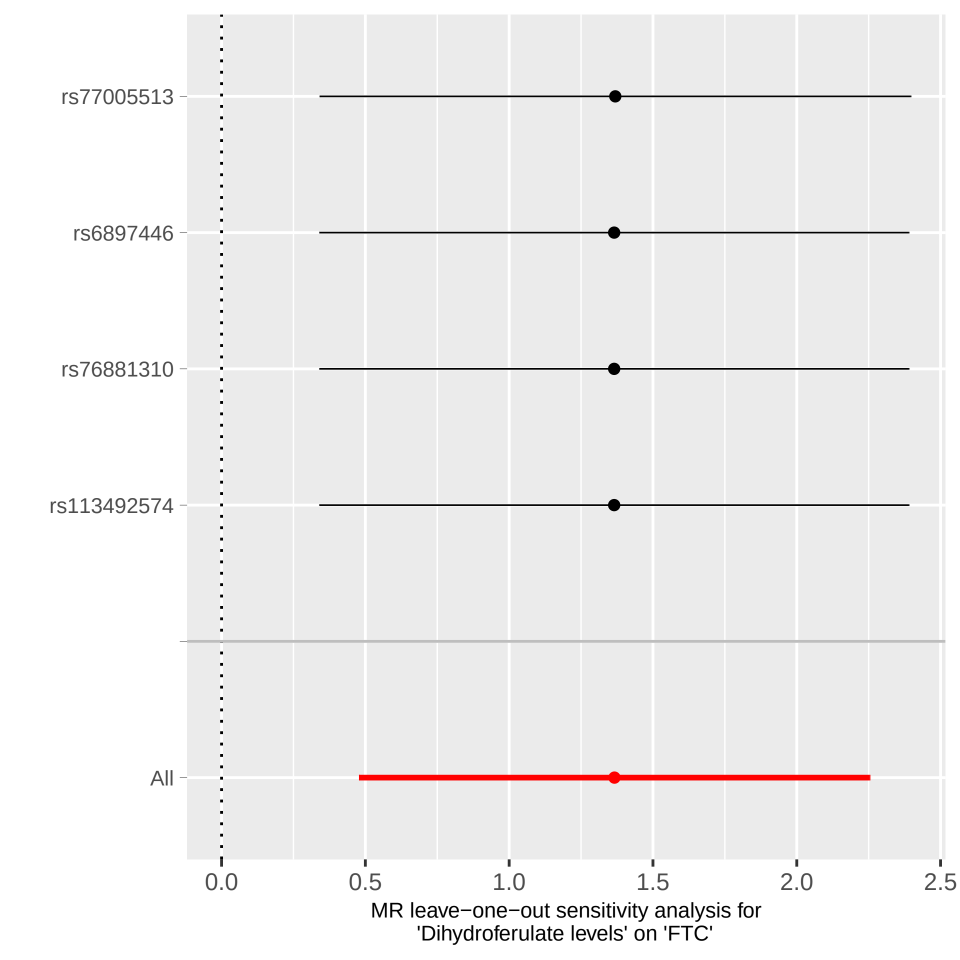

rs77005513
rs6897446
rs76881310
rs113492574
All
0.0
0.5
1.0
1.5
2.0
2.5
MR leave−one−out sensitivity analysis for
'Dihydroferulate levels' on 'FTC'

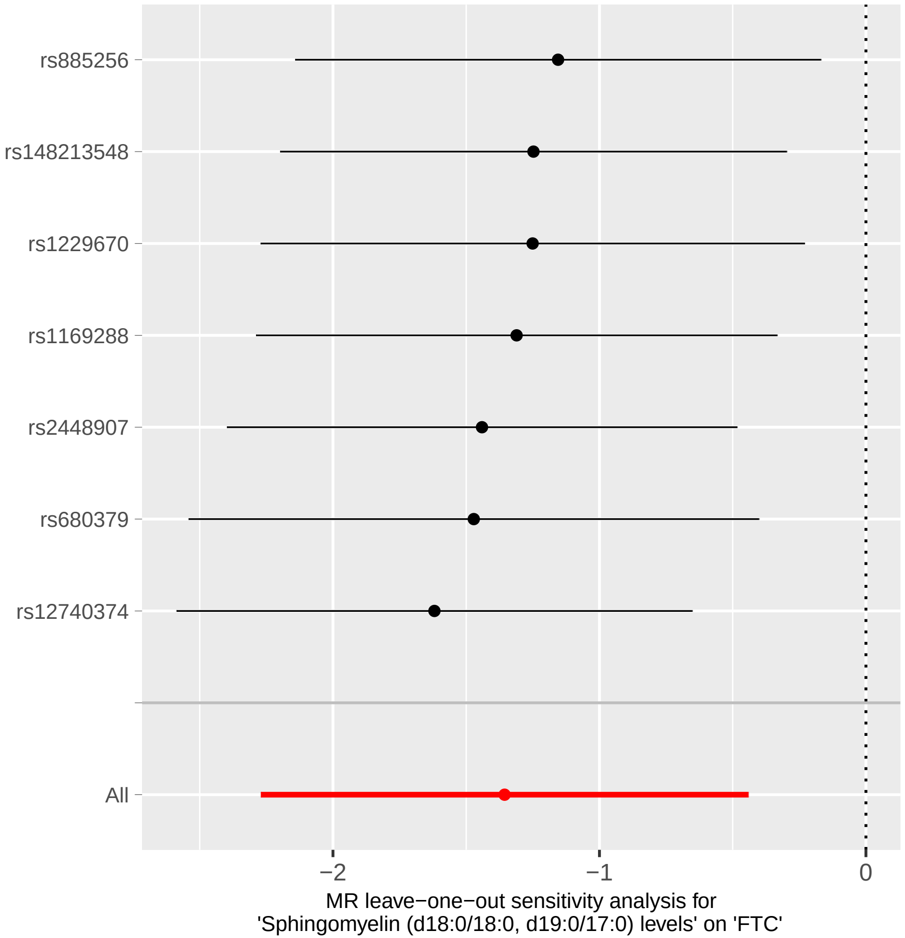

rs885256
rs148213548
rs1229670
rs1169288
rs2448907
rs680379
rs12740374
All
−2
−1
0
MR leave−one−out sensitivity analysis for
'Sphingomyelin (d18:0/18:0, d19:0/17:0) levels' on 'FTC'

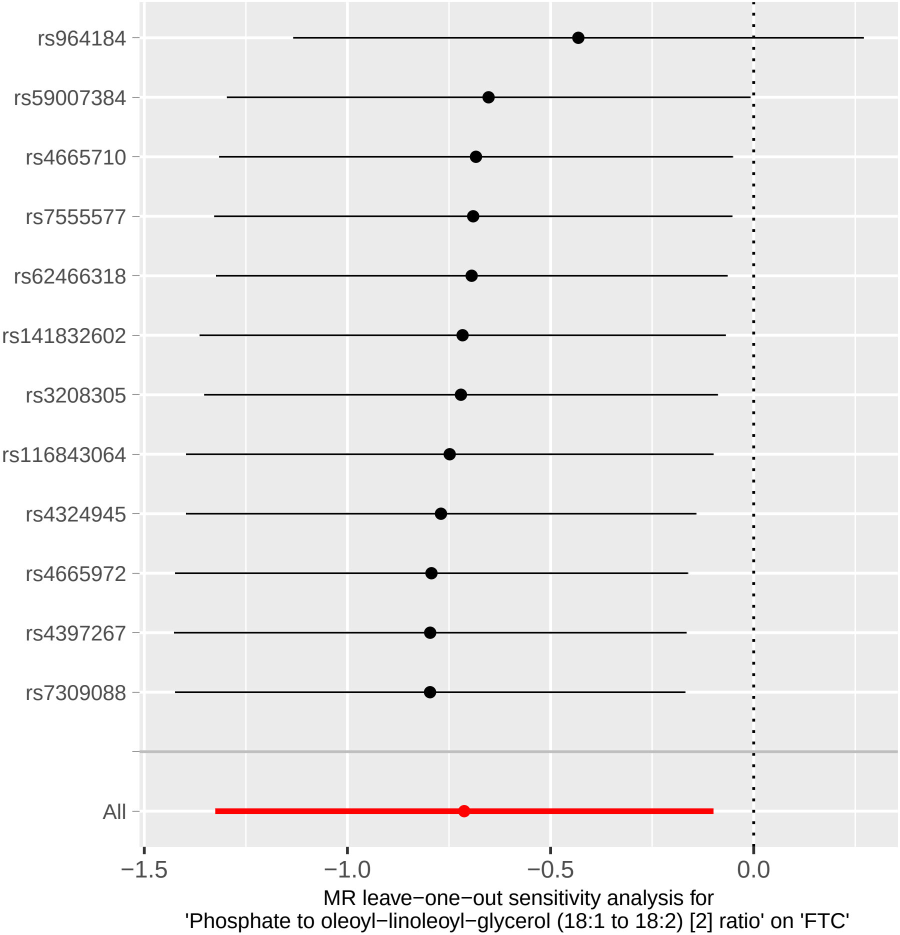
rs964184
rs59007384
rs4665710
rs7555577
rs62466318
rs141832602
rs3208305
rs116843064
rs4324945
rs4665972
rs4397267
rs7309088
All
−1.5
−1.0
−0.5
0.0
MR leave−one−out sensitivity analysis for
'Phosphate to oleoyl−linoleoyl−glycerol (18:1 to 18:2) [2] ratio' on 'FTC'

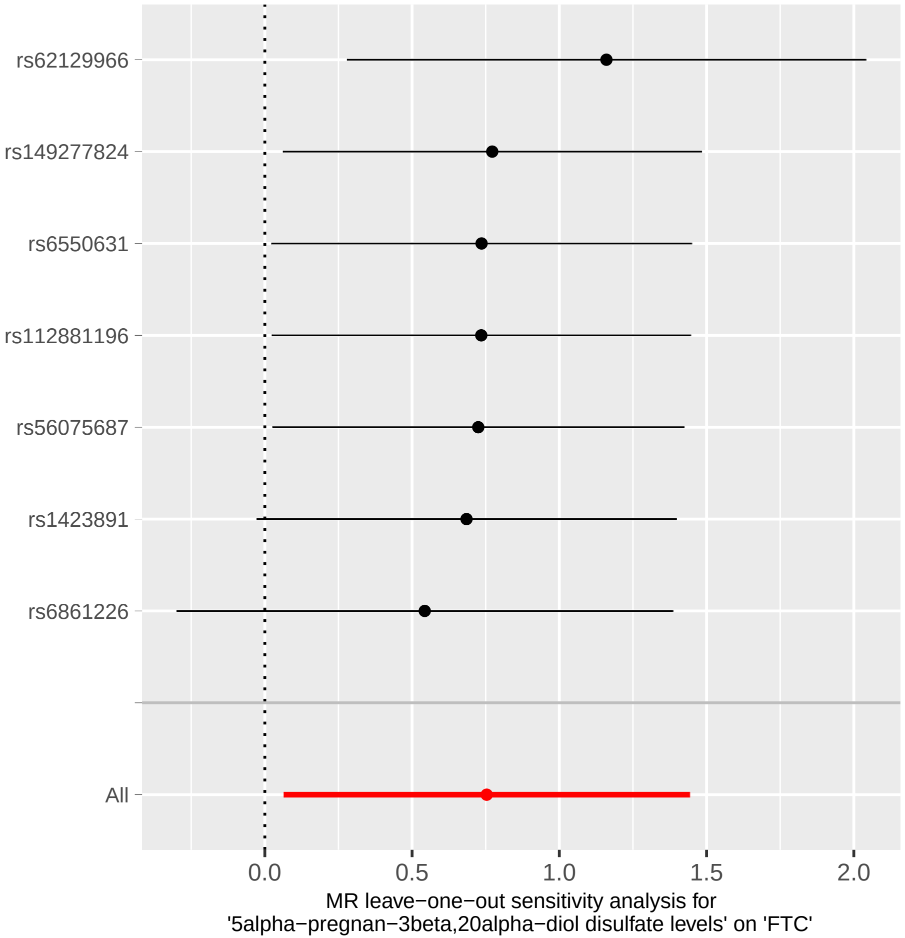

rs62129966
rs149277824
rs6550631
rs112881196
rs56075687
rs1423891
rs6861226
All
0.0
0.5
1.0
1.5
2.0
MR leave−one−out sensitivity analysis for
'5alpha−pregnan−3beta,20alpha−diol disulfate levels' on 'FTC'

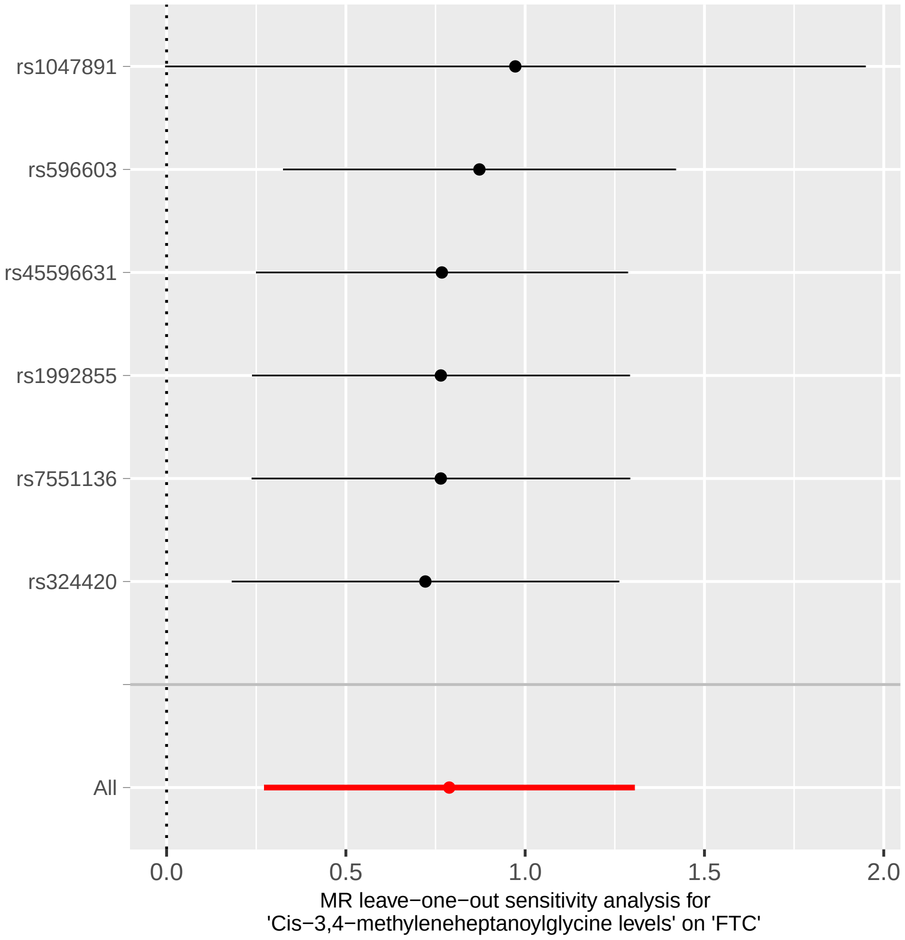

rs1047891
rs596603
rs45596631
rs1992855
rs7551136
rs324420
All
0.0
0.5
1.0
1.5
2.0
MR leave−one−out sensitivity analysis for
'Cis−3,4−methyleneheptanoylglycine levels' on 'FTC'

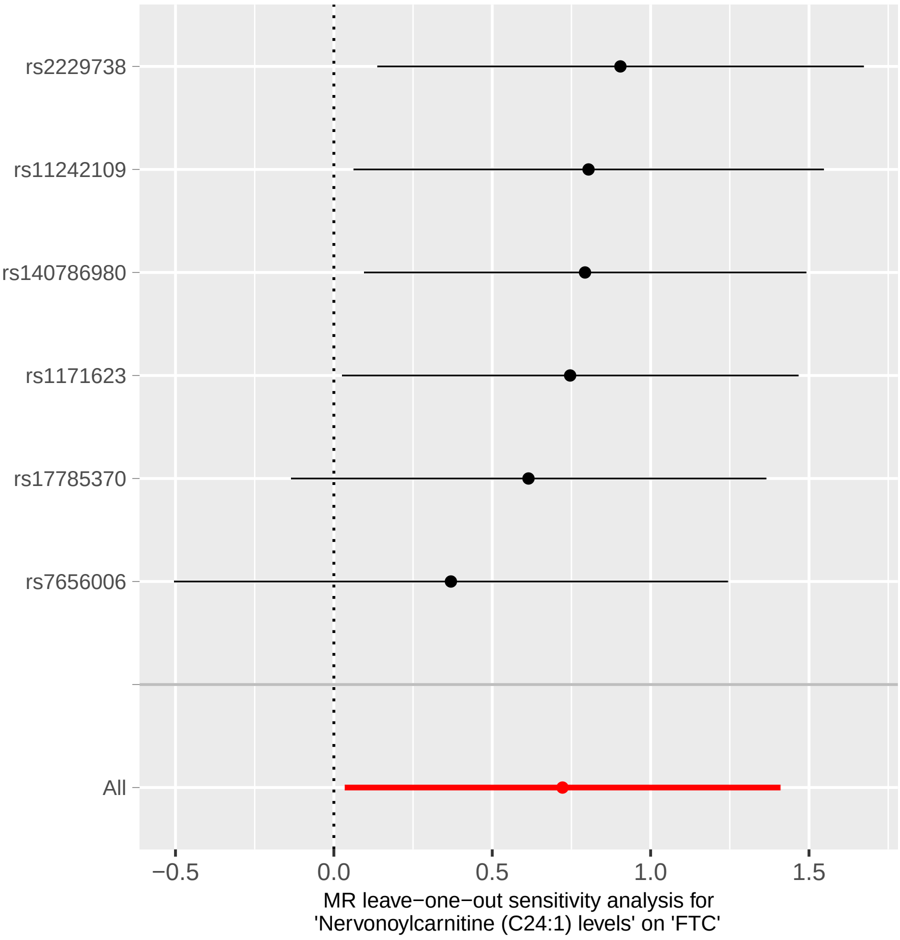
rs2229738
rs11242109
rs140786980
rs1171623
rs17785370
rs7656006
All
−0.5
0.0
0.5
1.0
1.5
MR leave−one−out sensitivity analysis for
'Nervonoylcarnitine (C24:1) levels' on 'FTC'

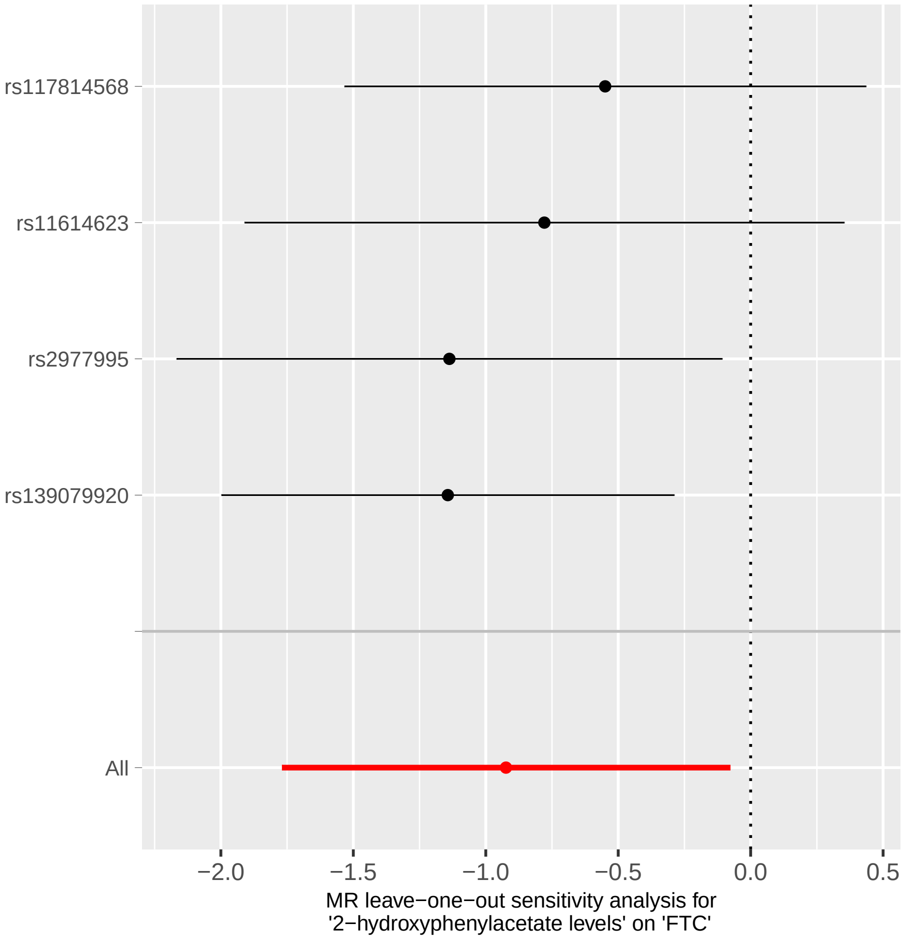
rs117814568
rs11614623
rs2977995
rs139079920
All
−2.0
−1.5
−1.0
−0.5
0.0
0.5
MR leave−one−out sensitivity analysis for
'2−hydroxyphenylacetate levels' on 'FTC'

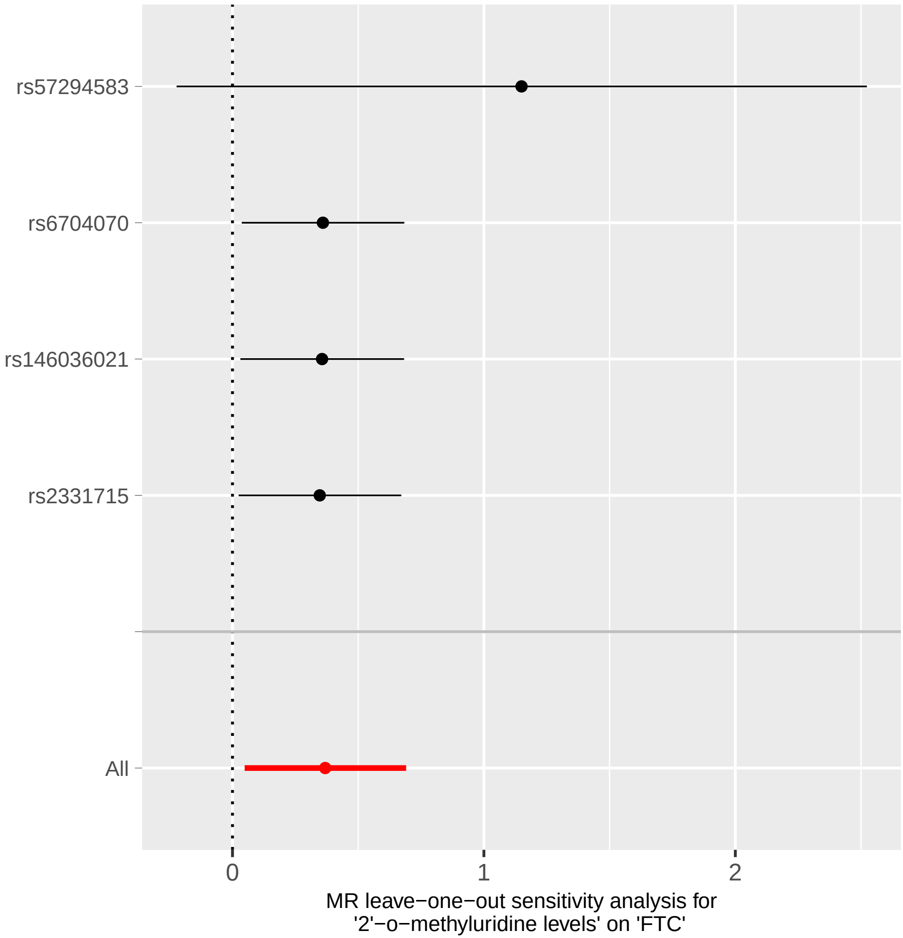

rs57294583
rs6704070
rs146036021
rs2331715
All
0
1
2
MR leave−one−out sensitivity analysis for
'2'−o−methyluridine levels' on 'FTC'

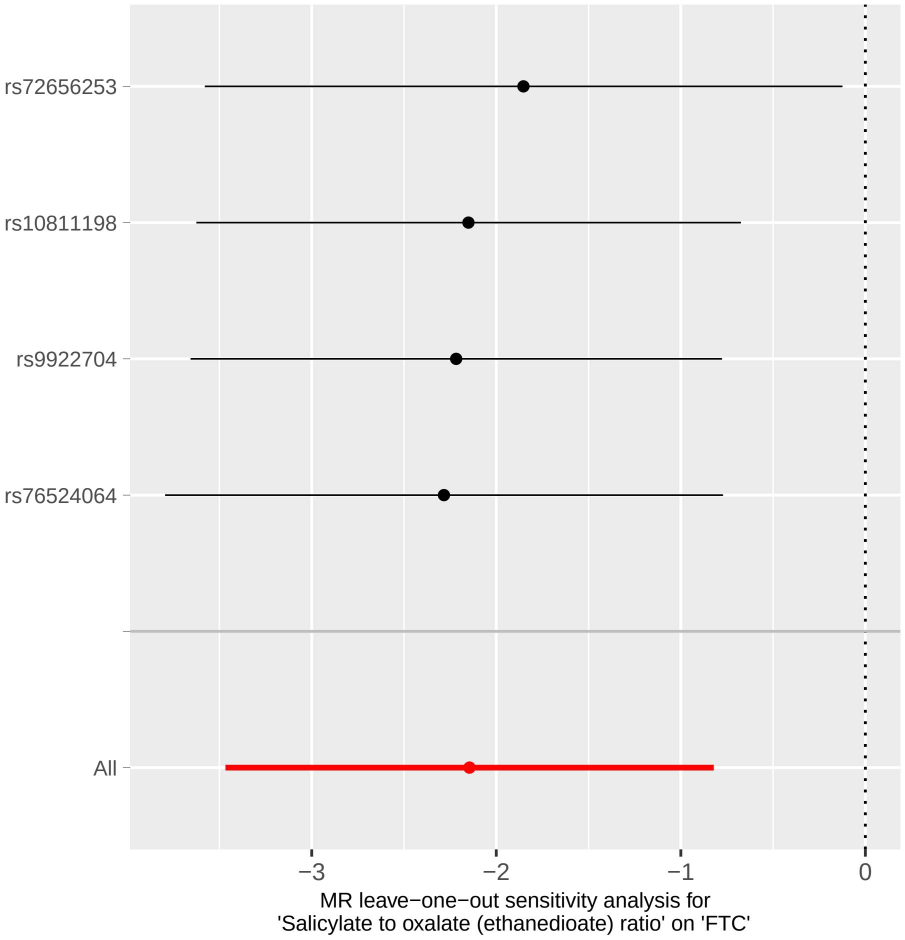
rs72656253
rs10811198
rs9922704
rs76524064
All
−3
−2
−1
0
MR leave−one−out sensitivity analysis for
'Salicylate to oxalate (ethanedioate) ratio' on 'FTC'

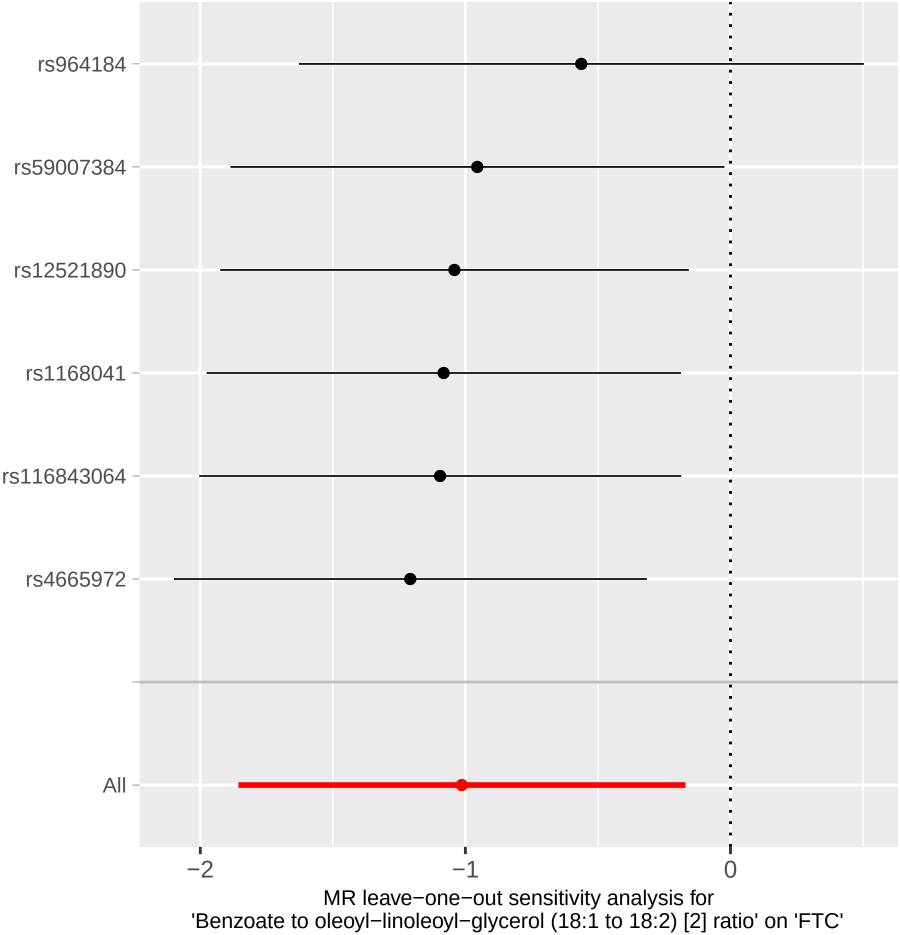
rs964184
rs59007384
rs12521890
rs1168041
rs116843064
rs4665972
All
−2
−1
0
MR leave−one−out sensitivity analysis for
'Benzoate to oleoyl−linoleoyl−glycerol (18:1 to 18:2) [2] ratio' on 'FTC'

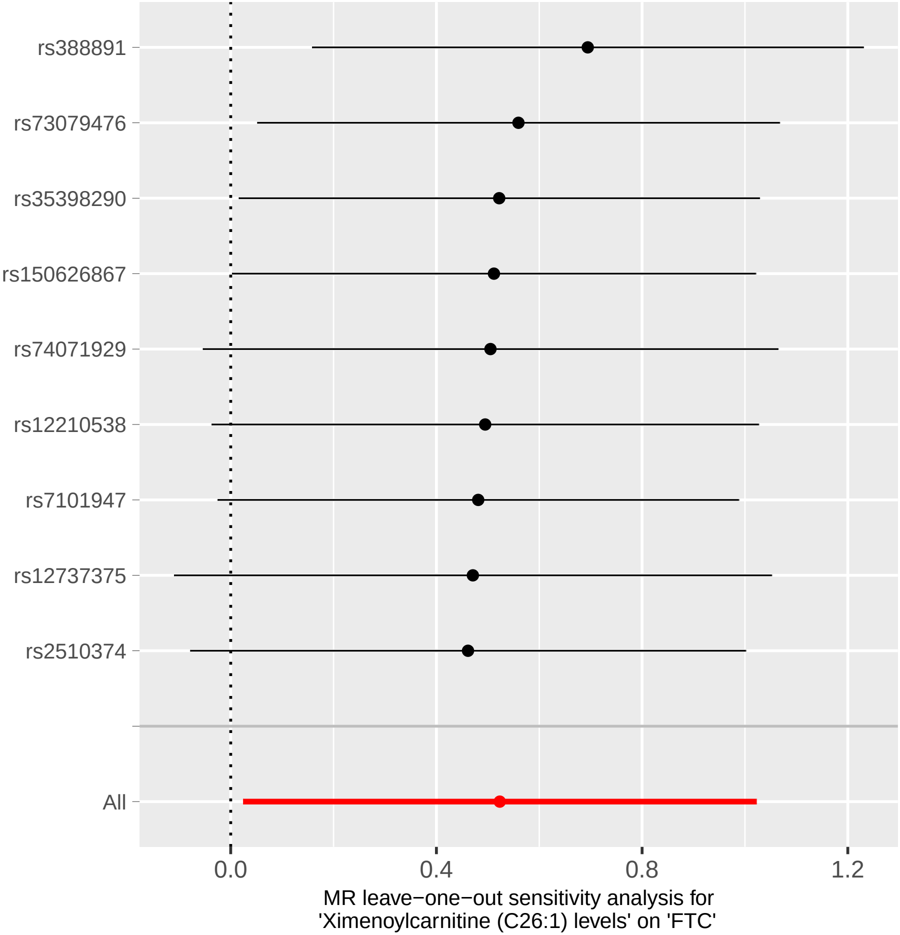
rs388891
rs73079476
rs35398290
rs150626867
rs74071929
rs12210538
rs7101947
rs12737375
rs2510374
All
0.0
0.4
0.8
1.2
MR leave−one−out sensitivity analysis for
'Ximenoylcarnitine (C26:1) levels' on 'FTC'

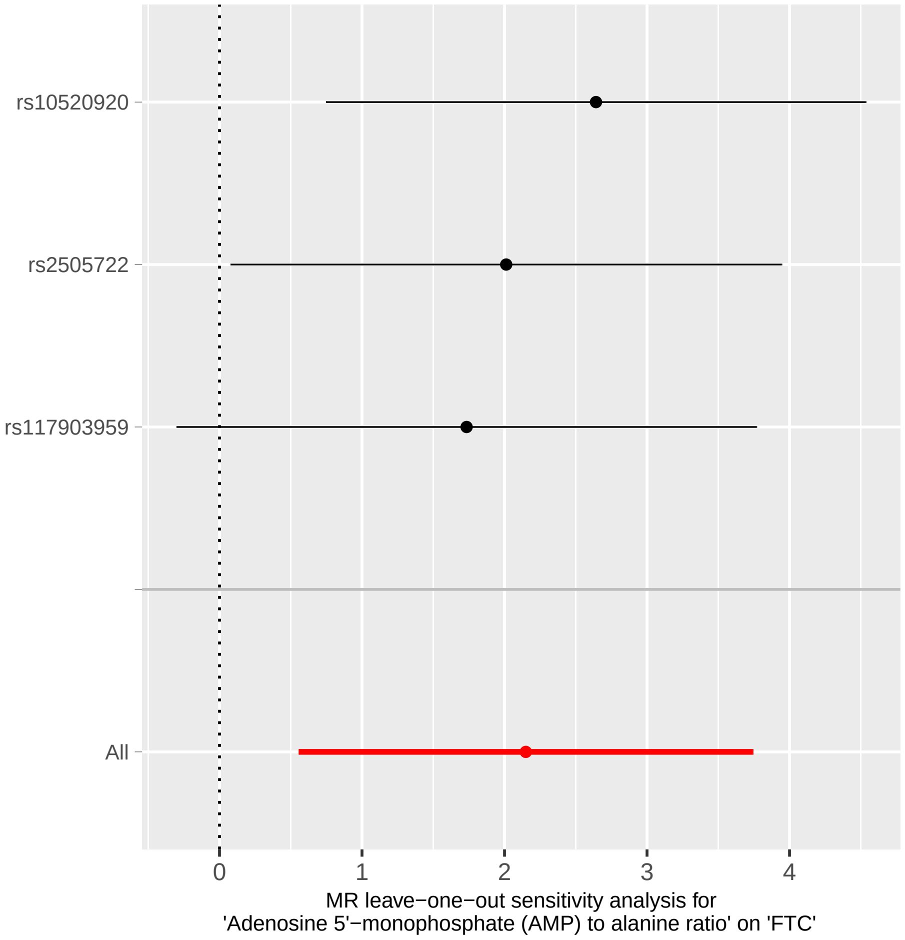

rs10520920
rs2505722
rs117903959
All
0
1
2
3
4
MR leave−one−out sensitivity analysis for
'Adenosine 5'−monophosphate (AMP) to alanine ratio' on 'FTC'

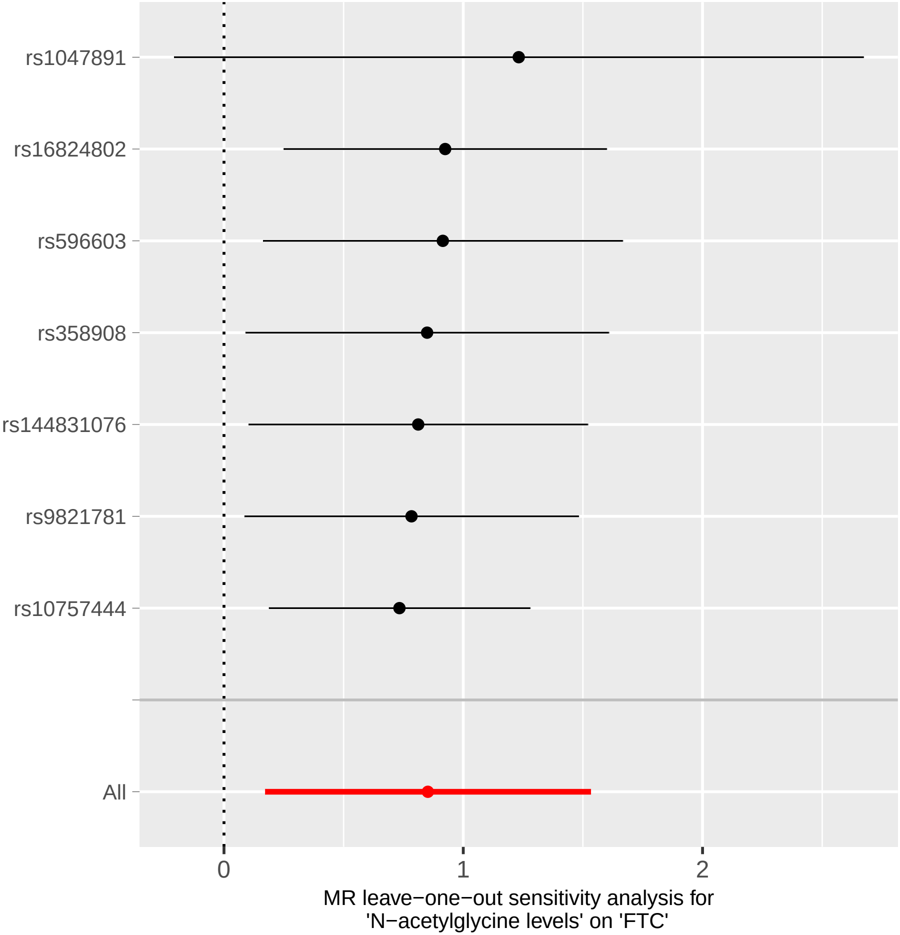
rs1047891
rs16824802
rs596603
rs358908
rs144831076
rs9821781
rs10757444
All
0
1
2
MR leave−one−out sensitivity analysis for
'N−acetylglycine levels' on 'FTC'

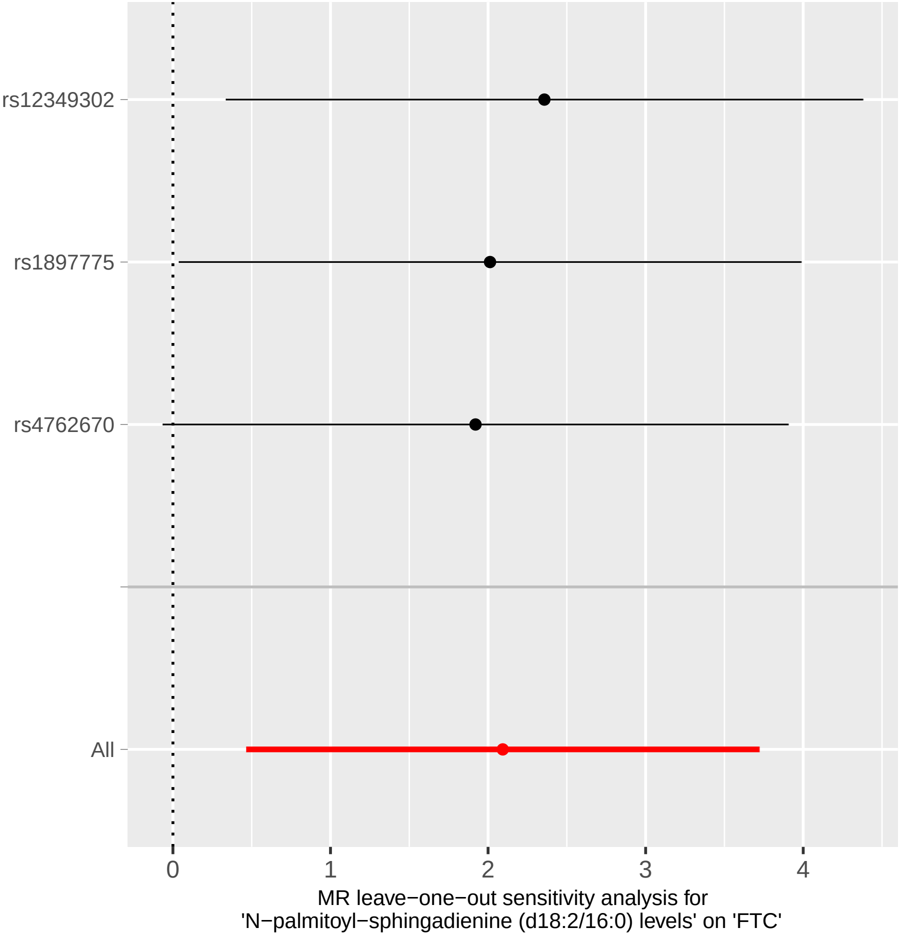

rs12349302
rs1897775
rs4762670
All
0
1
2
3
4
MR leave−one−out sensitivity analysis for
'N−palmitoyl−sphingadienine (d18:2/16:0) levels' on 'FTC'
