# Supplementary material for: Blood metabolites reflect the effect of gut microbiota on differentiated thyroid cancer: a Mendelian randomization analysis
Source: BMC Cancer. 2025 Feb 28;25:368. doi: 10.1186/s12885-025-13598-y (PMC11869591; doi:10.1186/s12885-025-13598-y)
Supplement: Supplementary file 3 — Supplementary Material 3. [file 12885_2025_13598_MOESM3_ESM.zip › Figure S5 Funnel plots for MR causal effects of microbiota abundance and metabolism on PTC.pdf]

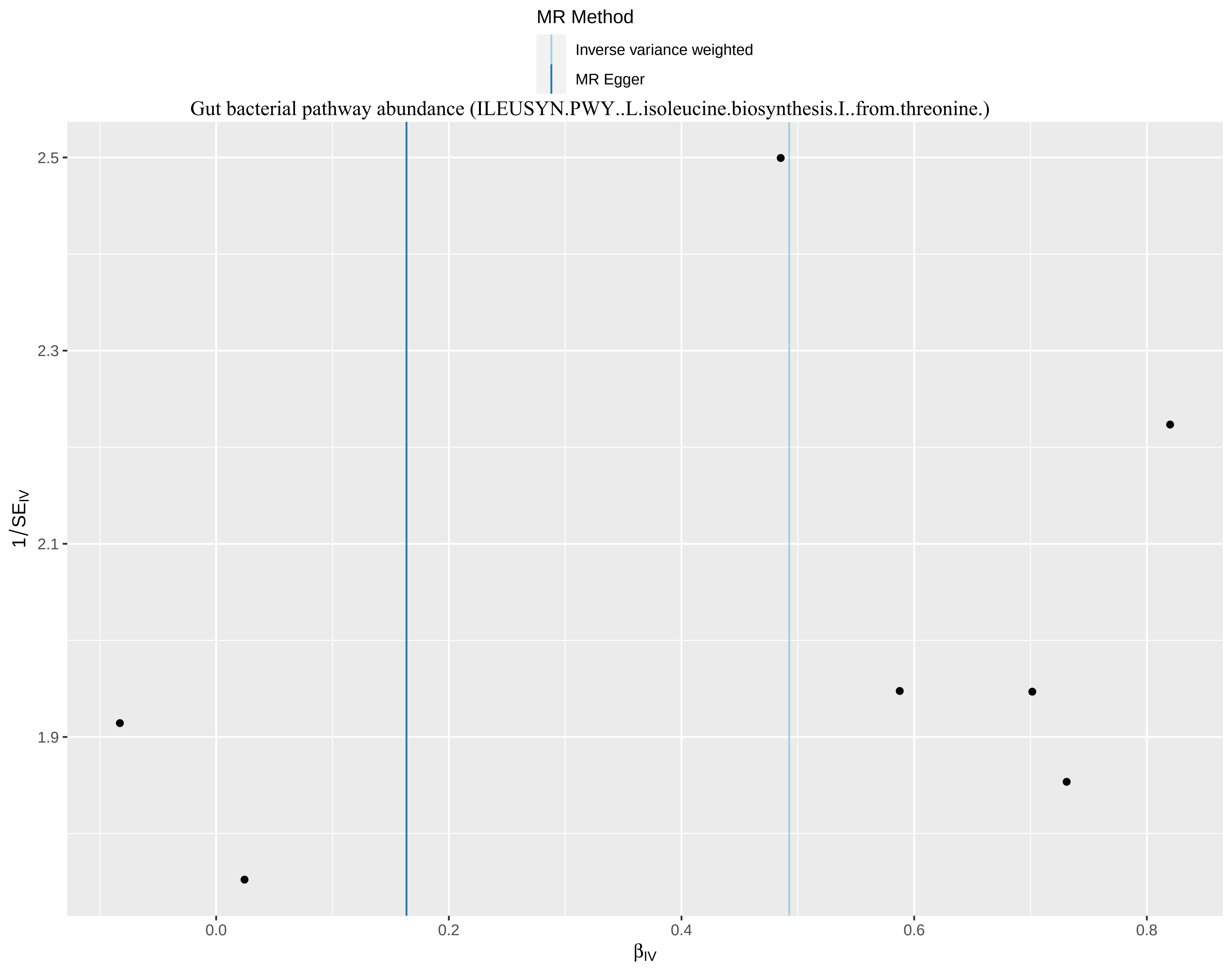

MR Method
Inverse variance weighted
MR Egger
Gut bacterial pathway abundance (ILEUSYN.PWY..L.isoleucine.biosynthesis.I..from.threonine.)
2.5
2.3
2.1
1.9
1/SE_IV
0.0
0.2
0.4
0.6
0.8
β_IV

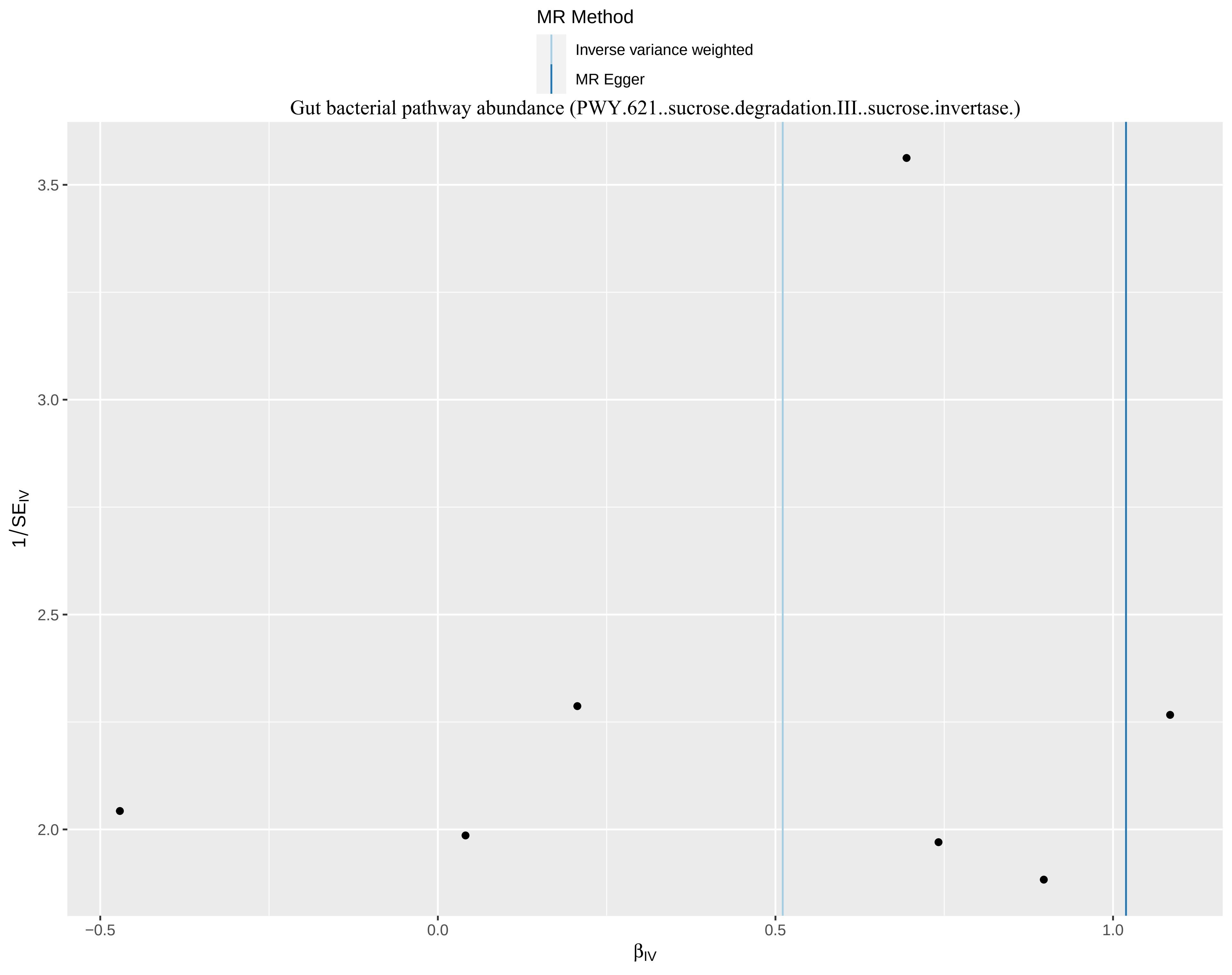

MR Method
Inverse variance weighted
MR Egger
Gut bacterial pathway abundance (PWY.621..sucrose.degradation.III..sucrose.invertase.)
3.5
3.0
2.5
2.0
1/SE_IV
−0.5
0.0
0.5
1.0
β_IV

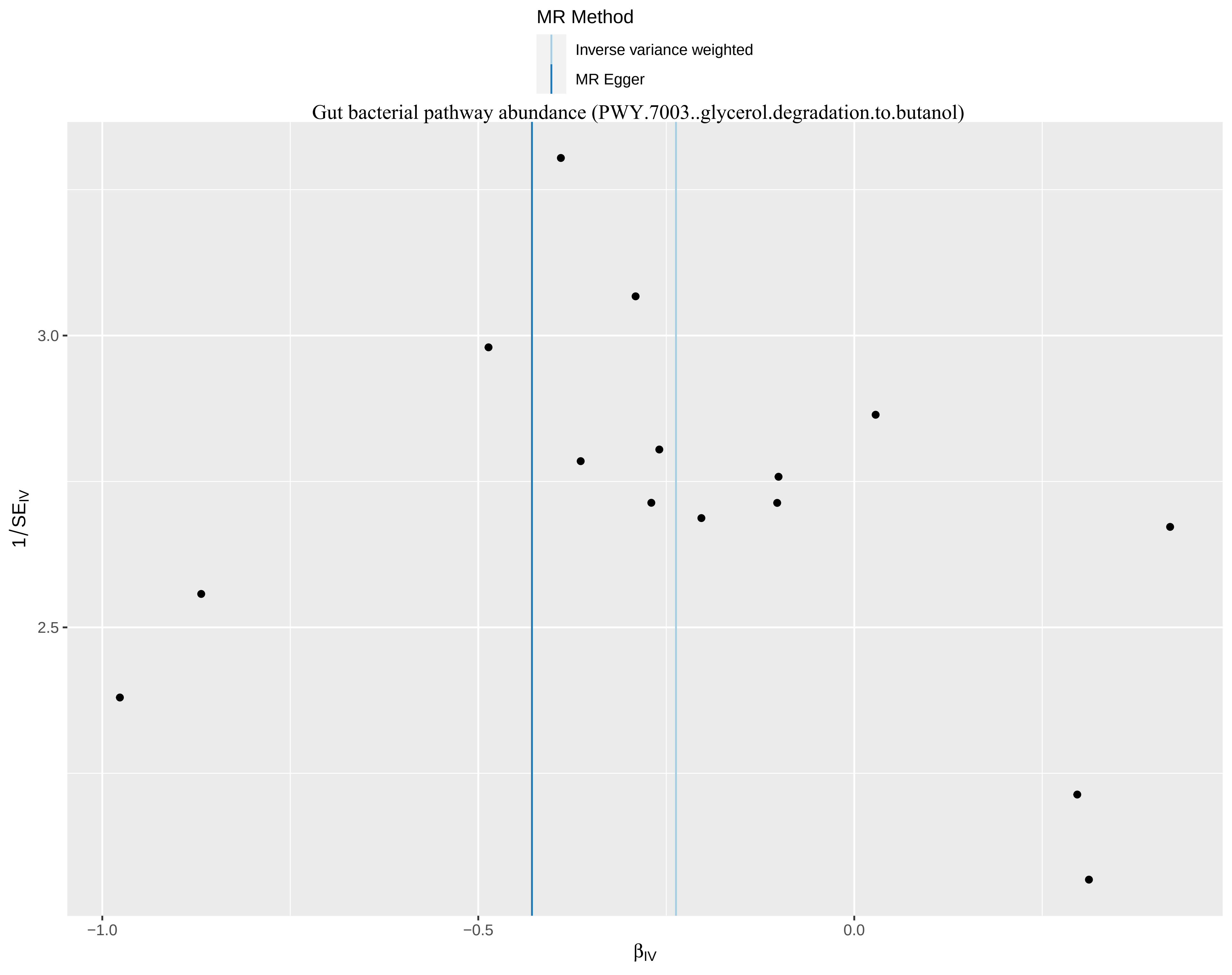
MR Method
Inverse variance weighted
MR Egger
Gut bacterial pathway abundance (PWY.7003..glycerol.degradation.to.butanol)
3.0
2.5
1/SE_IV
−1.0
−0.5
0.0
β_IV

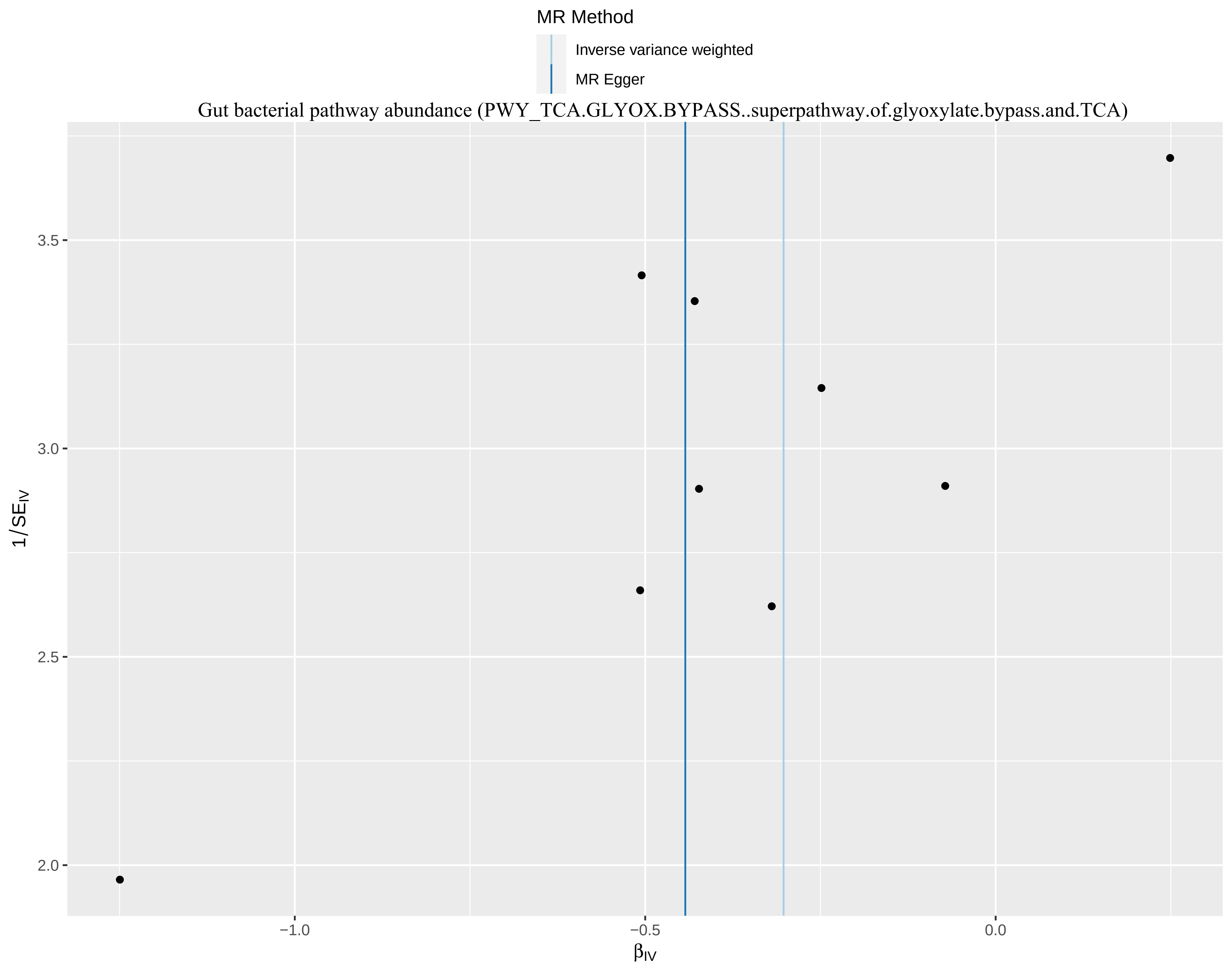

MR Method
Inverse variance weighted
MR Egger
Gut bacterial pathway abundance (PWY_TCA.GLYOX.BYPASS..superpathway.of.glyoxylate.bypass.and.TCA)
3.5
3.0
2.5
2.0
1/SE_IV
−1.0
−0.5
0.0
β_IV

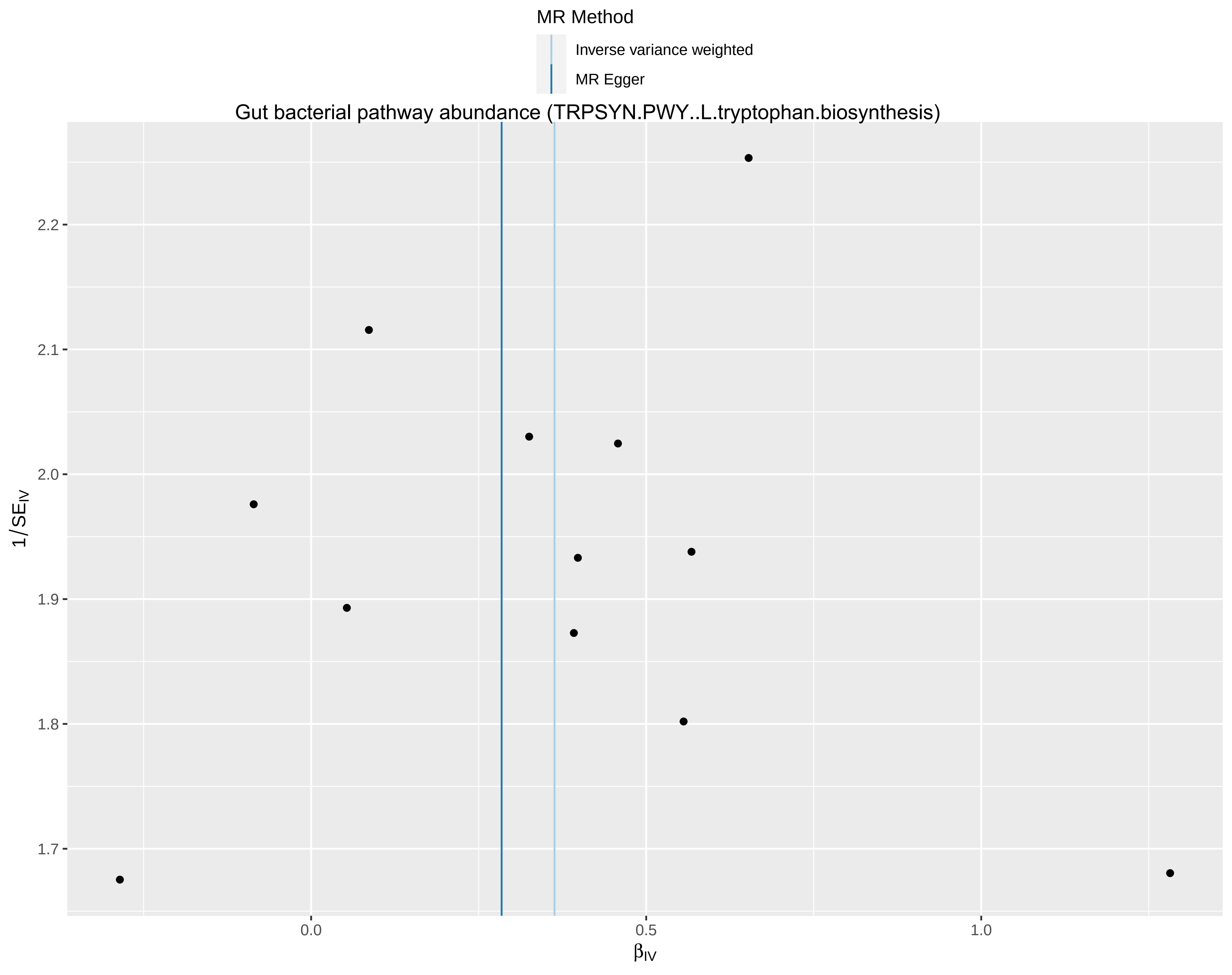
MR Method
Inverse variance weighted
MR Egger
Gut bacterial pathway abundance (TRPSYN.PWY..L.tryptophan.biosynthesis)
2.2
2.1
2.0
1.9
1.8
1.7
1/SE_IV
0.0
0.5
1.0
β_IV

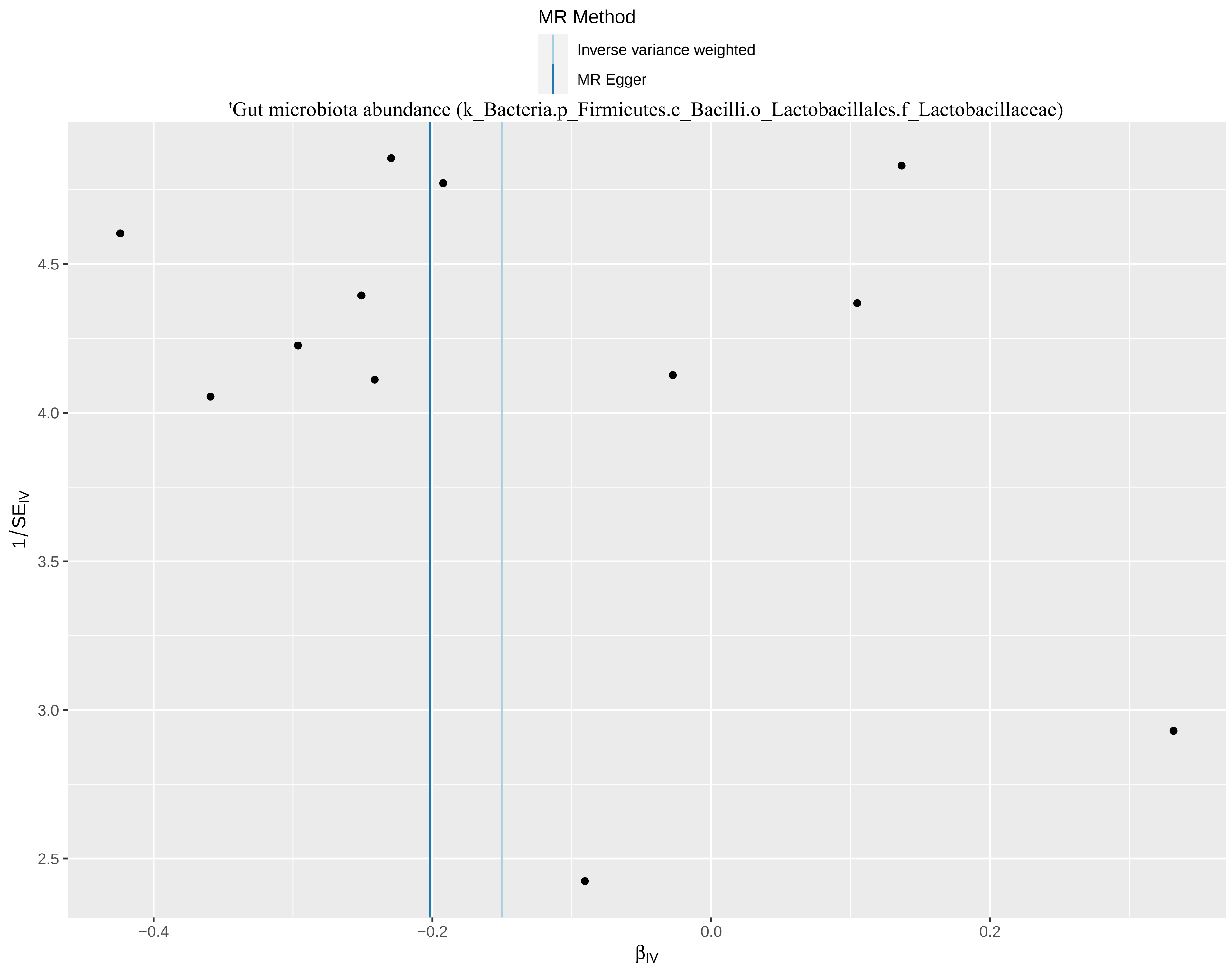
MR Method
Inverse variance weighted
MR Egger
'Gut microbiota abundance (k_Bacteria.p_Firmicutes.c_Bacilli.o_Lactobacillales.f_Lactobacillaceae)
4.5
4.0
3.5
3.0
2.5
1/SEIV
−0.4
−0.2
0.0
0.2
βIV

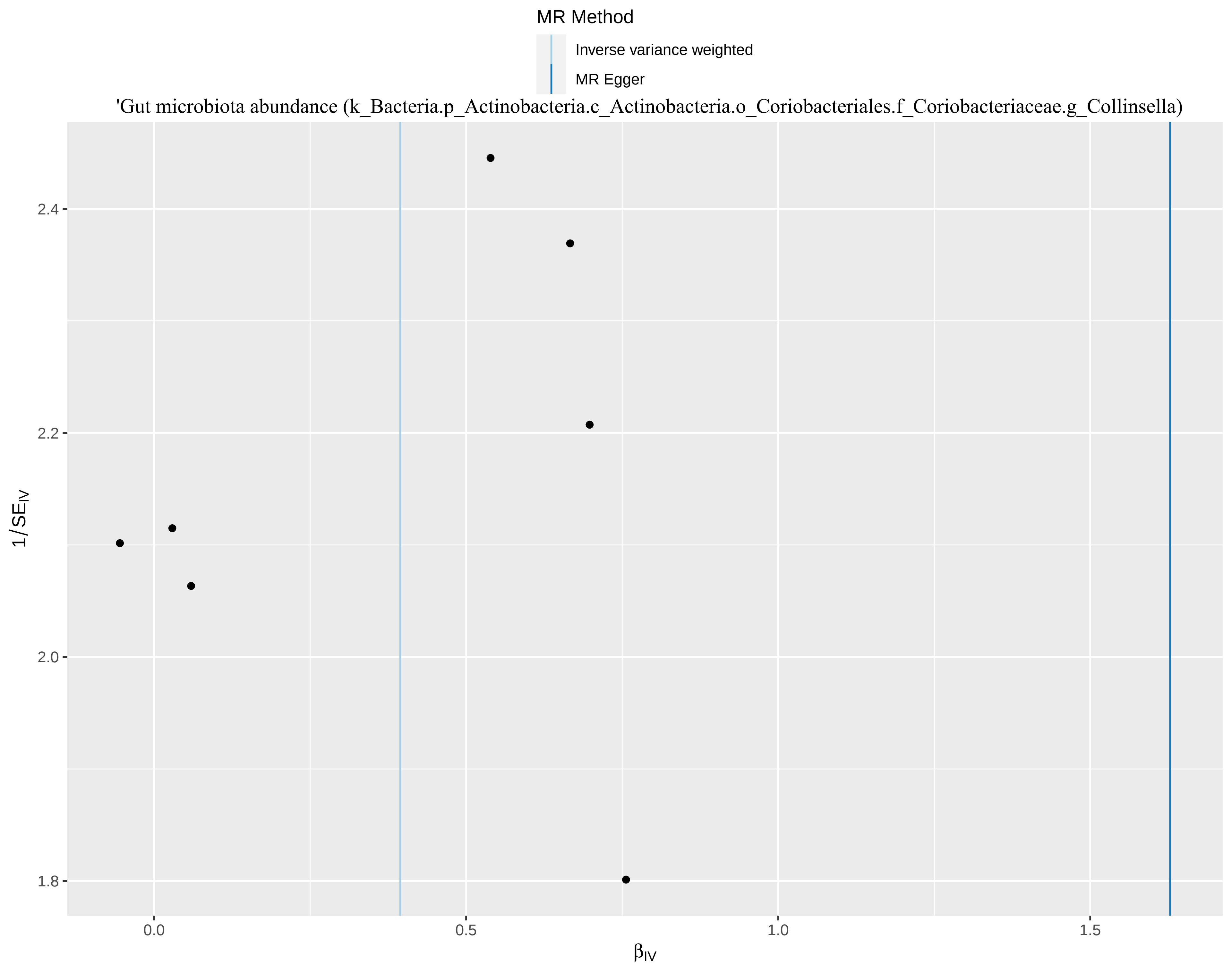
MR Method
Inverse variance weighted
MR Egger
'Gut microbiota abundance (k_Bacteria.p_Actinobacteria.c_Actinobacteria.o_Coriobacteriales.f_Coriobacteriaceae.g_Collinsella)
2.4
2.2
2.0
1.8
1/SE_IV
0.0
0.5
1.0
1.5
β_IV

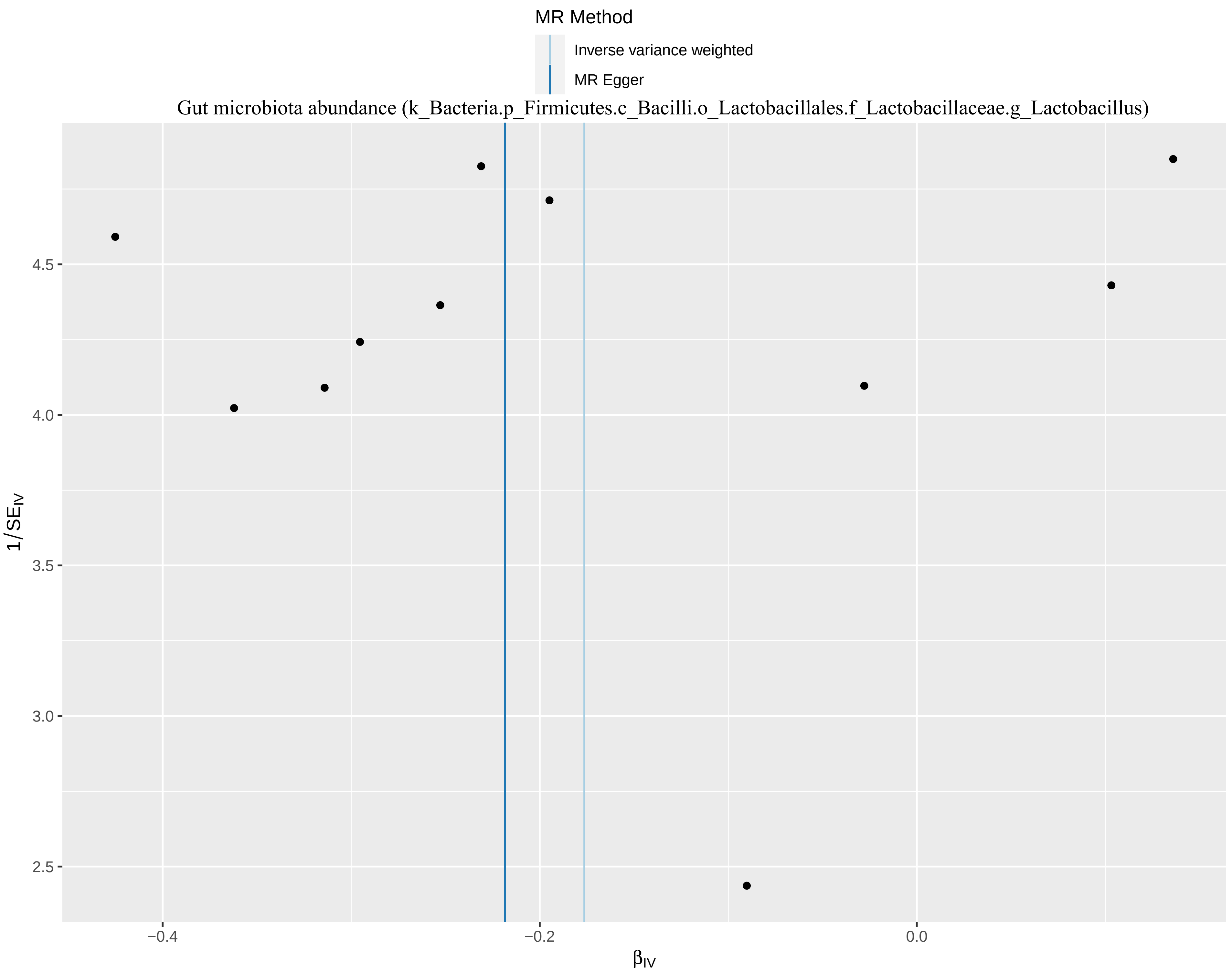
MR Method
Inverse variance weighted
MR Egger
Gut microbiota abundance (k_Bacteria.p_Firmicutes.c_Bacilli.o_Lactobacillales.f_Lactobacillaceae.g_Lactobacillus)
4.5
4.0
3.5
3.0
2.5
1/SE_IV
−0.4
−0.2
0.0
β_IV

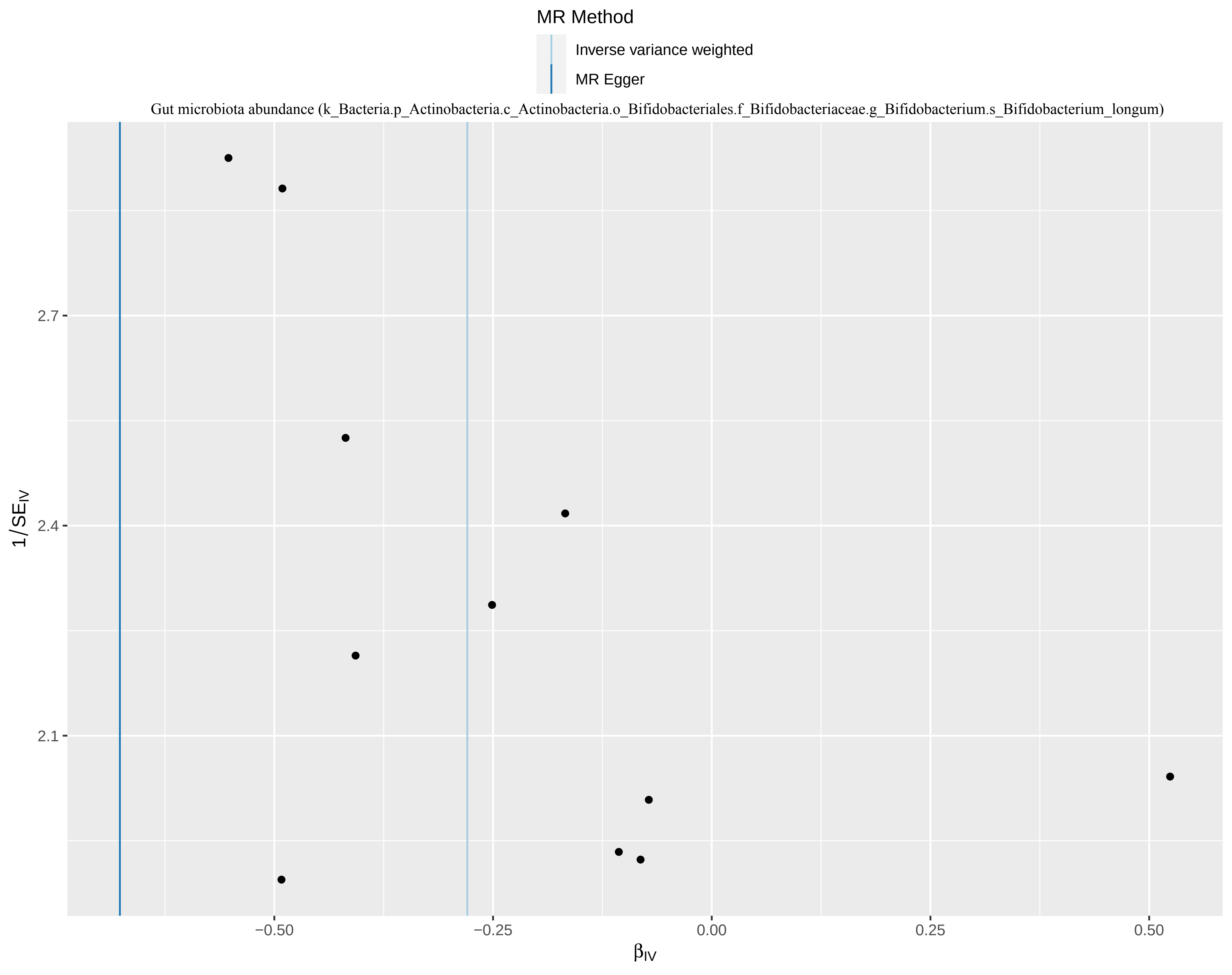
MR Method
Inverse variance weighted
MR Egger
Gut microbiota abundance (k_Bacteria.p_Actinobacteria.c_Actinobacteria.o_Bifidobacteriales.f_Bifidobacteriaceae.g_Bifidobacterium.s_Bifidobacterium_longum)
2.7
2.4
2.1
$1/SE_{IV}$
−0.50
−0.25
0.00
0.25
0.50
β_{IV}
