# Supplementary material for: Blood metabolites reflect the effect of gut microbiota on differentiated thyroid cancer: a Mendelian randomization analysis
Source: BMC Cancer. 2025 Feb 28;25:368. doi: 10.1186/s12885-025-13598-y (PMC11869591; doi:10.1186/s12885-025-13598-y)
Supplement: Supplementary file 3 — Supplementary Material 3. [file 12885_2025_13598_MOESM3_ESM.zip › Figure S6 Funnel plots for MR causal effects of microbiota abundance and metabolism on FTC.pdf]

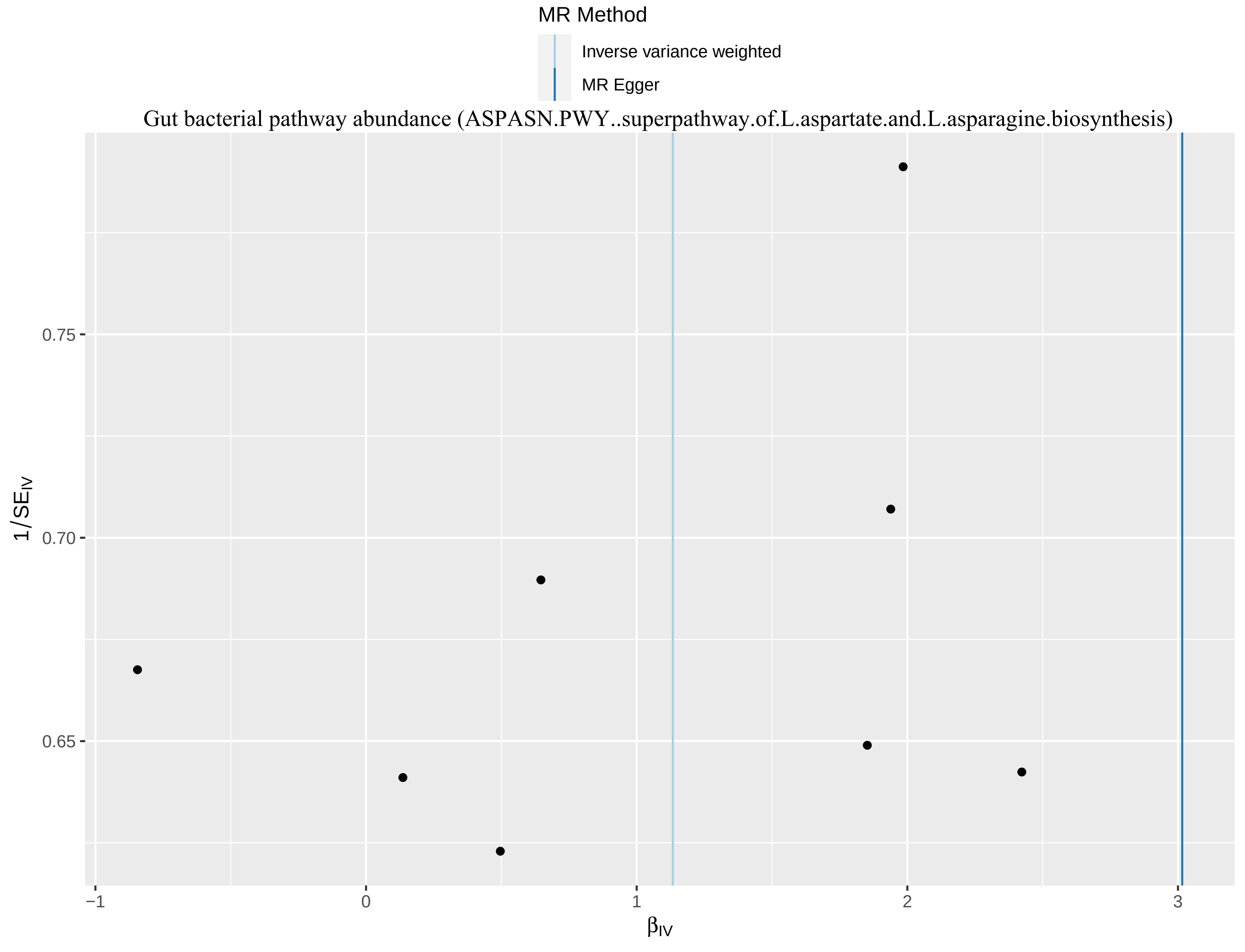

MR Method
Inverse variance weighted
MR Egger
Gut bacterial pathway abundance (ASPASN.PWY..superpathway.of.L.aspartate.and.L.asparagine.biosynthesis)
0.75
0.70
0.65
1/SE_IV
−1
0
1
2
3
β_IV

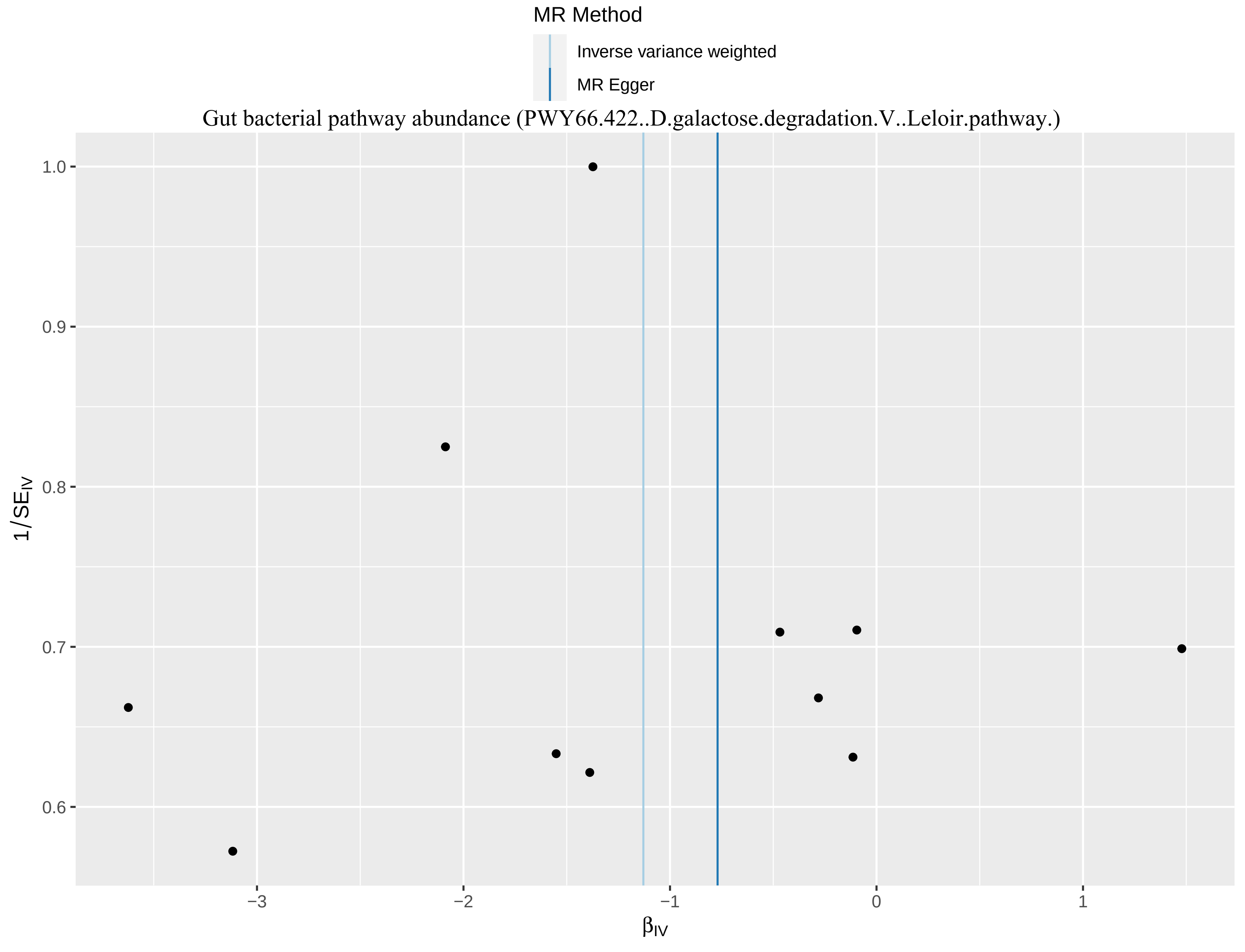

MR Method
Inverse variance weighted
MR Egger
Gut bacterial pathway abundance (PWY66.422..D.galactose.degradation.V..Leloir.pathway.)
1.0
0.9
0.8
0.7
0.6
1/SE_IV
−3
−2
−1
0
1
β_IV

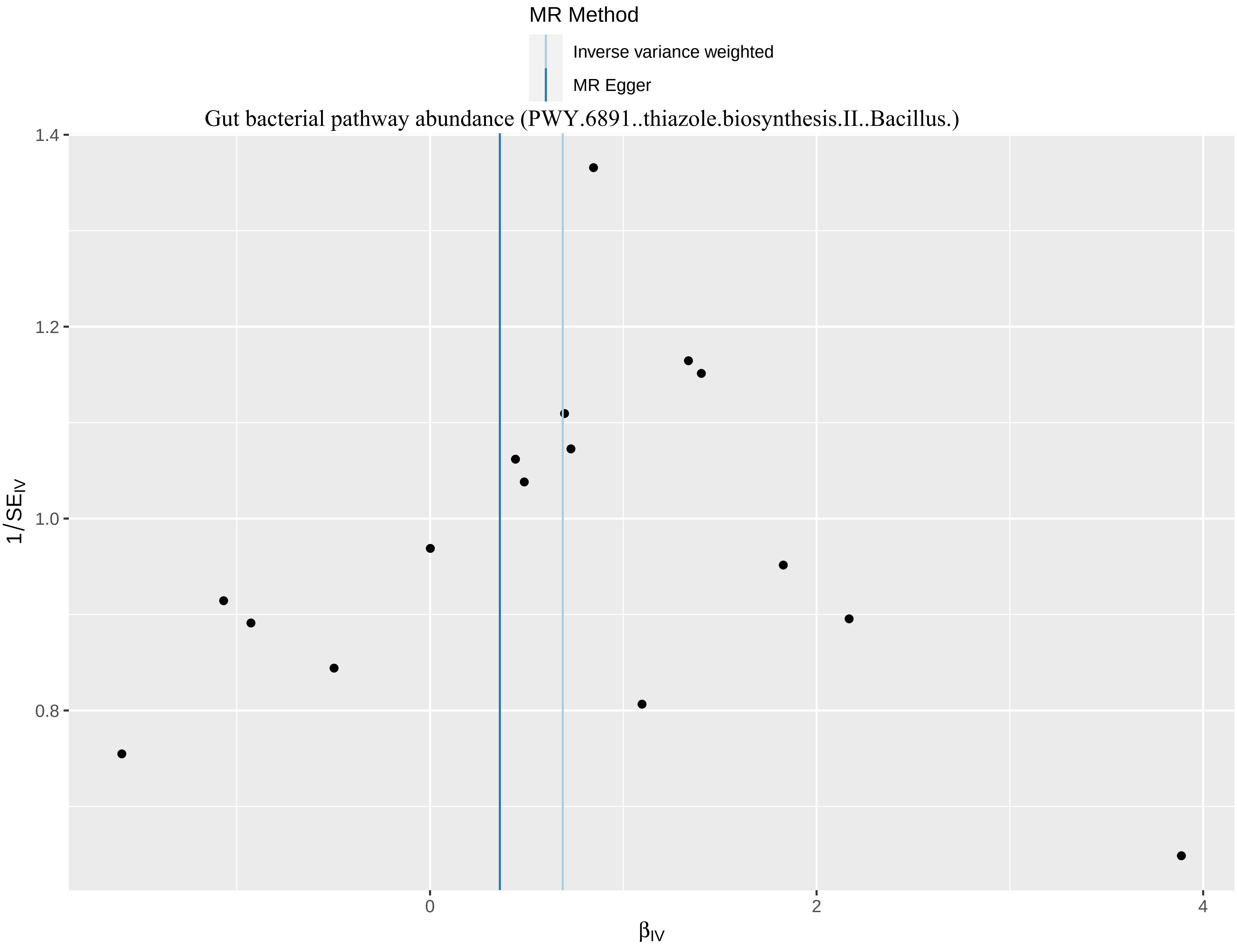

MR Method
Inverse variance weighted
MR Egger
Gut bacterial pathway abundance (PWY.6891..thiazole.biosynthesis.II..Bacillus.)
1.4
1.2
1.0
0.8
1/SE_IV
0
2
4
β_IV

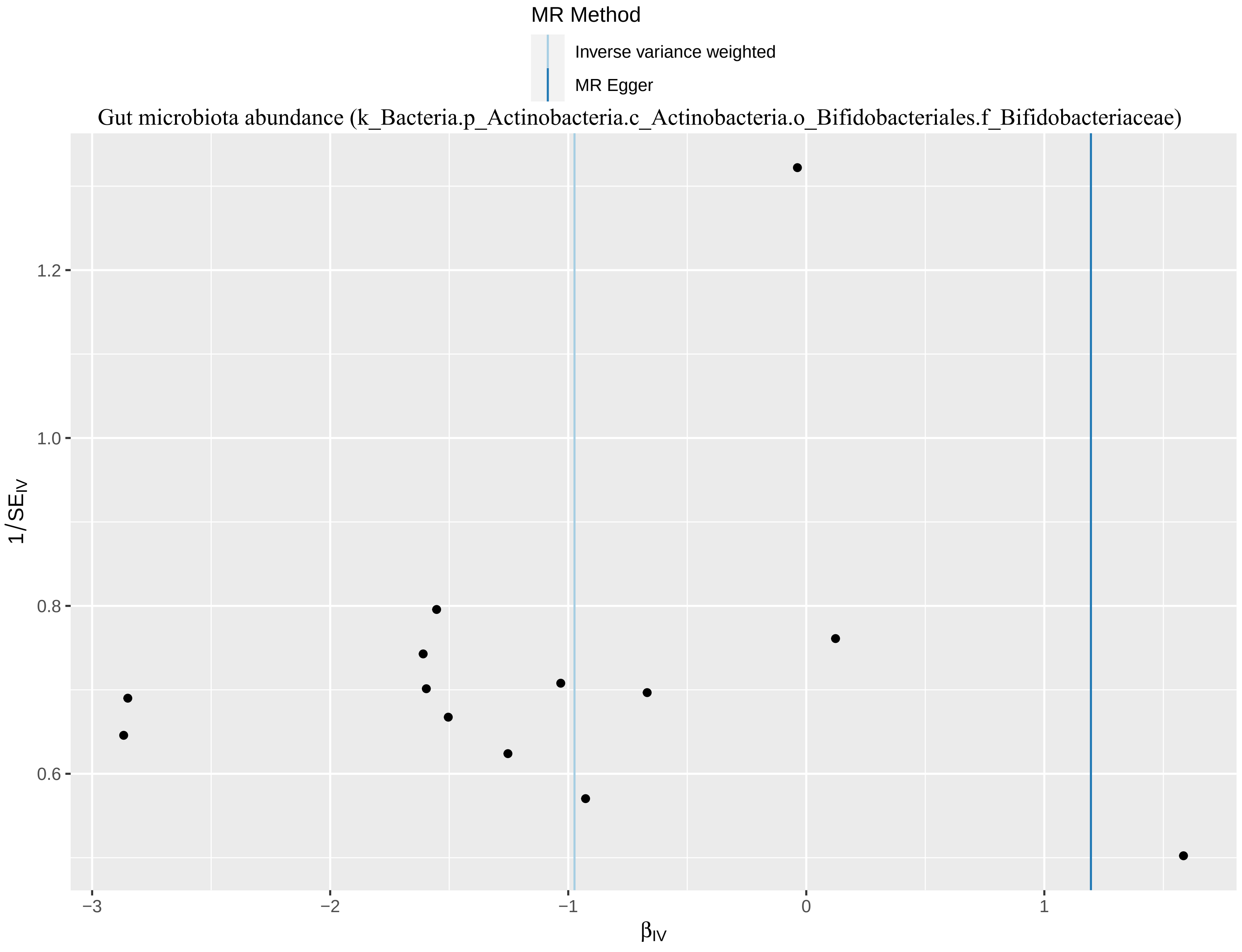

MR Method
Inverse variance weighted
MR Egger
Gut microbiota abundance (k_Bacteria.p_Actinobacteria.c_Actinobacteria.o_Bifidobacteriales.f_Bifidobacteriaceae)
1.2
1.0
0.8
0.6
1/SE_IV
−3
−2
−1
0
1
β_IV

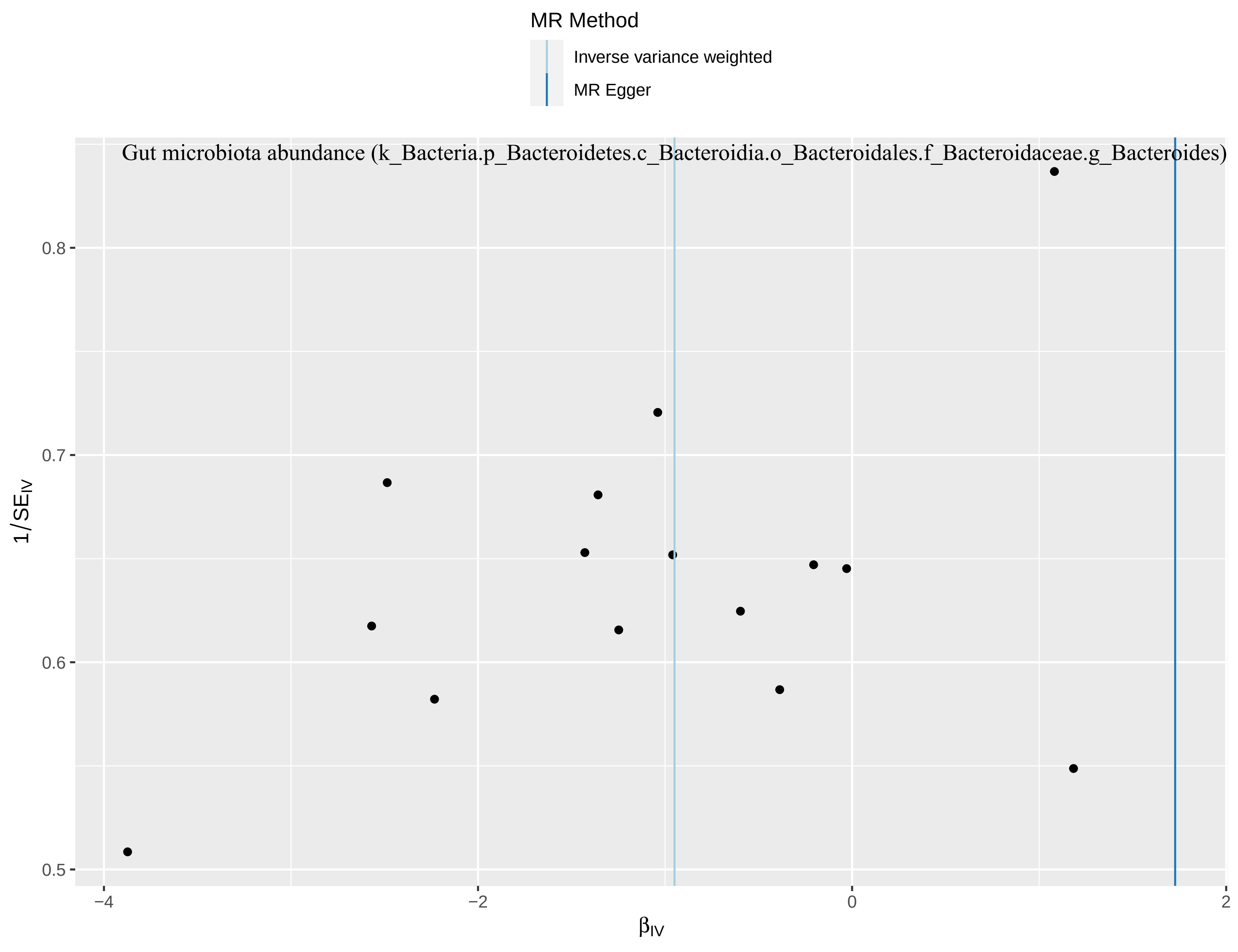

MR Method
Inverse variance weighted
MR Egger
Gut microbiota abundance (k_Bacteria.p_Bacteroidetes.c_Bacteroidia.o_Bacteroidales.f_Bacteroidaceae.g_Bacteroides)
0.8
0.7
0.6
0.5
1/SE_IV
−4
−2
0
2
β_IV

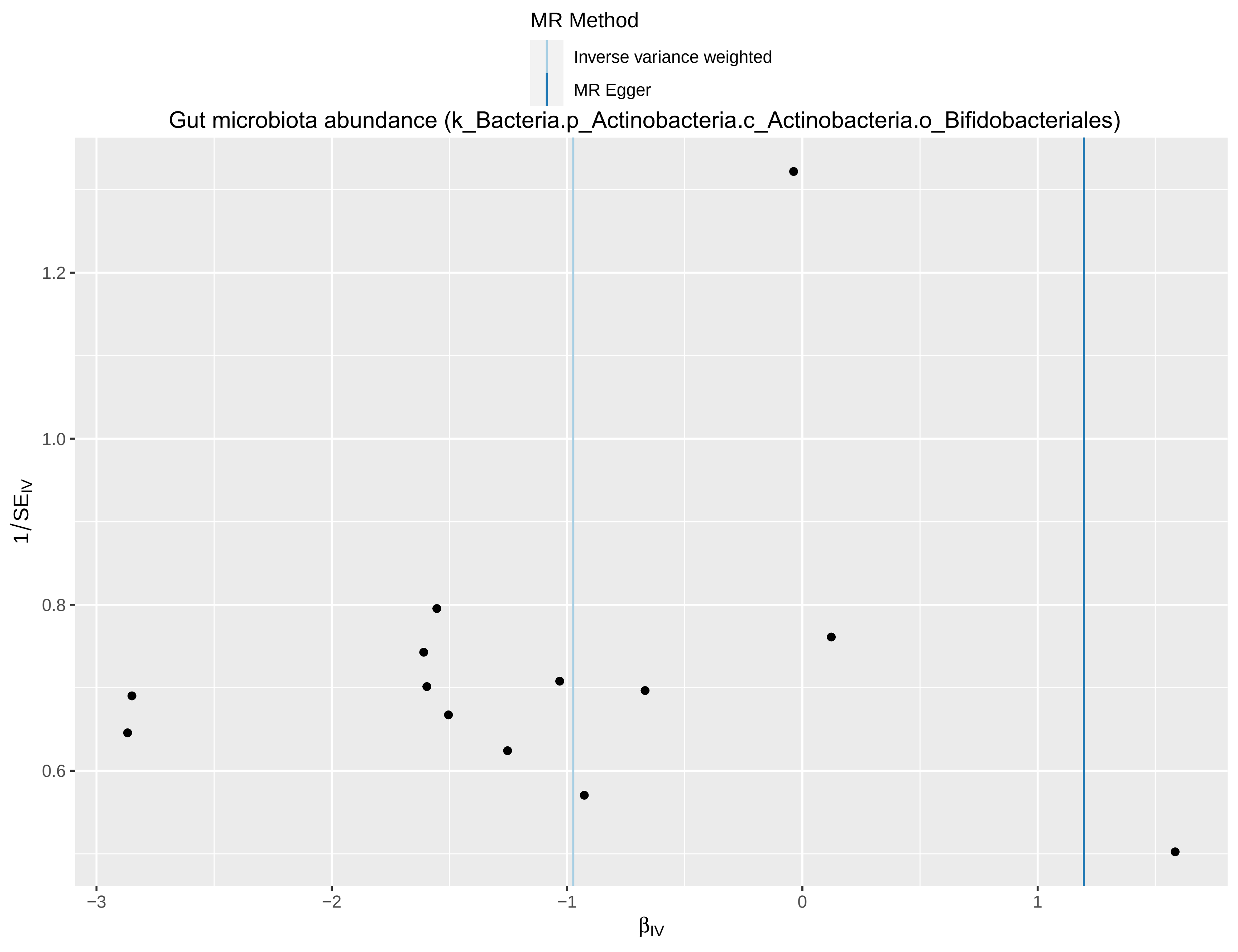

MR Method
Inverse variance weighted
MR Egger
Gut microbiota abundance (k_Bacteria.p_Actinobacteria.c_Actinobacteria.o_Bifidobacteriales)
1.2
1.0
0.8
0.6
1/SE_IV
−3
−2
−1
0
1
β_IV
