# Supplementary material for: Blood metabolites reflect the effect of gut microbiota on differentiated thyroid cancer: a Mendelian randomization analysis
Source: BMC Cancer. 2025 Feb 28;25:368. doi: 10.1186/s12885-025-13598-y (PMC11869591; doi:10.1186/s12885-025-13598-y)
Supplement: Supplementary file 3 — Supplementary Material 3. [file 12885_2025_13598_MOESM3_ESM.zip › Figure S7 Funnel plots for MR causal effects of blood metabolites on PTC.pdf]

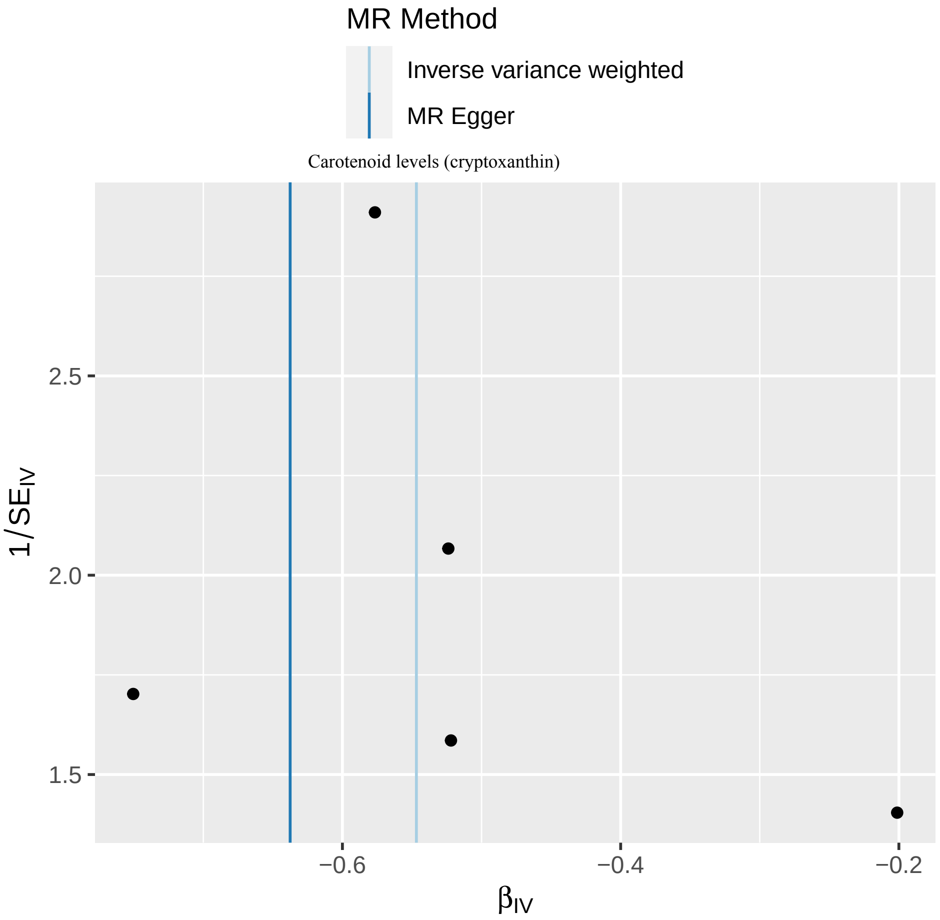
MR Method
Inverse variance weighted
MR Egger
Carotenoid levels (cryptoxanthin)
2.5
2.0
1.5
−0.6
−0.4
−0.2
β_{IV}
$1/SE_{IV}$

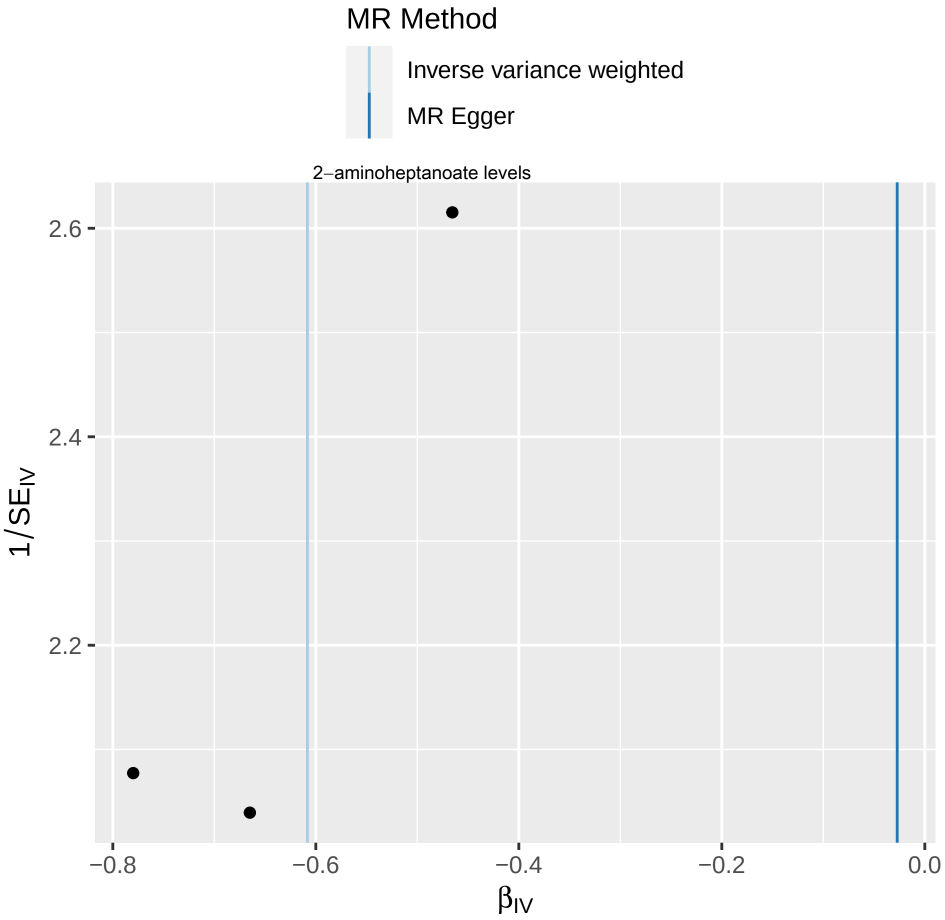

MR Method
Inverse variance weighted
MR Egger
2−aminoheptanoate levels
2.6
2.4
2.2
1/SE$_{IV}$
−0.8
−0.6
−0.4
−0.2
0.0
β$_{IV}$

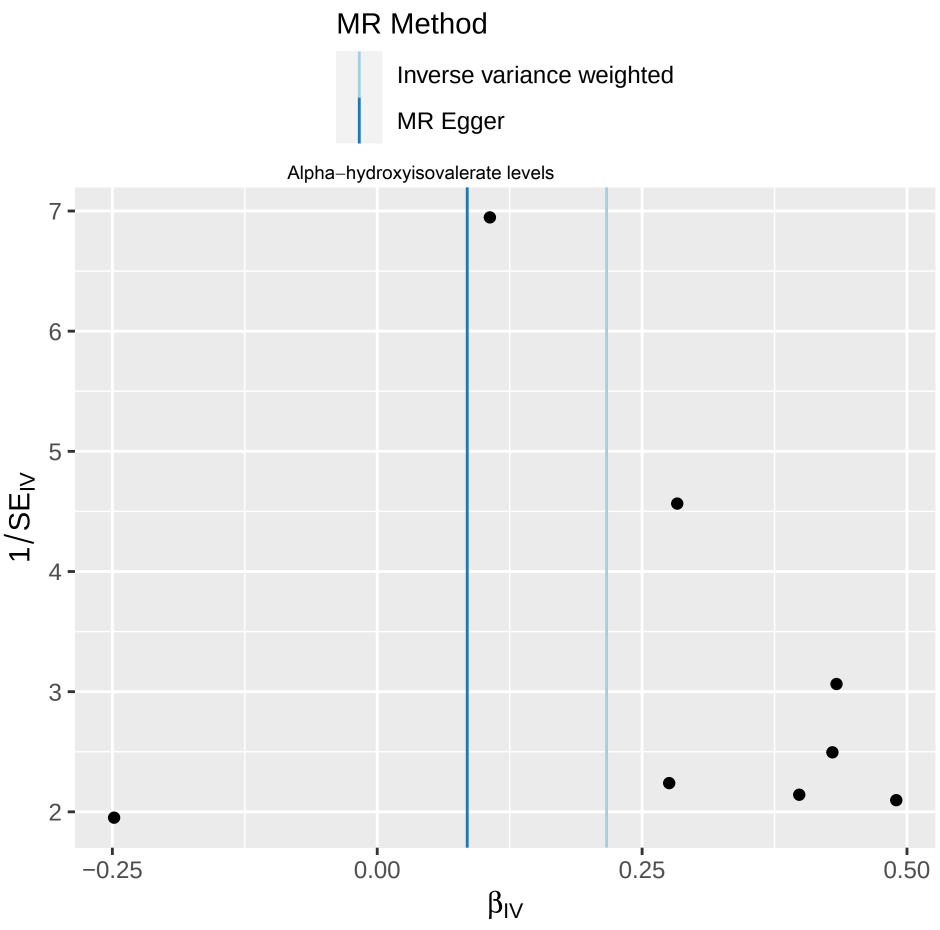
MR Method
Inverse variance weighted
MR Egger
Alpha−hydroxyisovalerate levels
7
6
5
4
3
2
$1/SE_{IV}$
−0.25
0.00
0.25
0.50
β_{IV}

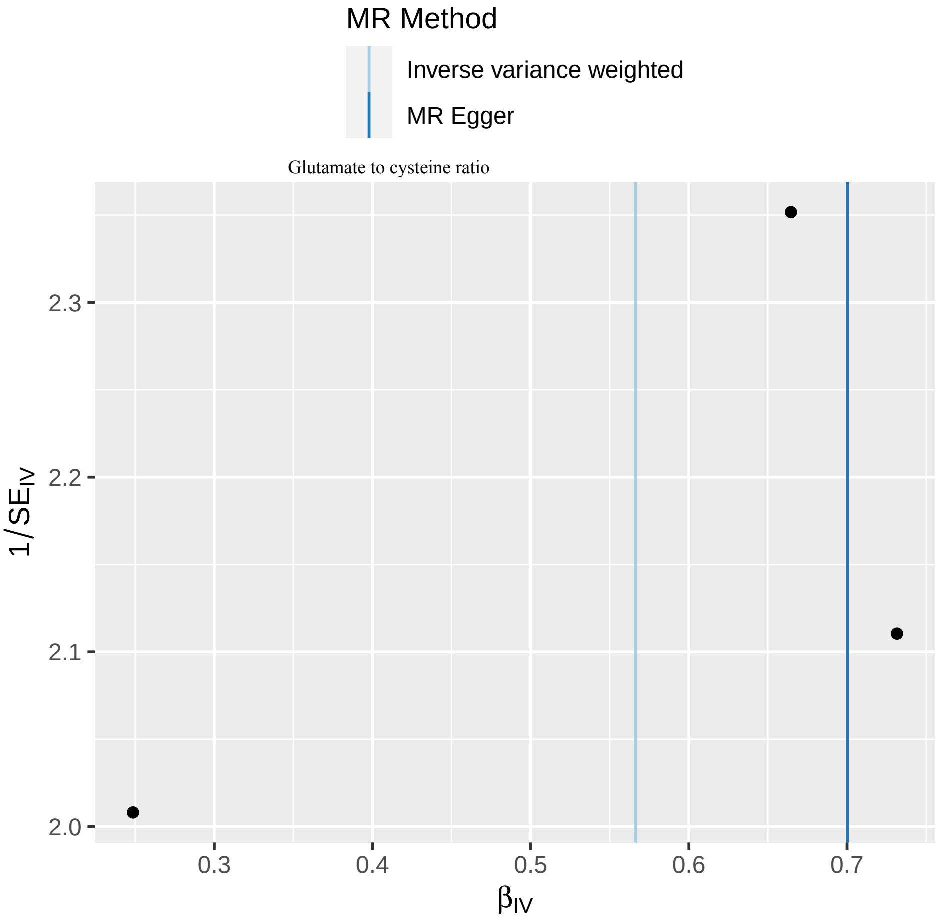
MR Method
Inverse variance weighted
MR Egger
Glutamate to cysteine ratio
2.3
2.2
2.1
2.0
$1/SE_{IV}$
0.3
0.4
0.5
0.6
0.7
β_{IV}

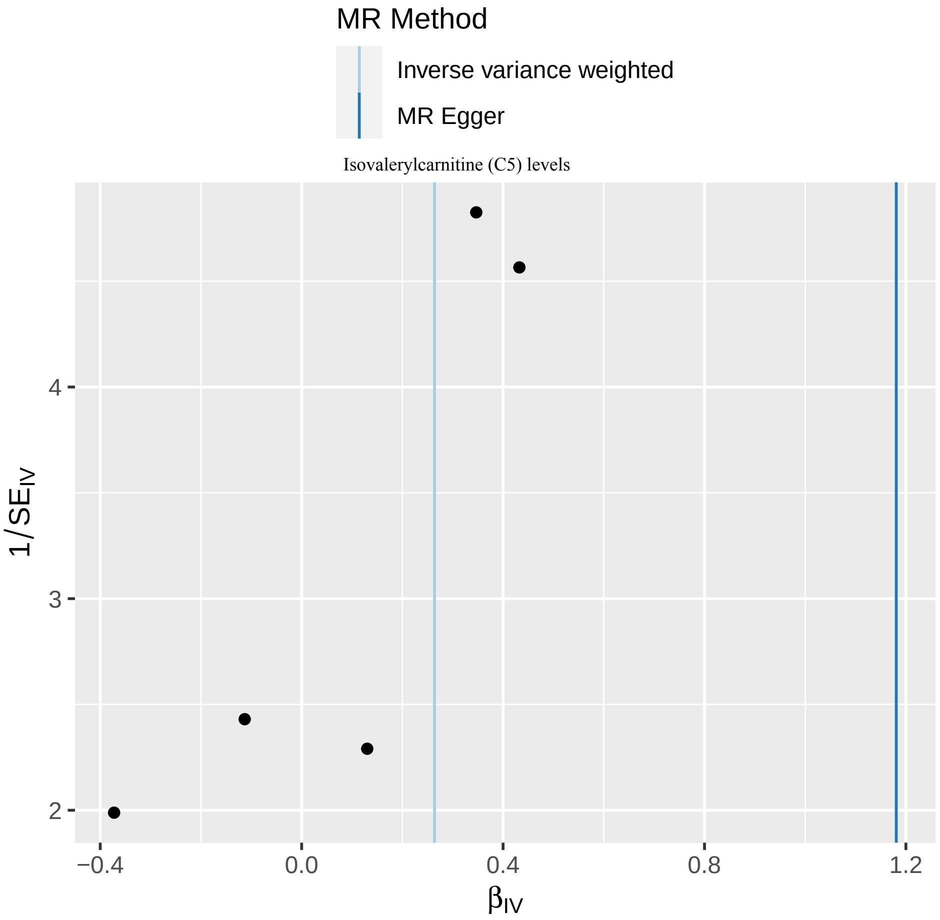

MR Method
Inverse variance weighted
MR Egger
Isovalerylcarnitine (C5) levels
4
3
2
1/SE_IV
−0.4
0.0
0.4
0.8
1.2
β_IV

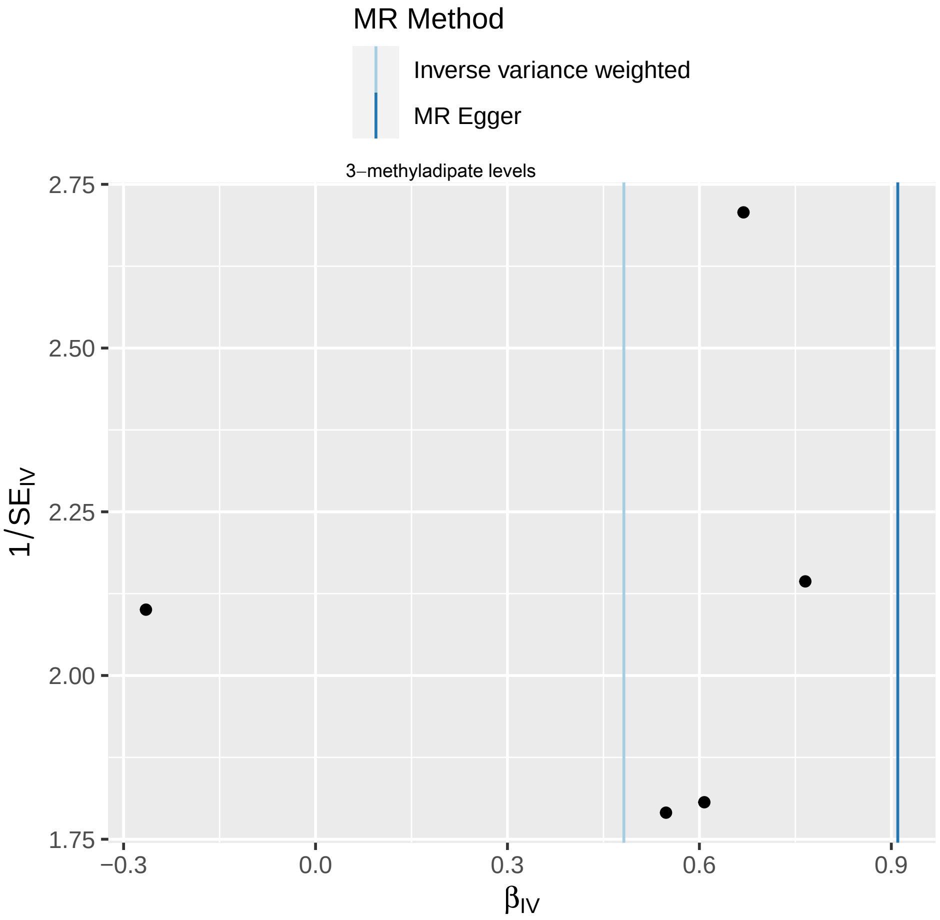
MR Method
Inverse variance weighted
MR Egger
3−methyladipate levels
2.75
2.50
2.25
2.00
1.75
−0.3
0.0
0.3
0.6
0.9
$1/SE_{IV}$
β_{IV}

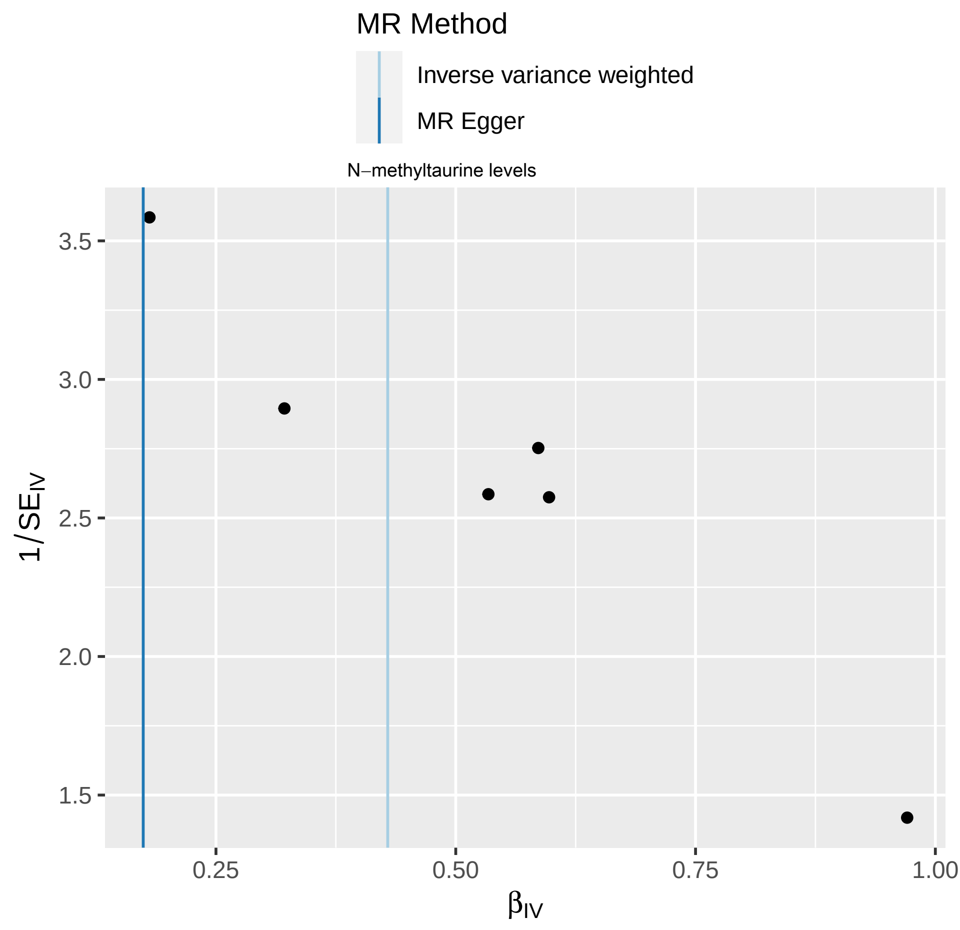
MR Method
Inverse variance weighted
MR Egger
N−methyltaurine levels
3.5
3.0
2.5
2.0
1.5
1/SE_IV
0.25
0.50
0.75
1.00
β_IV

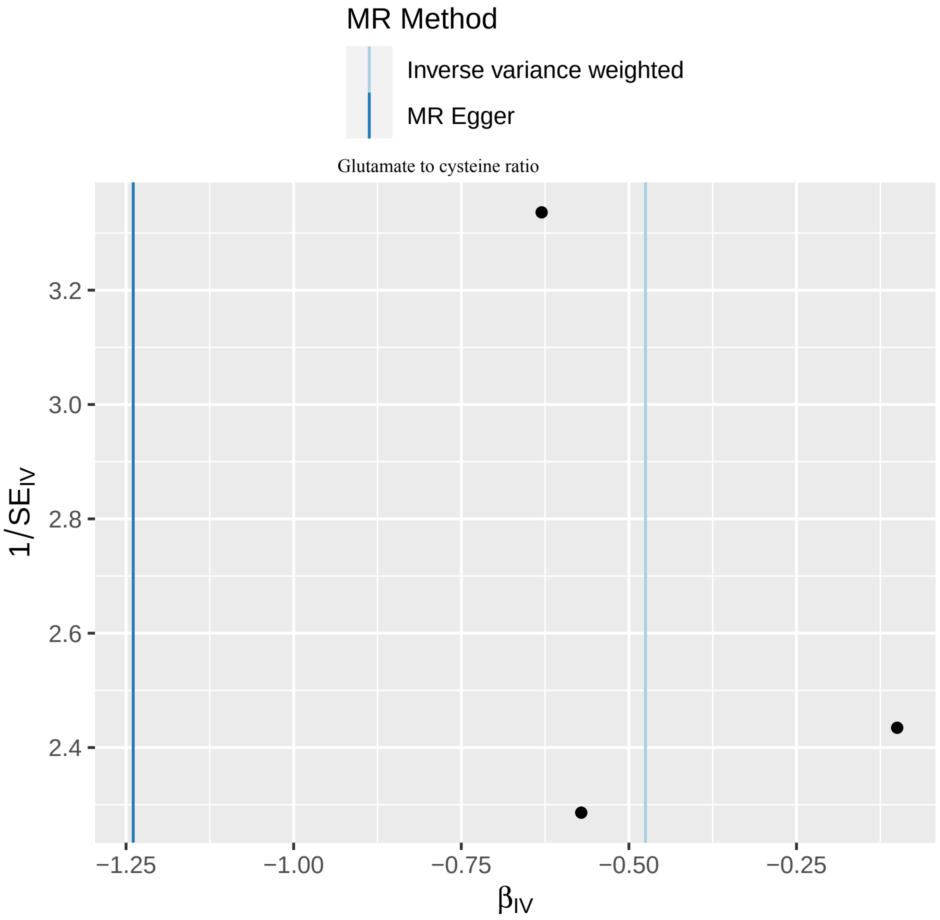
MR Method
Inverse variance weighted
MR Egger
Glutamate to cysteine ratio
3.2
3.0
2.8
2.6
2.4
1/SE_IV
−1.25
−1.00
−0.75
−0.50
−0.25
β_IV

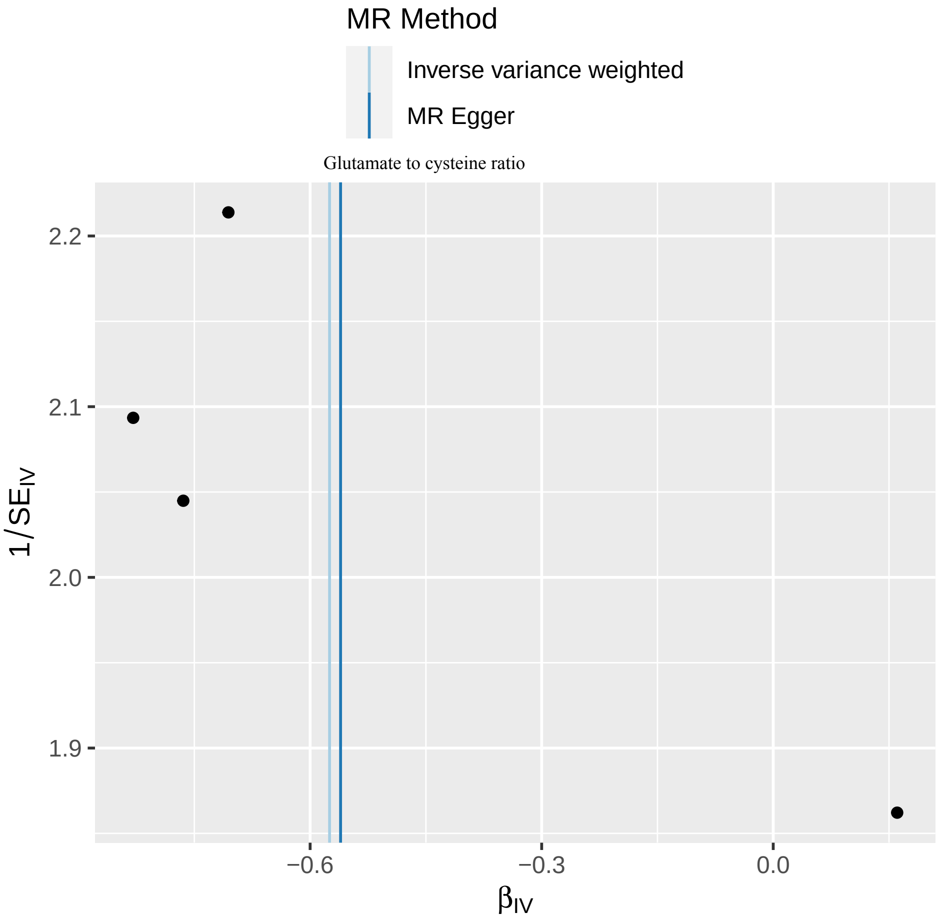
MR Method
Inverse variance weighted
MR Egger
Glutamate to cysteine ratio
2.2
2.1
2.0
1.9
1/SE$_{IV}$
−0.6
−0.3
0.0
β$_{IV}$

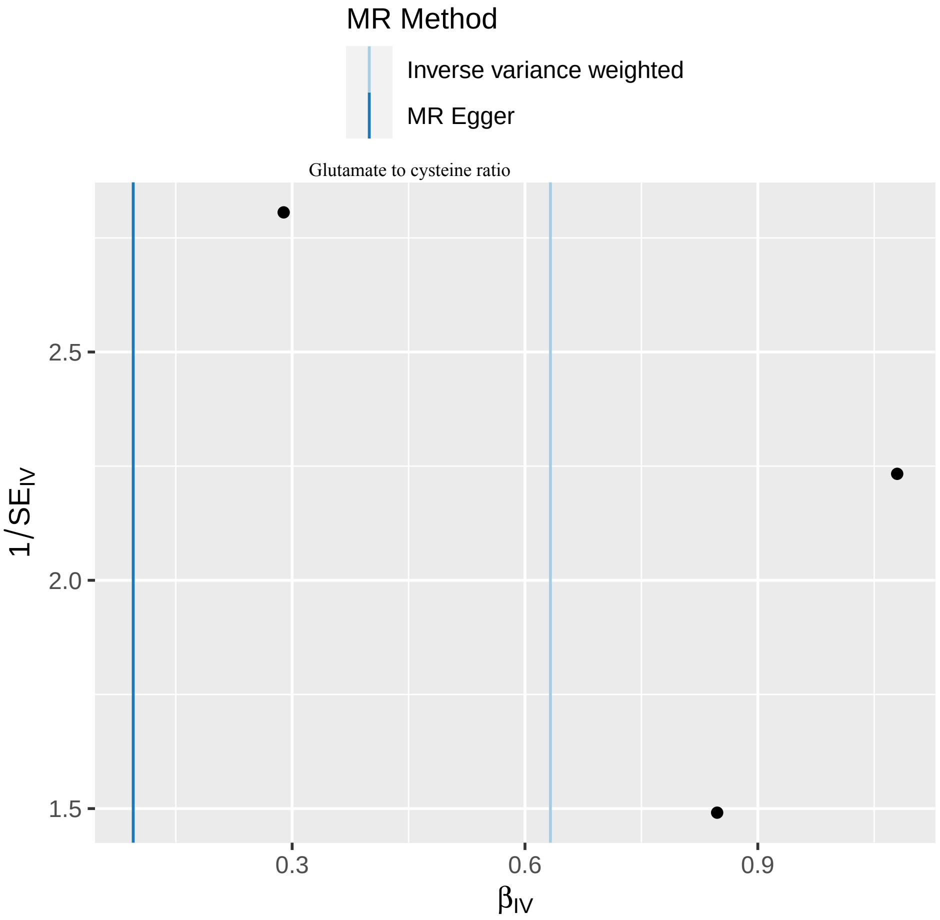
MR Method
Inverse variance weighted
MR Egger
Glutamate to cysteine ratio
2.5
2.0
1.5
0.3
0.6
0.9
1/SE_IV
β_IV

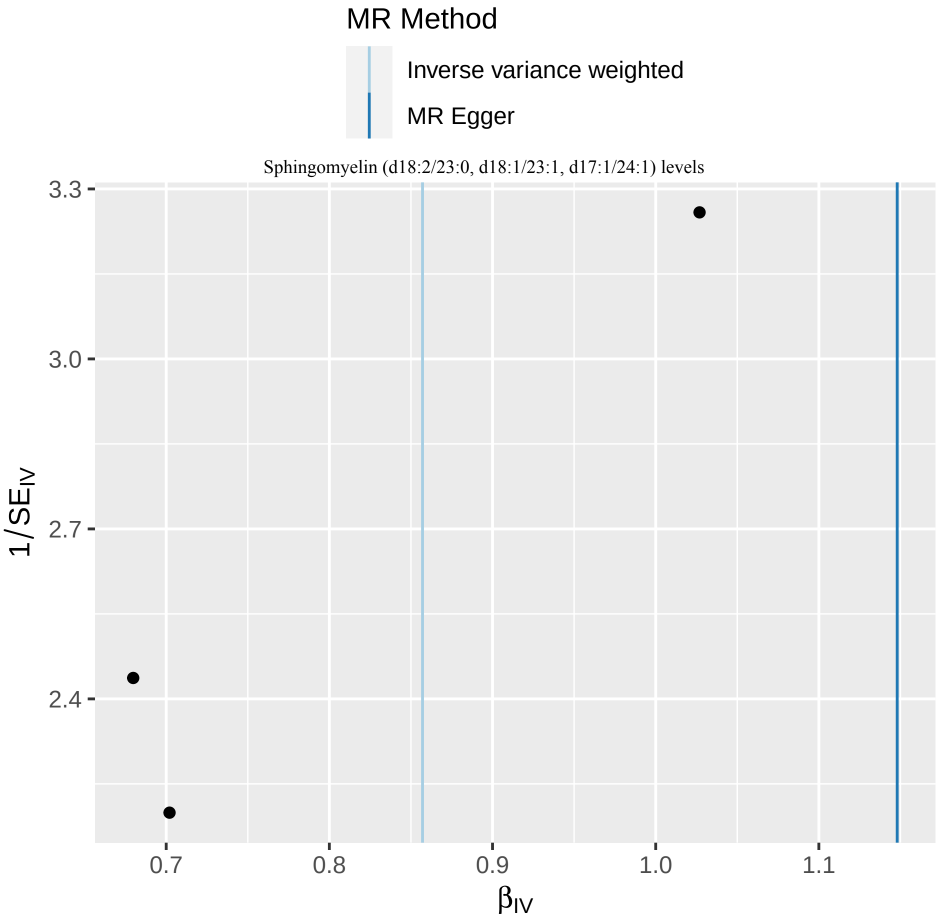

MR Method
Inverse variance weighted
MR Egger
Sphingomyelin (d18:2/23:0, d18:1/23:1, d17:1/24:1) levels
3.3
3.0
2.7
2.4
$1/SE_{IV}$
0.7
0.8
0.9
1.0
1.1
β_{IV}

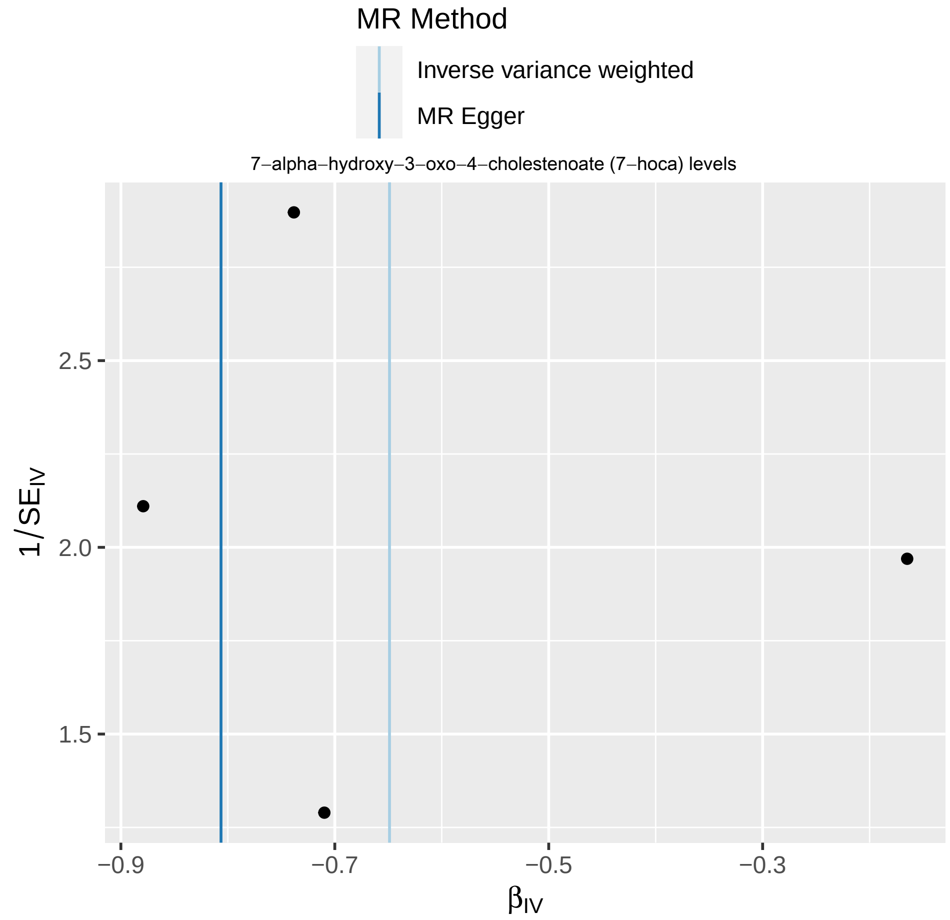

MR Method
Inverse variance weighted
MR Egger
7−alpha−hydroxy−3−oxo−4−cholestenoate (7−hoca) levels
2.5
2.0
1.5
1/SE_IV
−0.9
−0.7
−0.5
−0.3
β_IV

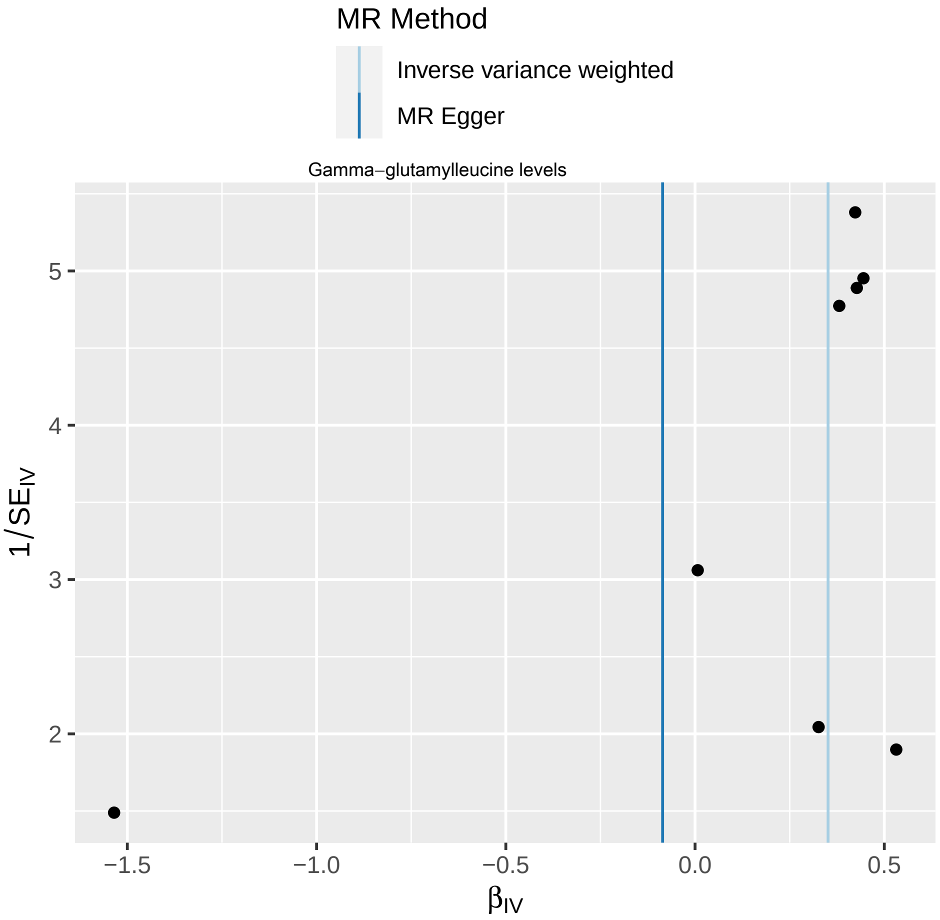
MR Method
Inverse variance weighted
MR Egger
Gamma−glutamylleucine levels
5
4
3
2
1/SE_IV
−1.5
−1.0
−0.5
0.0
0.5
β_IV

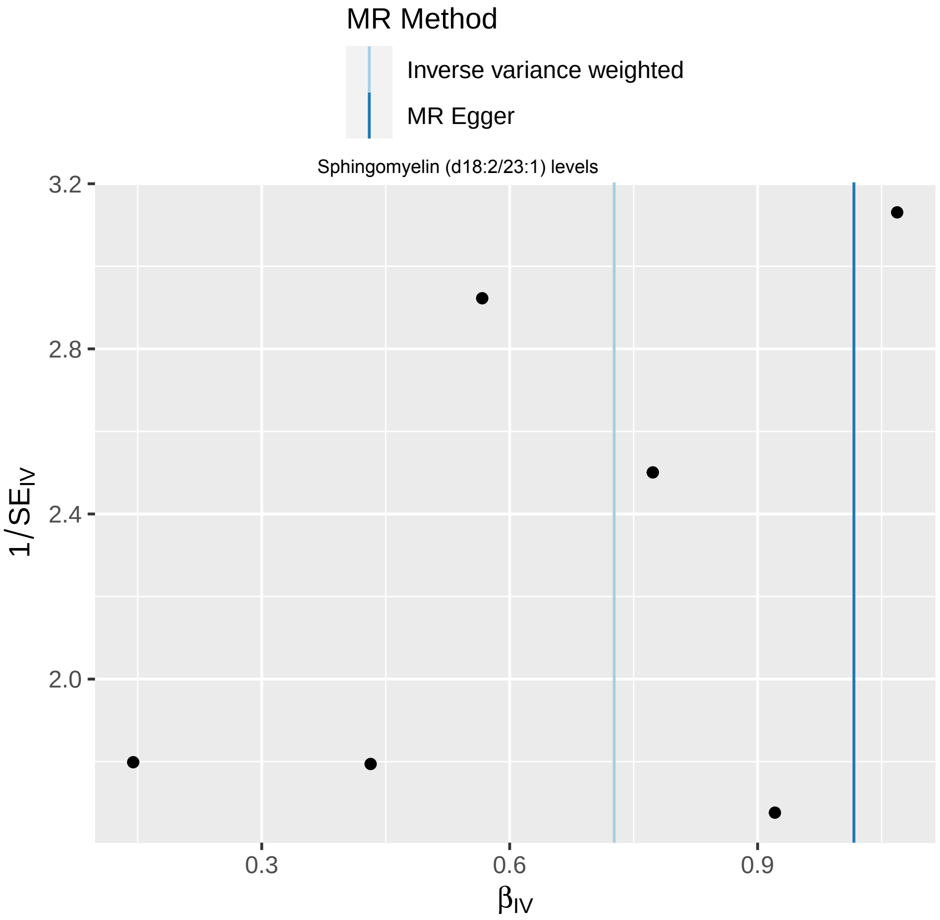

MR Method
Inverse variance weighted
MR Egger
Sphingomyelin (d18:2/23:1) levels
3.2
2.8
2.4
2.0
1/SE_IV
0.3
0.6
0.9
β_IV

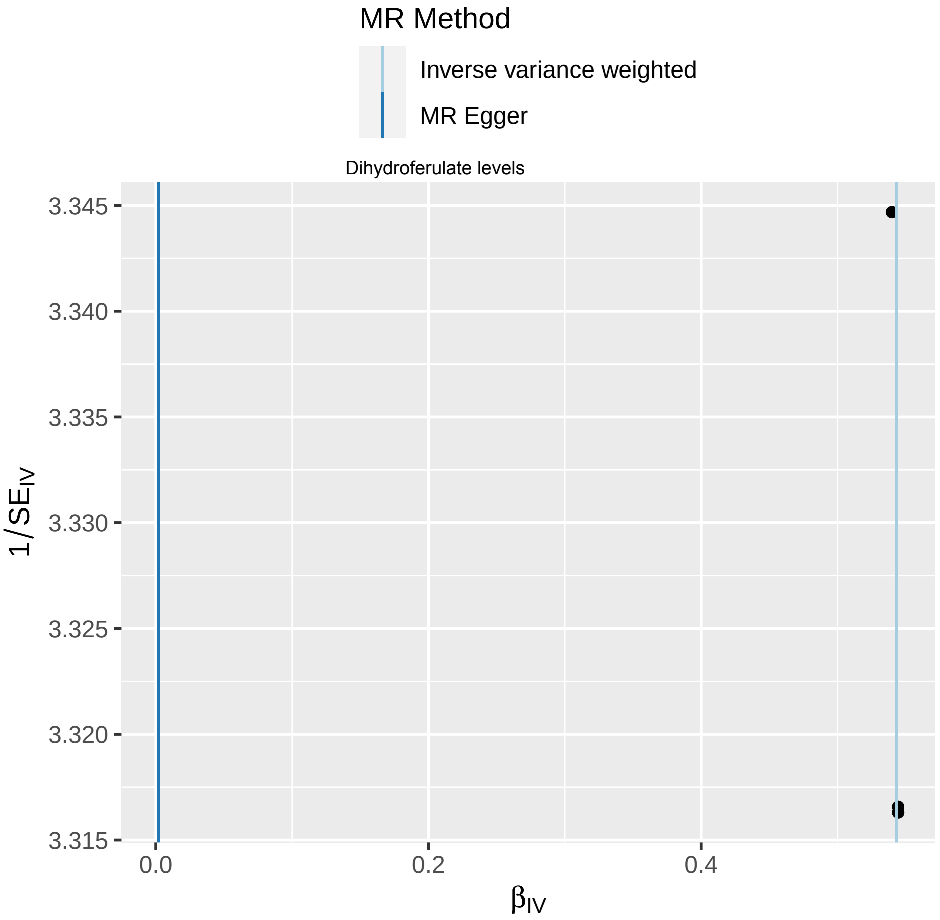
MR Method
Inverse variance weighted
MR Egger
Dihydroferulate levels
3.345
3.340
3.335
3.330
3.325
3.320
3.315
1/SE_IV
0.0
0.2
0.4
β_IV

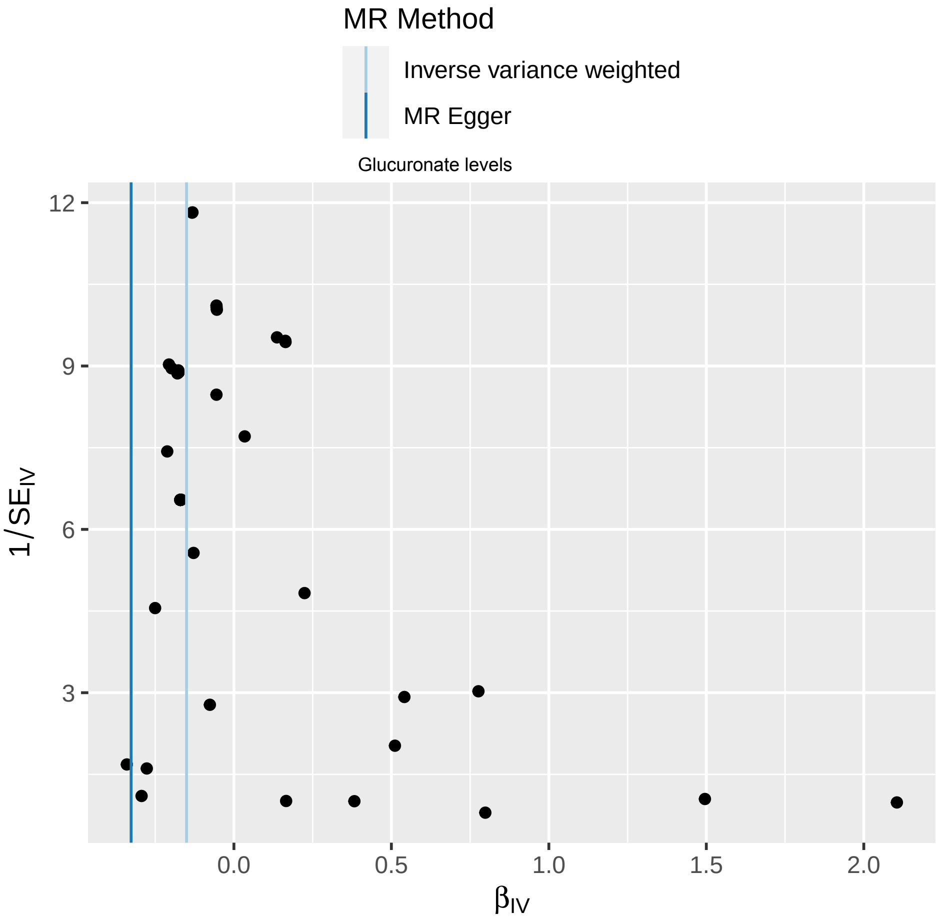

MR Method
Inverse variance weighted
MR Egger
Glucuronate levels
12
9
6
3
0.0
0.5
1.0
1.5
2.0
$1/SE_{IV}$
β_{IV}

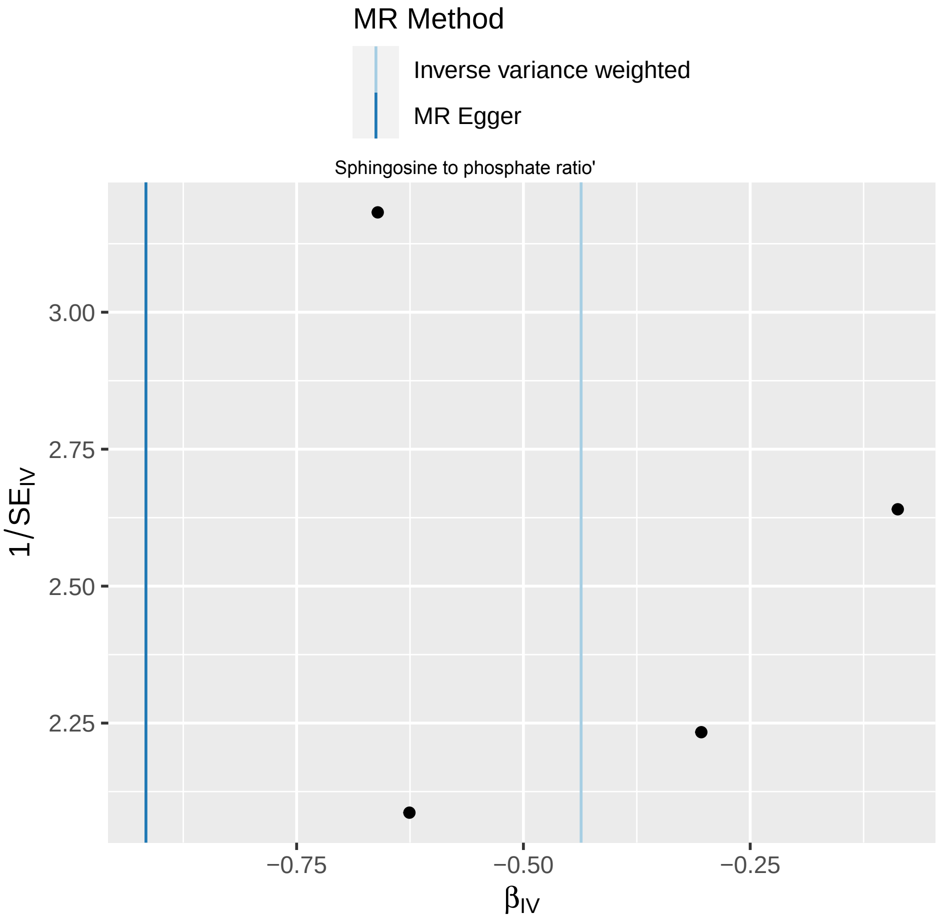
MR Method
Inverse variance weighted
MR Egger
Sphingosine to phosphate ratio'
3.00
2.75
2.50
2.25
1/SE_IV
−0.75
−0.50
−0.25
β_IV

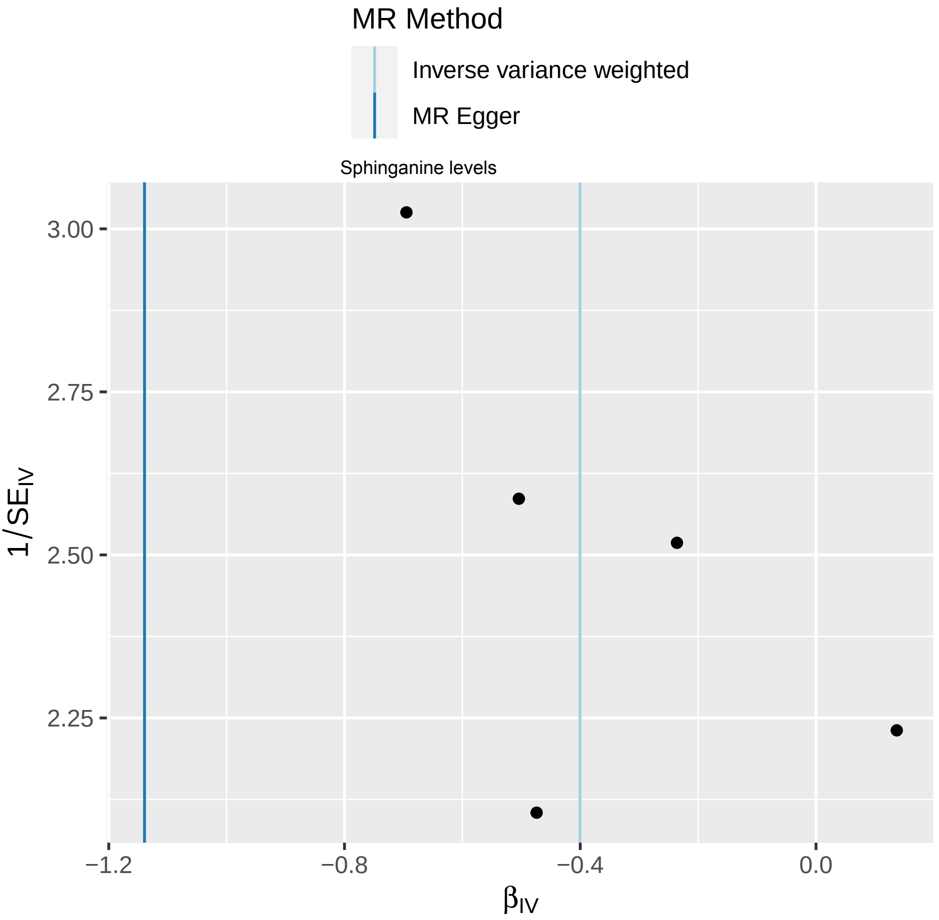
MR Method
Inverse variance weighted
MR Egger
Sphinganine levels
3.00
2.75
2.50
2.25
$1/SE_{IV}$
−1.2
−0.8
−0.4
0.0
β_{IV}

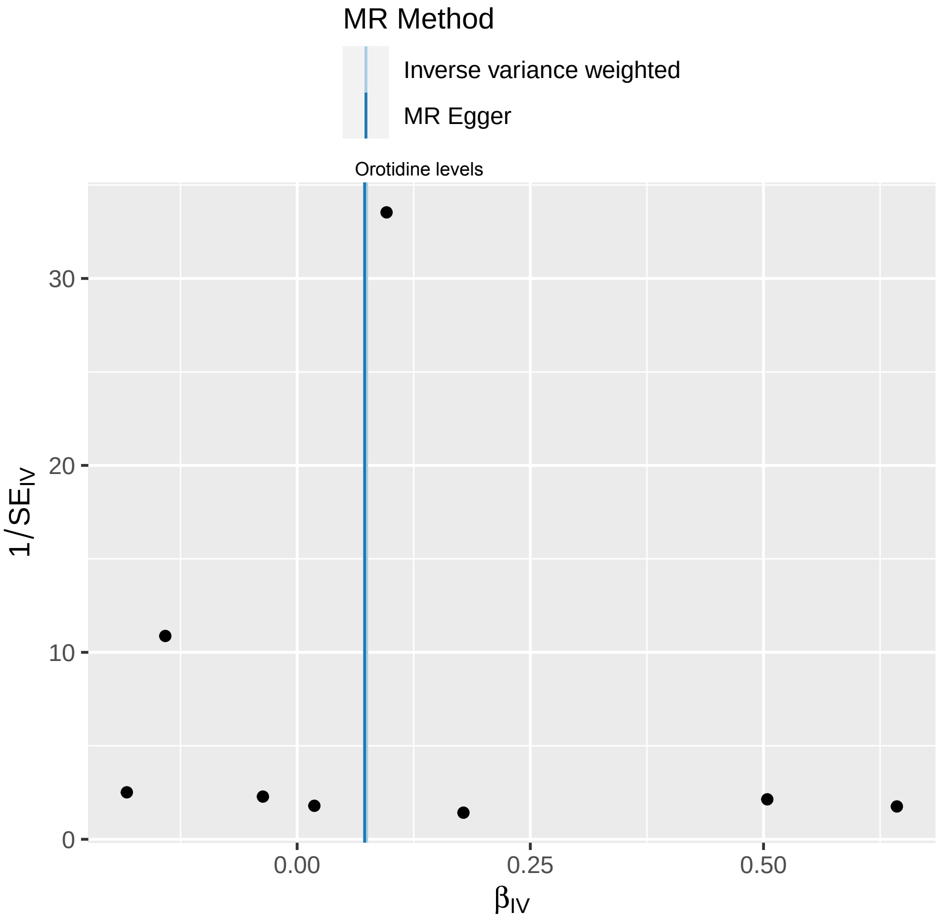
MR Method
Inverse variance weighted
MR Egger
Orotidine levels
1/SE_IV
30
20
10
0
0.00
0.25
0.50
β_IV
