# Supplementary material for: Blood metabolites reflect the effect of gut microbiota on differentiated thyroid cancer: a Mendelian randomization analysis
Source: BMC Cancer. 2025 Feb 28;25:368. doi: 10.1186/s12885-025-13598-y (PMC11869591; doi:10.1186/s12885-025-13598-y)
Supplement: Supplementary file 3 — Supplementary Material 3. [file 12885_2025_13598_MOESM3_ESM.zip › Figure S8 Funnel plots for MR causal effects of blood metabolites on FTC.pdf]

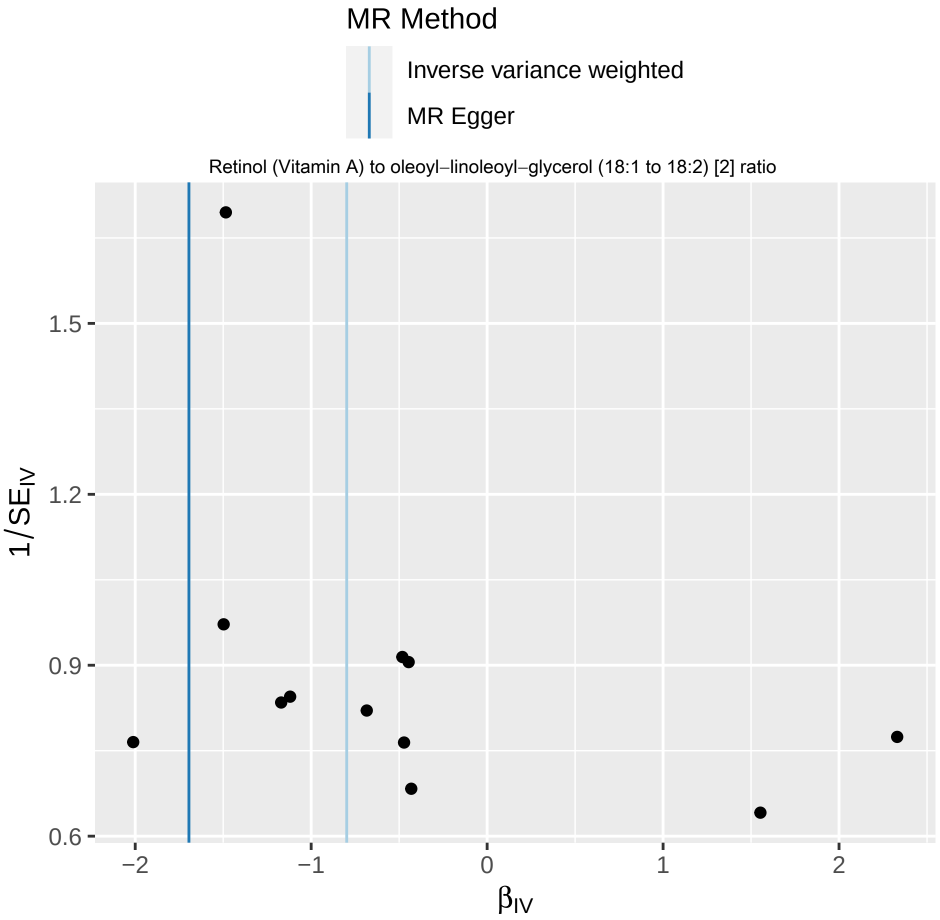

MR Method
Inverse variance weighted
MR Egger
Retinol (Vitamin A) to oleoyl−linoleoyl−glycerol (18:1 to 18:2) [2] ratio
1.5
1.2
0.9
0.6
$1/SE_{IV}$
−2
−1
0
1
2
β_{IV}

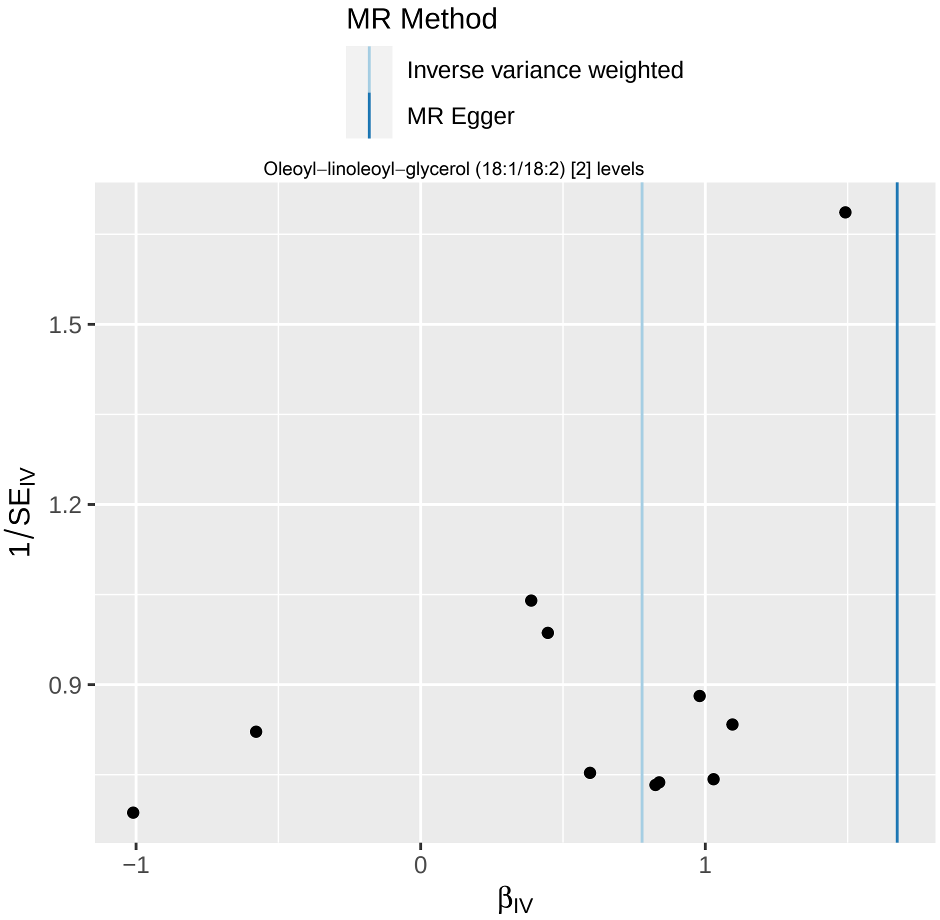
MR Method
Inverse variance weighted
MR Egger
Oleoyl−linoleoyl−glycerol (18:1/18:2) [2] levels
1.5
1.2
0.9
1/SE$_{IV}$
−1
0
1
β$_{IV}$

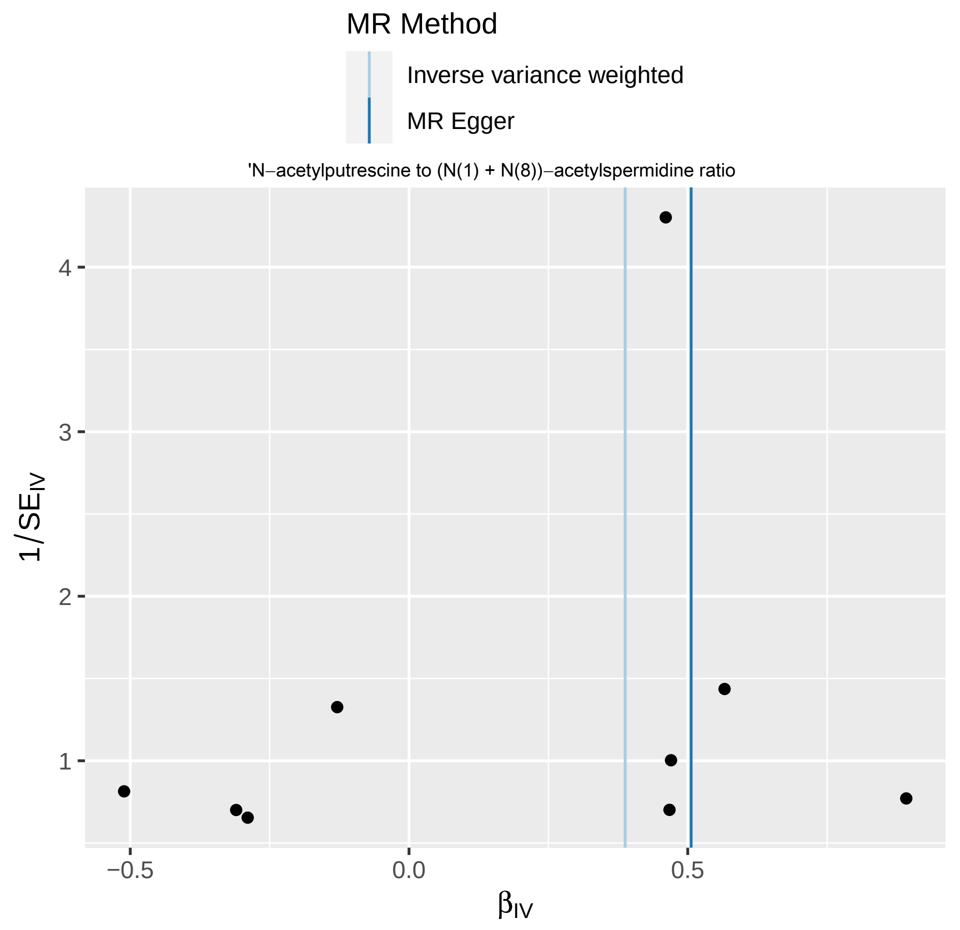

MR Method
Inverse variance weighted
MR Egger
'N−acetylputrescine to (N(1) + N(8))−acetylspermidine ratio
1/SE$_{IV}$
4
3
2
1
−0.5
0.0
0.5
β$_{IV}$

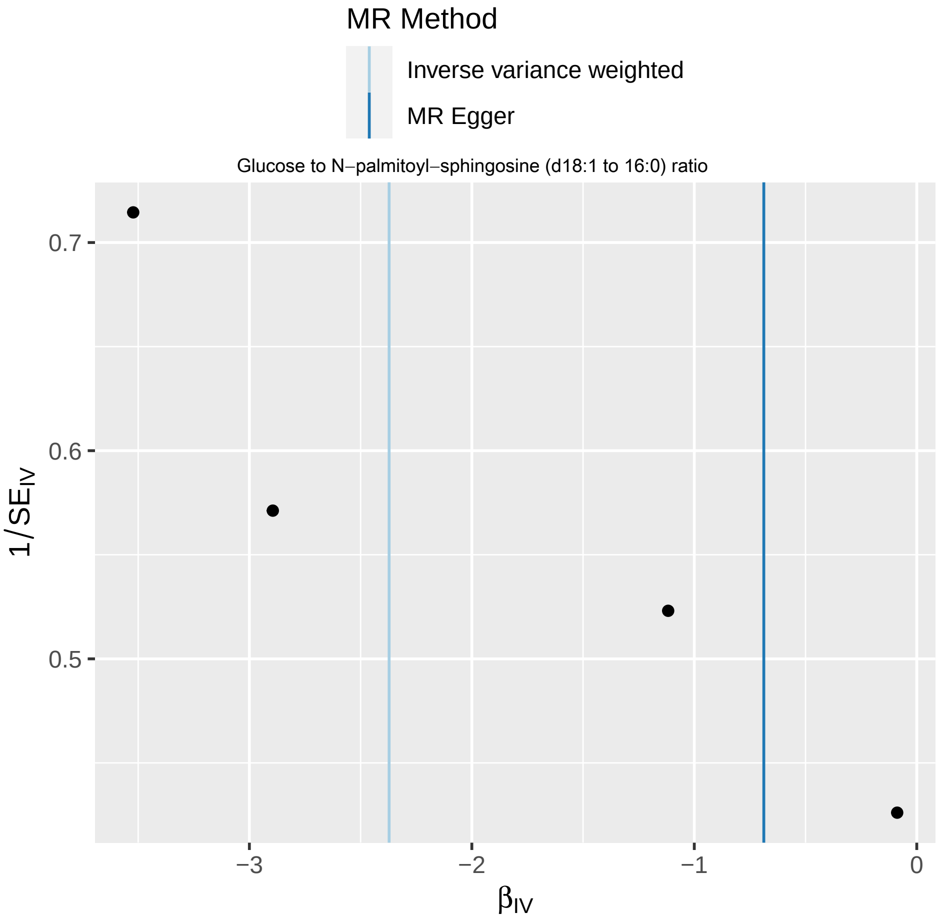
MR Method
Inverse variance weighted
MR Egger
Glucose to N−palmitoyl−sphingosine (d18:1 to 16:0) ratio
0.7
0.6
0.5
−3
−2
−1
0
$1/SE_{IV}$
β_{IV}

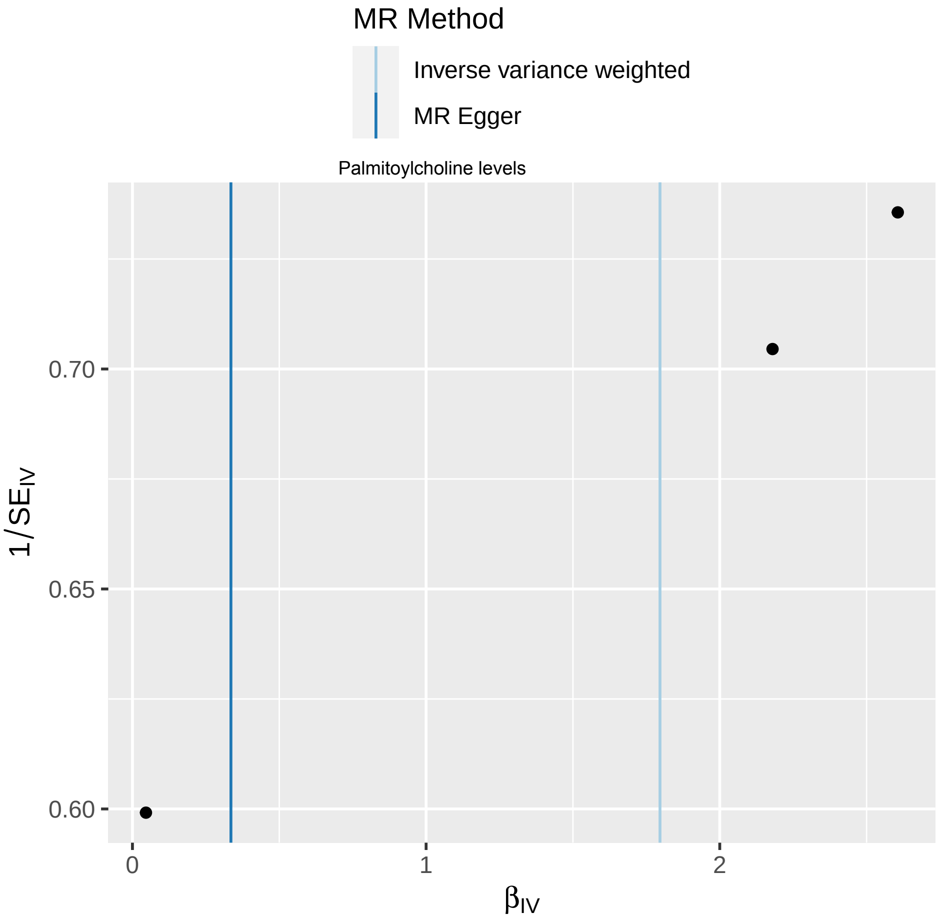
MR Method
Inverse variance weighted
MR Egger
Palmitoylcholine levels
0.70
0.65
0.60
0
1
2
1/SE_IV
β_IV

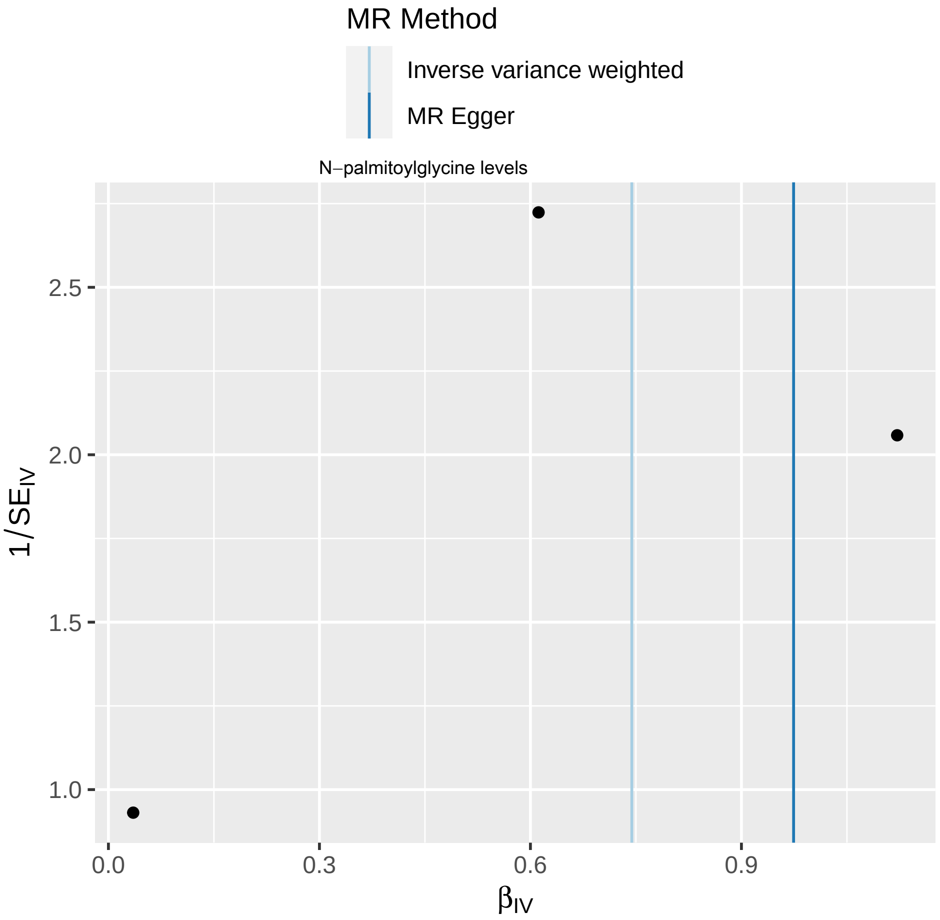
MR Method
Inverse variance weighted
MR Egger
N−palmitoylglycine levels
2.5
2.0
1.5
1.0
0.0
0.3
0.6
0.9
$1/SE_{IV}$
β_{IV}

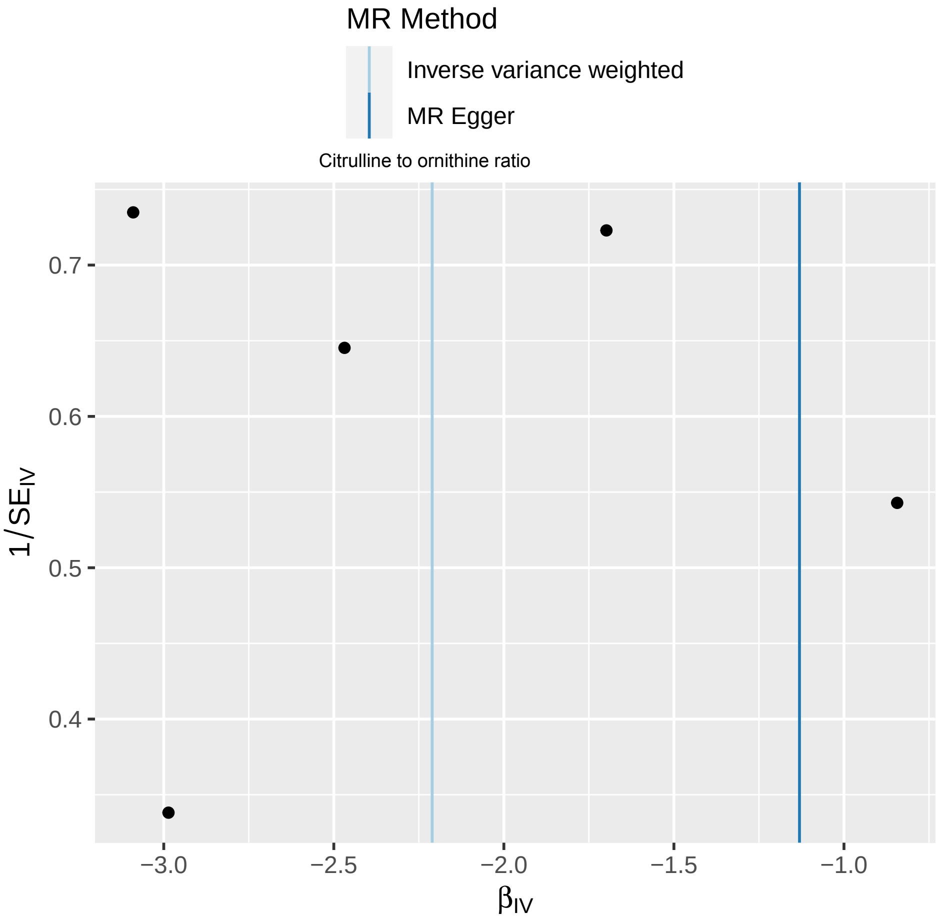

MR Method
Inverse variance weighted
MR Egger
Citrulline to ornithine ratio
0.7
0.6
0.5
0.4
1/SE$_{IV}$
−3.0
−2.5
−2.0
−1.5
−1.0
β$_{IV}$

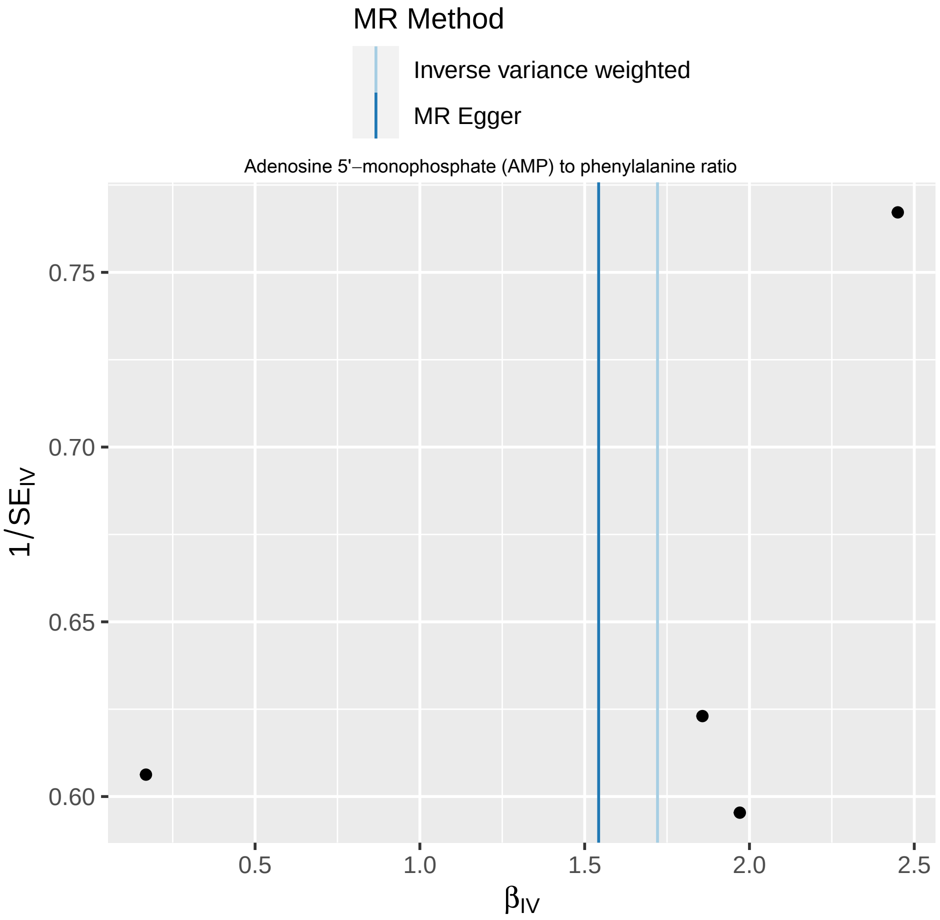

MR Method
Inverse variance weighted
MR Egger
Adenosine 5'−monophosphate (AMP) to phenylalanine ratio
0.75
0.70
0.65
0.60
1/SE_IV
0.5
1.0
1.5
2.0
2.5
β_IV

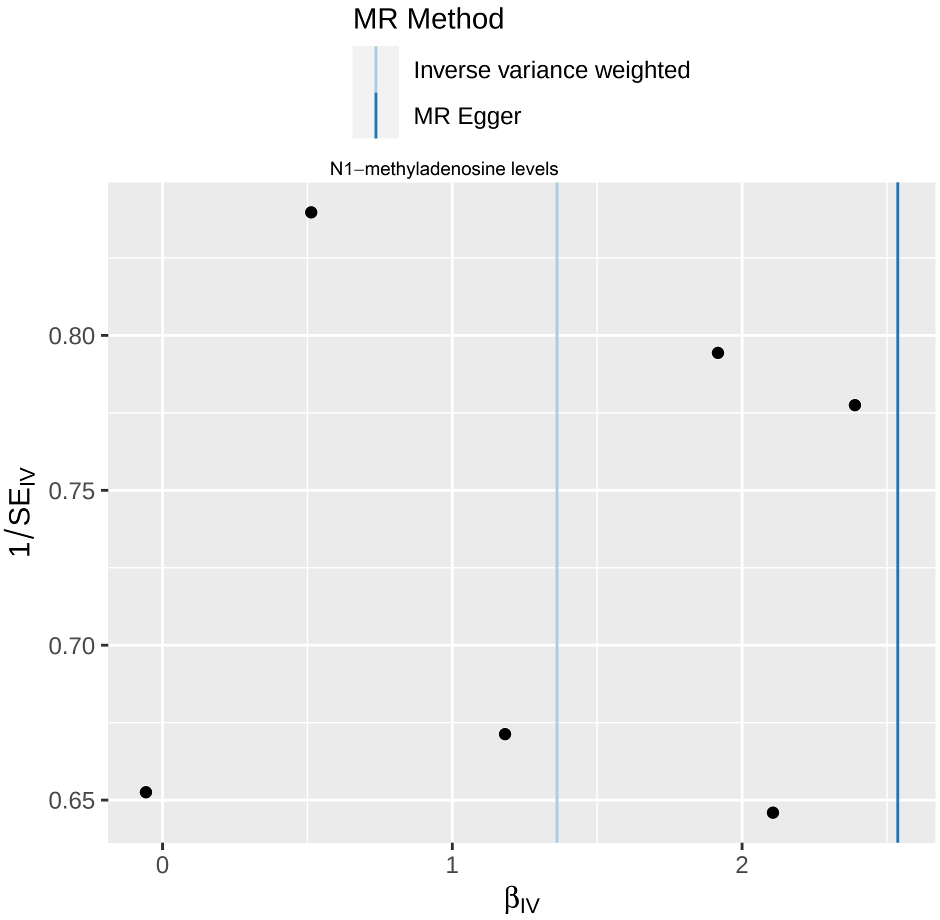

MR Method
Inverse variance weighted
MR Egger
N1−methyladenosine levels
0.80
0.75
0.70
0.65
1/SE$_{IV}$
0
1
2
β$_{IV}$

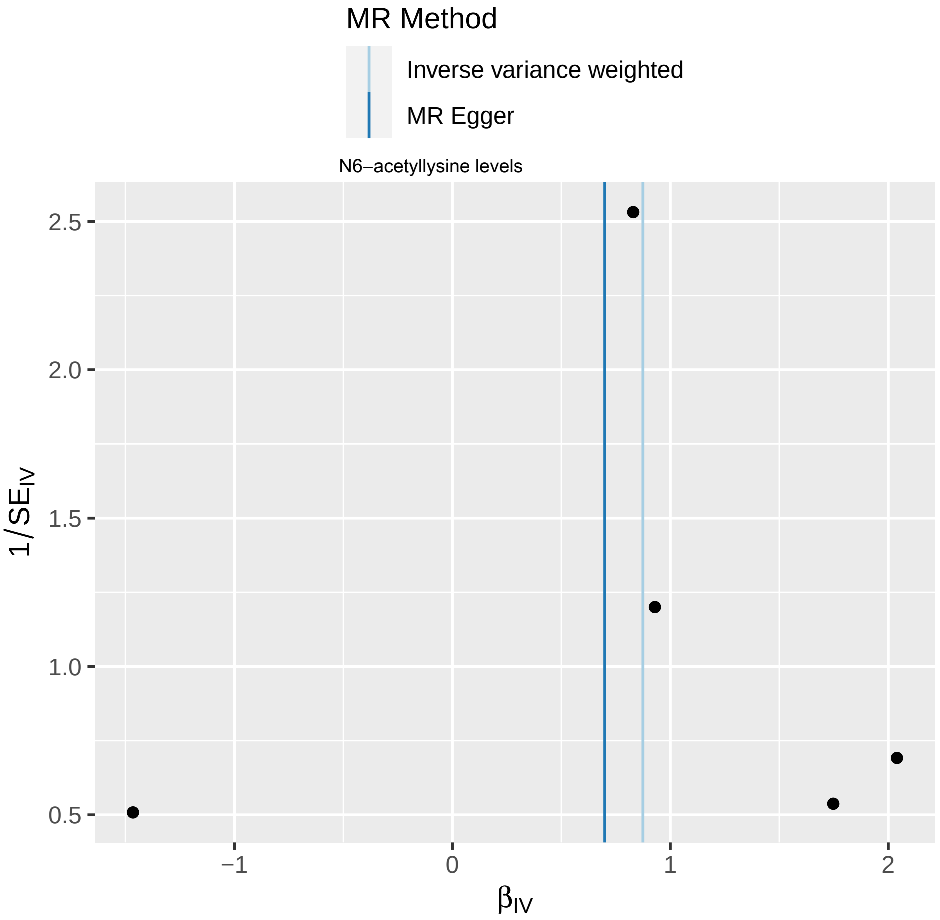
MR Method
Inverse variance weighted
MR Egger
N6−acetyllysine levels
2.5
2.0
1.5
1.0
0.5
1/SE$_{IV}$
−1
0
1
2
β$_{IV}$

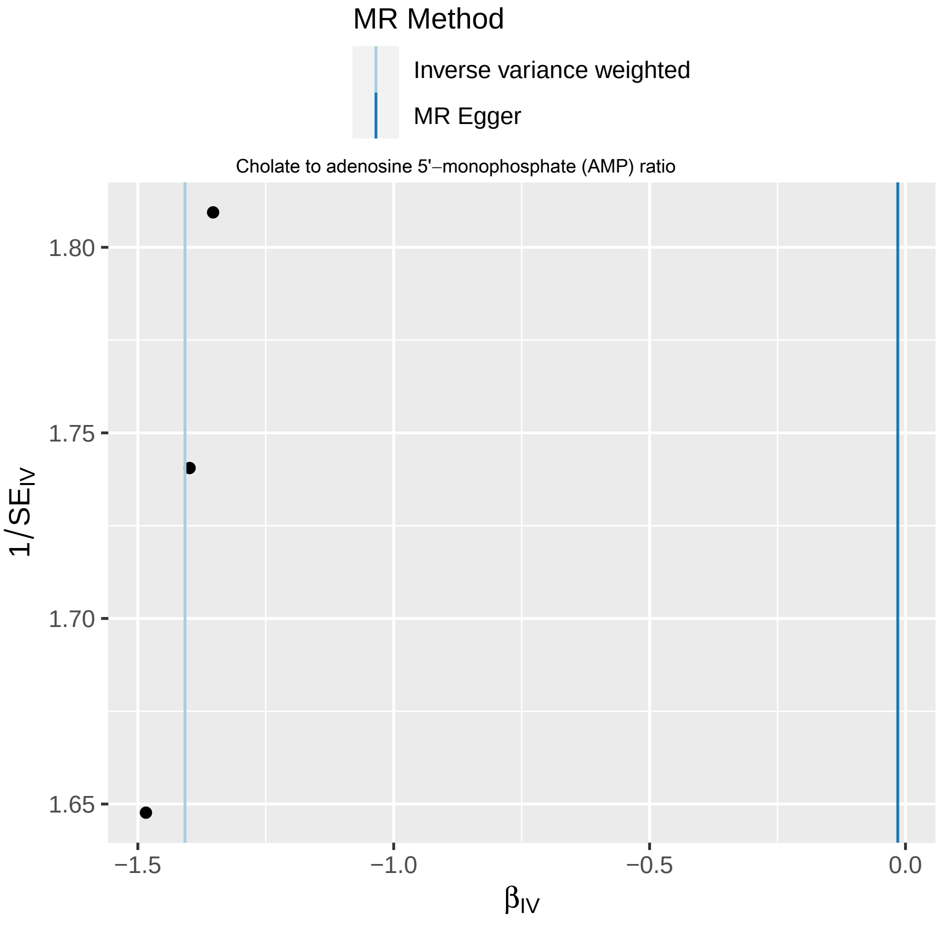

MR Method
Inverse variance weighted
MR Egger
Cholate to adenosine 5'−monophosphate (AMP) ratio
1.80
1.75
1.70
1.65
$1/SE_{IV}$
−1.5
−1.0
−0.5
0.0
β_{IV}

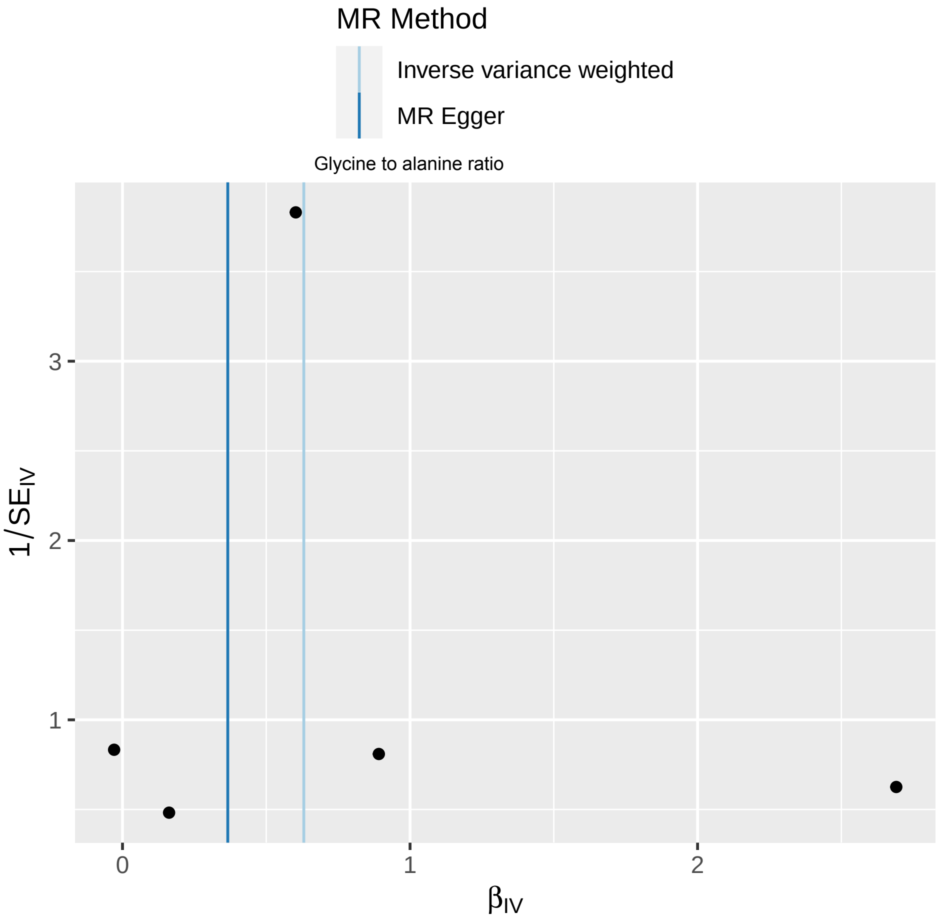
MR Method
Inverse variance weighted
MR Egger
Glycine to alanine ratio
$1/SE_{IV}$
3
2
1
0
1
2
β_{IV}

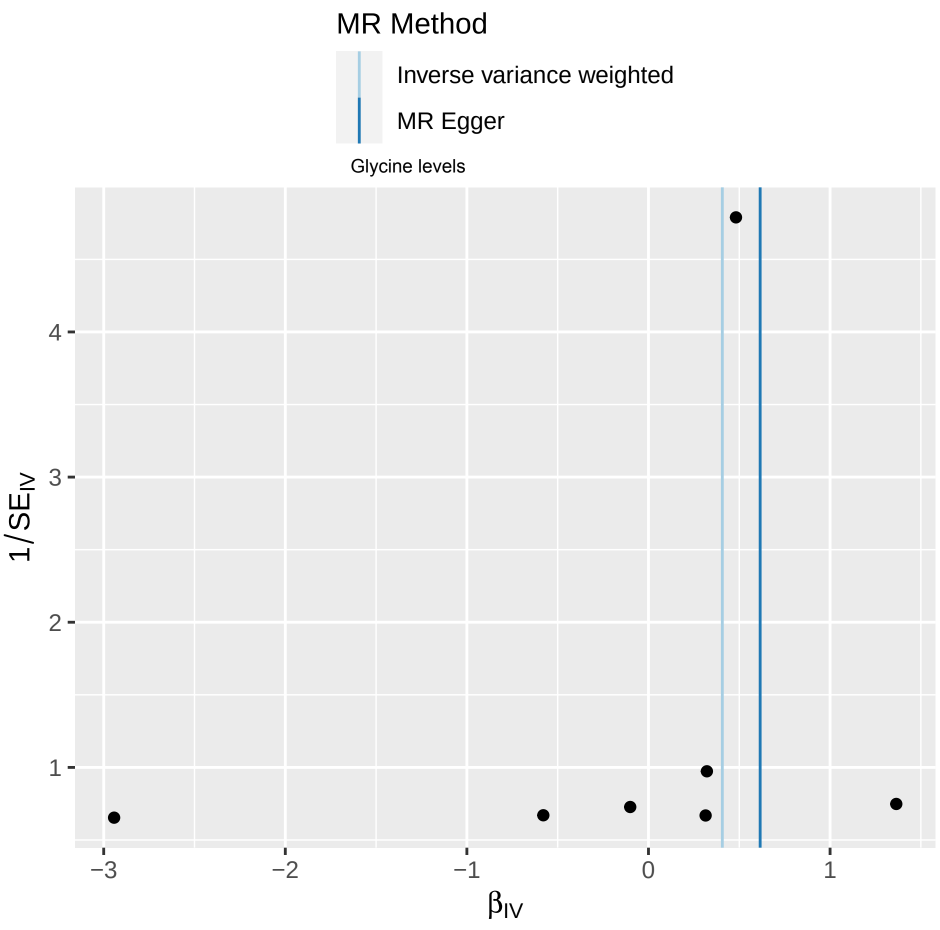

MR Method
Inverse variance weighted
MR Egger
Glycine levels
1/SE$_{IV}$
4
3
2
1
−3
−2
−1
0
1
β_{IV}

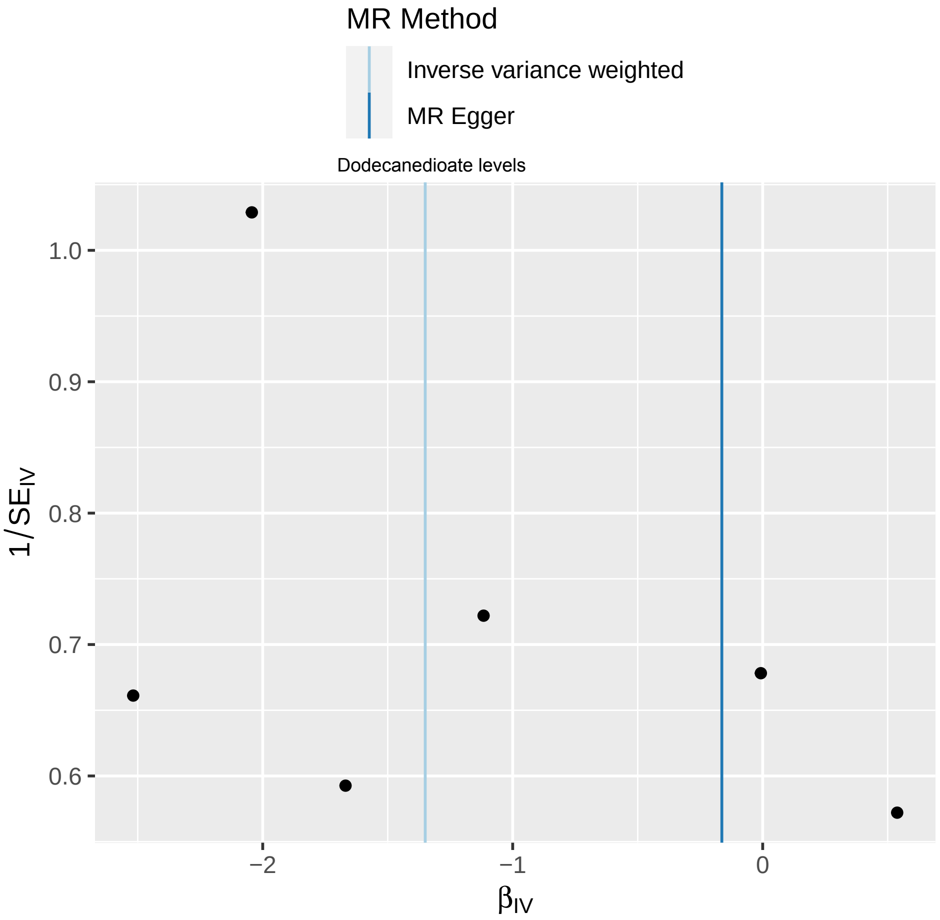
MR Method
Inverse variance weighted
MR Egger
Dodecanedioate levels
1.0
0.9
0.8
0.7
0.6
−2
−1
0
β_{IV}
$1/SE_{IV}$

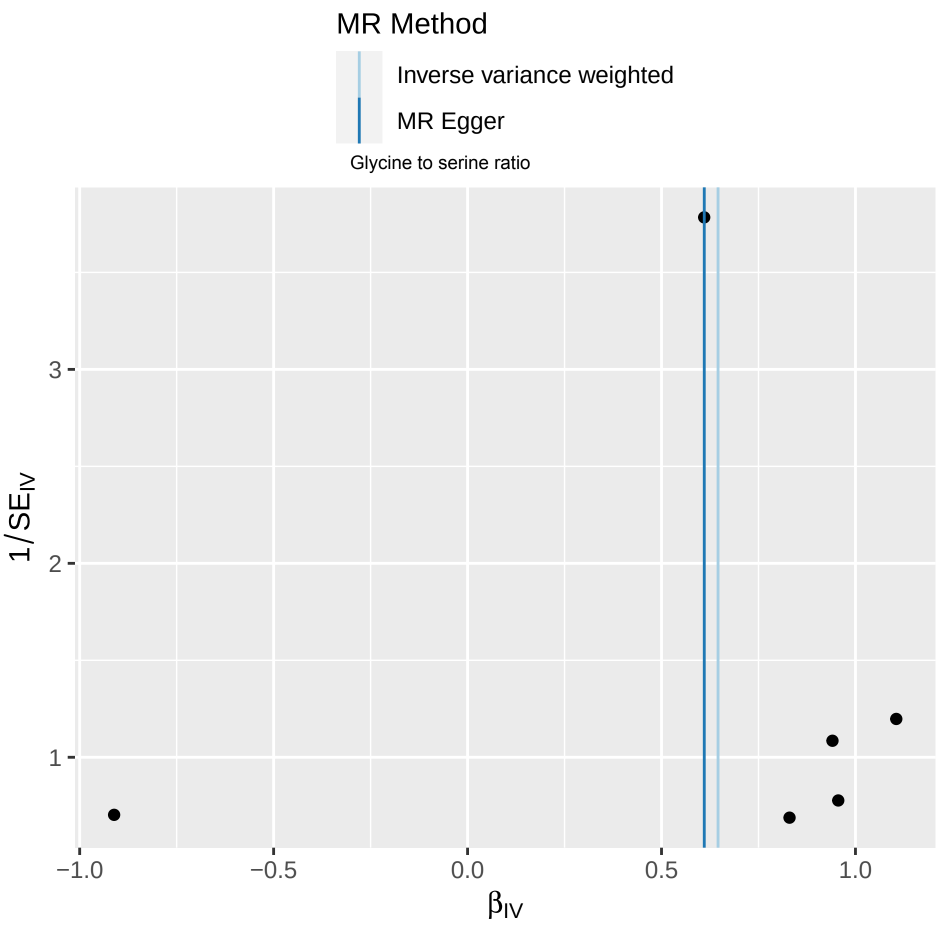

MR Method
Inverse variance weighted
MR Egger
Glycine to serine ratio
1/SE_IV
3
2
1
−1.0
−0.5
0.0
0.5
1.0
β_IV

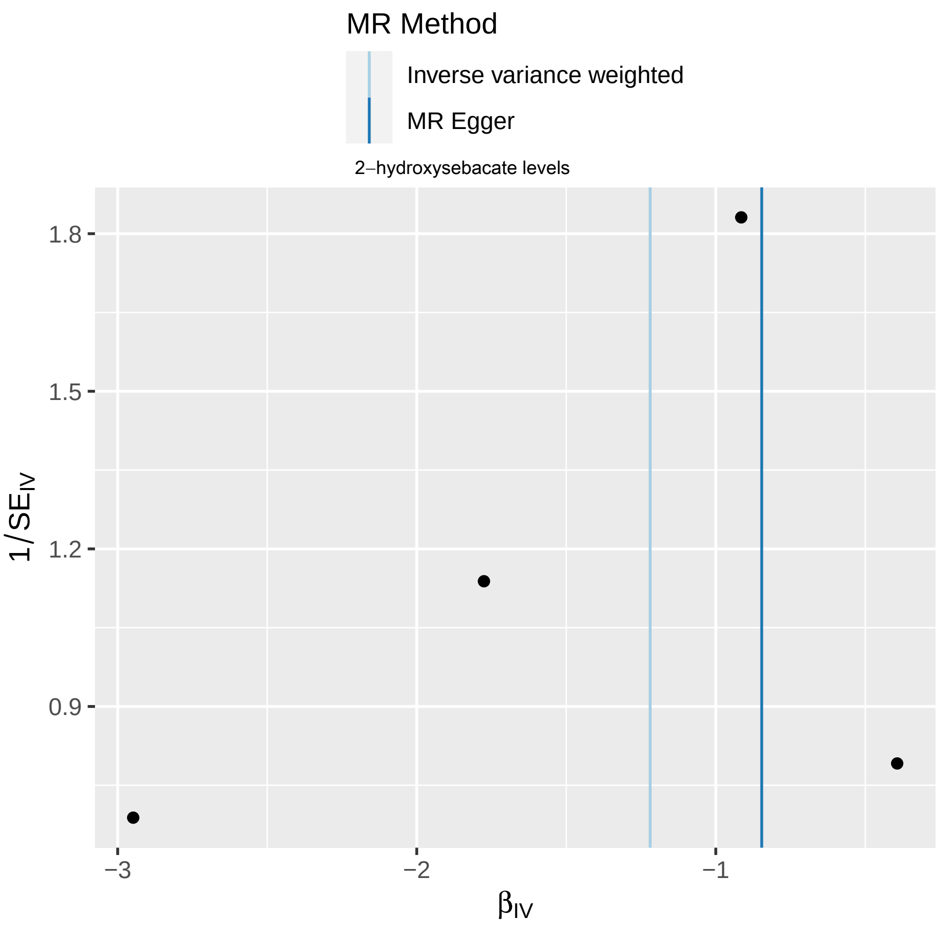
MR Method
Inverse variance weighted
MR Egger
2−hydroxysebacate levels
1.8
1.5
1.2
0.9
−3
−2
−1
β_{IV}
$1/SE_{IV}$

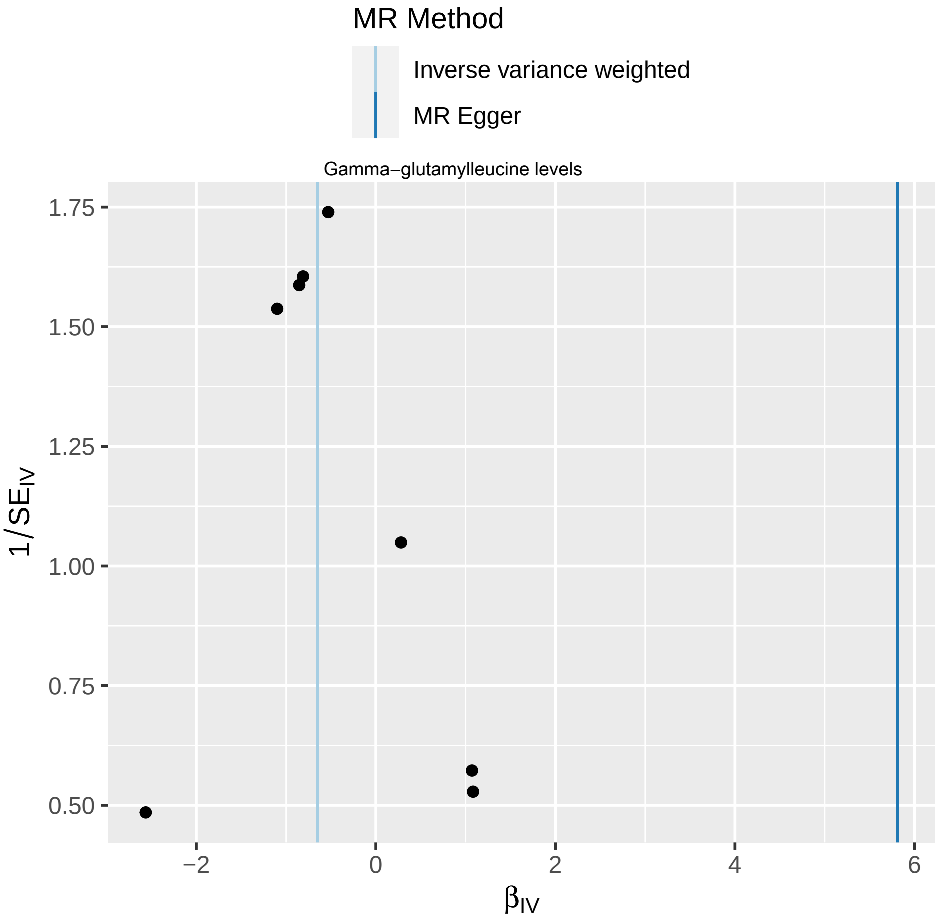
MR Method
Inverse variance weighted
MR Egger
Gamma−glutamylleucine levels
1.75
1.50
1.25
1.00
0.75
0.50
1/SE_IV
−2
0
2
4
6
β_IV

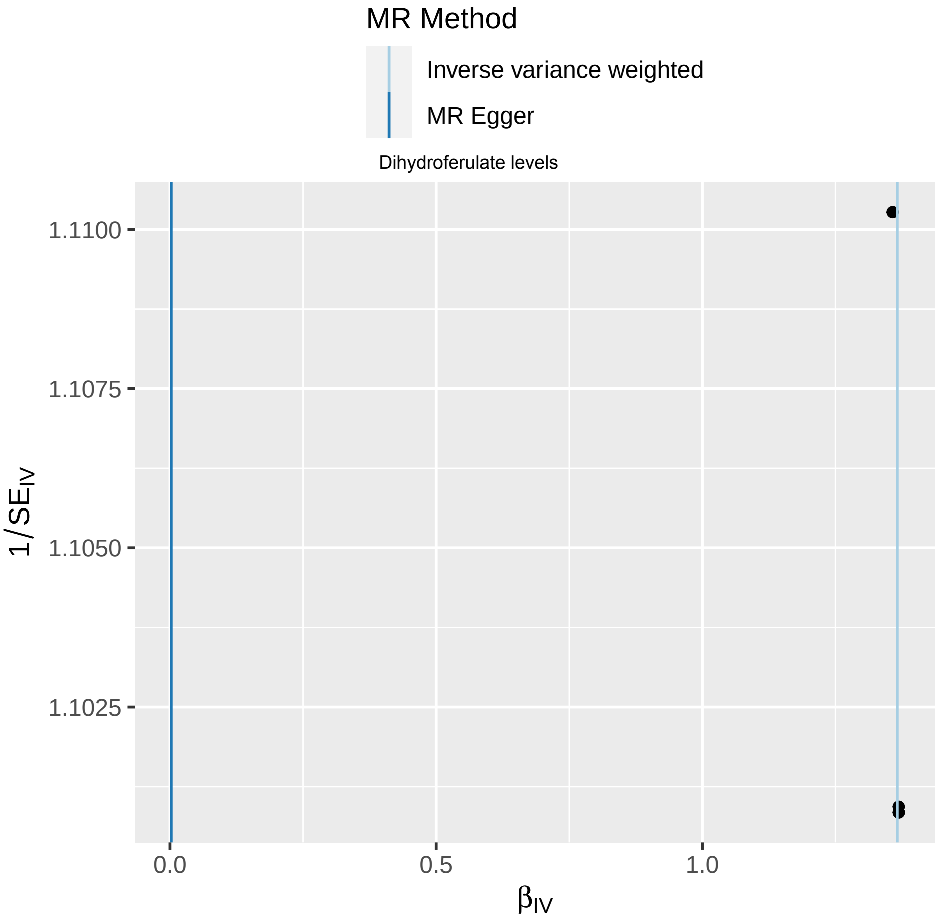
MR Method
Inverse variance weighted
MR Egger
Dihydroferulate levels
1.1100
1.1075
1.1050
1.1025
1/SE_IV
0.0
0.5
1.0
β_IV

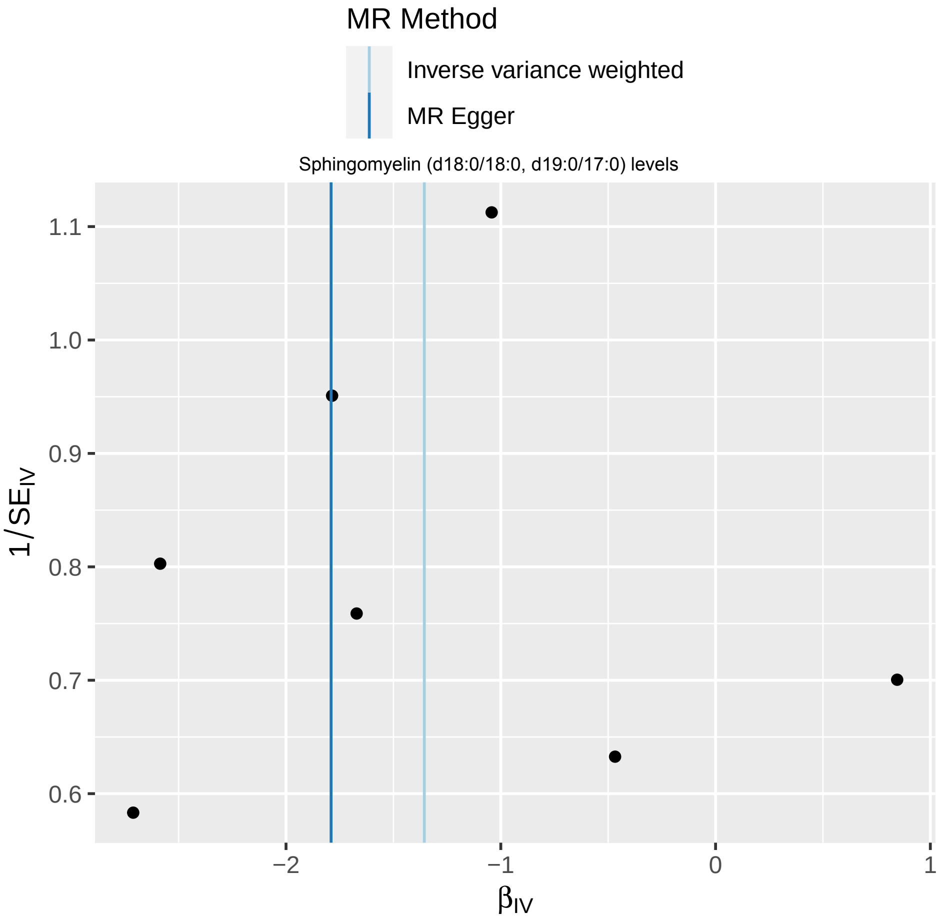

MR Method
Inverse variance weighted
MR Egger
Sphingomyelin (d18:0/18:0, d19:0/17:0) levels
1.1
1.0
0.9
0.8
0.7
0.6
1/SE_IV
−2
−1
0
1
β_IV

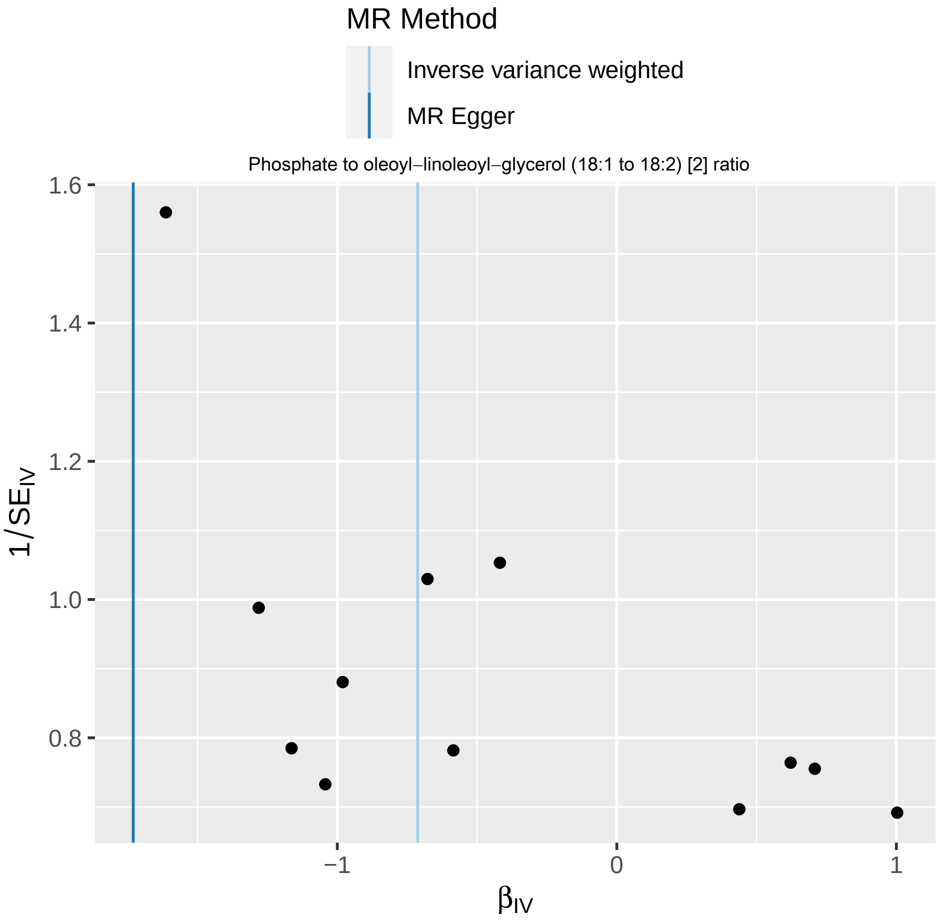
MR Method
Inverse variance weighted
MR Egger
Phosphate to oleoyl−linoleoyl−glycerol (18:1 to 18:2) [2] ratio
1.6
1.4
1.2
1.0
0.8
1/SE$_{IV}$
−1
0
1
β$_{IV}$

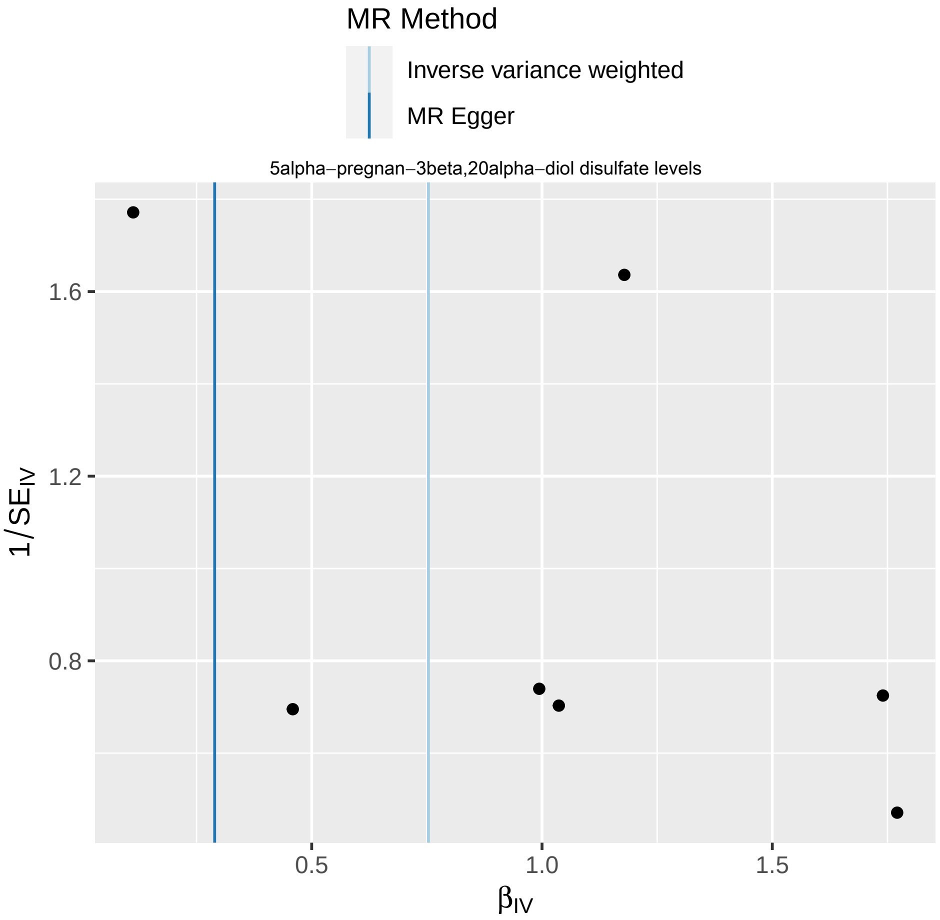

MR Method
Inverse variance weighted
MR Egger
5alpha−pregnan−3beta,20alpha−diol disulfate levels
1.6
1.2
0.8
0.5
1.0
1.5
$1/SE_{IV}$
β_{IV}

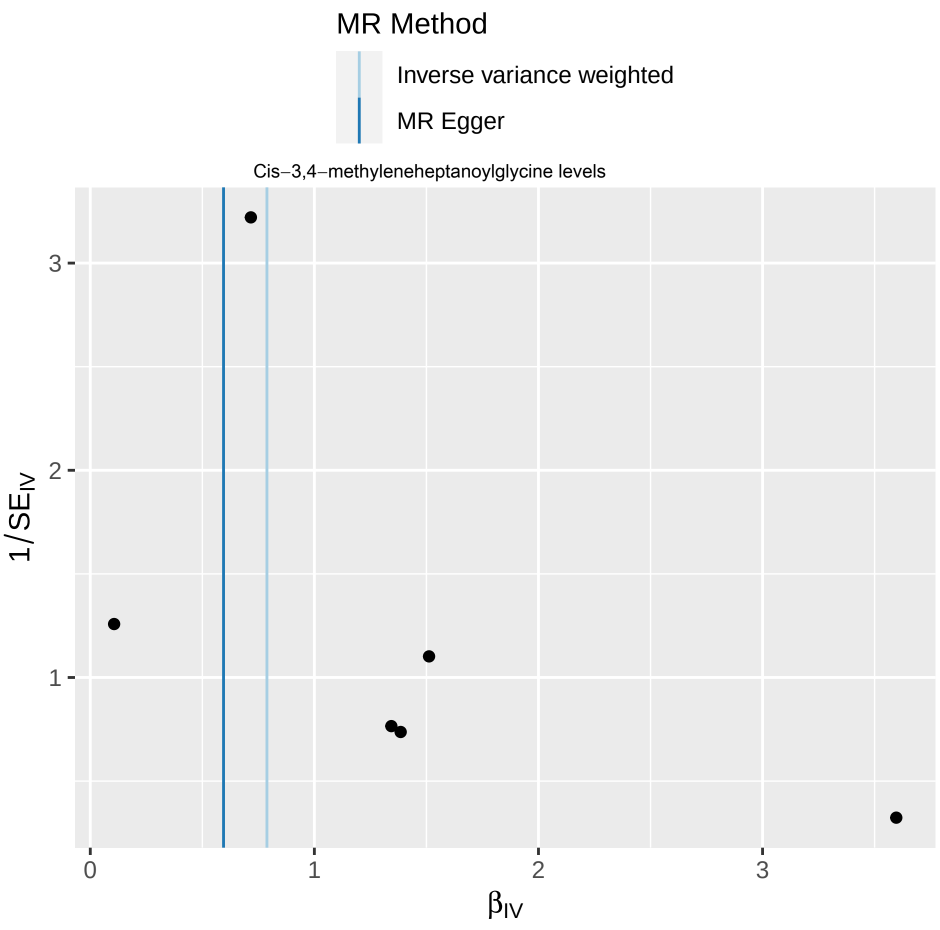
MR Method
Inverse variance weighted
MR Egger
Cis−3,4−methyleneheptanoylglycine levels
1/SE_IV
β_IV
0
1
2
3
1
2
3

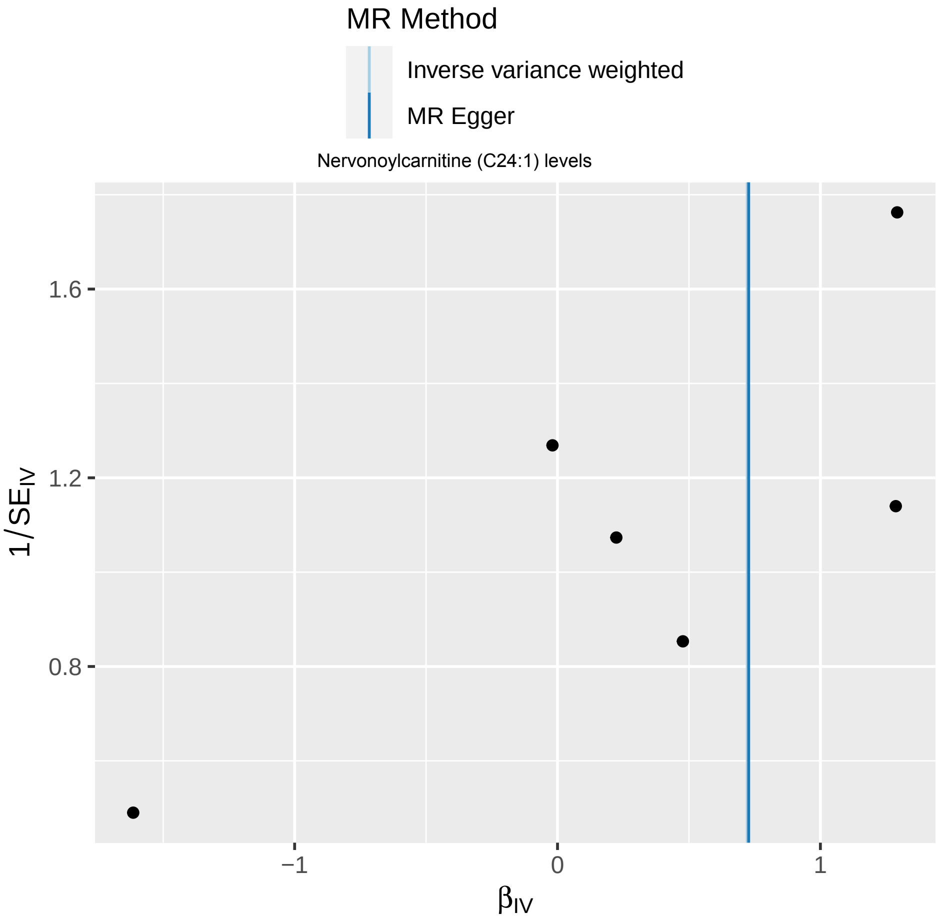

MR Method
Inverse variance weighted
MR Egger
Nervonoylcarnitine (C24:1) levels
1.6
1.2
0.8
1/SE_IV
−1
0
1
β_IV

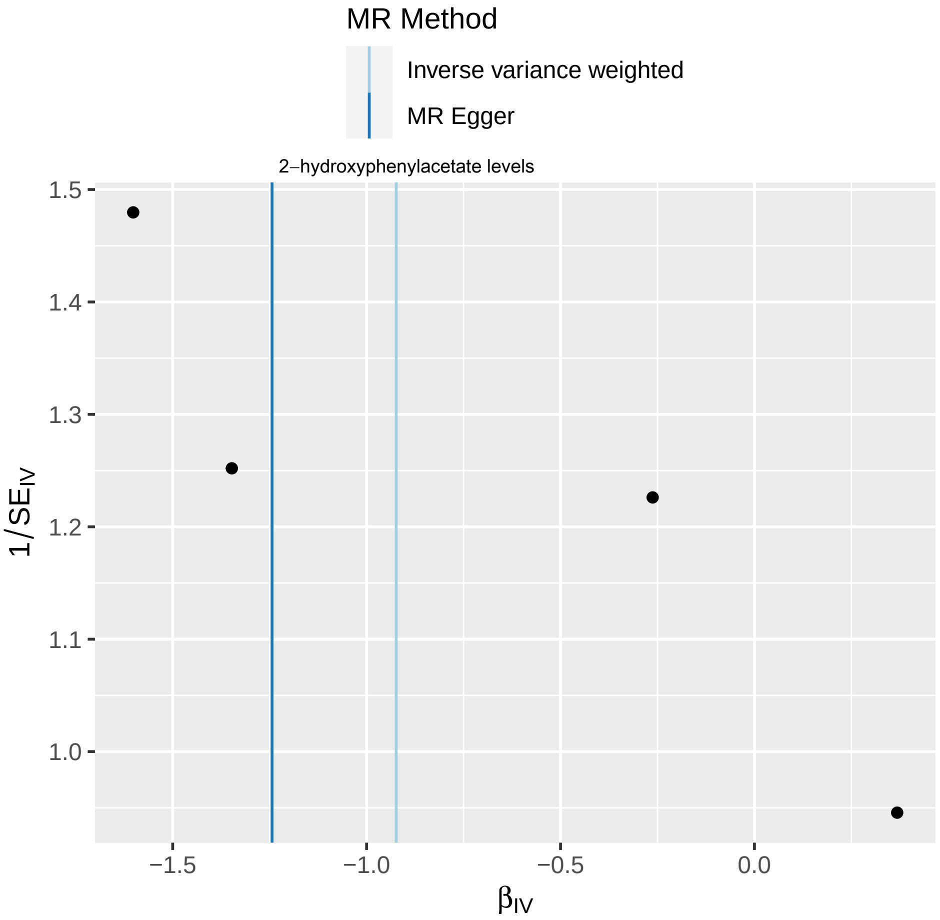

MR Method
Inverse variance weighted
MR Egger
2−hydroxyphenylacetate levels
1.5
1.4
1.3
1.2
1.1
1.0
$1/SE_{IV}$
−1.5
−1.0
−0.5
0.0
β_{IV}

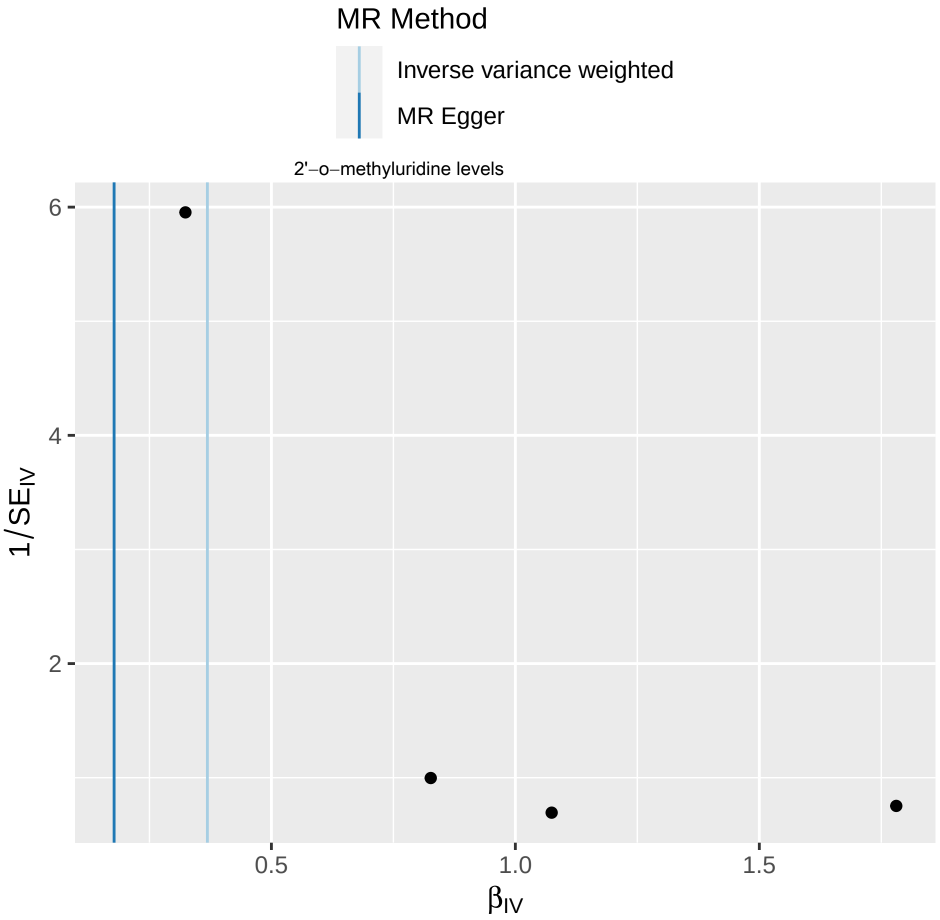

MR Method
Inverse variance weighted
MR Egger
2'−o−methyluridine levels
6
4
2
1/SE$_{IV}$
0.5
1.0
1.5
β$_{IV}$

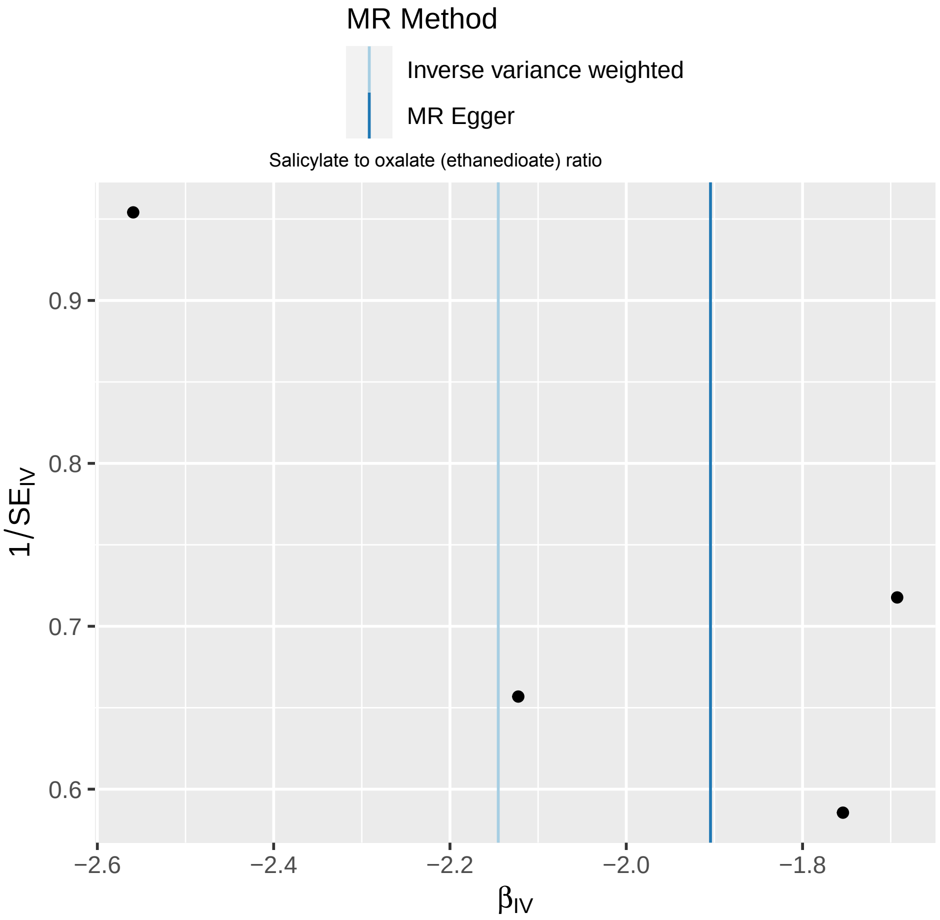
MR Method
Inverse variance weighted
MR Egger
Salicylate to oxalate (ethanedioate) ratio
1/SE_IV
0.9
0.8
0.7
0.6
−2.6
−2.4
−2.2
−2.0
−1.8
β_IV

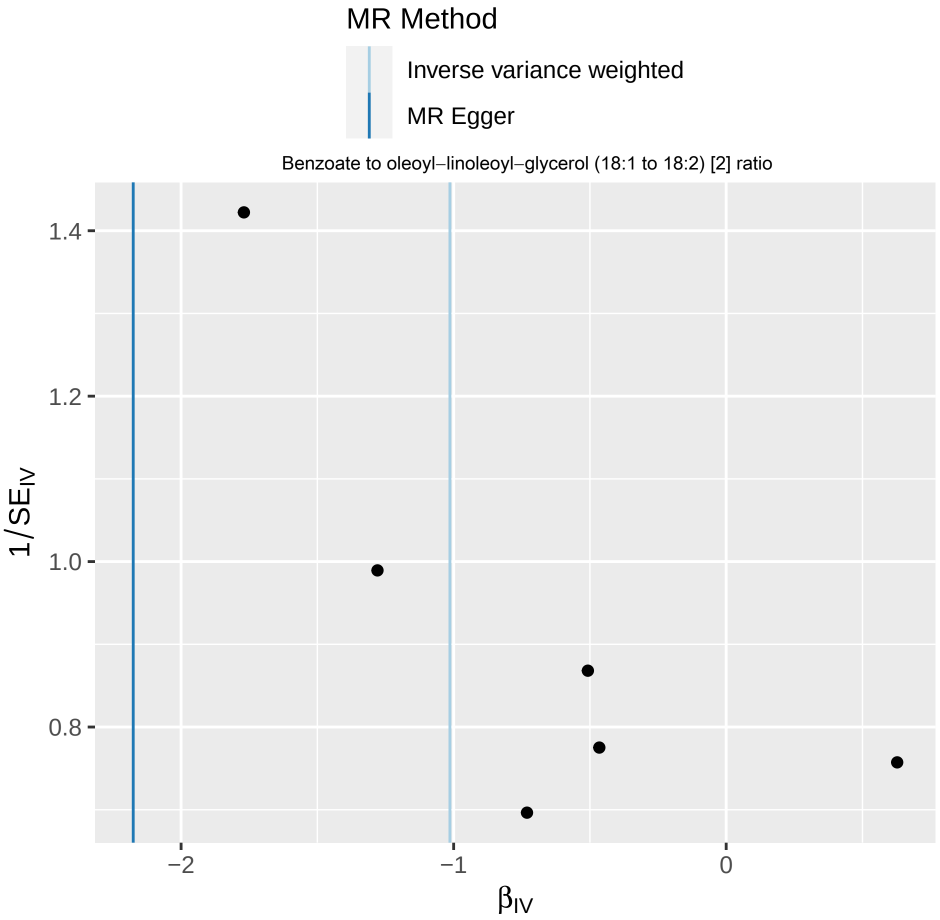
MR Method
Inverse variance weighted
MR Egger
Benzoate to oleoyl−linoleoyl−glycerol (18:1 to 18:2) [2] ratio
1.4
1.2
1.0
0.8
1/SE$_{IV}$
−2
−1
0
β$_{IV}$

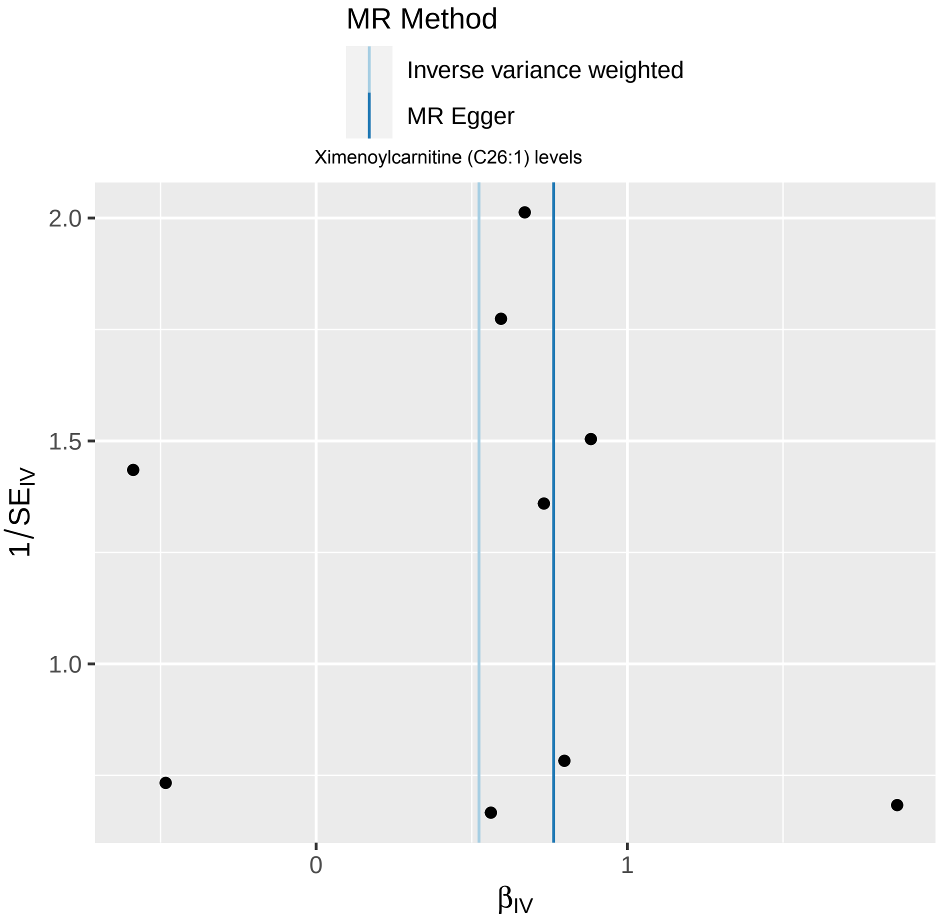

MR Method
Inverse variance weighted
MR Egger
Ximenoylcarnitine (C26:1) levels
2.0
1.5
1.0
1/SE_IV
0
1
β_IV

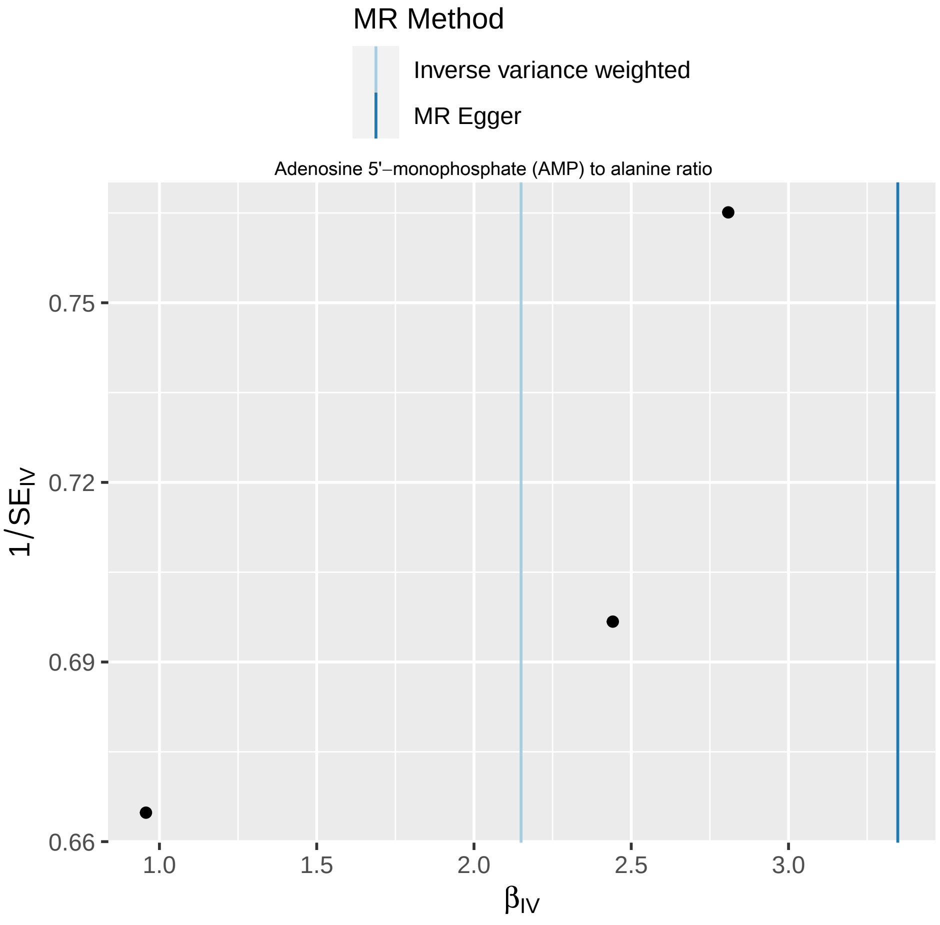

MR Method
Inverse variance weighted
MR Egger
Adenosine 5'−monophosphate (AMP) to alanine ratio
0.75
0.72
0.69
0.66
1/SE_IV
1.0
1.5
2.0
2.5
3.0
β_IV

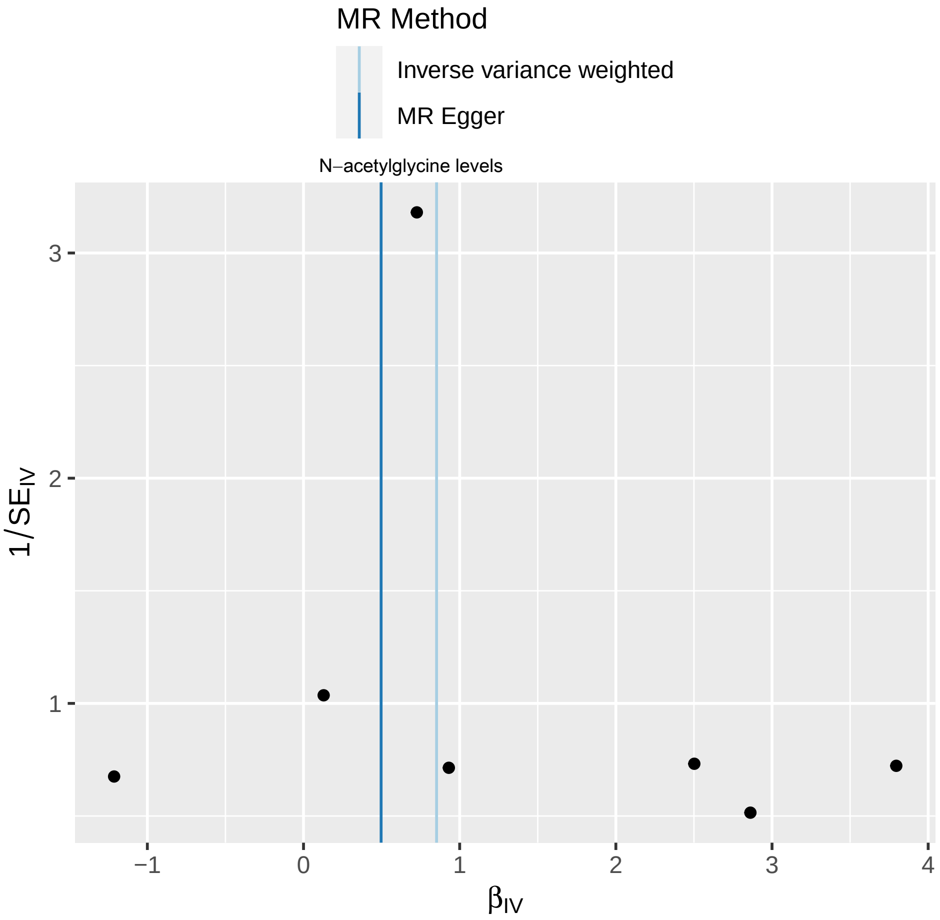

MR Method
Inverse variance weighted
MR Egger
N−acetylglycine levels
3
2
1
1/SE_IV
−1
0
1
2
3
4
β_IV

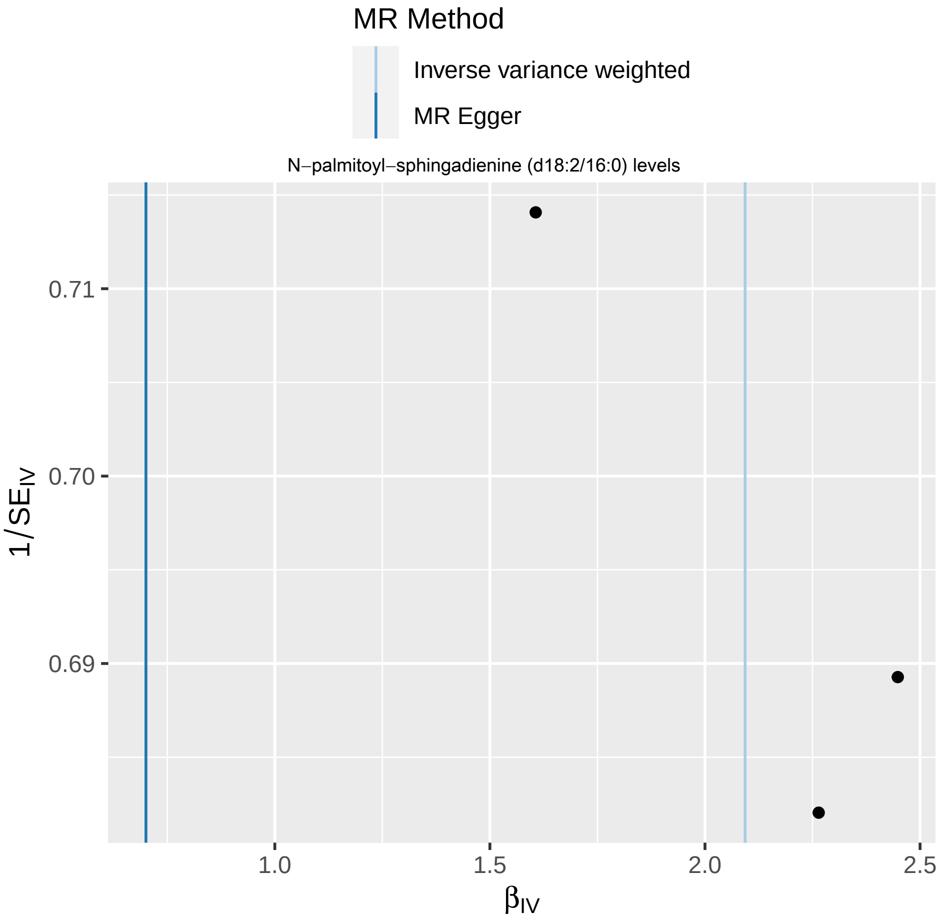

MR Method
Inverse variance weighted
MR Egger
N−palmitoyl−sphingadienine (d18:2/16:0) levels
0.71
0.70
0.69
1/SE$_{IV}$
1.0
1.5
2.0
2.5
β_{IV}
